# Conservation of Nucleosome Positions in Duplicated and Orthologous Gene Pairs

**DOI:** 10.1100/2012/298174

**Published:** 2012-02-15

**Authors:** Hiromi Nishida

**Affiliations:** Agricultural Bioinformatics Research Unit, Graduate School of Agriculture and Life Sciences, The University of Tokyo, Bunkyo-ku, Tokyo 113-8657, Japan

## Abstract

Although nucleosome positions tend to be conserved in gene promoters, whether they are conserved in duplicated and orthologous genes is unknown. In order to elucidate how nucleosome positions are conserved between duplicated and orthologous gene pairs, I performed 2 comparative studies. First, I compared the nucleosome position profiles of duplicated genes in the filamentous ascomycete *Aspergillus fumigatus*. After identifying 63 duplicated gene pairs among 9630 protein-encoding genes, I compared the nucleosome position profiles of the paired genes. Although nucleosome positions are conserved more in gene promoters than in gene bodies, their profiles were diverse, suggesting evolutionary changes after gene duplication. Next, I examined the conservation of nucleosome position profiles in 347 *A. fumigatus* orthologs of *S. cerevisiae* genes that showed notably high conservation of nucleosome positions between the parent strain and 2 deletion mutants. In only 11 (3.2%) of the 347 gene pairs, the nucleosome position profile was highly conserved (Spearman's rank correlation coefficient > 0.7). The absence of nucleosome position conservation in promoters of orthologous genes suggests organismal specificity of nucleosome arrangements.

## 1. Introduction

Nucleosomes are histone octamers around which DNA is wrapped in 1.65 turns [1]. Neighboring nucleosomes are separated by unwrapped linker DNA. Nucleosome density is lower, and nucleosome position is more conserved in the promoters than in the bodies of genes [2–5]. It is thought that nucleosome positioning in the gene promoter plays an important role in transcriptional regulation.

Although nucleosome positions can be partially simulated using a DNA-sequence-based approach [6], these simulations are limited due to variations between species. The nucleosome positioning mechanism varies between the 2 ascomycetous yeasts, *Saccharomyces cerevisiae,* and *Schizosaccharomyces pombe* [7]. Nucleosome positioning differs even among phylogenetically close ascomycetous yeast species [5].

Gene duplication is a driving force behind gene creation, and generating novel functions in newly created genes. Approximately one-half of cellular functions have been gained through gene duplication [8]. The duplicated genes encode similar amino acid sequences and often similar protein functions. It is uncertain, however, whether duplicated genes have similar nucleosome position profiles. In this study, I compared nucleosome positions in the promoter and body regions of duplicated gene pairs in the filamentous ascomycete *Aspergillus fumigatus*.

Previous analyses have found that nucleosome positions in *A. fumigatus* are conserved more in gene promoters than in gene bodies, even after treatment with the histone deacetylase inhibitor trichostatin A [4, 9]. In addition, nucleosome positions in *S. cerevisiae* are more conserved in gene promoters than in gene bodies between the control and the histone acetyltransferase gene *ELP3* deletion mutant, and between the control and the histone deacetylase gene *HOS2* deletion mutant [10]. The proteins Elp3 and Hos2 show the highest and the third highest evolutionary conservation, respectively, among the fungal histone modification proteins [11].

How well are nucleosome positions conserved in genes of the same origins? If there is a “nucleosome position code” that regulates nucleosome positioning, common nucleosome positions should remain in the promoters of orthologous genes across distinct species. In this study, I compared nucleosome positions in the promoters of duplicated and orthologous genes in *A. fumigatus* and *S. cerevisiae*.

## 2. Materials and Methods

### 2.1. Identification of Duplicated Gene Pairs in *Aspergillus fumigates*

Protein-coding gene pairs aligned over more than 80% of query length and more than 70% aminoacid sequence identity were selected by performing a BLAST search of 9630 *A. fumigatus* proteins at Fungal Genomes Central on NCBI (http://www.ncbi.nlm.nih.gov/projects/genome/guide/fungi/). Pairs in which the lengths differ by more than 25% were not used. Thus, we identified 63 duplicated *A. fumigatus* gene pairs (Tables 1 and 2).

### 2.2. Identification of Orthologous Gene Pairs in *Aspergillus fumigates* and *Saccharomyces cerevisiae*

In a comparison of nucleosome positioning between *A. fumigatus *and* S. cerevisiae*, I focused on 466 genes (Table 3) that showed notably high conservation of nucleosome positioning in the promoters of the control and the *ELP3 *and* HOS2* deletion mutants from the previous study [10].

A total of 3339 ortholog clusters were identified (See table 1 in Supplementary Material available at doi: 10.1100/2012/298174) between *A. fumigatus *and* S. cerevisiae* by ortholog cluster analysis in the Microbial Genome Database for Comparative Analysis (MBGD, http://mbgd.nibb.ac.jp/) [12]. Of these orthologous gene pairs, 347 (Table 4) are yeast genes that showed a high level of nucleosome positioning conservation in the control and deletion mutants. I focused on these 347 orthologous pairs to compare nucleosome positioning between species. The same number of pairs of *A. fumigatus* and *S. cerevisiae* genes chosen at random were used as a control.

### 2.3. Nucleosome Position Profile

Nucleosome mapping numbers at each genomic position were determined [13] based on genome-wide nucleosome mapping data for *A. fumigatus* [9] and *S. cerevisiae* [10]. In this analysis, a 1-kb region upstream of the translational start site was defined as a gene promoter. When the length of the gene body region is more than 1 kb, a 1-kb region downstream of the translational start site was defined as the gene body. When the length of the gene body is less than 1 kb, the region between the translational start and end sites was defined as the gene body. Analyses of nucleosome position data including calculation of Spearman's rank correlation coefficient were performed using the statistics software R (http://www.r-project.org/).

## 3. Results and Discussion

### 3.1. Nucleosome Position Profiles of Duplicated Genes in *Aspergillus fumigates*

**Figure 1:**
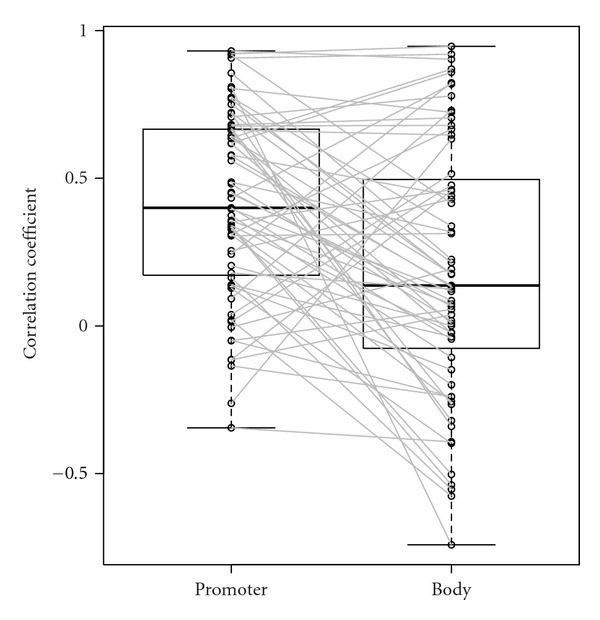
Boxplots of Spearman's rank correlation coefficients of nucleosome position profiles in the promoter and body regions of 63 duplicated gene pairs. Circles represent the correlation coefficients and values of the same genes are connected by lines.

I compared nucleosome position profiles in each of the 63 duplicated gene pairs. Nucleosome positioning was conserved more in gene promoters than in gene bodies (Figure 1), as observed in the comparison of nucleosome positioning between trichostatin A-treated and -untreated *A. fumigatus* [4]. This result suggests that nucleosome positioning in the gene promoter plays an important role in transcriptional regulation [14].

Single-gene duplications and gene cluster duplications consisting of multiple genes were identified. One cluster of 4 genes (*AFUA_1G00420* to *AFUA_1G00470*) is a duplication of another 4-gene cluster (*AFUA_8G04120* to *AFUA_8G04080*) (Table 2). Among these gene pairs, the nucleosome position profile was poorly conserved in the gene promoter between *AFUA_1G00470* and *AFUA_8G04080* and in the gene body between *AFUA_1G00440* and *AFUA_8G04110* (Spearman's rank correlation coefficients were 0.43 and 0.23, resp.) (Table 2). With the exception of these 2 cases, the nucleosome position profile was highly conserved (correlation coefficients were higher than 0.7) (Table 2).

We analyzed another pair of duplicated clusters (9 genes) (*AFUA_1G16030* to *AFUA_1G16120* and *AFUA_5G14930* to *AFUA_5G15030*). The genes in each cluster have evolved for the same period after the duplication (Table 2). At present, conservation of the nucleosome position profiles varies among the 9 genes (Table 2). For example, the nucleosome position profile is poorly conserved in the gene promoters of 3 gene pairs (*AFUA_1G16050* and *AFUA_5G14950*, *AFUA_16110* and *AFUA_15020*, *AFUA_1G16120* and *AFUA_15030*) (Spearman's rank correlation coefficients are −0.35, −0.26, and −0.14, resp.). On the other hand, the nucleosome position profile is highly conserved in the promoters of *AFUA_16070* and *AFUA_5G14980* and was strongly correlated (correlation coefficient = 0.93). These results suggest that transcriptional regulation of duplicated genes is associated with nucleosome positions in the gene promoters.

### 3.2. Nucleosome Position Profiles of Orthologous Gene Promoters in *Aspergillus fumigates* and *Saccharomyces cerevisiae*

**Figure 2:**
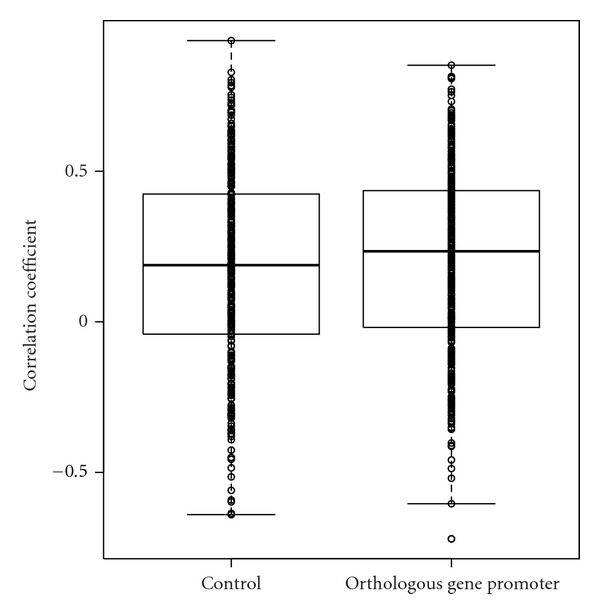
Boxplots of Spearman's rank correlation coefficients between nucleosome position profiles in the promoters of 347 orthologous gene pairs between *Aspergillus fumigatus* and *Saccharomyces cerevisiae*. The same number of gene pairs was chosen at random to serve as a control. Dots indicate correlation coefficients. The distributions of correlation coefficients did not significantly differ (*P-*value = 0.28 in Kolmogorov-Smirnov test) between the orthologous gene promoters and the controls.

I compared the nucleosome position profiles in the promoters of 347 orthologous pairs of yeast genes that showed notably high conservation in the control and mutant strains. In the 63 duplicated *A. fumigatus* gene pairs, 13 (20.6%) gene promoter profiles and 11 (17.5%) gene body profiles were highly correlated (Spearman's rank correlation coefficient > 0.7) (Table 1, Figure 1). On the other hand, of the 347 orthologous gene pairs, only 11 (3.2%) nucleosome position profiles were highly correlated (Spearman's rank correlation coefficient > 0.7) (Table 4, Figure 2). The distribution of correlation coefficients of the 347 orthologous gene promoters did not significantly differ from that of the control (gene pairs chosen at random) (*P-*value = 0.28 Kolmogorov-Smirnov test). One potential cause of this low conservation is the large evolutionary distance between the 2 fungi. *A. fumigatus* and *S. cerevisiae* belong to the subphyla Pezizomycotina and Saccharomycotina, respectively. Alternatively, this low conservation may represent a difference in mechanisms regulating the nucleosome arrangement, since the nucleosomal (nucleosome-bound) DNA lengths differ between the 2 fungi [9, 10].

**Figure 3:**
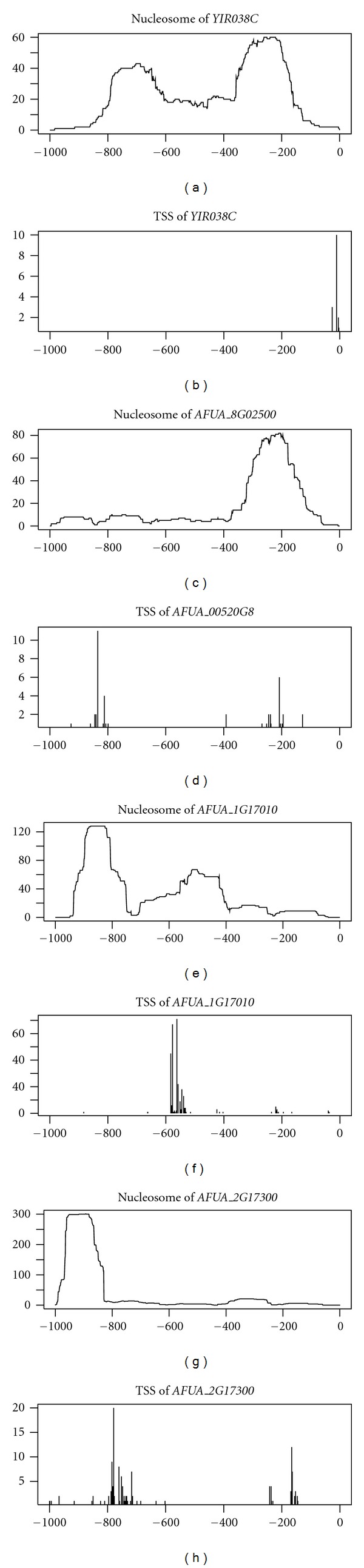
Mapping numbers of nucleosomes and transcription start sites in the promoter regions of *YIR038C*, *AFUA_8G02500*, *AFUA_1G17010*, and *AFUA_2G17300*. Position 0 indicates the translational start site.

Nucleosome position profiles in gene promoters are thought to be related to gene function. For example, *YIR038C* of *S. cerevisiae* encodes an amino acid sequence protein (glutathione S-transferase) similar to 3 genes (*AFUA_1G17010*, *AFUA_2G17300*, and *AFUA_8G02500*) in *A. fumigatus* (Table 4). Although the nucleosome position profiles show some conservation between *YIR038C* and *AFUA_8G02500* (Spearman's rank correlation coefficient = 0.55, except for one nucleosome position loss in *A. fumigatus*), they are poorly conserved between *YIR038C* and *AFUA_1G17010* (Spearman's rank correlation coefficient = −0.03) and between *YIR038C* and *AFUA_2G17300* (Spearman's rank correlation coefficient = −0.07) (Table 4, Figure 3).

Interestingly, although the nucleosome position profile of *AFUA_8G02500* is completely different from that of *AFUA_2G17300*, the transcription start site patterns are very similar between these genes (Figure 3), suggesting that the relationship between transcription start site and nucleosome position in the gene promoter varies.

## Supplementary Material

Ortholog cluster between *Aspergillus fumigatus* and *Saccharomyces cerevisiae* genes.

## Figures and Tables

**Table 1:** Duplicated gene pairs in *Aspergillus fumigatus*.

Gene pair	Chromosome	Gene body region		Gene direction	Function
*AFUA1G00150*	1	25442	27017	+	RING finger protein
*AFUA6G09370*	6	2245549	2247121	+	RING finger protein

*AFUA1G00420*	1	135528	137781	+	Carboxypeptidase S1, putative
*AFUA8G04120*	8	897824	900076	−	Carboxypeptidase S1, putative

*AFUA1G00440*	1	138359	140093	−	DUF895 domain membrane protein
*AFUA8G04110*	8	895512	897246	+	DUF895 domain membrane protein

*AFUA1G00450*	1	143117	144466	+	N-acetylglucosamine-6-phosphate deacetylase(NagA), putative
*AFUA8G04100*	8	891135	892484	−	N-acetylglucosamine-6-phosphate deacetylase(NagA), putative

*AFUA1G00470*	1	148615	150219	+	Betaine aldehyde dehydrogenase, putative
*AFUA8G04080*	8	885376	886980	−	Betaine aldehyde dehydrogenase (BadH), putative

*AFUA1G00530*	1	164101	164771	−	Thermoresistant gluconokinase family protein
*AFUA4G12050*	4	3163747	3164530	−	Thermoresistant gluconokinase

*AFUA1G00550*	1	177114	178593	+	Hypothetical protein
*AFUA1G00910*	1	328785	330274	+	Hypothetical protein

*AFUA1G00580*	1	184790	186873	+	Acid phosphatase (PhoG), putative
*AFUA8G04050*	8	870757	872487	−	Acid phosphatase (PhoG), putative

*AFUA1G00650*	1	215584	217006	+	Alpha-1,3-glucanase, putative
*AFUA7G08510*	7	1973398	1974759	+	Alpha-1,3-glucanase, putative

*AFUA1G00920*	1	331676	332955	−	Hypothetical protein
*AFUA3G06425*	3	1582748	1584037	−	Hypothetical protein

*AFUA1G01050*	1	385302	386244	−	Hypothetical protein
*AFUA8G06160*	8	1465695	1466634	+	Hypothetical protein

*AFUA1G02550*	1	744583	746335	−	Tubulin alpha-1 subunit
*AFUA2G14990*	2	3947008	3948834	−	Tubulin alpha-2 subunit

*AFUA1G02730*	1	788249	789373	−	Mitochondrial phosphate carrier protein (Ptp), putative
*AFUA1G15140*	1	4070230	4071449	−	Mitochondrial phosphate carrier protein (Mir1), putative

*AFUA1G05760*	1	1658382	1659697	−	Arsenite efflux transporter
*AFUA5G15010*	5	3882425	3883746	+	Arsenite permease (ArsB), putative

*AFUA1G05760*	1	1658382	1659697	−	Arsenite efflux transporter
*AFUA1G16100*	1	4378898	4380216	+	Arsenite permease (ArsB), putative

*AFUA1G10910*	1	2848155	2850137	−	Tubulin beta, putative
*AFUA7G00250*	7	70221	71948	+	Tubulin beta-2 subunit

*AFUA1G11260*	1	2971529	2971889	−	Conserved hypothetical protein
*AFUA6G00270*	6	79542	79886	−	Conserved hypothetical protein

*AFUA1G11610*	1	3060058	3060510	+	3-Dehydroquinate dehydratase, type II
*AFUA3G14850*	3	3929721	3930173	+	3-Dehydroquinate dehydratase, type II

*AFUA1G11890*	1	3129447	3131489	+	Serine palmitoyltransferase 2, putative
*AFUA6G00300*	6	85851	87692	−	Serine palmitoyltransferase 1, putative

*AFUA1G12850*	1	3398837	3400611	+	Nitrate transporter (nitrate permease)
*AFUA1G17470*	1	4782320	4783995	+	High-affinity nitrate transporter NrtB

*AFUA1G15970*	1	4338407	4339712	+	Aldo-keto reductase (AKR13), putative
*AFUA8G01560*	8	401581	402815	−	Aldo-keto reductase (YakC), putative

*AFUA1G16030*	1	4358866	4360256	−	Conserved hypothetical protein
*AFUA5G14930*	5	3863218	3864521	−	Conserved hypothetical protein

*AFUA1G16040*	1	4363493	4365310	+	Metalloreductase, putative
*AFUA5G14940*	5	3867797	3869542	+	Cell surface metalloreductase (FreA), putative

*AFUA1G16050*	1	4366573	4368232	+	Hypothetical protein
*AFUA5G14950*	5	3870635	3872465	+	Hypothetical protein

*AFUA1G16070*	1	4370346	4373694	+	Conserved hypothetical protein
*AFUA5G14980*	5	3874539	3877890	+	Conserved hypothetical protein

*AFUA1G16080*	1	4374579	4375298	−	Hypothetical protein
*AFUA5G14990*	5	3878797	3879579	−	Hypothetical protein

*AFUA1G16090*	1	4377842	4378249	−	Arsenate reductase (ArsC), putative
*AFUA5G15000*	5	3881386	3881835	−	Arsenate reductase (ArsC), putative

*AFUA1G16100*	1	4378898	4380216	+	Arsenite permease (ArsB), putative
*AFUA5G15010*	5	3882425	3883746	+	Arsenite permease (ArsB), putative

*AFUA1G16110*	1	4380474	4381455	−	Arsenic methyltransferase (Cyt19), putative
*AFUA5G15020*	5	3883929	3884993	−	Arsenic methyltransferase (Cyt19), putative

*AFUA1G16120*	1	4385650	4386671	−	Arsenic resistance protein (ArsH), putative
*AFUA8G07150*	8	1751693	1752688	+	ArsH protein

*AFUA1G16120*	1	4385650	4386671	−	Arsenic resistance protein (ArsH), putative
*AFUA5G15030*	5	3887287	3888230	−	Arsenic resistance protein (ArsH), putative

*AFUA2G00800*	2	178081	179459	−	PelA protein
*AFUA5G10380*	5	2658656	2659968	+	Pectin lyase, putative

*AFUA2G00800*	2	178081	179459	−	PelA protein
*AFUA7G05030*	7	1182104	1183794	−	Pectin lyase B

*AFUA2G04010*	2	1092973	1094679	−	Alpha, alpha-trehalose-phosphate synthase subunit, putative
*AFUA6G12950*	6	3268958	3270783	+	Alpha, alpha-trehalose-phosphate Synthase subunitTPS1, putative

*AFUA2G11270*	2	2897275	2904954	−	Alpha-1,3-glucan synthase, putative
*AFUA3G00910*	3	210186	217666	+	Alpha-1,3-glucan synthase, putative

*AFUA3G00340*	3	71186	72734	+	Glycosyl hydrolase, putative
*AFUA4G02720*	4	751306	752715	−	Glycosyl hydrolase, putative

*AFUA3G00680*	3	151397	153549	−	Copper amine oxidase
*AFUA7G04180*	7	943893	946136	+	Amine oxidase

*AFUA3G01560*	3	393032	394802	−	Aminoacid permease, putative
*AFUA5G04260*	5	1140953	1142714	+	Arginine transporter, putative

*AFUA3G02420*	3	597476	598286	+	ThiJ/PfpI family protein
*AFUA4G01400*	4	369123	369944	−	ThiJ/PfpI family protein

*AFUA3G03080*	3	824479	825425	+	Endo-1,3(4)-beta-glucanase, putative
*AFUA6G14540*	6	3702416	3703383	−	Endo-1,3(4)-beta-glucanase, putative

*AFUA3G03980*	3	1134609	1136404	+	Cytochrome P450 monooxygenase, putative
*AFUA5G10050*	5	2589301	2591081	−	Cytochrome P450 monooxygenase, putative

*AFUA3G08160*	3	2094223	2095759	−	Eukaryotic translation initiation Factor eIF4A, putative
*AFUA5G02410*	5	621886	623486	−	DEAD/DEAH box helicase, putative

*AFUA3G14420*	3	3831868	3834921	+	Chitin synthase G
*AFUA5G00760*	5	211013	213795	+	Chitin synthase C

*AFUA4G00510*	4	133477	135102	−	Hypothetical protein
*AFUA7G08600*	7	2009436	2011052	+	Hypothetical protein

*AFUA4G03110*	4	868474	870249	−	Monosaccharide transporter
*AFUA5G10690*	5	2737348	2739178	−	Monosaccharide transporter

*AFUA4G03680*	4	1031586	1032539	−	Oxidoreductase, short-chain dehydrogenase/reductase family
*AFUA6G03520*	6	764308	765294	−	Short-chain dehydrogenase/reductase family protein, putative

*AFUA4G09440*	4	2462920	2466158	−	Sodium P-type ATPase, putative
*AFUA6G03690*	6	810027	813362	−	Sodium transport ATPase, putative

*AFUA4G14360*	4	3774307	3776166	+	Capsular associated protein, putative
*AFUA5G07560*	5	1889791	1891689	−	Capsular associated protein, putative

*AFUA5G00145*	5	15749	16327	−	Hypothetical protein
*AFUA6G11710*	6	2915903	2916483	+	Conserved hypothetical protein

*AFUA5G00145*	5	15749	16327	−	Hypothetical protein
*AFUA7G08440*	7	1942319	1942804	+	Hypothetical protein

*AFUA5G01030*	5	266294	267439	−	Glyceraldehyde 3-phosphate dehydrogenase(Ccg-7), putative
*AFUA5G01970*	5	503797	505194	+	Glyceraldehyde 3-phosphate dehydrogenase GpdA

*AFUA5G06240*	5	1494455	1495619	−	Alcohol dehydrogenase, putative
*AFUA7G01010*	7	270494	271675	−	Alcohol dehydrogenase, putative

*AFUA5G07980*	5	2019069	2020841	+	Hypothetical protein
*AFUA5G14920*	5	3857350	3859167	+	Hypothetical protein

*AFUA5G09130*	5	2345689	2346728	−	Polysaccharide deacetylase family protein
*AFUA6G05030*	6	1195846	1196956	+	Polysaccharide deacetylase family protein

*AFUA5G15030*	5	3887287	3888230	−	Arsenic resistance protein (ArsH), putative
*AFUA8G07150*	8	1751693	1752688	+	ArsH protein

*AFUA6G06750*	6	1475239	1476209	+	14-3-3 family protein
*AFUA2G03290*	2	867203	868250	−	14-3-3 family protein ArtA, putative

*AFUA6G07070*	6	1587643	1589124	+	Cellobiohydrolase D
*AFUA6G11610*	6	2878078	2879676	−	1,4-beta-D-glucan-cellobiohydrolyase, putative

*AFUA6G11430*	6	2837140	2839051	+	Aldehyde dehydrogenase, putative
*AFUA7G01000*	7	267518	269163	−	Aldehyde dehydrogenase, putative

*AFUA6G11710*	6	2915903	2916483	+	Conserved hypothetical protein
*AFUA7G08440*	7	1942319	1942804	+	Hypothetical protein

*AFUA6G13490*	6	3431912	3433612	−	Glutamate decarboxylase
*AFUA8G06020*	8	1428812	1430515	+	Glutamate decarboxylase

*AFUA7G00360*	7	102030	103064	−	UDP-galactose 4-epimerase, putative
*AFUA8G00860*	8	203496	204338	+	UDP-galactose 4-epimerase, putative

*AFUA7G07050*	7	1725686	1726402	−	Hypothetical protein
*AFUA7G08300*	7	1870244	1870960	−	Hypothetical protein

*AFUA7G07060*	7	1728876	1732992	−	Hypothetical protein
*AFUA7G08310*	7	1873437	1877817	−	Hypothetical protein

**Table 2:** Spearman's rank correlation coefficients of nucleosome position profiles in the promoter and body regions of 63 duplicated gene pairs in *Aspergillus fumigatus*.

Gene pair		Gene promoter	Gene body
*AFUA1G00150*	*AFUA6G09370*	0.750176913	0.215204254
*AFUA1G00420*	*AFUA8G04120*	0.80370974	0.723149072
*AFUA1G00440*	*AFUA8G04110*	0.856048561	0.225149091
*AFUA1G00450*	*AFUA8G04100*	0.9213602	0.94633912
*AFUA1G00470*	*AFUA8G04080*	0.433118045	0.818800395
*AFUA1G00530*	*AFUA4G12050*	0.326735006	−0.199291887
*AFUA1G00550*	*AFUA1G00910*	0.676910334	0.678763937
*AFUA1G00580*	*AFUA8G04050*	0.305291793	0.312927993
*AFUA1G00650*	*AFUA7G08510*	0.448171525	0.085599209
*AFUA1G00920*	*AFUA3G06425*	0.719748778	−0.340001527
*AFUA1G01050*	*AFUA8G06160*	0.635149308	0.176679577
*AFUA1G02550*	*AFUA2G14990*	0.139079116	−0.036644732
*AFUA1G02730*	*AFUA1G15140*	0.452193259	0.067186644
*AFUA1G05760*	*AFUA5G15010*	0.395849711	0.117006666
*AFUA1G05760*	*AFUA1G16100*	0.127911436	−0.397363108
*AFUA1G10910*	*AFUA7G00250*	0.310718577	0.665906466
*AFUA1G11260*	*AFUA6G00270*	0.617303556	−0.02397179
*AFUA1G11610*	*AFUA3G14850*	0.636281069	0.869300724
*AFUA1G11890*	*AFUA6G00300*	0.254487564	0.429087849
*AFUA1G12850*	*AFUA1G17470*	0.644447287	0.008468365
*AFUA1G15970*	*AFUA8G01560*	0.373850536	−0.106800689
*AFUA1G16030*	*AFUA5G14930*	0.618349765	0.858488283
*AFUA1G16040*	*AFUA5G14940*	0.481054005	0.017330056
*AFUA1G16050*	*AFUA5G14950*	−0.345037268	−0.392132138
*AFUA1G16070*	*AFUA5G14980*	0.930922124	0.902581516
*AFUA1G16080*	*AFUA5G14990*	0.559980787	0.136819379
*AFUA1G16090*	*AFUA5G15000*	0.773867924	0.338119153
*AFUA1G16100*	*AFUA5G15010*	0.681170098	0.70434823
*AFUA1G16110*	*AFUA5G15020*	−0.261864905	0.632982505
*AFUA1G16120*	*AFUA8G07150*	0.356869915	0.074502754
*AFUA1G16120*	*AFUA5G15030*	−0.135442129	−0.239074416
*AFUA2G00800*	*AFUA5G10380*	0.352063916	0.460712971
*AFUA2G00800*	*AFUA7G05030*	−0.114184443	0.457081245
*AFUA2G04010*	*AFUA6G12950*	0.134790545	−0.14813806
*AFUA2G11270*	*AFUA3G00910*	−0.004207858	0.192024608
*AFUA3G00340*	*AFUA4G02720*	−0.050259987	0.055757479
*AFUA3G00680*	*AFUA7G04180*	0.399912713	−0.044615588
*AFUA3G01560*	*AFUA5G04260*	0.179395067	0.125177632
*AFUA3G02420*	*AFUA4G01400*	0.662712481	0.646400554
*AFUA3G03080*	*AFUA6G14540*	0.3401707	−0.021961056
*AFUA3G03980*	*AFUA5G10050*	0.486726534	0.318475376
*AFUA3G08160*	*AFUA5G02410*	0.309645464	−0.255654021
*AFUA3G14420*	*AFUA5G00760*	0.917685134	0.415111431
*AFUA4G00510*	*AFUA7G08600*	0.578582743	0.441363777
*AFUA4G03110*	*AFUA5G10690*	0.721540193	0.176996881
*AFUA4G03680*	*AFUA6G03520*	0.662657833	−0.320417166
*AFUA4G09440*	*AFUA6G03690*	0.204159184	0.000609208
*AFUA4G14360*	*AFUA5G07560*	0.038083073	0.515103795
*AFUA5G00145*	*AFUA6G11710*	0.809402393	−0.741045496
*AFUA5G00145*	*AFUA7G08440*	0.019652845	−0.575600966
*AFUA5G01030*	*AFUA5G01970*	0.576211178	0.132479521
*AFUA5G06240*	*AFUA7G01010*	0.163674862	−0.552899419
*AFUA5G07980*	*AFUA5G14920*	0.669562689	−0.265207346
*AFUA5G09130*	*AFUA6G05030*	0.092452938	0.729278817
*AFUA5G15030*	*AFUA8G07150*	0.337482368	−0.502391454
*AFUA6G06750*	*AFUA2G03290*	0.333402926	−0.538389013
*AFUA6G07070*	*AFUA6G11610*	−0.048961745	−0.239430043
*AFUA6G11430*	*AFUA7G01000*	0.0145492	0.476692417
*AFUA6G11710*	*AFUA7G08440*	0.243362623	0.823124582
*AFUA6G13490*	*AFUA8G06020*	0.768186533	0.174847009
*AFUA7G00360*	*AFUA8G00860*	−0.115050268	0.03815086
*AFUA7G07050*	*AFUA7G08300*	0.70756842	0.778653567
*AFUA7G07060*	*AFUA7G08310*	0.906852725	0.919839072

**Table 3:** Genes of* Saccharomyces cerevisiae* with highly conserved nucleosome positions in the promoters of the control and histone modification gene deletion mutants.

Chromosome	Gene	Correlation coefficient between the control and the *ELP3* deletion	Correlation coefficient between the control and the *HOS2* deletion	Translational start site	Transcription direction
chr01	*YAL064W-B*	0.974719955	0.965814243	12047	+
chr01	*YAL056W*	0.989801003	0.982157328	39260	+
chr01	*YAL047C*	0.950243691	0.953129232	56858	−
chr01	*YAR019C*	0.970115162	0.960628643	175133	−
chr01	*YAR033W*	0.968807875	0.987221166	188101	+
chr02	*YBL111C*	0.995802065	0.99455065	5009	−
chr02	*YBL108C-A*	0.959497013	0.969966542	7733	−
chr02	*YBL101C*	0.958517125	0.991324096	28299	−
chr02	*YBL087C*	0.95719187	0.957273203	60735	−
chr02	*YBL061C*	0.984960212	0.992728731	107408	−
chr02	*YBL060W*	0.960330289	0.99357258	107934	+
chr02	*YBL051C*	0.97653734	0.989165943	124762	−
chr02	*YBL032W*	0.956874854	0.980366627	160187	+
chr02	*YBL005W-B*	0.950781322	0.952679326	221333	+
chr02	*YBL005W-A*	0.950781322	0.952679326	221333	+
chr02	*YBR023C*	0.960238499	0.992906362	287925	−
chr02	*YBR029C*	0.959231389	0.987222377	297742	−
chr02	*YBR047W*	0.964564522	0.963289004	331831	+
chr02	*YBR060C*	0.959229871	0.974718825	362512	−
chr02	*YBR084W*	0.962172866	0.981530879	411048	+
chr02	*YBR090C*	0.967658785	0.971792497	427052	−
chr02	*YBR091C*	0.965037862	0.985960321	427478	−
chr02	*YBR131W*	0.953323249	0.969685452	497157	+
chr02	*YBR136W*	0.963546822	0.974854426	505662	+
chr02	*YBR173C*	0.978518391	0.961438459	582167	−
chr02	*YBR179C*	0.988489146	0.991754907	589109	−
chr02	*YBR180W*	0.987132212	0.975974209	589736	+
chr02	*YBR204C*	0.970958946	0.978433534	633376	−
chr02	*YBR243C*	0.972253467	0.961252065	706788	−
chr02	*YBR244W*	0.974202306	0.992702483	707523	+
chr02	*YBR249C*	0.955742682	0.976101912	717989	−
chr02	*YBR250W*	0.954149188	0.976352348	719028	+
chr02	*YBR251W*	0.96273261	0.961120273	721385	+
chr02	*YBR258C*	0.966601899	0.955155711	730157	−
chr02	*YBR260C*	0.969346019	0.970918059	734634	−
chr02	*YBR279W*	0.956081387	0.964626558	761253	+
chr02	*YBR290W*	0.981586823	0.967935658	782587	+
chr03	*YCL064C*	0.957150698	0.95270514	16880	−
chr03	*YCL058W-A*	0.969895345	0.992394829	23584	+
chr03	*YCL057C-A*	0.990886192	0.987170576	24325	−
chr03	*YCL057W*	0.963062357	0.961804623	24768	+
chr03	*YCL038C*	0.978210537	0.957633548	56527	−
chr03	*YCL035C*	0.981360705	0.973568151	61173	−
chr03	*YCL019W*	0.953241664	0.978688459	85102	+
chr03	*YCR026C*	0.96886665	0.97932414	166335	−
chr03	*YCR043C*	0.951764864	0.966166673	206640	−
chr03	*YCR053W*	0.989728241	0.992623598	216693	+
chr03	*YCR087C-A*	0.975398919	0.993232435	264464	−
chr03	*YCR090C*	0.965637294	0.95018878	272860	−
chr03	*YCR108C*	0.985803086	0.993860549	316185	−
chr04	*YDL248W*	0.964701365	0.980217326	1802	+
chr04	*YDL247W*	0.989686199	0.997997741	5985	+
chr04	*YDL233W*	0.957672252	0.97857489	36798	+
chr04	*YDL232W*	0.979592686	0.970413477	38488	+
chr04	*YDL225W*	0.950053133	0.978441348	52446	+
chr04	*YDL208W*	0.987264893	0.987894794	87513	+
chr04	*YDL189W*	0.965815356	0.98561241	122217	+
chr04	*YDL174C*	0.956215571	0.953787348	147590	−
chr04	*YDL147W*	0.971755059	0.950911485	190925	+
chr04	*YDL116W*	0.965936123	0.976379579	251566	+
chr04	*YDL110C*	0.974238254	0.97295169	264964	−
chr04	*YDL102W*	0.957786538	0.980122821	276872	+
chr04	*YDL085W*	0.97887937	0.960150217	303211	+
chr04	*YDL035C*	0.975573212	0.977145228	392054	−
chr04	*YDL025C*	0.96134673	0.972526893	407203	−
chr04	*YDR019C*	0.95269772	0.953635877	485362	−
chr04	*YDR028C*	0.960937436	0.959981321	500876	−
chr04	*YDR034C-C*	0.967222636	0.9814374	519353	−
chr04	*YDR034C-D*	0.967222636	0.981437429	519353	−
chr04	*YDR037W*	0.951365292	0.956861294	525437	+
chr04	*YDR054C*	0.955928802	0.982048103	562325	−
chr04	*YDR055W*	0.950321012	0.952540035	563525	+
chr04	*YDR062W*	0.974021833	0.967688258	576471	+
chr04	*YDR109C*	0.954396163	0.952291074	675664	−
chr04	*YDR110W*	0.986081905	0.974555387	676099	+
chr04	*YDR120C*	0.960200845	0.969981962	693258	−
chr04	*YDR162C*	0.967301741	0.984347121	781097	−
chr04	*YDR233C*	0.967024058	0.967951444	930353	−
chr04	*YDR234W*	0.967415159	0.980945022	931125	+
chr04	*YDR238C*	0.97602642	0.973622232	940812	−
chr04	*YDR261C-D*	0.984848646	0.993722663	992345	−
chr04	*YDR261C-C*	0.984848646	0.9937227	992345	−
chr04	*YDR262W*	0.990989397	0.995915841	993130	+
chr04	*YDR270W*	0.951139826	0.96263534	1005671	+
chr04	*YDR281C*	0.984619329	0.959392909	1022317	−
chr04	*YDR300C*	0.975849644	0.971677938	1062787	−
chr04	*YDR301W*	0.952923341	0.977515222	1063348	+
chr04	*YDR307W*	0.951918886	0.970033626	1075861	+
chr04	*YDR310C*	0.951723931	0.969273153	1084312	−
chr04	*YDR311W*	0.953685357	0.980595738	1085062	+
chr04	*YDR317W*	0.98969221	0.991598049	1102181	+
chr04	*YDR322W*	0.954843272	0.97204312	1110586	+
chr04	*YDR328C*	0.957421993	0.983957835	1126013	−
chr04	*YDR334W*	0.953030288	0.963155512	1135927	+
chr04	*YDR359C*	0.977489172	0.969606029	1194877	−
chr04	*YDR365C*	0.985073544	0.998926611	1206375	−
chr04	*YDR367W*	0.990041805	0.994689201	1212840	+
chr04	*YDR369C*	0.957655919	0.96987305	1217572	−
chr04	*YDR379W*	0.962899057	0.974939821	1230159	+
chr04	*YDR397C*	0.966298918	0.975133337	1266890	−
chr04	*YDR420W*	0.971217177	0.991886941	1306259	+
chr04	*YDR424C*	0.966881808	0.989813233	1319833	−
chr04	*YDR432W*	0.955728738	0.982171718	1328775	+
chr04	*YDR438W*	0.971626925	0.98904082	1338266	+
chr04	*YDR444W*	0.973566433	0.976502447	1350282	+
chr04	*YDR453C*	0.952661492	0.954481014	1365654	−
chr04	*YDR476C*	0.969940159	0.964679279	1411119	−
chr04	*YDR477W*	0.954743343	0.979243738	1412365	+
chr04	*YDR479C*	0.977460825	0.994501786	1416866	−
chr04	*YDR480W*	0.967727074	0.986953403	1417391	+
chr04	*YDR488C*	0.983114512	0.979187155	1430781	−
chr04	*YDR497C*	0.955855412	0.958236577	1445459	−
chr04	*YDR529C*	0.966273576	0.971812076	1496540	−
chr05	*YEL072W*	0.968131913	0.977435534	13720	+
chr05	*YEL043W*	0.979079858	0.96227706	70478	+
chr05	*YEL038W*	0.981572221	0.988369151	80462	+
chr05	*YEL021W*	0.951315475	0.951565922	116167	+
chr05	*YER004W*	0.956348638	0.987671859	159579	+
chr05	*YER026C*	0.964952897	0.993875293	208473	−
chr05	*YER076C*	0.965398244	0.971982059	313494	−
chr05	*YER083C*	0.953185048	0.981521771	327027	−
chr05	*YER094C*	0.976027049	0.982595427	349342	−
chr05	*YER095W*	0.963423619	0.988752501	349976	+
chr05	*YER107C*	0.982024941	0.98488468	374541	−
chr05	*YER109C*	0.969371754	0.986034001	377610	−
chr05	*YER173W*	0.960496827	0.952930419	536295	+
chr05	*YER188C-A*	0.991489293	0.996538184	569902	−
chr05	*YER189W*	0.997057108	0.998333831	571150	+
chr06	*YFL066C*	0.970012372	0.993098415	2615	−
chr06	*YFL065C*	0.957034943	0.97004682	3338	−
chr06	*YFL060C*	0.987757312	0.995138274	10969	−
chr06	*YFL059W*	0.976205235	0.962195117	11363	+
chr06	*YFL058W*	0.9750941	0.994474768	12929	+
chr06	*YFL028C*	0.966244522	0.959308346	80211	−
chr06	*YFL026W*	0.957755207	0.952711873	82578	+
chr06	*YFR009W*	0.979200258	0.985708335	162482	+
chr06	*YFR013W*	0.972958971	0.986214291	169914	+
chr06	*YFR037C*	0.965415915	0.972070667	229173	−
chr07	*YGL255W*	0.961366208	0.972062316	20978	+
chr07	*YGL248W*	0.971369747	0.971756841	35653	+
chr07	*YGL223C*	0.956215218	0.984994924	80364	−
chr07	*YGL215W*	0.965861339	0.95181154	87980	+
chr07	*YGL201C*	0.954296336	0.979120204	120911	−
chr07	*YGL180W*	0.982084299	0.994334205	160071	+
chr07	*YGL171W*	0.969321982	0.974163208	182396	+
chr07	*YGL163C*	0.975825825	0.968348392	196409	−
chr07	*YGL138C*	0.971247145	0.983410766	249536	−
chr07	*YGL120C*	0.981452639	0.964320425	283943	−
chr07	*YGL119W*	0.986610225	0.981525372	284448	+
chr07	*YGL108C*	0.961840326	0.985939242	304074	−
chr07	*YGL058W*	0.977732494	0.975176081	393992	+
chr07	*YGL056C*	0.960426378	0.969119302	397624	−
chr07	*YGL055W*	0.964044825	0.969447792	398631	+
chr07	*YGL048C*	0.971402915	0.953304338	411289	−
chr07	*YGL043W*	0.964287661	0.976335397	417487	+
chr07	*YGL028C*	0.960077919	0.965512687	442914	−
chr07	*YGL006W*	0.950151816	0.968357705	485925	+
chr07	*YGR001C*	0.958621356	0.990717019	498038	−
chr07	*YGR006W*	0.980877592	0.97721459	506074	+
chr07	*YGR027W-B*	0.973585999	0.969942324	536061	+
chr07	*YGR027W-A*	0.973585999	0.969942324	536061	+
chr07	*YGR054W*	0.964434273	0.980689617	596697	+
chr07	*YGR076C*	0.981588154	0.958402031	637581	−
chr07	*YGR082W*	0.986433799	0.977608093	644048	+
chr07	*YGR084C*	0.952879595	0.959716787	648146	−
chr07	*YGR109C*	0.967037071	0.989224447	706505	−
chr07	*YGR109W-B*	0.964433838	0.990506548	707614	+
chr07	*YGR109W-A*	0.964433838	0.990506548	707614	+
chr07	*YGR149W*	0.957244122	0.960107682	789036	+
chr07	*YGR161W-B*	0.958455297	0.972873884	811743	+
chr07	*YGR161W-A*	0.958455297	0.9728739	811743	+
chr07	*YGR161C-D*	0.9711882	0.974318881	823020	−
chr07	*YGR161C-C*	0.9711882	0.9743189	823020	−
chr07	*YGR162W*	0.967152562	0.972476532	824064	+
chr07	*YGR165W*	0.959888658	0.951709091	829121	+
chr07	*YGR166W*	0.978481923	0.950610672	830520	+
chr07	*YGR173W*	0.978946749	0.974220242	843859	+
chr07	*YGR178C*	0.97338186	0.951056442	853220	−
chr07	*YGR193C*	0.977703185	0.9816188	885746	−
chr07	*YGR198W*	0.954889766	0.972754857	894698	+
chr07	*YGR239C*	0.968888325	0.989258725	970058	−
chr07	*YGR240C*	0.966313371	0.971843468	973739	−
chr07	*YGR255C*	0.966583058	0.991666026	1003967	−
chr07	*YGR267C*	0.960278778	0.969707488	1025741	−
chr07	*YGR280C*	0.961084505	0.952670993	1051732	−
chr07	*YGR295C*	0.967286944	0.964457386	1082736	−
chr07	*YGR296W*	0.971130914	0.991185295	1084871	+
chr08	*YHL044W*	0.958725508	0.965538017	13563	+
chr08	*YHL029C*	0.988947037	0.966760586	47966	−
chr08	*YHL028W*	0.983034685	0.977043411	48761	+
chr08	*YHL024W*	0.961645582	0.966633627	56647	+
chr08	*YHL020C*	0.978013871	0.986901259	67453	−
chr08	*YHL016C*	0.976082816	0.988408432	74241	−
chr08	*YHL007C*	0.973847748	0.981293306	97933	−
chr08	*YHL004W*	0.97418766	0.993677693	99215	+
chr08	*YHL001W*	0.959021584	0.965334128	104272	+
chr08	*YHR001W-A*	0.991644256	0.988918444	107821	+
chr08	*YHR056C*	0.972836967	0.982728846	217836	−
chr08	*YHR081W*	0.961720079	0.960688905	267540	+
chr08	*YHR091C*	0.955695121	0.987437891	286772	−
chr08	*YHR101C*	0.984820398	0.996893941	315971	−
chr08	*YHR102W*	0.96127201	0.995534701	316575	+
chr08	*YHR107C*	0.950250496	0.982165489	328039	−
chr08	*YHR118C*	0.973725305	0.961118169	345631	−
chr08	*YHR127W*	0.95014104	0.981438554	360916	+
chr08	*YHR136C*	0.953476026	0.965665058	375103	−
chr08	*YHR148W*	0.978585198	0.950179497	393537	+
chr08	*YHR153C*	0.959414518	0.982780385	402685	−
chr08	*YHR165C*	0.958747068	0.984253541	436950	−
chr08	*YHR214C-D*	0.987951207	0.966688891	550941	−
chr08	*YHR215W*	0.987951207	0.966688891	552099	+
chr08	*YHR216W*	0.972948554	0.975124764	554396	+
chr09	*YIL158W*	0.961579117	0.957386446	46201	+
chr09	*YIL154C*	0.954401073	0.971892535	55021	−
chr09	*YIL137C*	0.976010283	0.976810775	92788	−
chr09	*YIL135C*	0.976089791	0.979613921	96375	−
chr09	*YIL134W*	0.974541094	0.979148771	97395	+
chr09	*YIL129C*	0.958227819	0.99048832	113237	−
chr09	*YIL125W*	0.963070211	0.960333013	122689	+
chr09	*YIL063C*	0.970956253	0.957414461	243741	−
chr09	*YIL061C*	0.95394965	0.989156182	245556	−
chr09	*YIL046W*	0.967209958	0.952996831	268650	+
chr09	*YIL033C*	0.989017477	0.989306501	291668	−
chr09	*YIL031W*	0.985799922	0.98683422	292632	+
chr09	*YIL030C*	0.957449018	0.97109633	300008	−
chr09	*YIR022W*	0.960636707	0.971998311	398730	+
chr09	*YIR024C*	0.961569219	0.970038905	403488	−
chr09	*YIR038C*	0.973108358	0.975735046	424510	−
chr10	*YJL221C*	0.992889333	0.995409763	18536	−
chr10	*YJL219W*	0.992889333	0.995409763	19497	+
chr10	*YJL197W*	0.963017116	0.967339021	63804	+
chr10	*YJL181W*	0.971567753	0.971225117	85658	+
chr10	*YJL176C*	0.979890865	0.967535459	94528	−
chr10	*YJL174W*	0.983474082	0.9822043	95090	+
chr10	*YJL173C*	0.986867668	0.989398406	96527	−
chr10	*YJL151C*	0.952976229	0.987631587	136770	−
chr10	*YJL149W*	0.975756648	0.990848833	137376	+
chr10	*YJL118W*	0.953268112	0.959468203	191638	+
chr10	*YJL113W*	0.982940363	0.963188993	197913	+
chr10	*YJL114W*	0.982940363	0.963188993	197913	+
chr10	*YJL093C*	0.967675882	0.964352732	256807	−
chr10	*YJL092W*	0.963240719	0.958166876	257418	+
chr10	*YJL066C*	0.953982678	0.974252054	314867	−
chr10	*YJL050W*	0.950677364	0.95235067	342517	+
chr10	*YJL048C*	0.96132641	0.958967756	348632	−
chr10	*YJL039C*	0.950918953	0.970557445	373794	−
chr10	*YJL034W*	0.963347804	0.980266739	381322	+
chr10	*YJR010W*	0.971667854	0.97428271	456232	+
chr10	*YJR021C*	0.956762721	0.994264722	469572	−
chr10	*YJR029W*	0.958070547	0.974229894	478337	+
chr10	*YJR028W*	0.958070547	0.9742299	478337	+
chr10	*YJR041C*	0.976618081	0.981063923	513756	−
chr10	*YJR048W*	0.960606245	0.975055837	526328	+
chr10	*YJR049C*	0.954983464	0.986149168	528469	−
chr10	*YJR055W*	0.976932113	0.992763445	538765	+
chr10	*YJR065C*	0.975569729	0.987649101	559151	−
chr10	*YJR095W*	0.97047495	0.971814385	609769	+
chr10	*YJR113C*	0.969228269	0.954716018	638969	−
chr10	*YJR115W*	0.952485858	0.960980837	639936	+
chr10	*YJR141W*	0.957349189	0.956946497	695900	+
chr10	*YJR160C*	0.978268521	0.997490962	739810	−
chr10	*YJR161C*	0.965251269	0.976575544	743993	−
chr11	*YKL191W*	0.950933251	0.978993764	81040	+
chr11	*YKL179C*	0.975707433	0.967918905	112508	−
chr11	*YKL167C*	0.956085616	0.955561088	134139	−
chr11	*YKL165C*	0.960609423	0.966621061	140696	−
chr11	*YKL157W*	0.95635277	0.9813363	154996	+
chr11	*YKL127W*	0.955985661	0.982208081	203185	+
chr11	*YKL125W*	0.976449752	0.960074911	207891	+
chr11	*YKL113C*	0.971544612	0.954413703	225519	−
chr11	*YKL065C*	0.96005541	0.969818928	316701	−
chr11	*YKL064W*	0.974644257	0.972632809	317408	+
chr11	*YKL059C*	0.957153957	0.961241915	329087	−
chr11	*YKL020C*	0.955860253	0.977812511	401723	−
chr11	*YKL013C*	0.972958978	0.953444609	417666	−
chr11	*YKR007W*	0.981387104	0.972277081	451077	+
chr11	*YKR024C*	0.971944038	0.981215773	487015	−
chr11	*YKR031C*	0.952123552	0.969614994	506037	−
chr11	*YKR036C*	0.959340192	0.988104612	510275	−
chr11	*YKR041W*	0.982786597	0.957524998	517840	+
chr11	*YKR052C*	0.954392373	0.96688713	533106	−
chr11	*YKR082W*	0.962931616	0.991018501	592467	+
chr11	*YKR084C*	0.953228662	0.976903834	598532	−
chr11	*YKR086W*	0.952006608	0.976533657	599499	+
chr12	*YLL050C*	0.976179673	0.990695339	40413	−
chr12	*YLL002W*	0.972688874	0.981122544	146290	+
chr12	*YLR001C*	0.966206724	0.98012374	153976	−
chr12	*YLR012C*	0.956516633	0.962463803	170280	−
chr12	*YLR013W*	0.978873849	0.985557204	171338	+
chr12	*YLR024C*	0.953442868	0.956160837	193282	−
chr12	*YLR025W*	0.971468039	0.96202948	194453	+
chr12	*YLR029C*	0.961637928	0.991619508	202591	−
chr12	*YLR059C*	0.963450626	0.974770886	260548	−
chr12	*YLR085C*	0.9560163	0.984640913	301990	−
chr12	*YLR087C*	0.953704478	0.990085627	315732	−
chr12	*YLR096W*	0.977966477	0.979318401	332591	+
chr12	*YLR104W*	0.966174553	0.97921157	346586	+
chr12	*YLR133W*	0.961307333	0.951823465	408446	+
chr12	*YLR135W*	0.977560494	0.977991425	413282	+
chr12	*YLR137W*	0.967174193	0.983596278	417007	+
chr12	*YLR162W-A*	0.968123219	0.962766706	490407	+
chr12	*YLR208W*	0.96802684	0.969296393	559553	+
chr12	*YLR223C*	0.966783974	0.974989936	585492	−
chr12	*YLR224W*	0.974582795	0.978003757	586466	+
chr12	*YLR286C*	0.972231318	0.978358686	710138	−
chr12	*YLR299W*	0.962625418	0.970226227	726071	+
chr12	*YLR307W*	0.962710223	0.990591743	745622	+
chr12	*YLR323C*	0.953205594	0.975247431	778952	−
chr12	*YLR326W*	0.979769576	0.991583861	782174	+
chr12	*YLR355C*	0.967858474	0.973834311	839252	−
chr12	*YLR356W*	0.968277882	0.974767083	840320	+
chr12	*YLR378C*	0.974122358	0.977246394	877177	−
chr12	*YLR380W*	0.973258508	0.97821865	878282	+
chr12	*YLR410W-A*	0.951545011	0.9529714	941481	+
chr12	*YLR410W-B*	0.951545011	0.952971407	941481	+
chr12	*YLR426W*	0.9668981	0.990317101	987059	+
chr12	*YLR427W*	0.968631588	0.972538491	988425	+
chr12	*YLR429W*	0.950464544	0.951528134	990774	+
chr12	*YLR443W*	0.964525656	0.991440098	1022622	+
chr13	*YML121W*	0.974659011	0.985969584	26930	+
chr13	*YML115C*	0.97337994	0.996063492	41794	−
chr13	*YML080W*	0.989302036	0.986381735	108806	+
chr13	*YML078W*	0.975873108	0.974136336	111002	+
chr13	*YML045W*	0.993016416	0.993250991	184461	+
chr13	*YML045W-A*	0.993016416	0.993251	184461	+
chr13	*YML041C*	0.969801821	0.979224272	195755	−
chr13	*YML020W*	0.975260683	0.95439232	231149	+
chr13	*YML004C*	0.966616377	0.98388072	262685	−
chr13	*YML003W*	0.968338912	0.981828638	263483	+
chr13	*YMR010W*	0.985774326	0.979191015	285099	+
chr13	*YMR011W*	0.963732981	0.981283867	288078	+
chr13	*YMR027W*	0.961041993	0.957831173	325876	+
chr13	*YMR036C*	0.969260192	0.963784402	343519	−
chr13	*YMR060C*	0.990018805	0.990517987	392514	−
chr13	*YMR066W*	0.98321165	0.95995151	401540	+
chr13	*YMR078C*	0.969392941	0.976546196	424727	−
chr13	*YMR081C*	0.981254312	0.956294421	431094	−
chr13	*YMR110C*	0.961424621	0.983151943	491991	−
chr13	*YMR116C*	0.977129849	0.989254306	500687	−
chr13	*YMR137C*	0.971971845	0.978526214	544962	−
chr13	*YMR138W*	0.978634859	0.963362392	545154	+
chr13	*YMR152W*	0.958913422	0.961601086	563095	+
chr13	*YMR197C*	0.960464224	0.981756931	659197	−
chr13	*YMR210W*	0.974579381	0.983178959	687515	+
chr13	*YMR214W*	0.977906077	0.988639434	695349	+
chr13	*YMR219W*	0.964555974	0.962486321	707132	+
chr13	*YMR224C*	0.975063127	0.950620617	720652	−
chr13	*YMR229C*	0.960514554	0.957025636	731122	−
chr13	*YMR241W*	0.956225901	0.967912184	751960	+
chr13	*YMR319C*	0.950340769	0.968043162	914536	−
chr14	*YNL339C*	0.975221324	0.997373072	6098	−
chr14	*YNL334C*	0.968865734	0.991228175	12876	−
chr14	*YNL322C*	0.954706246	0.977799203	34234	−
chr14	*YNL311C*	0.986814003	0.993782307	51687	−
chr14	*YNL310C*	0.956852702	0.980516471	52430	−
chr14	*YNL309W*	0.988062215	0.993825499	52661	+
chr14	*YNL301C*	0.968107997	0.966680876	64562	−
chr14	*YNL295W*	0.952849161	0.964836983	76946	+
chr14	*YNL261W*	0.950477134	0.976226949	155101	+
chr14	*YNL260C*	0.968893453	0.965562883	157456	−
chr14	*YNL255C*	0.959418743	0.961868386	167791	−
chr14	*YNL248C*	0.959305496	0.962132738	182609	−
chr14	*YNL241C*	0.952591706	0.968306617	197944	−
chr14	*YNL234W*	0.975005906	0.964281927	210234	+
chr14	*YNL224C*	0.973438019	0.985601741	227100	−
chr14	*YNL212W*	0.967303893	0.964441355	247462	+
chr14	*YNL166C*	0.973079673	0.979903307	323567	−
chr14	*YNL156C*	0.961076874	0.96913283	341970	−
chr14	*YNL112W*	0.953769466	0.987266001	413641	+
chr14	*YNL099C*	0.959678116	0.95727388	439285	−
chr14	*YNL097C*	0.978605808	0.986440434	442360	−
chr14	*YNL082W*	0.955790378	0.980457797	473392	+
chr14	*YNL055C*	0.982685169	0.981068004	518846	−
chr14	*YNL042W-B*	0.956836355	0.979900708	547114	+
chr14	*YNL029C*	0.976101029	0.988675718	578774	−
chr14	*YNL027W*	0.96462873	0.978413199	579581	+
chr14	*YNR012W*	0.958400399	0.977490746	647434	+
chr14	*YNR015W*	0.967843094	0.979265652	653389	+
chr14	*YNR023W*	0.975180387	0.972628935	670420	+
chr14	*YNR026C*	0.966267575	0.984095961	674691	−
chr14	*YNR036C*	0.955455936	0.956914254	694824	−
chr14	*YNR039C*	0.97056523	0.9708253	699433	−
chr14	*YNR075W*	0.950188074	0.953766447	779916	+
chr14	*YNR075C-A*	0.986389631	0.991000914	781603	−
chr15	*YOL166W-A*	0.969218719	0.964760148	585	+
chr15	*YOL157C*	0.977029388	0.98945557	24293	−
chr15	*YOL156W*	0.979765672	0.992685071	25272	+
chr15	*YOL148C*	0.957761501	0.971157209	47573	−
chr15	*YOL104C*	0.976917372	0.981386975	117454	−
chr15	*YOL100W*	0.961328138	0.959394148	129237	+
chr15	*YOL089C*	0.971692903	0.984860678	153490	−
chr15	*YOL086C*	0.976082094	0.966862518	160594	−
chr15	*YOL077C*	0.956963602	0.983941333	186723	−
chr15	*YOL068C*	0.959180595	0.953847182	201879	−
chr15	*YOL062C*	0.954102242	0.967526899	211995	−
chr15	*YOL058W*	0.969401099	0.978364009	219210	+
chr15	*YOL045W*	0.966108727	0.955785397	243496	+
chr15	*YOL031C*	0.971563515	0.959211572	267530	−
chr15	*YOL026C*	0.954115513	0.976281121	274354	−
chr15	*YOL023W*	0.960879668	0.960550998	278057	+
chr15	*YOL006C*	0.971429281	0.978082289	315388	−
chr15	*YOR043W*	0.970409112	0.977199399	410870	+
chr15	*YOR048C*	0.95240535	0.972598244	421651	−
chr15	*YOR056C*	0.95552512	0.980531823	431628	−
chr15	*YOR058C*	0.977288174	0.978577416	436347	−
chr15	*YOR071C*	0.980341941	0.989920087	461278	−
chr15	*YOR075W*	0.983591979	0.981471486	468214	+
chr15	*YOR089C*	0.978934603	0.984066065	490830	−
chr15	*YOR104W*	0.965107091	0.973591736	517643	+
chr15	*YOR124C*	0.97179062	0.983946384	558643	−
chr15	*YOR129C*	0.979558976	0.983142651	569559	−
chr15	*YOR132W*	0.955282168	0.965555781	573176	+
chr15	*YOR148C*	0.987044445	0.98569999	609198	−
chr15	*YOR192C-C*	0.980704362	0.966062169	704225	−
chr15	*YOR193W*	0.973179583	0.992657727	710447	+
chr15	*YOR204W*	0.985619071	0.960639074	722912	+
chr15	*YOR216C*	0.971045967	0.970515948	748980	−
chr15	*YOR247W*	0.959336754	0.957210052	797677	+
chr15	*YOR294W*	0.972999786	0.97236413	868339	+
chr15	*YOR336W*	0.973501735	0.969442343	949770	+
chr15	*YOR365C*	0.981766719	0.98630012	1025570	−
chr15	*YOR372C*	0.955936893	0.971693401	1036469	−
chr15	*YOR389W*	0.964833697	0.976852438	1074211	+
chr15	*YOR390W*	0.98673199	0.997343789	1076782	+
chr15	*YOR391C*	0.952090431	0.990889663	1079256	−
chr15	*YOR393W*	0.952090431	0.990889663	1080274	+
chr16	*YPL283C*	0.990485958	0.9953985	6007	−
chr16	*YPL281C*	0.961921468	0.977623026	10870	−
chr16	*YPL280W*	0.961921468	0.977623026	11887	+
chr16	*YPL278C*	0.965633274	0.959753752	15355	−
chr16	*YPL257W-B*	0.976897418	0.976806903	56748	+
chr16	*YPL257W-A*	0.976897418	0.976807	56748	+
chr16	*YPL255W*	0.96664841	0.970266728	67725	+
chr16	*YPL254W*	0.95123182	0.962107221	69485	+
chr16	*YPL206C*	0.975807996	0.972912432	163596	−
chr16	*YPL196W*	0.978338347	0.984467392	175042	+
chr16	*YPL180W*	0.976152618	0.987674239	205247	+
chr16	*YPL171C*	0.96524809	0.961521166	227370	−
chr16	*YPL158C*	0.980225654	0.985491741	254309	−
chr16	*YPL157W*	0.98638216	0.988124386	254813	+
chr16	*YPL146C*	0.98509812	0.956540524	277528	−
chr16	*YPL139C*	0.981115598	0.987215069	291050	−
chr16	*YPL126W*	0.988937478	0.982334739	310209	+
chr16	*YPL082C*	0.980458525	0.992300343	404080	−
chr16	*YPL078C*	0.957476311	0.95576197	408741	−
chr16	*YPL038W*	0.953416137	0.966495031	480532	+
chr16	*YPL030W*	0.973101156	0.975002429	493541	+
chr16	*YPL007C*	0.954338314	0.983931954	543845	−
chr16	*YPR010C*	0.983770387	0.983449754	581193	−
chr16	*YPR017C*	0.986325514	0.989002388	593914	−
chr16	*YPR018W*	0.981145288	0.96510122	594473	+
chr16	*YPR020W*	0.950323294	0.983815562	599867	+
chr16	*YPR048W*	0.980910651	0.976100682	659179	+
chr16	*YPR060C*	0.960902075	0.969265445	675628	−
chr16	*YPR062W*	0.96911875	0.967204709	677162	+
chr16	*YPR088C*	0.957617837	0.976321629	713026	−
chr16	*YPR141C*	0.978516693	0.973377917	817919	−
chr16	*YPR145C-A*	0.961127516	0.965220022	824922	−
chr16	*YPR156C*	0.966866802	0.956230015	839773	−
chr16	*YPR158C-D*	0.981640164	0.971311933	856253	−
chr16	*YPR158C-C*	0.981640164	0.971311933	856253	−
chr16	*YPR161C*	0.969622155	0.984283791	866418	−
chr16	*YPR165W*	0.959239007	0.967322323	875364	+
chr16	*YPR176C*	0.986542093	0.977514778	892074	−
chr16	*YPR187W*	0.964480751	0.98804279	911253	+
chr16	*YPR203W*	0.968955474	0.985644074	943876	+

**Table 4:** Spearman's rank correlation coefficients between nucleosome position profiles in the promoters of 347 orthologous gene pairs between *Aspergillus fumigatus* and *Saccharomyces cerevisiae*.

*S. cerevisiae* gene	*A. fumigatus* gene	Correlation coefficient
*YCR053W*	*AFUA_3G08980*	0.851750303
*YOL006C*	*AFUA_1G03500*	0.814728478
*YLR208W*	*AFUA_4G06090*	0.810778356
*YJR160C*	*AFUA_8G07240*	0.807607644
*YKL064W*	*AFUA_2G08070*	0.771984405
*YDL208W*	*AFUA_1G13570*	0.763687527
*YDL247W*	*AFUA_8G07240*	0.751799318
*YJL034W*	*AFUA_2G04620*	0.751475734
*YJL174W*	*AFUA_2G07590*	0.731359519
*YGL006W*	*AFUA_1G10880*	0.706118907
*YLR307W*	*AFUA_6G10430*	0.701428537
*YOR056C*	*AFUA_5G04000*	0.693624039
*YJR049C*	*AFUA_5G12870*	0.6873278
*YOR132W*	*AFUA_5G07150*	0.684358232
*YDR028C*	*AFUA_8G02720*	0.681519128
*YKL059C*	*AFUA_2G06220*	0.677236775
*YDR019C*	*AFUA_1G10780*	0.673590566
*YDR262W*	*AFUA_8G05360*	0.668577442
*YJR160C*	*AFUA_3G01700*	0.655149877
*YBL087C*	*AFUA_2G03380*	0.654275917
*YDR120C*	*AFUA_3G04200*	0.653965099
*YMR110C*	*AFUA_4G13500*	0.649573592
*YDR479C*	*AFUA_2G01510*	0.649340576
*YLR307W*	*AFUA_4G09940*	0.641152119
*YLL050C*	*AFUA_5G10570*	0.636927762
*YMR011W*	*AFUA_2G11520*	0.631877767
*YGR149W*	*AFUA_3G08240*	0.627253467
*YKL125W*	*AFUA_1G02590*	0.626251676
*YDL247W*	*AFUA_3G01700*	0.621782331
*YHR165C*	*AFUA_2G03030*	0.616638486
*YLR355C*	*AFUA_3G14490*	0.615946987
*YLR307W*	*AFUA_3G07210*	0.615345397
*YNL156C*	*AFUA_4G07680*	0.605449075
*YOL157C*	*AFUA_8G07070*	0.600254438
*YPL196W*	*AFUA_3G08740*	0.596649007
*YOL156W*	*AFUA_2G11520*	0.589475104
*YGL055W*	*AFUA_7G05920*	0.583471232
*YER094C*	*AFUA_4G07420*	0.580111415
*YER026C*	*AFUA_4G13680*	0.579632053
*YKR031C*	*AFUA_3G05630*	0.578947614
*YJR041C*	*AFUA_1G13200*	0.577042969
*YCL057C-A*	*AFUA_1G13195*	0.56703028
*YKL167C*	*AFUA_6G12620*	0.566497151
*YDR334W*	*AFUA_7G02370*	0.556810391
*YDL085W*	*AFUA_1G11960*	0.555058025
*YIR038C*	*AFUA_8G02500*	0.553657653
*YGL056C*	*AFUA_1G06660*	0.550891198
*YLR133W*	*AFUA_1G15930*	0.547961885
*YLR137W*	*AFUA_2G09930*	0.542448721
*YER095W*	*AFUA_1G10410*	0.540102076
*YCR090C*	*AFUA_2G15510*	0.540015424
*YIR022W*	*AFUA_3G12840*	0.539096148
*YJR048W*	*AFUA_2G13110*	0.537701308
*YDL247W*	*AFUA_8G01340*	0.536753536
*YGL119W*	*AFUA_6G04380*	0.528264148
*YDR054C*	*AFUA_5G09200*	0.520282879
*YNR036C*	*AFUA_5G10750*	0.51817226
*YJL050W*	*AFUA_4G07160*	0.517374235
*YKR036C*	*AFUA_5G13140*	0.515007541
*YML041C*	*AFUA_2G05030*	0.514819597
*YML080W*	*AFUA_1G16550*	0.512494533
*YJL093C*	*AFUA_1G14250*	0.5107227
*YDR238C*	*AFUA_1G10970*	0.509749906
*YJL197W*	*AFUA_2G14130*	0.501902821
*YER173W*	*AFUA_8G02820*	0.501581347
*YJR160C*	*AFUA_8G01340*	0.499250408
*YOL058W*	*AFUA_2G04310*	0.497155736
*YGR006W*	*AFUA_1G16990*	0.496383573
*YPR161C*	*AFUA_5G05510*	0.495551014
*YBR136W*	*AFUA_4G04760*	0.49434371
*YJR049C*	*AFUA_2G01350*	0.493221085
*YPR060C*	*AFUA_5G13130*	0.490832735
*YDR055W*	*AFUA_4G06820*	0.482973721
*YLR104W*	*AFUA_5G04040*	0.47610876
*YIL129C*	*AFUA_6G11010*	0.473123981
*YDR300C*	*AFUA_2G07570*	0.469287567
*YOL156W*	*AFUA_7G00950*	0.46726022
*YGL201C*	*AFUA_5G10890*	0.462753539
*YKR086W*	*AFUA_1G03820*	0.461061937
*YPL157W*	*AFUA_6G08610*	0.460257721
*YIL046W*	*AFUA_2G14110*	0.45798838
*YGL048C*	*AFUA_4G04660*	0.457664607
*YHR216W*	*AFUA_2G03610*	0.456902272
*YDR424C*	*AFUA_1G04850*	0.455530713
*YPR141C*	*AFUA_2G14280*	0.452133975
*YLR427W*	*AFUA_1G07150*	0.444534443
*YHL004W*	*AFUA_1G06570*	0.439699159
*YLL002W*	*AFUA_5G09540*	0.431520497
*YKR052C*	*AFUA_6G12550*	0.429429023
*YNL260C*	*AFUA_1G09000*	0.425937756
*YNL112W*	*AFUA_2G10750*	0.423331447
*YFL026W*	*AFUA_3G14330*	0.422540136
*YCL038C*	*AFUA_2G15370*	0.420548714
*YPR156C*	*AFUA_4G01140*	0.419838437
*YHL024W*	*AFUA_3G06230*	0.417835282
*YBR173C*	*AFUA_5G10740*	0.414366599
*YGR178C*	*AFUA_1G09630*	0.410618083
*YMR036C*	*AFUA_6G08200*	0.408252022
*YDL147W*	*AFUA_3G06610*	0.40670988
*YJR095W*	*AFUA_2G16930*	0.405617695
*YPL171C*	*AFUA_2G17960*	0.40043227
*YBL061C*	*AFUA_8G05620*	0.398124519
*YHL001W*	*AFUA_6G03830*	0.397471165
*YNL255C*	*AFUA_1G07630*	0.390527675
*YGL006W*	*AFUA_3G10690*	0.389206341
*YIL125W*	*AFUA_4G11650*	0.387564721
*YMR011W*	*AFUA_5G01160*	0.381368332
*YMR010W*	*AFUA_5G03510*	0.378223656
*YMR319C*	*AFUA_4G14640*	0.376638524
*YDL110C*	*AFUA_1G09160*	0.376540694
*YOL086C*	*AFUA_5G06240*	0.376281416
*YGR076C*	*AFUA_4G09000*	0.373900773
*YOR204W*	*AFUA_4G07660*	0.368301524
*YMR229C*	*AFUA_2G16040*	0.367246087
*YLR025W*	*AFUA_1G06420*	0.363738278
*YPL281C*	*AFUA_6G06770*	0.363265962
*YOR294W*	*AFUA_7G04430*	0.363077313
*YLR299W*	*AFUA_7G04760*	0.36169498
*YCL038C*	*AFUA_2G06170*	0.359699115
*YKL113C*	*AFUA_3G06060*	0.351986208
*YHL020C*	*AFUA_5G09420*	0.350312962
*YMR224C*	*AFUA_6G11410*	0.348946198
*YDR444W*	*AFUA_3G04240*	0.345942811
*YER107C*	*AFUA_1G09020*	0.34238252
*YDR162C*	*AFUA_2G03680*	0.342381673
*YNL212W*	*AFUA_3G08750*	0.341561675
*YNL097C*	*AFUA_3G11940*	0.339603963
*YKL191W*	*AFUA_6G07100*	0.337319743
*YPL030W*	*AFUA_6G02570*	0.336821966
*YNL224C*	*AFUA_3G05330*	0.336636223
*YKR024C*	*AFUA_5G11050*	0.328747023
*YPR020W*	*AFUA_1G16280*	0.323479927
*YPL171C*	*AFUA_2G04060*	0.322952534
*YOR393W*	*AFUA_6G06770*	0.321210691
*YPR088C*	*AFUA_5G03880*	0.31284654
*YGL058W*	*AFUA_6G14210*	0.312000165
*YFL059W*	*AFUA_5G08090*	0.309971957
*YBR243C*	*AFUA_2G11240*	0.307271969
*YJL048C*	*AFUA_3G06360*	0.306215007
*YNL055C*	*AFUA_4G06910*	0.305279025
*YOR058C*	*AFUA_2G16260*	0.305221634
*YGR240C*	*AFUA_4G00960*	0.304340045
*YBR279W*	*AFUA_2G11000*	0.303224
*YPL078C*	*AFUA_8G05440*	0.300712891
*YLR059C*	*AFUA_3G11820*	0.296403753
*YLR380W*	*AFUA_6G12690*	0.295562758
*YDL174C*	*AFUA_1G17520*	0.29517154
*YCL057W*	*AFUA_7G05930*	0.293762978
*YDR234W*	*AFUA_5G08890*	0.293096856
*YDR438W*	*AFUA_5G12140*	0.287954196
*YGL028C*	*AFUA_8G05610*	0.287899385
*YML004C*	*AFUA_6G07940*	0.285261452
*YOL045W*	*AFUA_2G02850*	0.284622252
*YLR429W*	*AFUA_2G14270*	0.283845667
*YDR397C*	*AFUA_3G02340*	0.283499213
*YBR260C*	*AFUA_3G06280*	0.282196097
*YCR087C-A*	*AFUA_7G04700*	0.281322207
*YMR011W*	*AFUA_7G00950*	0.280854319
*YOL148C*	*AFUA_1G16580*	0.274850272
*YDR379W*	*AFUA_1G12680*	0.273041289
*YJL151C*	*AFUA_5G10590*	0.272979399
*YML020W*	*AFUA_5G12090*	0.266526854
*YGR082W*	*AFUA_6G11380*	0.265129767
*YJR113C*	*AFUA_1G04280*	0.262789584
*YIL031W*	*AFUA_5G03200*	0.256820972
*YDR432W*	*AFUA_3G10100*	0.255963453
*YDR359C*	*AFUA_4G07560*	0.255317292
*YDR233C*	*AFUA_6G13670*	0.253861938
*YGR255C*	*AFUA_4G12930*	0.252312468
*YFR009W*	*AFUA_4G06070*	0.245534257
*YPL280W*	*AFUA_3G08490*	0.243781447
*YOR075W*	*AFUA_2G09670*	0.239487119
*YJL219W*	*AFUA_7G00950*	0.239124986
*YCR026C*	*AFUA_2G14770*	0.234276623
*YOR391C*	*AFUA_3G08490*	0.233098887
*YGL180W*	*AFUA_4G09050*	0.231299038
*YLR323C*	*AFUA_5G07720*	0.231114462
*YGL006W*	*AFUA_7G01030*	0.222088168
*YBR179C*	*AFUA_5G13392*	0.219453048
*YKR082W*	*AFUA_4G05840*	0.214144582
*YLR087C*	*AFUA_2G13520*	0.209434557
*YCL064C*	*AFUA_4G07810*	0.204494803
*YOL156W*	*AFUA_5G01160*	0.203744073
*YBR249C*	*AFUA_7G04070*	0.202306902
*YJL093C*	*AFUA_3G07540*	0.201465401
*YOL077C*	*AFUA_1G02210*	0.196710807
*YLR426W*	*AFUA_1G06280*	0.196041865
*YMR060C*	*AFUA_2G03840*	0.194299595
*YOR048C*	*AFUA_1G13730*	0.193994391
*YDL174C*	*AFUA_7G02560*	0.193117882
*YJL221C*	*AFUA_8G07070*	0.187015236
*YGR054W*	*AFUA_3G05970*	0.181434929
*YPR176C*	*AFUA_7G04460*	0.180215936
*YML003W*	*AFUA_1G09870*	0.173517378
*YKR084C*	*AFUA_2G04630*	0.171646807
*YDR270W*	*AFUA_4G12620*	0.171601357
*YOR336W*	*AFUA_2G02360*	0.164107564
*YFL058W*	*AFUA_5G02470*	0.160695353
*YNL301C*	*AFUA_2G07380*	0.151927636
*YJL221C*	*AFUA_7G06380*	0.150927464
*YGR280C*	*AFUA_7G03690*	0.150422383
*YOL089C*	*AFUA_6G01960*	0.149731857
*YGR193C*	*AFUA_3G08270*	0.14875632
*YDR328C*	*AFUA_5G06060*	0.143711765
*YPL254W*	*AFUA_2G06060*	0.143220591
*YPL206C*	*AFUA_2G00990*	0.138505935
*YEL021W*	*AFUA_2G08360*	0.13720587
*YOL068C*	*AFUA_4G12120*	0.134069861
*YJR160C*	*AFUA_7G06390*	0.131179873
*YNL310C*	*AFUA_6G08230*	0.130305791
*YIL061C*	*AFUA_5G13480*	0.126828721
*YGL163C*	*AFUA_6G12910*	0.12597657
*YGR173W*	*AFUA_5G06770*	0.123606414
*YJL092W*	*AFUA_2G03910*	0.122708164
*YJR065C*	*AFUA_5G11560*	0.121854722
*YBR023C*	*AFUA_8G05630*	0.120791924
*YBR084W*	*AFUA_3G08650*	0.118154138
*YMR197C*	*AFUA_4G10710*	0.114428905
*YMR241W*	*AFUA_5G04220*	0.114113659
*YNL097C*	*AFUA_7G01870*	0.109316946
*YGL171W*	*AFUA_1G16290*	0.103112508
*YOR365C*	*AFUA_4G13340*	0.094260129
*YHR215W*	*AFUA_6G11330*	0.092128581
*YKL013C*	*AFUA_6G02370*	0.088958318
*YOR043W*	*AFUA_4G06130*	0.087990985
*YPR062W*	*AFUA_1G05050*	0.087018417
*YDR497C*	*AFUA_2G07910*	0.078863767
*YDR477W*	*AFUA_2G01700*	0.074801159
*YLR378C*	*AFUA_5G08130*	0.074624328
*YBR060C*	*AFUA_5G08110*	0.073589072
*YGL120C*	*AFUA_5G11620*	0.069009268
*YIL063C*	*AFUA_2G10810*	0.06328373
*YOL157C*	*AFUA_7G06380*	0.06047767
*YGL043W*	*AFUA_3G07670*	0.059398891
*YMR027W*	*AFUA_5G06710*	0.058232831
*YHR215W*	*AFUA_8G01910*	0.05787337
*YDL247W*	*AFUA_7G05190*	0.05184022
*YPR048W*	*AFUA_5G07290*	0.049809159
*YBL051C*	*AFUA_5G01940*	0.048514144
*YHR148W*	*AFUA_2G08320*	0.047441878
*YJR160C*	*AFUA_7G05190*	0.045923723
*YJL039C*	*AFUA_5G12670*	0.042943521
*YNL029C*	*AFUA_5G12160*	0.031203315
*YGR267C*	*AFUA_5G03140*	0.029526621
*YNL027W*	*AFUA_1G06900*	0.02747405
*YDL247W*	*AFUA_7G06390*	0.023887472
*YHR107C*	*AFUA_5G03080*	0.020068991
*YKL165C*	*AFUA_4G03970*	0.01938034
*YNR012W*	*AFUA_2G05430*	0.019075112
*YLR085C*	*AFUA_4G04420*	0.018617278
*YPL082C*	*AFUA_1G05830*	0.017285628
*YCL064C*	*AFUA_1G06150*	0.014476469
*YNR039C*	*AFUA_1G12090*	0.011820922
*YOR216C*	*AFUA_1G08830*	0.008439713
*YOR148C*	*AFUA_4G07550*	0.006702846
*YDR420W*	*AFUA_4G00500*	0.006566287
*YPR018W*	*AFUA_5G03720*	−0.000683184
*YDR322W*	*AFUA_5G12810*	−0.002517201
*YNR015W*	*AFUA_3G08390*	−0.013154917
*YBR251W*	*AFUA_5G11540*	−0.01637247
*YHL028W*	*AFUA_5G09020*	−0.020657813
*YPL146C*	*AFUA_2G05550*	−0.023787247
*YKL127W*	*AFUA_3G11830*	−0.026619404
*YJR160C*	*AFUA_2G10910*	−0.029508087
*YIR038C*	*AFUA_1G17010*	−0.030714781
*YDL247W*	*AFUA_2G10910*	−0.033905332
*YBR204C*	*AFUA_1G03540*	−0.038361835
*YJR010W*	*AFUA_3G06530*	−0.038908707
*YMR210W*	*AFUA_6G04640*	−0.046477426
*YPR187W*	*AFUA_1G05160*	−0.048502572
*YDR037W*	*AFUA_6G07640*	−0.052027721
*YIL134W*	*AFUA_6G05170*	−0.053118026
*YLR286C*	*AFUA_5G03760*	−0.062116003
*YKL179C*	*AFUA_1G14240*	−0.062203351
*YDR109C*	*AFUA_4G04680*	−0.063717983
*YOL157C*	*AFUA_3G07380*	−0.064624439
*YIR038C*	*AFUA_2G17300*	−0.068756542
*YDL102W*	*AFUA_2G16600*	−0.088010728
*YOR389W*	*AFUA_2G01940*	−0.09375584
*YBR244W*	*AFUA_3G12270*	−0.097477745
*YJL219W*	*AFUA_2G11520*	−0.098366126
*YPL126W*	*AFUA_7G02610*	−0.101632712
*YDL189W*	*AFUA_1G09400*	−0.104331651
*YHL016C*	*AFUA_1G04870*	−0.106608293
*YLR380W*	*AFUA_4G13930*	−0.111437921
*YPR165W*	*AFUA_6G06900*	−0.117573203
*YOL062C*	*AFUA_5G07930*	−0.122120003
*YGL248W*	*AFUA_1G14890*	−0.124318908
*YLR029C*	*AFUA_1G04660*	−0.129375946
*YMR214W*	*AFUA_2G08300*	−0.130867913
*YOR124C*	*AFUA_6G12270*	−0.136050424
*YOL023W*	*AFUA_1G06520*	−0.136914472
*YPL280W*	*AFUA_3G01210*	−0.13706189
*YLR380W*	*AFUA_7G06760*	−0.141209958
*YNL241C*	*AFUA_3G08470*	−0.153108798
*YOR391C*	*AFUA_3G01210*	−0.154014157
*YOL089C*	*AFUA_6G02330*	−0.161608796
*YGL215W*	*AFUA_3G10040*	−0.170171082
*YMR137C*	*AFUA_2G15220*	−0.174259735
*YER004W*	*AFUA_6G08930*	−0.179730339
*YDR365C*	*AFUA_2G05420*	−0.181946191
*YDR529C*	*AFUA_4G06790*	−0.185927497
*YDR062W*	*AFUA_6G00300*	−0.189190157
*YOL086C*	*AFUA_7G01010*	−0.190813468
*YOL026C*	*AFUA_5G03630*	−0.19138562
*YIL033C*	*AFUA_3G10000*	−0.195925157
*YBR290W*	*AFUA_4G13740*	−0.19955841
*YAR019C*	*AFUA_4G06750*	−0.204744755
*YHL007C*	*AFUA_2G04680*	−0.20737573
*YMR078C*	*AFUA_7G05480*	−0.222707833
*YDR311W*	*AFUA_4G11690*	−0.22686955
*YOL100W*	*AFUA_3G12670*	−0.230714484
*YGR162W*	*AFUA_2G09490*	−0.232235851
*YGL255W*	*AFUA_1G01550*	−0.248116851
*YPL038W*	*AFUA_6G01910*	−0.252388553
*YCL035C*	*AFUA_1G06100*	−0.255759517
*YNL082W*	*AFUA_2G13410*	−0.259394819
*YDR062W*	*AFUA_1G11890*	−0.263523207
*YOR390W*	*AFUA_2G16210*	−0.269616243
*YJR141W*	*AFUA_3G07970*	−0.2738444
*YOR365C*	*AFUA_2G17650*	−0.274036262
*YDL116W*	*AFUA_1G10860*	−0.278315121
*YKL020C*	*AFUA_1G12550*	−0.278379939
*YDL174C*	*AFUA_3G06820*	−0.286419799
*YBR029C*	*AFUA_1G07010*	−0.296926336
*YLR096W*	*AFUA_1G11080*	−0.297540349
*YFL028C*	*AFUA_6G05080*	−0.303502181
*YOR089C*	*AFUA_3G10740*	−0.30892361
*YPR010C*	*AFUA_2G13000*	−0.309890067
*YJL219W*	*AFUA_5G01160*	−0.321415181
*YNL097C*	*AFUA_4G11660*	−0.329160424
*YMR116C*	*AFUA_4G13170*	−0.329415498
*YPL280W*	*AFUA_5G01430*	−0.331554435
*YHR091C*	*AFUA_2G14030*	−0.337931682
*YIL030C*	*AFUA_2G08650*	−0.350942099
*YFR037C*	*AFUA_7G05510*	−0.356342064
*YOR391C*	*AFUA_5G01430*	−0.356843862
*YDL174C*	*AFUA_1G00510*	−0.402217656
*YML121W*	*AFUA_5G09650*	−0.410825283
*YFL060C*	*AFUA_2G08580*	−0.411505108
*YDR301W*	*AFUA_8G04040*	−0.413112754
*YJL221C*	*AFUA_3G07380*	−0.413321446
*YLR326W*	*AFUA_5G12410*	−0.458648708
*YBL108C-A*	*AFUA_4G03360*	−0.487139446
*YGR165W*	*AFUA_5G08380*	−0.519339895
*YKL157W*	*AFUA_4G09030*	−0.604021491
*YNL334C*	*AFUA_2G08580*	−0.720610634
