# Supplementary material for: Conservation of Nucleosome Positions in Duplicated and Orthologous Gene Pairs
Source: ScientificWorldJournal. 2012 Feb 15;2012:298174. doi: 10.1100/2012/298174 (PMC3289889; doi:10.1100/2012/298174)
Supplement: Supplementary file 1 — Ortholog cluster between Aspergillus fumigatus and Saccharomyces cerevisiae genes. [file 298174.f1.docx]

| Supplementary Table 1. Orthologous gene clusters in *Aspergillus fumigatus* and *Saccharomyces cerevisiae*. | | | |
| --- | --- | --- | --- |
| Gene name | *S. cerevisiae* gene | *A. fumigatus* gene | Function |
| *ARG2* | *YJL071W* | *AFUA_2G11490* | Acetylglutamate synthase (glutamate N-acetyltransferase), mitochondrial enzyme that catalyzes the first step in the biosynthesis of the arginine precursor ornithine; forms a complex with Arg5,6p |
| *ARO8* | *YGL202W* | *AFUA_2G13630* | Aromatic aminotransferase Aro8 |
| *ASN1* | *YGR124W YPR145W* | *AFUA_4G06900* | Asparagine synthetase Asn2 |
| *BNA4* | *YBL098W* | *AFUA_6G07340* | Kynurenine 3-monooxygenase |
| *CHA1* | *YCL064C* | *AFUA_1G06150 AFUA_4G07810* | Catabolic L-serine deaminase, catalyzes the degradation of both L-serine and L-threonine; required to use serine or threonine as the sole nitrogen source, transcriptionally induced by serine and threonine |
| *CYS4* | *YGR155W* | *AFUA_2G07620* | Cystathionine beta-synthase |
| *DPH2* | *YKL191W* | *AFUA_6G07100* | Diphthamide biosynthesis protein Dph2 |
| *DPH5* | *YLR172C* | *AFUA_1G14020* | Diphthine synthase |
| *DPL1* | *YDR294C* | *AFUA_4G10470* | Dihydrosphingosine phosphate lyase, regulates intracellular levels of sphingolipid long-chain base phosphates (LCBPs), degrades phosphorylated long chain bases, prefers C16 dihydrosphingosine-l-phosphate as a substrate |
| *DYS1* | *YHR068W* | *AFUA_5G01740* | Deoxyhypusine synthase, catalyzes formation of deoxyhypusine, the first step in hypusine biosynthesis; triggers posttranslational hypusination of translation elongation factor eIF-5A and regulates its intracellular levels; tetrameric |
| *GAD1* | *YMR250W* | *AFUA_6G13490 AFUA_8G06020* | Glutamate decarboxylase |
| *GLO1* | *YML004C* | *AFUA_6G07940* | Lactoylglutathione lyase |
| *GLY1* | *YEL046C* | *AFUA_2G14930* | Alanine racemase |
| *HIS1* | *YER055C* | *AFUA_7G04500* | ATP phosphoribosyltransferase, a hexameric enzyme, catalyzes the first step in histidine biosynthesis; mutations cause histidine auxotrophy and sensitivity to Cu, Co, and Ni salts; transcription is regulated by general amino acid control |
| *HIS2* | *YFR025C* | *AFUA_4G04030* | Histidinol-phosphatase |
| *HIS4* | *YCL030C* | *AFUA_1G14570* | Phosphoribosyl-AMP cyclohydrolase |
| *HIS5* | *YIL116W* | *AFUA_1G14090* | Histidinol-phosphate aminotransferase |
| *HIS6* | *YIL020C* | *AFUA_5G06160* | 5-proFAR isomerase His6 |
| *HIS7* | *YBR248C* | *AFUA_2G06230* | Glutamine amidotransferase:cyclase |
| *HOM2* | *YDR158W* | *AFUA_3G06830* | Aspartic beta semi-aldehyde dehydrogenase, catalyzes the second step in the common pathway for methionine and threonine biosynthesis; expression regulated by Gcn4p and the general control of amino acid synthesis |
| *HOM3* | *YER052C* | *AFUA_5G05590* | Aspartate kinase (L-aspartate 4-P-transferase); cytoplasmic enzyme that catalyzes the first step in the common pathway for methionine and threonine biosynthesis; expression regulated by Gcn4p and the general control of amino acid synthesis |
| *HOM6* | *YJR139C* | *AFUA_3G11640* | Homoserine dehydrogenase |
| *LEU4* | *YOR108W YNL104C* | *AFUA_1G15000* | Alpha-isopropylmalate synthase II (2-isopropylmalate synthase), catalyzes the first step in the leucine biosynthesis pathway; the minor isozyme, responsible for the residual alpha-IPMS activity detected in a leu4 null mutant |
| *LYS12* | *YIL094C* | *AFUA_6G07390* | Homo-isocitrate dehydrogenase, an NAD-linked mitochondrial enzyme required for the fourth step in the biosynthesis of lysine, in which homo-isocitrate is oxidatively decarboxylated to alpha-ketoadipate |
| *MET7* | *YOR241W* | *AFUA_4G08060* | Folylpolyglutamate synthetase, catalyzes extension of the glutamate chains of the folate coenzymes, required for methionine synthesis and for maintenance of mitochondrial DNA |
| *OXP1* | *YKL215C* | *AFUA_6G14330* | 5-oxoprolinase; enzyme is ATP-dependent and functions as a dimer; similar to mouse Oplah gene; green fluorescent protein (GFP)-fusion protein localizes to the cytoplasm |
| *PKP1* | *YIL042C* | *AFUA_2G13600* | Mitochondrial protein kinase involved in negative regulation of pyruvate dehydrogenase complex activity by phosphorylating the ser-133 residue of the Pda1p subunit; acts in concert with kinase Pkp2p and phosphatases Ptc5p and Ptc6p |
| *PRO1* | *YHR033W YDR300C* | *AFUA_2G07570* | Gamma-glutamyl kinase, catalyzes the first step in proline biosynthesis |
| *PRO2* | *YOR323C* | *AFUA_2G07350* | Gamma-glutamyl phosphate reductase, catalyzes the second step in proline biosynthesis |
| *SAM1* | *YLR180W YDR502C* | *AFUA_1G10630* | S-adenosylmethionine synthetase, catalyzes transfer of the adenosyl group of ATP to the sulfur atom of methionine; one of two differentially regulated isozymes (Sam1p and Sam2p) |
| *SER2* | *YGR208W* | *AFUA_3G06550* | Phosphoserine phosphatase of the phosphoglycerate pathway, involved in serine and glycine biosynthesis, expression is regulated by the available nitrogen source |
| *SER3* | *YER081W YIL074C* | *AFUA_5G05500* | 3-phosphoglycerate dehydrogenase, catalyzes the first step in serine and glycine biosynthesis; isozyme of Ser33p |
| *STR2* | *YLL058W YML082W YJR130C* | *AFUA_3G05480* | Putative protein of unknown function with similarity to Str2p, which is a cystathionine gamma-synthase important in sulfur metabolism; YLL058W is not an essential gene |
| *STR3* | *YGL184C* | *AFUA_4G03950* | Cystathionine beta-lyase, converts cystathionine into homocysteine |
| *THR1* | *YHR025W* | *AFUA_5G05820* | Homoserine kinase, conserved protein required for threonine biosynthesis; expression is regulated by the GCN4-mediated general amino acid control pathway |
| *TRM5* | *YHR070W* | *AFUA_3G08030* | TRNA methyltransferase Trm5 |
| *UGA2* | *YBR006W* | *AFUA_3G00110 AFUA_4G08170* | Succinate semialdehyde dehydrogenase involved in the utilization of gamma-aminobutyrate as a nitrogen source; part of the 4-aminobutyrate and glutamate degradation pathways; localized to the cytoplasm |
|  | *YHR112C* | *AFUA_3G01170 AFUA_5G13810 AFUA_7G01590* | Putative protein of unknown function; green fluorescent protein (GFP)-fusion protein localizes to the cytoplasm |
|  | *YGR012W* | *AFUA_8G05200* | Putative cysteine synthase, localized to the mitochondrial outer membrane |
|  | *YML096W* | *AFUA_4G12480* | Putative protein of unknown function with similarity to asparagine synthetases; green fluorescent protein (GFP)-fusion protein localizes to the cytoplasm; YML096W is not an essential gene and partially overlaps the verified gene RAD10 |
|  | *YFR018C* | *AFUA_4G08280* | Putative protein of unknown function |
| *APA1* | *YCL050C YDR530C* | *AFUA_8G05000* | Bis(5'-nucleosyl)-tetraphosphatase |
| *GUK1* | *YDR454C* | *AFUA_1G08840* | Guanylate kinase, converts GMP to GDP; required for growth and mannose outer chain elongation of cell wall N-linked glycoproteins |
| *HNT1* | *YDL125C* | *AFUA_6G12680* | Adenosine 5'-monophosphoramidase; interacts physically and genetically with Kin28p, a CDK and TFIIK subunit, and genetically with CAK1; member of the histidine triad superfamily of nucleotide-binding proteins and similar to Hint |
| *NMD5* | *YJR132W* | *AFUA_2G10010* | Karyopherin, a carrier protein involved in nuclear import of proteins; importin beta homolog |
| *RNH201* | *YNL072W* | *AFUA_1G07600* | Ribonuclease HI large subunit |
| *RNH70* | *YGR276C* | *AFUA_2G14950* | 3'-5' exoribonuclease; required for maturation of 3' ends of 5S rRNA and tRNA-Arg3 from dicistronic transcripts |
| *RNY1* | *YPL123C* | *AFUA_1G16600 AFUA_3G11220* | Ribonuclease T2 family |
| *YSA1* | *YBR111C* | *AFUA_5G08240* | Nudix hydrolase family member with ADP-ribose pyrophosphatase activity; shown to metabolize O-acetyl-ADP-ribose to AMP and acetylated ribose 5'-phosphate |
| *GRX5* | *YPL059W* | *AFUA_4G05950* | Glutaredoxin Grx5 |
| *GRX6* | *YPL156C YBR014C YDL010W* | *AFUA_1G09090* | Cis-golgi localized monothiol glutaredoxin that binds an iron-sulfur cluster; more similar in activity to dithiol than other monothiol glutaredoxins; involved in the oxidative stress response; functional overlap with GRX7 |
| *PAC1* | *YOR269W* | *AFUA_6G12970* | Nuclear migration protein NudF |
| *TRR1* | *YHR106W YDR353W* | *AFUA_4G12990* | Mitochondrial thioredoxin reductase involved in protection against oxidative stress, required with Glr1p to maintain the redox state of Trx3p; contains active-site motif present in prokaryotic orthologs; binds NADPH and FAD |
| *AAH1* | *YNL141W* | *AFUA_2G09150* | Adenosine deaminase |
| *ADE1* | *YAR015W* | *AFUA_3G06210* | Phosphoribosyl-aminoimidazole-succinocarboxamide synthase |
| *ADE13* | *YLR359W* | *AFUA_2G11940* | Adenylosuccinate lyase Ade13 |
| *ADE16* | *YLR028C YMR120C* | *AFUA_4G07690* | Phosphoribosylaminoimidazolecarboxamide formyltransferase/IMP cyclohydrolase |
| *ADE2* | *YOR128C* | *AFUA_4G12600* | Phosphoribosyl-aminoimidazole carboxylase |
| *ADE4* | *YMR300C* | *AFUA_6G03750* | Amidophosphoribosyltransferase |
| *ADE5,7* | *YGL234W* | *AFUA_6G04730* | Bifunctional purine biosynthetic protein Ade1 |
| *ADO1* | *YJR105W* | *AFUA_5G06390* | Adenosine kinase, required for the utilization of S-adenosylmethionine (AdoMet); may be involved in recycling adenosine produced through the methyl cycle |
| *APT1* | *YML022W YDR441C* | *AFUA_7G02310* | Apparent pseudogene, not transcribed or translated under normal conditions; encodes a protein with similarity to adenine phosphoribosyltransferase, but artificially expressed protein exhibits no enzymatic activity |
| *DAL1* | *YIR027C* | *AFUA_2G02250* | Allantoinase, converts allantoin to allantoate in the first step of allantoin degradation; expression sensitive to nitrogen catabolite repression |
| *DAL2* | *YIR029W* | *AFUA_3G12560* | Allantoicase, converts allantoate to urea and ureidoglycolate in the second step of allantoin degradation; expression sensitive to nitrogen catabolite repression and induced by allophanate, an intermediate in allantoin degradation |
| *DAL3* | *YIR032C* | *AFUA_8G04760* | Ureidoglycolate hydrolase |
| *DUR1,2* | *YBR208C* | *AFUA_1G15520* | Urea amidolyase |
| *GUA1* | *YMR217W* | *AFUA_3G01110* | GMP synthase, an enzyme that catalyzes the second step in the biosynthesis of GMP from inosine 5'-phosphate (IMP); transcription is not subject to regulation by guanine but is negatively regulated by nutrient starvation |
| *IMD2* | *YML056C YHR216W YLR432W* | *AFUA_2G03610* | IMP dehydrogenase |
| *MEU1* | *YLR017W* | *AFUA_6G08720* | Methylthioadenosine phosphorylase activity; regulates ADH2 gene expression |
| *PNP1* | *YLR209C* | *AFUA_6G05320* | Purine nucleoside phosphorylase I, inosine and guanosine-specific |
| *URH1* | *YDR400W* | *AFUA_1G05530* | Uridine nucleosidase Urh1 |
| *CDD1* | *YLR245C* | *AFUA_8G02770* | Cytidine deaminase |
| *DCD1* | *YHR144C* | *AFUA_2G06240* | Deoxycytidylate deaminase |
| *DUT1* | *YBR252W* | *AFUA_1G02890* | DUTPase (Dut), putaive |
| *FCY1* | *YPR062W* | *AFUA_1G05050* | Cytosine deaminase, zinc metalloenzyme that catalyzes the hydrolytic deamination of cytosine to uracil; of biomedical interest because it also catalyzes the deamination of 5-fluorocytosine to form anticancer drug 5-fluorouracil (5FU) |
| *FSH2* | *YMR222C YOR280C* | *AFUA_3G06620* | Dihydrofolate reductase |
| *PRS3* | *YHL011C* | *AFUA_4G10790* | 5-phospho-ribosyl-1(alpha)-pyrophosphate synthetase, synthesizes PRPP, which is required for nucleotide, histidine, and tryptophan biosynthesis; one of five related enzymes, which are active as heteromultimeric complexes |
| *PRS5* | *YOL061W* | *AFUA_3G13380* | 5-phospho-ribosyl-1(alpha)-pyrophosphate synthetase, synthesizes PRPP, which is required for nucleotide, histidine, and tryptophan biosynthesis; one of five related enzymes, which are active as heteromultimeric complexes |
| *THI11* | *YJR156C YDL244W YFL058W YNL332W* | *AFUA_5G02470* | Protein involved in synthesis of the thiamine precursor hydroxymethylpyrimidine (HMP); member of a subtelomeric gene family including THI5, THI11, THI12, and THI13 |
| *URA10* | *YML106W YMR271C* | *AFUA_2G11290* | Major orotate phosphoribosyltransferase isozyme that catalyzes the fifth enzymatic step in de novo biosynthesis of pyrimidines, converting orotate into orotidine-5'-phosphate; minor OPRTase encoded by URA10 |
| *URA3* | *YEL021W* | *AFUA_2G08360* | Orotidine-5'-phosphate decarboxylase, catalyzes the sixth enzymatic step in the de novo biosynthesis of pyrimidines, converting OMP into uridine monophosphate (UMP); converts 5-FOA into 5-fluorouracil, a toxic compound |
| *URA4* | *YLR420W* | *AFUA_8G00850* | Dihydroorotase, catalyzes the third enzymatic step in the de novo biosynthesis of pyrimidines, converting carbamoyl-L-aspartate into dihydroorotate |
| *URA7* | *YBL039C YJR103W* | *AFUA_7G05210* | Major CTP synthase isozyme (see also URA8), catalyzes the ATP-dependent transfer of the amide nitrogen from glutamine to UTP, forming CTP, the final step in de novo biosynthesis of pyrimidines; involved in phospholipid biosynthesis |
| *URK1* | *YNR012W* | *AFUA_2G05430* | Uridine/cytidine kinase, component of the pyrimidine ribonucleotide salvage pathway that converts uridine into UMP and cytidine into CMP; involved in the pyrimidine deoxyribonucleotide salvage pathway, converting deoxycytidine into dCMP |
| *ACB1* | *YGR037C* | *AFUA_2G11060* | Acyl CoA binding protein family |
| *BTS1* | *YPL069C* | *AFUA_6G09770 AFUA_1G13160 AFUA_8G02400* | Geranylgeranyl diphosphate synthase |
| *CKI1* | *YLR133W YDR147W* | *AFUA_1G15930* | Choline kinase, catalyzing the first step in phosphatidylcholine synthesis via the CDP-choline (Kennedy pathway); exhibits some ethanolamine kinase activity contributing to phosphatidylethanolamine synthesis via the CDP-ethanolamine pathway |
| *CRD1* | *YDL142C* | *AFUA_6G04460* | Cardiolipin synthase; produces cardiolipin, which is a phospholipid of the mitochondrial inner membrane that is required for normal mitochondrial membrane potential and function; also required for normal vacuolar ion homeostasis |
| *CYB5* | *YNL111C* | *AFUA_2G04710 AFUA_3G03970 AFUA_5G10060* | Cytochrome b5 reductase |
| *ERG13* | *YML126C* | *AFUA_3G10660 AFUA_8G07210* | 3-hydroxy-3-methylglutaryl-CoA synthase, catalyzes the formation of HMG-CoA from acetyl-CoA and acetoacetyl-CoA; involved in the second step in mevalonate biosynthesis |
| *ERG24* | *YNL280C* | *AFUA_1G03150 AFUA_1G05720* | C-14 sterol reductase, acts in ergosterol biosynthesis; mutants accumulate the abnormal sterol ignosterol (ergosta-8,14 dienol), and are viable under anaerobic growth conditions but inviable on rich medium under aerobic conditions |
| *ERG26* | *YGL001C* | *AFUA_2G15030 AFUA_2G17400* | C-3 sterol dehydrogenase, catalyzes the second of three steps required to remove two C-4 methyl groups from an intermediate in ergosterol biosynthesis |
| *ERG4* | *YGL012W* | *AFUA_1G07140 AFUA_5G14350* | C-24(28) sterol reductase, catalyzes the final step in ergosterol biosynthesis; mutants are viable, but lack ergosterol |
| *FAA1* | *YOR317W YIL009W YMR246W* | *AFUA_2G09910* | Fatty acid activator Faa4 |
| *FMS1* | *YMR020W* | *AFUA_3G00100 AFUA_3G13510 AFUA_8G01470* | Flavin-containing amine oxidase |
| *GPI11* | *YDR302W* | *AFUA_2G01070* | ER membrane protein involved in a late step of glycosylphosphatidylinositol anchor assembly; involved in the addition of phosphoethanolamine to the multiply mannosylated GPI intermediate; human PIG-Fp is a functional homolog |
| *HES1* | *YOR237W YPL145C* | *AFUA_3G11750* | Oxysterol binding protein (Osh5) |
| *IFA38* | *YBR159W* | *AFUA_2G11540* | Ketoreductase |
| *INP52* | *YOR109W YNL106C* | *AFUA_7G03680* | SacI domain and endonuclease/exonuclease/phosphatase family protein |
| *IRC24* | *YIR035C YIR036C* | *AFUA_3G02370* | Short-chain dehydrogenase/reductase |
| *LAP2* | *YNL045W* | *AFUA_2G07520* | Leukotriene A4 hydrolase |
| *LCB1* | *YMR296C* | *AFUA_6G12390* | Component of serine palmitoyltransferase, responsible along with Lcb2p for the first committed step in sphingolipid synthesis, which is the condensation of serine with palmitoyl-CoA to form 3-ketosphinganine |
| *LCB2* | *YDR062W* | *AFUA_1G11890 AFUA_6G00300* | Component of serine palmitoyltransferase, responsible along with Lcb1p for the first committed step in sphingolipid synthesis, which is the condensation of serine with palmitoyl-CoA to form 3-ketosphinganine |
| *LCB3* | *YJL134W YKR053C* | *AFUA_5G14050* | Dihydrosphingosine 1-phosphate phosphatase, membrane protein involved in sphingolipid metabolism; has similarity to Lcb3p |
| *LCB4* | *YLR260W YOR171C* | *AFUA_1G10980* | Sphingosine kinase (SphK) |
| *MUQ1* | *YGR007W* | *AFUA_4G05940* | Choline phosphate cytidylyltransferase, catalyzes the second step of phosphatidylethanolamine biosynthesis; involved in the maintenance of plasma membrane; similar to mammalian CTP: phosphocholine cytidylyl-transferases |
| *MVD1* | *YNR043W* | *AFUA_4G07130* | Mevalonate pyrophosphate decarboxylase, essential enzyme involved in the biosynthesis of isoprenoids and sterols, including ergosterol; acts as a homodimer |
| *NCR1* | *YPL006W* | *AFUA_6G09980* | Patched sphingolipid transporter (Ncr1) |
| *OSH2* | *YAR042W YDL019C* | *AFUA_7G02480* | Member of an oxysterol-binding protein family with seven members in S. cerevisiae; family members have overlapping, redundant functions in sterol metabolism and collectively perform a function essential for viability |
| *OSH3* | *YHR073W* | *AFUA_3G05880* | Member of an oxysterol-binding protein family with seven members in S. cerevisiae; family members have overlapping, redundant functions in sterol metabolism and collectively perform a function essential for viability |
| *OSH6* | *YHR001W YKR003W* | *AFUA_2G03790* | Member of an oxysterol-binding protein family with overlapping, redundant functions in sterol metabolism and which collectively perform a function essential for viability; GFP-fusion protein localizes to the cell periphery |
| *PDR16* | *YNL264C YNL231C* | *AFUA_5G13000* | CRAL/TRIO domain protein |
| *PLB1* | *YOL011W YMR006C YMR008C* | *AFUA_5G01340 AFUA_3G14680 AFUA_4G08720* | Phospholipase B involved in phospholipid metabolism; displays transacylase activity in vitro; overproduction confers resistance to lysophosphatidylcholine |
| *SCS3* | *YGL126W* | *AFUA_1G05870* | Protein required for inositol prototrophy, identified as an ortholog of the FIT family of proteins involved in triglyceride droplet biosynthesis; disputed role in the synthesis of inositol phospholipids from inositol |
| *SEC14* | *YMR079W YKL091C* | *AFUA_3G09910* | Phosphatidylinositol/phosphatidylcholine transfer protein; involved in regulating PtdIns, PtdCho, and ceramide metabolism, products of which regulate intracellular transport and UPR; functionally homologous to mammalian PITPs |
| *TGL1* | *YKL140W* | *AFUA_5G08960* | Steryl ester hydrolase, one of three gene products responsible for steryl ester hydrolase activity and involved in sterol homeostasis; localized to lipid particle membranes |
| *TGL2* | *YDR058C* | *AFUA_7G02040* | Triacylglycerol lipase |
| *YEH1* | *YLL012W YLR020C* | *AFUA_2G14600* | Steryl ester hydrolase, one of three gene products responsible for steryl ester hydrolase activity and involved in sterol homeostasis; localized to lipid particle membranes |
|  | *YML131W* | *AFUA_1G15610 AFUA_7G04530* | Putative protein of unknown function with similarity to medium chain dehydrogenase/reductases; expression induced by stresses including osmotic shock, DNA damaging agents, and other chemicals; GFP-fusion protein localizes to the cytoplasm |
|  | *YJR107W* | *AFUA_6G06510* | Putative protein of unknown function; has sequence or structural similarity to lipases |
|  | *YJL068C* | *AFUA_5G09860* | Non-essential intracellular esterase that can function as an S-formylglutathione hydrolase; may be involved in the detoxification of formaldehyde, which can be metabolized to S-formylglutathione; similar to human esterase D |
| *ABZ1* | *YNR033W* | *AFUA_6G04820* | Para-aminobenzoate synthase PabaA |
| *BIO2* | *YGR286C* | *AFUA_6G03670* | Biotin synthase |
| *BIO3* | *YNR058W* | *AFUA_1G16810 AFUA_7G06840* | 7,8-diamino-pelargonic acid aminotransferase that mediate biotin synthesis |
| *BNA1* | *YJR025C* | *AFUA_8G04650* | 3-hydroxyanthranilic acid dioxygenase, required for the de novo biosynthesis of NAD from tryptophan via kynurenine; expression regulated by Hst1p |
| *BNA2* | *YJR078W* | *AFUA_3G14250 AFUA_4G09830* | Indoleamine 2,3-dioxygenase pyrrole 2,3-dioxygenase) |
| *BNA5* | *YLR231C* | *AFUA_2G10360 AFUA_4G09840* | Kynureninase |
| *BUD16* | *YEL029C YNR027W* | *AFUA_1G02900* | Pyridoxal kinase |
| *COQ4* | *YDR204W* | *AFUA_3G06410* | Coenzyme Q biosynthesis protein Coq4, putative |
| *COQ6* | *YGR255C* | *AFUA_4G12930* | Ubiquinone biosynthesis monooxgenase (Coq6) |
| *FAU1* | *YER183C* | *AFUA_7G05990* | 5,10-methenyltetrahydrofolate synthetase, involved in folic acid biosynthesis |
| *FOL1* | *YNL256W* | *AFUA_2G09840* | Folic acid synthesis protein |
| *FOL2* | *YGR267C* | *AFUA_5G03140* | GTP cyclohydrolase I |
| *FOL3* | *YMR113W YKL132C* | *AFUA_4G06740* | Putative dihydrofolate synthetase; has similarity to Fol3p and to E. coli folylpolyglutamate synthetase/dihydrofolate synthetase; the authentic, non-tagged protein is detected in highly purified mitochondria in high-throughput studies |
| *GRE2* | *YGL157W YGL039W YOL151W YDR541C* | *AFUA_2G10280 AFUA_3G02250* | 3-methylbutanal reductase and NADPH-dependent methylglyoxal reductase (D-lactaldehyde dehydrogenase); stress induced (osmotic, ionic, oxidative, heat shock and heavy metals); regulated by the HOG pathway |
| *GSH2* | *YOL049W* | *AFUA_5G06610* | Glutathione synthetase, catalyzes the ATP-dependent synthesis of glutathione from gamma-glutamylcysteine and glycine; induced by oxidative stress and heat shock |
| *HEM4* | *YOR278W* | *AFUA_6G12800* | Uroporphyrinogen-III synthase (UroS) |
| *MET1* | *YKR069W* | *AFUA_3G06600* | Siroheme synthase |
| *MET12* | *YPL023C* | *AFUA_5G03480* | Methylenetetrahydrofolate reductase |
| *NPT1* | *YOR209C* | *AFUA_7G01880* | Nicotinate phosphoribosyltransferase, acts in the salvage pathway of NAD+ biosynthesis; required for silencing at rDNA and telomeres and has a role in silencing at mating-type loci; localized to the nucleus |
| *PAN6* | *YIL145C* | *AFUA_5G11040* | Pantoate--beta-alanine ligase |
| *PDX3* | *YBR035C* | *AFUA_5G10650* | Pyridoxamine phosphate oxidase |
| *PUS9* | *YOL066C YDL036C* | *AFUA_2G16360* | Bifunctional enzyme with DRAP deaminase and tRNA:pseudouridine synthase activity; the deaminase catalyzes the third step in riboflavin biosynthesis and the synthase catalyzes formation of pseudouridine at position 32 in cytoplasmic tRNAs |
| *RIB1* | *YBL033C* | *AFUA_1G13300* | GTP cyclohydrolase II; catalyzes the first step of the riboflavin biosynthesis pathway |
| *RIB2* | *YOL066C* | *AFUA_2G13200 AFUA_3G07440* | Bifunctional enzyme with DRAP deaminase and tRNA:pseudouridine synthase activity; the deaminase catalyzes the third step in riboflavin biosynthesis and the synthase catalyzes formation of pseudouridine at position 32 in cytoplasmic tRNAs |
| *RIB3* | *YDR487C* | *AFUA_6G13140* | 3,4-dihydroxy-2-butanone-4-phosphate synthase (DHBP synthase), required for riboflavin biosynthesis from ribulose-5-phosphate, also has an unrelated function in mitochondrial respiration |
| *RIB4* | *YOL143C* | *AFUA_6G06345* | Lumazine synthase (6,7-dimethyl-8-ribityllumazine synthase, also known as DMRL synthase); catalyzes synthesis of immediate precursor to riboflavin |
| *RIB5* | *YBR256C* | *AFUA_1G06240* | Riboflavin synthase, alpha subunit |
| *RIB7* | *YBR153W* | *AFUA_3G00360 AFUA_4G04730* | Diaminohydroxyphoshoribosylaminopyrimidine deaminase; catalyzes the second step of the riboflavin biosynthesis pathway |
| *SAH1* | *YER043C* | *AFUA_1G10130* | S-adenosyl-L-homocysteine hydrolase, catabolizes S-adenosyl-L-homocysteine which is formed after donation of the activated methyl group of S-adenosyl-L-methionine to an acceptor |
| *SER1* | *YOR184W* | *AFUA_6G04970* | 3-phosphoserine aminotransferase, catalyzes the formation of phosphoserine from 3-phosphohydroxypyruvate, required for serine and glycine biosynthesis; regulated by the general control of amino acid biosynthesis mediated by Gcn4p |
| *THI6* | *YPL214C* | *AFUA_2G08970* | Bifunctional enzyme with thiamine-phosphate pyrophosphorylase and 4-methyl-5-beta-hydroxyethylthiazole kinase activities, required for thiamine biosynthesis; GFP-fusion protein localizes to the cytoplasm in a punctate pattern |
| *THI80* | *YOR143C* | *AFUA_7G05410* | Thiamine pyrophosphokinase Thi80 |
| *UBA4* | *YHR111W* | *AFUA_5G10480* | Protein that activates Urm1p before its conjugation to proteins (urmylation); one target is the thioredoxin peroxidase Ahp1p, suggesting a role of urmylation in the oxidative stress response |
|  | *YHR003C YKL027W* | *AFUA_5G08610* | Protein of unknown function, localized to the mitochondrial outer membrane |
|  | *YJR142W* | *AFUA_5G11110 AFUA_7G06170* | Putative protein of unknown function; GST fusion protein is a Dbf2-Mob1 phosphoylation target in a proteome chip analysis; synthetic lethal with PH085 deletion; plays a role in restricting Ty1 transposition |
| *ALO1* | *YML086C* | *AFUA_1G14950* | Sugar 1,4-lactone oxidase |
| *ARA1* | *YBR149W* | *AFUA_4G11730* | Glycerol dehydrogenase (GldB) |
| *AYR1* | *YIL124W* | *AFUA_2G14460 AFUA_4G04530 AFUA_6G03370* | Short-chain dehydrogenase/reductase |
| *BCH1* | *YMR237W YOR299W* | *AFUA_3G12800* | Clathrin-coated vesiclec protein (Bud7) |
| *BSC1* | *YIR019C YDL037C* | *AFUA_2G11210* | GPI-anchored cell surface glycoprotein required for pseudohyphal formation, invasive growth, flocculation, and biofilms; transcriptionally regulated by the MAPK pathway and the cAMP pathway (via Flo8p) |
| *CTS2* | *YDR371W* | *AFUA_3G11280 AFUA_7G08490 AFUA_8G01410* | Class V chitinase ChiB1 |
| *DSF1* | *YEL070W YNR073C* | *AFUA_4G14450* | Deletion suppressor of mpt5 mutation |
| *EMI2* | *YCL040W YDR516C* | *AFUA_2G16330 AFUA_6G02230* | Glucokinase GlkA |
| *GAL10* | *YHR210C YNR071C YBR019C* | *AFUA_3G05740* | Putative protein of unknown function; non-essential gene; highly expressed under anaeorbic conditions; sequence similarity to aldose 1-epimerases such as GAL10 |
| *GCY1* | *YOR120W YDR368W* | *AFUA_1G09930* | NADPH-dependent aldo-keto reductase, reduces multiple substrates including 2-methylbutyraldehyde and D,L-glyceraldehyde, expression is induced by osmotic and oxidative stress; functionally redundant with other aldo-keto reductases |
| *GOR1* | *YNL274C* | *AFUA_1G14400 AFUA_4G11840* | Glyoxylate reductase; null mutation results in increased biomass after diauxic shift; the authentic, non-tagged protein is detected in highly purified mitochondria in high-throughput studies |
| *GUT1* | *YHL032C* | *AFUA_4G11540 AFUA_6G08470* | Glycerol kinase, converts glycerol to glycerol-3-phosphate; glucose repression of expression is mediated by Adr1p and Ino2p-Ino4p; derepression of expression on non-fermentable carbon sources is mediated by Opi1p and Rsf1p |
| *HOR2* | *YER062C YIL053W* | *AFUA_1G10570* | Constitutively expressed isoform of DL-glycerol-3-phosphatase; involved in glycerol biosynthesis, induced in response to both anaerobic and, along with the Hor2p/Gpp2p isoform, osmotic stress |
| *KNH1* | *YDL049C YJL174W* | *AFUA_2G07590* | Glycoprotein involved in cell wall beta-glucan assembly; null mutation leads to severe growth defects, aberrant multibudded morphology, and mating defects |
| *MNL1* | *YHR204W* | *AFUA_7G02290* | ER glycosyl hydrolase (Edem) |
| *PCM1* | *YEL058W* | *AFUA_1G06210* | Essential N-acetylglucosamine-phosphate mutase; converts GlcNAc-6-P to GlcNAc-1-P, which is a precursor for the biosynthesis of chitin and for the formation of N-glycosylated mannoproteins and glycosylphosphatidylinositol anchors |
| *PGM3* | *YMR278W* | *AFUA_2G02120* | Phosphoglucomutase |
| *PGU1* | *YJR153W* | *AFUA_1G17220 AFUA_4G13920 AFUA_8G01970* | Extracellular endo-polygalacturonase |
| *RBK1* | *YCR036W* | *AFUA_4G14650 AFUA_5G02530* | Putative ribokinase |
| *SLG1* | *YOR008C YOL105C YNL283C* | *AFUA_4G13670* | Partially redundant sensor-transducer of the stress-activated PKC1-MPK1 signaling pathway involved in maintenance of cell wall integrity and recovery from heat shock; secretory pathway Wsc2p is required for the arrest of secretion response |
| *SOR1* | *YLR070C YDL246C YJR159W* | *AFUA_7G02550 AFUA_1G11030 AFUA_8G02000* | Sorbitol/xylitol dehydrogenase |
| *XKS1* | *YGR194C* | *AFUA_5G09840* | D-xylulose kinase |
|  | *YJR096W* | *AFUA_5G08290 AFUA_2G02950 AFUA_3G14270* | Aldo-keto reductase |
|  | *YDL124W* | *AFUA_1G07650* | NADPH-dependent alpha-keto amide reductase; reduces aromatic alpha-keto amides, aliphatic alpha-keto esters, and aromatic alpha-keto esters; member of the aldo-keto reductase family |
|  | *YJL218W* | *AFUA_3G03380 AFUA_3G11510* | Putative protein of unknown function, similar to bacterial galactoside O-acetyltransferases; induced by oleate in an OAF1/PIP2-dependent manner; promoter contains an oleate response element consensus sequence; non-essential gene |
|  | *YDR248C* | *AFUA_1G00530 AFUA_4G12050* | Putative protein of unknown function; sequence similarity to bacterial and human gluconokinase; green fluorescent protein (GFP)-fusion protein localizes to the cytoplasm; upregulated by deletion of the RNAP-II associated factor, PAF1 |
|  | *YBL086C* | *AFUA_1G05655* | Protein of unknown function; green fluorescent protein (GFP)-fusion protein localizes to the cell periphery |
| *DIA3* | *YDL024C YHR215W YAR071W YBR092C YBR093C* | *AFUA_6G11330 AFUA_8G01910* | One of three repressible acid phosphatases, a glycoprotein that is transported to the cell surface by the secretory pathway by phosphate starvation |
| *GTR1* | *YML121W* | *AFUA_5G09650* | Cytoplasmic GTP binding protein and negative regulator of the Ran/Tc4 GTPase cycle; component of GSE complex, which is required for sorting of Gap1p; involved in phosphate transport and telomeric silencing; similar to human RagA and RagB |
| *PHO81* | *YGR233C* | *AFUA_4G06020* | Cyclin-dependent kinase inhibitor, regulates Pho80p-Pho85p and Pcl7p-Pho85p cyclin-CDK complexes in response to phosphate levels; inhibitory activity for Pho80p-Pho85p requires myo-D-inositol heptakisphosphate generated by Vip1p |
| *AGX1* | *YFL030W* | *AFUA_1G09470* | Aminotransferase, class V |
| *ALT1* | *YLR089C YDR111C* | *AFUA_6G07770* | Alanine transaminase (glutamic pyruvic transaminase); involved in alanine biosynthetic and catabolic processes; the authentic, non-tagged protein is detected in highly purified mitochondria in high-throughput studies |
| *AMD2* | *YDR242W* | *AFUA_8G05220 AFUA_6G05020 AFUA_6G08000 AFUA_1G14530 AFUA_3G15230 AFUA_5G01240 AFUA_4G00370 AFUA_5G00470* | General amidase |
| *BNA3* | *YJL060W* | *AFUA_4G11190* | Kynurenine aminotransferase |
| *DDI2* | *YFL061W YNL335W* | *AFUA_1G10870* | Urea hydro-lyase/cyanamide hydratase |
| *FMO1* | *YHR176W* | *AFUA_3G01002 AFUA_5G03380* | Flavin-containing monooxygenase, localized to the cytoplasmic face of the ER membrane; catalyzes oxidation of biological thiols to maintain the ER redox buffer ratio for correct folding of disulfide-bonded proteins |
| *GLT1* | *YDL171C* | *AFUA_1G07380* | Glutamate synthase Glt1 |
| *HER2* | *YMR293C* | *AFUA_3G04130* | Glutamyl-tRNA amidotransferase, A subunit |
| *ISU1* | *YOR226C YPL135W* | *AFUA_4G06770* | Conserved protein of the mitochondrial matrix, required for synthesis of mitochondrial and cytosolic iron-sulfur proteins, performs a scaffolding function in mitochondria during Fe/S cluster assembly; isu1 isu2 double mutant is inviable |
| *MET10* | *YFR030W* | *AFUA_6G08920* | Assimilatory sulfite reductase |
| *MET14* | *YKL001C* | *AFUA_1G10820* | Adenylylsulfate kinase, required for sulfate assimilation and involved in methionine metabolism |
| *MET16* | *YPR167C* | *AFUA_3G06540* | 3'-phosphoadenylsulfate reductase, reduces 3'-phosphoadenylyl sulfate to adenosine-3',5'-bisphosphate and free sulfite using reduced thioredoxin as cosubstrate, involved in sulfate assimilation and methionine metabolism |
| *MET3* | *YJR010W* | *AFUA_3G06530* | ATP sulfurylase, catalyzes the primary step of intracellular sulfate activation, essential for assimilatory reduction of sulfate to sulfide, involved in methionine metabolism |
| *NFU1* | *YKL040C* | *AFUA_1G04680* | NifU-related protein |
| *NIT2* | *YJL126W* | *AFUA_4G12240 AFUA_6G12100* | Nit protein, one of two proteins in S. cerevisiae with similarity to the Nit domain of NitFhit from fly and worm and to the mouse and human Nit protein which interacts with the Fhit tumor suppressor; nitrilase superfamily member |
| *NIT3* | *YLR351C* | *AFUA_6G13230* | Nit protein, one of two proteins in S. cerevisiae with similarity to the Nit domain of NitFhit from fly and worm and to the mouse and human Nit protein which interacts with the Fhit tumor suppressor; nitrilase superfamily member |
|  | *YJR149W* | *AFUA_5G09600 AFUA_2G09850 AFUA_4G07940* | Oxidoreductase, 2-nitropropane dioxygenase family, putative |
| *AAP1* | *YHR047C YKL157W* | *AFUA_4G09030* | Aminopeptidase yscII; may have a role in obtaining leucine from dipeptide substrates; sequence coordinates have changed since RT-PCR analysis showed that the adjacent ORF YKL158W comprises the 5' exon of APE2/YKL157W |
| *ACS2* | *YLR153C* | *AFUA_4G11080* | Acetyl-coA synthetase isoform which, along with Acs1p, is the nuclear source of acetyl-coA for histone acetylation; mutants affect global transcription; required for growth on glucose; expressed under anaerobic conditions |
| *AMD1* | *YML035C* | *AFUA_8G02860* | AMP deaminase, tetrameric enzyme that catalyzes the deamination of AMP to form IMP and ammonia; may be involved in regulation of intracellular adenine nucleotide pools |
| *DCP2* | *YNL118C* | *AFUA_5G12420* | Catalytic subunit of the Dcp1p-Dcp2p decapping enzyme complex, which removes the 5' cap structure from mRNAs prior to their degradation; member of the Nudix hydrolase family |
| *FSP2* | *YJL216C YBR299W YGR292W YGR287C YOL157C YIL172C YJL221C* | *AFUA_3G07380 AFUA_7G06380 AFUA_8G07070* | Protein of unknown function, similar to alpha-D-glucosidases; transcriptionally activated by both Pdr8p and Yrm1p, along with transporters and other genes involved in the pleiotropic drug resistance phenomenon |
| *GDB1* | *YPR184W* | *AFUA_1G02140* | Glycogen debranching enzyme Gdb1 |
| *GIP2* | *YER054C YIL045W* | *AFUA_2G13850* | Protein phosphatase regulatory subunit Gac1 |
| *GLG1* | *YKR058W* | *AFUA_4G08730* | Glg1p |
| *GLG1* | *YJL137C YKR058W* | *AFUA_1G05580* | Glycogenin |
| *GND1* | *YGR256W YHR183W* | *AFUA_6G08050* | 6-phosphogluconate dehydrogenase (decarboxylating), catalyzes an NADPH regenerating reaction in the pentose phosphate pathway; required for growth on D-glucono-delta-lactone and adaptation to oxidative stress |
| *ICL2* | *YPR006C* | *AFUA_6G02860* | 2-methylisocitrate lyase of the mitochondrial matrix, functions in the methylcitrate cycle to catalyze the conversion of 2-methylisocitrate to succinate and pyruvate; ICL2 transcription is repressed by glucose and induced by ethanol |
| *IRC21* | *YMR073C* | *AFUA_2G03880* | Heme/steroid binding protein |
| *MAK10* | *YEL053C* | *AFUA_2G09320* | Amino-acid N-acetyltransferase subunit Mak10 |
| *NDE1* | *YMR145C YDL085W* | *AFUA_1G11960* | Mitochondrial external NADH dehydrogenase, a type II NAD(P)H:quinone oxidoreductase that catalyzes the oxidation of cytosolic NADH; Nde1p and Nde2p provide cytosolic NADH to the mitochondrial respiratory chain |
| *NQM1* | *YGR043C YLR354C* | *AFUA_5G09230* | Transaldolase |
| *NTH1* | *YBR001C YDR001C* | *AFUA_4G13530* | Neutral trehalase, degrades trehalose; required for thermotolerance and may mediate resistance to other cellular stresses; may be phosphorylated by Cdc28p |
| *OYE2* | *YHR179W YPL171C* | *AFUA_2G04060 AFUA_2G17960* | Old Yellow Enzyme |
| *PMU1* | *YKL128C* | *AFUA_1G10590 AFUA_2G01840* | GPI anchored protein |
| *PPG1* | *YNR032W* | *AFUA_5G11370* | Ser/Thr protein phosphatase |
| *SKI8* | *YGL213C* | *AFUA_1G08860* | Ski complex component and WD-repeat protein, mediates 3'-5' RNA degradation by the cytoplasmic exosome; also required for meiotic double-strand break recombination; null mutants have superkiller phenotype |
| *TAH18* | *YPR048W* | *AFUA_5G07290* | Protein of unknown function that plays a pro-death role in response to oxidative stress; highly conserved across species and similar to human protein NDOR1; allele is synthetically lethal with the pol3-13 allele of DNA polymerase delta |
| *YTP1* | *YNL237W* | *AFUA_5G06090* | Probable type-III integral membrane protein of unknown function, has regions of similarity to mitochondrial electron transport proteins |
|  | *YEL020C* | *AFUA_8G05230* | 2-hydroxyphytanoyl-CoA lyase |
|  | *YMR244W* | *AFUA_1G13940* | Putative protein of unknown function |
|  | *YOR356W* | *AFUA_3G10110* | Mitochondrial protein with similarity to flavoprotein-type oxidoreductases; found in a large supramolecular complex with other mitochondrial dehydrogenases |
| *AAD14* | *YCR107W YDL243C YNL331C* | *AFUA_2G11250* | Aryl-alcohol dehydrogenase Aad14 |
| *ADH1* | *YMR083W YBR145W YMR303C YOL086C* | *AFUA_5G06240 AFUA_7G01010* | Alcohol dehydrogenase |
| *ADH6* | *YCR105W YMR318C* | *AFUA_3G11900* | Oxidoreductase, zinc-binding |
| *BDH1* | *YAL061W YAL060W* | *AFUA_2G15930* | (2R,3R)-2,3-butanediol dehydrogenase |
| *FDH1* | *YOR388C* | *AFUA_6G04920* | NAD-dependent formate dehydrogenase AciA/Fdh |
| *IAH1* | *YOR126C* | *AFUA_2G08920* | GDSL Lipase/Acylhydrolase family protein |
| *MSC7* | *YHR039C* | *AFUA_6G03770* | Oxidoreductase (Msc7) |
|  | *YPL088W* | *AFUA_4G11260* | Putative aryl alcohol dehydrogenase; transcription is activated by paralogous transcription factors Yrm1p and Yrr1p along with genes involved in multidrug resistance |
|  | *YIL151C YKR096W* | *AFUA_5G08220* | Protein of unknown function that may interact with ribosomes, based on co-purification experiments; green fluorescent protein (GFP)-fusion protein localizes to the nucleus and cytoplasm; predicted to contain a PINc domain |
|  | *YHL008C* | *AFUA_4G03435* | Putative protein of unknown function, may be involved in the uptake of chloride ions; does not appear to be involved in monocarboxylic acid transport; green fluorescent protein (GFP)-fusion protein localizes to the vacuole |
| *CDC19* | *YOR347C YAL038W* | *AFUA_6G07430* | Pyruvate kinase |
| *ENO1* | *YMR323W YOR393W YPL281C YGR254W YHR174W* | *AFUA_6G06770* | Enolase/allergen Asp F 22 |
|  | *YOR283W* | *AFUA_4G03580 AFUA_7G05820* | Phosphatase with some similarity to GPM1/YKL152C, a phosphoglycerate mutase; YOR283W is not an essential gene |
|  | *YKR043C* | *AFUA_6G02600 AFUA_6G08500* | Putative protein of unknown function; green fluorescent protein (GFP)-fusion protein localizes to the cytoplasm and nucleus |
|  | *YLR345W* | *AFUA_6G05100* | Similar to 6-phosphofructo-2-kinase/fructose-2,6-bisphosphatase enzymes responsible for the metabolism of fructoso-2,6-bisphosphate; mRNA expression is repressed by the Rfx1p-Tup1p-Ssn6p repressor complex; YLR345W is not an essential gene |
| *ACO2* | *YJL200C* | *AFUA_1G06810* | Aconitate hydratase, mitochondrial |
| *LSC1* | *YOR142W* | *AFUA_5G06130* | Alpha subunit of succinyl-CoA ligase, which is a mitochondrial enzyme of the TCA cycle that catalyzes the nucleotide-dependent conversion of succinyl-CoA to succinate; phosphorylated |
| *LSC2* | *YGR244C* | *AFUA_4G04520* | Beta subunit of succinyl-CoA ligase, which is a mitochondrial enzyme of the TCA cycle that catalyzes the nucleotide-dependent conversion of succinyl-CoA to succinate |
| *SDH1* | *YJL045W YKL148C* | *AFUA_3G07810* | Minor succinate dehydrogenase isozyme; homologous to Sdh1p, the major isozyme reponsible for the oxidation of succinate and transfer of electrons to ubiquinone; induced during the diauxic shift in a Cat8p-dependent manner |
| *SDH3* | *YMR118C YKL141W* | *AFUA_5G09680* | Cytochrome b subunit of succinate dehydrogenase (Sdh1p, Sdh2p, Sdh3p, Sdh4p), which couples the oxidation of succinate to the transfer of electrons to ubiquinone as part of the TCA cycle and the mitochondrial respiratory chain |
| *SDH4* | *YLR164W YDR178W* | *AFUA_1G15590* | Membrane anchor subunit of succinate dehydrogenase (Sdh1p, Sdh2p, Sdh3p, Sdh4p), which couples the oxidation of succinate to the transfer of electrons to ubiquinone as part of the TCA cycle and the mitochondrial respiratory chain |
| *KAP95* | *YLR347C* | *AFUA_1G15720* | Importin beta-1 subunit |
| *MSN5* | *YDR335W* | *AFUA_3G08780* | Karyopherin involved in nuclear import and export of proteins, including import of replication protein A and export of Swi6p, Far1p, and Pho4p; required for re-export of mature tRNAs after their retrograde import from the cytoplasm |
| *AGP2* | *YBR132C* | *AFUA_4G06230* | General amino acid permease (Agp2) |
| *FSF1* | *YOR271C* | *AFUA_1G16750* | Mitochondrial cation transporter |
| *JEN1* | *YKL217W* | *AFUA_7G05550 AFUA_8G00770* | Sugar transporter family protein |
| *MAL11* | *YGR289C YBR298C YDL247W YJR160C* | *AFUA_3G01700 AFUA_7G05190 AFUA_8G01340 AFUA_8G07240 AFUA_2G10910 AFUA_7G06390* | Alpha-glucoside permease, transports maltose, maltotriose, alpha-methylglucoside, and turanose; identical; encoded in a subtelomeric position in a region likely to have undergone duplication |
| *VPS73* | *YBR241C YGL104C* | *AFUA_3G13980* | Putative transporter, member of the sugar porter family; green fluorescent protein (GFP)-fusion protein localizes to the vacuolar membrane; YBR241C is not an essential gene |
|  | *YFL040W* | *AFUA_3G14170 AFUA_8G00890* | Putative transporter, member of the sugar porter family; YFL040W is not an essential gene |
| *MEP1* | *YGR121C YPR138C* | *AFUA_2G05880* | Ammonium permease of high capacity and low affinity; belongs to a ubiquitous family of cytoplasmic membrane proteins that transport only ammonium (NH4+); expression is under the nitrogen catabolite repression regulation ammonia permease |
| *TPN1* | *YGL186C* | *AFUA_2G03000 AFUA_1G10890 AFUA_6G07260* | Plasma membrane pyridoxine transporter; member of the purine-cytosine permease subfamily within the major facilitator superfamily; proton symporter with similarity to Fcy21p, Fcy2p, and Fcy22p |
| *AGC1* | *YPR021C* | *AFUA_7G05220* | Mitochondrial carrier protein |
| *APL2* | *YKL135C* | *AFUA_2G10340 AFUA_3G08970* | AP-2 adaptor complex subunit beta |
| *APL3* | *YBL037W* | *AFUA_4G04310* | Alpha-adaptin, large subunit of the clathrin associated protein complex (AP-2); involved in vesicle mediated transport |
| *APL4* | *YPR029C* | *AFUA_1G06030* | AP-1 adaptor complex subunit gamma |
| *APL5* | *YPL195W* | *AFUA_7G03640* | AP-3 complex subunit delta |
| *APM1* | *YPL259C* | *AFUA_5G09660* | AP-1 adaptor complex subunit mu |
| *APM4* | *YOL062C* | *AFUA_5G07930* | AP-2 adaptor complex subunit mu |
| *APS1* | *YLR170C* | *AFUA_2G01570* | AP-1 adaptor complex subunit sigma |
| *APS2* | *YJR058C* | *AFUA_1G14010* | AP-2 adaptor complex subunit sigma |
| *APS3* | *YJL024C* | *AFUA_6G13020* | AP-3 adaptor complex subunit sigma |
| *AQY1* | *YLL053C YPR192W* | *AFUA_4G03390* | Putative protein; in the Sigma 1278B strain background YLL053C is contiguous with AQY2 which encodes an aquaporin |
| *ARB1* | *YER036C* | *AFUA_5G07020* | ATPase of the ATP-binding cassette family involved in 40S and 60S ribosome biogenesis, has similarity to Gcn20p; shuttles from nucleus to cytoplasm, physically interacts with Tif6p, Lsg1p |
| *ARF1* | *YDL192W YDL137W* | *AFUA_1G11730* | ADP-ribosylation factor, GTPase of the Ras superfamily involved in regulation of coated formation vesicles in intracellular trafficking within the Golgi; functionally interchangeable with Arf1p |
| *ARL3* | *YPL051W* | *AFUA_1G16970* | ADP-ribosylation factor family protein |
| *ATG13* | *YPR185W* | *AFUA_2G04770* | Autophagy protein Atg13 |
| *ATG22* | *YCL038C* | *AFUA_2G06170 AFUA_2G15370* | MFS transporter family protein |
| *ATG3* | *YNR007C* | *AFUA_5G08170* | Autophagocytosis protein Aut1 |
| *AVT1* | *YJR001W* | *AFUA_5G05670* | Transporter |
| *AVT2* | *YEL064C* | *AFUA_4G06990* | Amino acid transporter |
| *AVT3* | *YKL146W* | *AFUA_2G00310 AFUA_4G07760* | Amino acid transporter |
| *AVT5* | *YBL089W YER119C* | *AFUA_5G09300* | Amino acid transporter |
| *BET1* | *YIL004C* | *AFUA_1G07420* | SNARE complex subunit (Bet1) |
| *BFR2* | *YDR299W* | *AFUA_6G09820* | Vesicle-mediated transport protein Bfr2/Che-1 |
| *BOS1* | *YLR078C* | *AFUA_7G05735* | V-SNARE protein Bos1 |
| *BPH1* | *YCR032W* | *AFUA_5G09220* | Beige/BEACH domain protein |
| *COP1* | *YDL145C* | *AFUA_3G08840* | Alpha subunit of COPI vesicle coatomer complex, which surrounds transport vesicles in the early secretory pathway |
| *CRC1* | *YOR100C* | *AFUA_6G14100* | Mitochondrial carnitine:acyl carnitine carrier, putative |
| *DNF1* | *YER166W YDR093W* | *AFUA_6G02070* | Aminophospholipid translocase that localizes primarily to the plasma membrane; contributes to endocytosis, protein transport and cell polarity; type 4 P-type ATPase |
| *DNF3* | *YMR162C* | *AFUA_4G10210* | Aminophospholipid translocase that maintains membrane lipid asymmetry in post-Golgi secretory vesicles; localizes to the trans-Golgi network; likely involved in protein transport; type 4 P-type ATPase |
| *DRS2* | *YAL026C* | *AFUA_2G08850* | Aminophospholipid translocase that maintains membrane lipid asymmetry in post-Golgi secretory vesicles; contributes to clathrin-coated vesicle formation and endocytosis; mutations in human homolog ATP8B1 result in liver disease |
| *EMP24* | *YGL200C* | *AFUA_1G11470* | Endosomal cargo receptor (P24) |
| *ERV25* | *YML012W* | *AFUA_4G13190* | Endosomal cargo receptor (Erv25) |
| *FCY2* | *YER060W YER056C YER060W-A* | *AFUA_2G09860* | Purine-cytosine permease |
| *FEN2* | *YCR028C* | *AFUA_4G00920* | MFS pantothenate transporter |
| *GYP1* | *YOR070C* | *AFUA_2G06080* | Cis-golgi GTPase-activating protein for the Rab family members Ypt1p and for Ypt1p, Sec4p, Ypt7p, and Ypt51p (in vitro); involved in vesicle docking and fusion |
| *HEF3* | *YLR249W YNL014W* | *AFUA_7G05660* | Translational elongation factor 3, stimulates the binding of aminoacyl-tRNA to ribosomes by releasing EF-1 alpha from the ribosomal complex; contains two ABC cassettes; binds and hydrolyses ATP |
| *LEU5* | *YHR002W* | *AFUA_2G16770* | Mitochondrial carrier protein (Leu5) |
| *LST8* | *YNL006W* | *AFUA_1G09560* | Protein transport protein (LST8) |
| *MCH2* | *YKL221W* | *AFUA_4G12260 AFUA_8G06610* | MFS transporter (Mch2) |
| *MCH4* | *YOL119C YOR306C* | *AFUA_2G12790 AFUA_2G03520 AFUA_7G00710* | MFS monocarboxylate transporter |
| *MDL1* | *YLR188W* | *AFUA_4G10000* | ABC multidrug transporter Mdr2 |
| *MTM1* | *YGR257C* | *AFUA_2G09250* | Mitochondrial protein of the mitochondrial carrier family, involved in activating mitochondrial Sod2p probably by facilitating insertion of an essential manganese cofactor |
| *NCE102* | *YGR131W YPR149W* | *AFUA_2G01590* | Protein of unknown function; expression induced in response to ketoconazole; promoter region contains a sterol regulatory element motif, which has been identified as a Upc2p-binding site |
| *NCE103* | *YNL036W* | *AFUA_4G11250 AFUA_8G06550* | Carbonic anhydrase; poorly transcribed under aerobic conditions and at an undetectable level under anaerobic conditions; involved in non-classical protein export pathway |
| *NEO1* | *YIL048W* | *AFUA_6G03950* | Phospholipid-translocating P-type ATPase |
| *NEW1* | *YPL226W* | *AFUA_6G03580* | ATP binding cassette protein that cosediments with polysomes and is required for biogenesis of the small ribosomal subunit; Asn/Gln-rich rich region supports [NU+] prion formation and susceptibility to [PSI+] prion induction |
| *NIP100* | *YPL174C* | *AFUA_2G13450* | Large subunit of the dynactin complex, which is involved in partitioning the mitotic spindle between mother and daughter cells; putative ortholog of mammalian p150(glued) |
| *NPL4* | *YBR170C* | *AFUA_1G02830* | Endoplasmic reticulum and nuclear membrane protein, forms a complex with Cdc48p and Ufd1p that recognizes ubiquitinated proteins in the endoplasmic reticulum and delivers them to the proteasome for degradation |
| *NUG1* | *YER006W* | *AFUA_1G10560* | GTPase that associates with nuclear 60S pre-ribosomes, required for export of 60S ribosomal subunits from the nucleus |
| *ODC1* | *YOR222W YPL134C* | *AFUA_1G09660* | Mitochondrial 2-oxodicarboxylate carrier protein, putative |
| *PDR10* | *YOR328W YOR153W YDR406W* | *AFUA_3G07300 AFUA_5G02260 AFUA_5G00790 AFUA_2G15130 AFUA_1G14330 AFUA_1G17440* | ABC multidrug transporter |
| *PEP1* | *YNR065C YBL017C YIL173W YJL222W* | *AFUA_8G02780* | Putative membrane glycoprotein with strong similarity to Vth2p and Pep1p/Vps10p, may be involved in vacuolar protein sorting |
| *PET8* | *YNL003C* | *AFUA_5G11850* | Mitochondrial carrier protein (Pet8) |
| *PEX3* | *YDR329C* | *AFUA_5G06300* | Peroxisomal membrane protein required for proper localization and stability of PMPs; anchors peroxisome retention factor Inp1p at the peroxisomal membrane; interacts with Pex19p |
| *PHO88* | *YBR106W* | *AFUA_5G01960* | Phosphate transporter (Pho88) |
| *PKR1* | *YMR123W* | *AFUA_2G05200* | ER membrane protein (Pkr1) |
| *RET2* | *YFR051C* | *AFUA_1G15860* | Delta subunit of the coatomer complex (COPI), which coats Golgi-derived transport vesicles; involved in retrograde transport between Golgi and ER |
| *RET3* | *YPL010W* | *AFUA_2G09180* | Coatomer subunit zeta |
| *RLI1* | *YDR091C* | *AFUA_1G10310* | Essential iron-sulfur protein required for ribosome biogenesis and translation initiation; facilitates binding of a multifactor complex of translation initiation factors to the small ribosomal subunit; predicted ABC family ATPase |
| *RPG1* | *YBR079C* | *AFUA_1G05200* | Eukaryotic translation initiation factor 3 subunit EifCa |
| *RSE1* | *YML049C* | *AFUA_6G13410* | Protein involved in pre-mRNA splicing; component of the pre-spliceosome; associates with U2 snRNA; involved in ER to Golgi transport |
| *SAL1* | *YNL083W* | *AFUA_2G15780* | ADP/ATP transporter; member of the Ca2+-binding subfamily of mitochondrial carriers, with two EF-hand motifs; transport activity of either Sal1p or Pet9p is critical for viability; polymorphic in different S. cerevisiae strains |
| *SAR1* | *YPL218W* | *AFUA_1G04940* | GTPase, GTP-binding protein of the ARF family, component of COPII coat of vesicles; required for transport vesicle formation during ER to Golgi protein transport |
| *SCD5* | *YOR329C* | *AFUA_4G09120* | Protein required for normal actin organization and endocytosis; targeting subunit for protein phosphatase type 1; undergoes Crm1p-dependent nuclear-cytoplasmic shuttling; multicopy suppressor of clathrin deficiency |
| *SCD6* | *YPR129W* | *AFUA_1G12390* | Protein containing an Lsm domain, may bind RNA and have a role in RNA processing; overproduction suppresses a null mutation in CHC1, which encodes the heavy chain of clathrin |
| *SCT1* | *YBL011W* | *AFUA_4G11360* | Glycerol 3-phosphate/dihydroxyacetone phosphate dual substrate-specific sn-1 acyltransferase of the glycerolipid biosynthesis pathway, prefers 16-carbon fatty acids, similar to Gpt2p, gene is constitutively transcribed |
| *SEC13* | *YLR208W* | *AFUA_4G06090* | Component of both the Nup84 nuclear pore sub-complex and the Sec13p-Sec31p complex of the COPII vesicle coat, required for vesicle formation in ER to Golgi transport and nuclear pore complex organization |
| *SEC16* | *YPL085W* | *AFUA_6G03960* | COPII vesicle coat protein required for ER transport vesicle budding; Sec16p is bound to the periphery of ER membranes and may act to stabilize initial COPII complexes; interacts with Sec23p, Sec24p and Sec31p |
| *SEC17* | *YBL050W* | *AFUA_2G12870* | Peripheral membrane protein required for vesicular transport between ER and Golgi and for the 'priming' step in homotypic vacuole fusion, part of the cis-SNARE complex; has similarity to alpha-SNAP |
| *SEC18* | *YBR080C* | *AFUA_3G12510* | ATPase required for the release of Sec17p during the 'priming' step in homotypic vacuole fusion and for ER to Golgi transport; homolog of the mammalian NSF |
| *SEC2* | *YNL272C* | *AFUA_3G06430* | Guanyl-nucleotide exchange factor for the small G-protein Sec4p; essential for post-Golgi vesicle transport; associates with the exocyst, via exocyst subunit Sec15p, on secretory vesicles |
| *SEC21* | *YNL287W* | *AFUA_2G02560* | Gamma subunit of coatomer, a heptameric protein complex that together with Arf1p forms the COPI coat; involved in ER to Golgi transport of selective cargo |
| *SEC22* | *YLR268W* | *AFUA_6G04150* | R-SNARE protein; assembles into SNARE complex with Bet1p, Bos1p and Sed5p; cycles between the ER and Golgi complex; involved in anterograde and retrograde transport between the ER and Golgi; synaptobrevin homolog |
| *SEC23* | *YPR181C* | *AFUA_1G03400* | GTPase-activating protein, stimulates the GTPase activity of Sar1p; component of the Sec23p-Sec24p heterodimer of the COPII vesicle coat, involved in ER to Golgi transport |
| *SEC24* | *YIL109C YNL049C* | *AFUA_6G12830* | Component of the Sec23p-Sec24p heterodimer of the COPII vesicle coat, required for cargo selection during vesicle formation in ER to Golgi transport; homologous to Sfb2p and Sfb3p |
| *SEC26* | *YDR238C* | *AFUA_1G10970* | Essential beta-coat protein of the COPI coatomer, involved in ER-to-Golgi protein trafficking and maintenance of normal ER morphology; shares 43% sequence identity with mammalian beta-coat protein (beta-COP) |
| *SEC27* | *YGL137W* | *AFUA_2G10610* | Essential beta'-coat protein of the COPI coatomer, involved in ER-to-Golgi and Golgi-to-ER transport; contains WD40 domains that mediate cargo selective interactions; 45% sequence identity to mammalian beta'-COP |
| *SEC31* | *YDL195W* | *AFUA_2G12980* | Component of the Sec13p-Sec31p complex of the COPII vesicle coat, required for vesicle formation in ER to Golgi transport |
| *SEC4* | *YFL005W* | *AFUA_4G04810* | Rab GTPase SrgA |
| *SEC7* | *YDR170C* | *AFUA_7G05700* | Guanine nucleotide exchange factor for ADP ribosylation factors involved in proliferation of the Golgi, intra-Golgi transport and ER-to-Golgi transport; found in the cytoplasm and on Golgi-associated coated vesicles |
| *SEO1* | *YGR065C YAL067C* | *AFUA_6G01930* | High-affinity plasma membrane H+-biotin symporter; mutation results in fatty acid auxotrophy; 12 transmembrane domain containing major facilitator subfamily member; mRNA levels negatively regulated by iron deprivation and biotin |
| *SHE4* | *YOR035C* | *AFUA_5G11600* | Protein containing a UCS domain, binds to myosin motor domains to regulate myosin function; involved in endocytosis, polarization of the actin cytoskeleton, and asymmetric mRNA localization |
| *SLY41* | *YOR307C* | *AFUA_5G04360* | Protein involved in ER-to-Golgi transport |
| *SNC1* | *YOR327C YAL030W* | *AFUA_6G02920* | SNARE protein Snc2 |
| *STP22* | *YCL008C* | *AFUA_3G14380* | Component of the ESCRT-I complex, which is involved in ubiquitin-dependent sorting of proteins into the endosome; homologous to the mouse and human Tsg101 tumor susceptibility gene; mutants exhibit a Class E Vps phenotype |
| *SYS1* | *YJL004C* | *AFUA_3G09790* | Integral membrane protein of the Golgi required for targeting of the Arf-like GTPase Arl3p to the Golgi; multicopy suppressor of ypt6 null mutation |
| *THI73* | *YLR004C* | *AFUA_6G00630* | Putative plasma membrane permease proposed to be involved in carboxylic acid uptake and repressed by thiamine; substrate of Dbf2p/Mob1p kinase; transcription is altered if mitochondrial dysfunction occurs |
| *TLG2* | *YOL018C* | *AFUA_4G10040* | Syntaxin-like t-SNARE that forms a complex with Tlg1p and Vti1p and mediates fusion of endosome-derived vesicles with the late Golgi; binds Vps45p, which prevents Tlg2p degradation and also facilitates t-SNARE complex formation |
| *TMA64* | *YDR117C* | *AFUA_1G12570* | Protein of unknown function that associates with ribosomes; has a putative RNA binding domain |
| *TNA1* | *YGR260W* | *AFUA_5G01680 AFUA_6G00710 AFUA_5G06290 AFUA_1G11820 AFUA_7G06120 AFUA_6G02730 AFUA_6G11320 AFUA_1G01812 AFUA_3G03820* | MFS transporter |
| *TPC1* | *YGR096W* | *AFUA_2G14980* | Mitochondrial membrane transporter that mediates uptake of the essential cofactor thiamine pyrophosphate into mitochondria; expression appears to be regulated by carbon source; member of the mitochondrial carrier family |
| *VID24* | *YBR105C YGR066C* | *AFUA_1G10950* | Peripheral membrane protein located at Vid vesicles; regulates fructose-1,6-bisphosphatase targeting to the vacuole; promotes proteasome-dependent catabolite degradation of FBPase |
| *VMR1* | *YHL035C YLL048C* | *AFUA_5G09190* | Protein of unknown function that may interact with ribosomes, based on co-purification experiments; member of the ATP-binding cassette family; potential Cdc28p substrate; detected in purified mitochondria in high-throughput studies |
| *VPS15* | *YBR097W* | *AFUA_6G11190* | Myristoylated serine/threonine protein kinase involved in vacuolar protein sorting; functions as a membrane-associated complex with Vps34p; active form recruits Vps34p to the Golgi membrane; interacts with the GDP-bound form of Gpa1p |
| *VPS21* | *YOR089C YNL093W* | *AFUA_3G10740* | GTPase required for transport during endocytosis and for correct sorting of vacuolar hydrolases; localized in endocytic intermediates; detected in mitochondria; geranylgeranylation required for membrane association; mammalian Rab5 homolog |
| *VPS30* | *YPL120W* | *AFUA_8G05170* | Subunit of phosphatidylinositol 3-kinase complexes I and II; Complex I is essential in autophagy and Complex II is required for vacuolar protein sorting; ortholog of the higher eukaryotic gene Beclin 1 |
| *VPS34* | *YLR240W* | *AFUA_5G08670* | Phosphatidylinositol 3-kinase responsible for the synthesis of phosphatidylinositol 3-phosphate; forms membrane-associated signal transduction complex with Vps15p to regulate protein sorting; activated by the GTP-bound form of Gpa1p |
| *VPS8* | *YAL002W* | *AFUA_1G05280* | Membrane-associated protein that interacts with Vps21p to facilitate soluble vacuolar protein localization; component of the CORVET complex; required for localization and trafficking of the CPY sorting receptor; contains RING finger motif |
| *VRP1* | *YLR337C* | *AFUA_1G11690* | Proline-rich, actin-associated protein Vrp1 |
| *VTI1* | *YMR197C* | *AFUA_4G10710* | Protein involved in cis-Golgi membrane traffic; v-SNARE that interacts with two t-SNARES, Sed5p and Pep12p; required for multiple vacuolar sorting pathways |
| *YCT1* | *YLL055W* | *AFUA_7G06790* | High-affinity cysteine-specific transporter with similarity to the Dal5p family of transporters; green fluorescent protein (GFP)-fusion protein localizes to the endoplasmic reticulum; YCT1 is not an essential gene |
| *YEA6* | *YEL006W YIL006W* | *AFUA_4G06780* | Mitochondrial NAD+ transporter, involved in the transport of NAD+ into the mitochondria (see also YEA6); member of the mitochondrial carrier subfamily; disputed role as a pyruvate transporter; has putative mouse and human orthologs |
| *YOP1* | *YPR028W* | *AFUA_5G06320* | Membrane protein that interacts with Yip1p to mediate membrane traffic; interacts with Sey1p to maintain ER morphology; overexpression leads to cell death and accumulation of internal cell membranes |
| *YPK9* | *YOR291W* | *AFUA_5G05520* | Vacuolar protein with a possible role in sequestering heavy metals; has similarity to the type V P-type ATPase Spf1p; homolog of human ATP13A2 (PARK9), mutations in which are associated with Parkinson disease and Kufor-Rakeb syndrome |
| *YPT31* | *YER031C YGL210W* | *AFUA_1G02190* | GTPase of the Ypt/Rab family, very similar to Ypt32p; involved in the exocytic pathway; mediates intra-Golgi traffic or the budding of post-Golgi vesicles from the trans-Golgi |
| *YPT7* | *YML001W* | *AFUA_5G12130* | GTPase; GTP-binding protein of the rab family; required for homotypic fusion event in vacuole inheritance, for endosome-endosome fusion, similar to mammalian Rab7 |
|  | *YDL119C* | *AFUA_4G12340* | Putative mitochondrial transport protein; GFP-fusion protein is induced in response to the DNA-damaging agent MMS; the authentic, non-tagged protein is detected in purified mitochondria |
|  | *YPR011C* | *AFUA_2G07400* | Putative transporter, member of the mitochondrial carrier family; the authentic, non-tagged protein is detected in highly purified mitochondria in high-throughput studies |
|  | *YMR166C* | *AFUA_7G05390* | Predicted transporter of the mitochondrial inner membrane; has similarity to human mitochondrial ATP-Mg/Pi carriers; YMR166C is not an essential gene |
|  | *YIL166C* | *AFUA_3G07950 AFUA_1G13350 AFUA_4G00120* | Putative protein with similarity to the allantoate permease subfamily of the major facilitator superfamily; mRNA expression is elevated by sulfur limitation; YIL166C is a non-essential gene |
|  | *YOL075C* | *AFUA_3G09680* | ABC efflux transporter |
|  | *YJR124C* | *AFUA_3G04000* | Putative protein of unknown function; expression induced under calcium shortage |
|  | *YFL054C* | *AFUA_6G08480* | Putative channel-like protein; similar to Fps1p; mediates passive diffusion of glycerol in the presence of ethanol |
|  | *YDL206W* | *AFUA_7G03880* | Putative protein of unknown function; YDL206W is not an essential protein |
| *APN2* | *YBL019W* | *AFUA_3G06180* | DNA lyase Apn2 |
| *DIN7* | *YOR033C YDR263C* | *AFUA_3G09050* | 5'-3' exonuclease and flap-endonuclease involved in recombination, double-strand break repair and DNA mismatch repair; member of the Rad2p nuclease family, with conserved N and I nuclease domains |
| *EBS1* | *YLR233C YDR206W* | *AFUA_5G06400* | Ebs1p |
| *HSP12* | *YFL014W* | *AFUA_1G17370 AFUA_6G12450* | Chaperone/heat shock protein Hsp12 |
| *IRC5* | *YFR038W* | *AFUA_1G13010* | SNF2 family helicase/ATPase PasG |
| *MEI5* | *YPL121C* | *AFUA_1G14695* | DNA repair protein Dds20/Mei5 |
| *MLH3* | *YPL164C* | *AFUA_4G06490* | DNA mismatch repair protein (Mlh3) |
| *MMS2* | *YGL087C* | *AFUA_4G12080* | Ubiquitin conjugating enzyme (MmsB) |
| *MMS21* | *YEL019C* | *AFUA_6G07700* | Chromosomal organization and DNA repair protein Mms21, putative |
| *MMS4* | *YBR098W* | *AFUA_5G13220* | Mms4p |
| *NAM8* | *YHR086W* | *AFUA_7G02230* | MRNA binding post-transcriptional regulator (Csx1), putative |
| *NTG1* | *YOL043C YAL015C* | *AFUA_2G01120* | DNA N-glycosylase and apurinic/apyrimidinic lyase involved in base excision repair; acts in both nucleus and mitochondrion; creates a double-strand break at mtDNA origins that stimulates replication in response to oxidative stress |
| *POL31* | *YJR006W* | *AFUA_4G07970* | DNA polymerase III subunit, essential for cell viability; involved in DNA replication and DNA repair |
| *RAD17* | *YOR368W* | *AFUA_4G12670* | Checkpoint protein, involved in the activation of the DNA damage and meiotic pachytene checkpoints; with Mec3p and Ddc1p, forms a clamp that is loaded onto partial duplex DNA; homolog of human and S. pombe Rad1 and U. maydis Rec1 proteins |
| *RAD30* | *YDR419W* | *AFUA_5G09000* | DNA polymerase eta, involved in tranlesion synthesis during post-replication repair; catalyzes the synthesis of DNA opposite cyclobutane pyrimidine dimers and other lesions; mutations in human pol eta are responsible for XPV |
| *RDH54* | *YBR073W* | *AFUA_1G15120* | DNA-dependent ATPase, stimulates strand exchange by modifying the topology of double-stranded DNA; involved in recombinational repair of DNA double-strand breaks during mitosis and meiosis; proposed to be involved in crossover interference |
| *REV1* | *YOR346W* | *AFUA_6G08570* | Deoxycytidyl transferase, forms a complex with the subunits of DNA polymerase zeta, Rev3p and Rev7p; involved in repair of abasic sites in damaged DNA |
| *REV7* | *YIL139C* | *AFUA_2G15880* | Accessory subunit of DNA polymerase zeta, involved in translesion synthesis during post-replication repair; required for mutagenesis induced by DNA damage; involved in double-strand break repair |
| *RNR1* | *YER070W YIL066C* | *AFUA_4G06690* | One of two large regulatory subunits of ribonucleotide-diphosphate reductase; the RNR complex catalyzes rate-limiting step in dNTP synthesis, regulated by DNA replication and DNA damage checkpoint pathways via localization of small subunits |
| *RPT1* | *YKL145W* | *AFUA_3G07820* | One of six ATPases of the 19S regulatory particle of the 26S proteasome involved in the degradation of ubiquitinated substrates; required for optimal CDC20 transcription; interacts with Rpn12p and the E3 ubiquitin-protein ligase Ubr1p |
| *RRD1* | *YIL153W* | *AFUA_6G08680* | Peptidyl-prolyl cis/trans-isomerase, activator of the phosphotyrosyl phosphatase activity of PP2A; involved in G1 phase progression, microtubule dynamics, bud morphogenesis and DNA repair; subunit of the Tap42p-Sit4p-Rrd1p complex |
| *RTS2* | *YOR077W* | *AFUA_3G02320* | Basic zinc-finger protein, similar to human and mouse Kin17 proteins which are chromatin-associated proteins involved in UV response and DNA replication |
| *SMC5* | *YOL034W* | *AFUA_6G02700* | Structural maintenance of chromosome complex subunit SmcA |
| *SMC6* | *YLR383W* | *AFUA_3G05440* | Protein involved in structural maintenance of chromosomes; essential subunit of Mms21-Smc5-Smc6 complex; required for growth, DNA repair, interchromosomal and sister chromatid recombination; homologous to S. pombe rad18 |
| *SSL1* | *YLR005W* | *AFUA_5G04333* | Component of the core form of RNA polymerase transcription factor TFIIH, which has both protein kinase and DNA-dependent ATPase/helicase activities and is essential for transcription and nucleotide excision repair; interacts with Tfb4p |
| *TFB3* | *YDR460W* | *AFUA_4G08900* | Subunit of TFIIH and nucleotide excision repair factor 3 complexes, involved in transcription initiation, required for nucleotide excision repair; ring finger protein similar to mammalian CAK and TFIIH subunit |
| *THI4* | *YGR144W* | *AFUA_6G08360* | Thiazole biosynthesis enzyme |
|  | *YDR061W* | *AFUA_6G11360* | Protein with similarity to ATP-binding cassette transporter family members; lacks predicted membrane-spanning regions; transcriptionally activated by Yrm1p along with genes involved in multidrug resistance |
| *ADR1* | *YDR216W YML081W YJR127C* | *AFUA_2G17220* | Putative protein of unknown function; green fluorescent protein (GFP)-fusion protein localizes to the nucleus; YML081w is not an essential gene |
| *CEF1* | *YMR213W* | *AFUA_2G05540* | Cell division control protein (Cdc5) |
| *CUP2* | *YGL166W YPR008W* | *AFUA_6G07780* | Copper-binding transcription factor; activates transcription of the metallothionein genes CUP1-1 and CUP1-2 in response to elevated copper concentrations |
| *CUS2* | *YNL286W* | *AFUA_1G10920* | Nuclear mRNA splicing factor-associated protein, putative |
| *DBP10* | *YDL031W* | *AFUA_6G11120* | ATP dependent RNA helicase (Dbp10) |
| *DBP7* | *YKR024C* | *AFUA_5G11050* | ATP dependent RNA helicase (Dbp7) |
| *DBP8* | *YHR169W* | *AFUA_3G10890* | ATPase, putative RNA helicase of the DEAD-box family; component of 90S preribosome complex involved in production of 18S rRNA and assembly of 40S small ribosomal subunit; ATPase activity stimulated by association with Esp2p |
| *DHR2* | *YKL078W* | *AFUA_2G09230* | ATP-dependent RNA helicase (Hrh1) |
| *GBP2* | *YCL011C YNL004W* | *AFUA_6G12300* | RNP domain protein |
| *GIS2* | *YNL255C* | *AFUA_1G07630* | Zinc knuckle domain protein (Byr3) |
| *IRS4* | *YJL083W YKR019C* | *AFUA_5G12070* | EH domain-containing protein involved in regulating phosphatidylinositol 4,5-bisphosphate levels and autophagy; Irs4p and Tax4p bind and activate the PtdIns phosphatase Inp51p; Irs4p and Tax4p are involved in localizing Atg17p to the PAS |
| *LEA1* | *YPL213W* | *AFUA_5G11000* | Component of U2 snRNP; disruption causes reduced U2 snRNP levels; physically interacts with Msl1p; putative homolog of human U2A' snRNP protein |
| *LHP1* | *YDL051W* | *AFUA_1G12590* | RNA-binding La domain protein |
| *LSM7* | *YNL147W* | *AFUA_1G14290* | Lsm protein; part of heteroheptameric complexes (Lsm2p-7p and either Lsm1p or 8p): cytoplasmic Lsm1p complex involved in mRNA decay; nuclear Lsm8p complex part of U6 snRNP and possibly involved in processing tRNA, snoRNA, and rRNA |
| *MSL1* | *YIR009W* | *AFUA_5G11930 AFUA_6G06490* | U1 small nuclear ribonucleoprotein A |
| *MXR2* | *YCL033C* | *AFUA_6G07840* | Methionine-R-sulfoxide reductase, involved in the response to oxidative stress; protects iron-sulfur clusters from oxidative inactivation along with MXR1; involved in the regulation of lifespan |
| *NMD3* | *YHR170W* | *AFUA_2G16750* | Nonsense-mediated mRNA decay protein 3 |
| *NOP13* | *YNL175C* | *AFUA_3G05840* | RNA binding protein Rnp24 |
| *NTO1* | *YPR031W* | *AFUA_7G05250* | PHD finger domain protein |
| *OLI1* | *Q0130* | *AFUA_4G09360* | F0-ATP synthase subunit c (ATPase-associated proteolipid), encoded on the mitochondrial genome; mutation confers oligomycin resistance; expression is specifically dependent on the nuclear genes AEP1 and AEP2 |
| *PBP2* | *YBR233W* | *AFUA_2G04940* | KH domain RNA binding protein |
| *PST2* | *YCR004C YDR032C* | *AFUA_1G02820* | Protein with similarity to members of a family of flavodoxin-like proteins; induced by oxidative stress in a Yap1p dependent manner; the authentic, non-tagged protein is detected in highly purified mitochondria in high-throughput studies |
| *PUF4* | *YGL014W* | *AFUA_1G02510* | Member of the PUF protein family, which is defined by the presence of Pumilio homology domains that confer RNA binding activity; preferentially binds mRNAs encoding nucleolar ribosomal RNA-processing factors |
| *RCL1* | *YOL010W* | *AFUA_8G04640* | RNA-3'-phosphate cyclase family protein |
| *REX2* | *YLR059C* | *AFUA_3G11820* | 3'-5' RNA exonuclease; involved in 3'-end processing of U4 and U5 snRNAs, 5S and 5.8S rRNAs, and RNase P and RNase MRP RNA; localized to mitochondria and null suppresses escape of mtDNA to nucleus in yme1 yme2 mutants; RNase D exonuclease |
| *RGM1* | *YMR182C YPL230W* | *AFUA_3G09820* | Putative transcription factor containing a C2H2 zinc finger; mutation affects transcriptional regulation of genes involved in growth on non-fermentable carbon sources, response to salt stress and cell wall biosynthesis |
| *ROK1* | *YGL171W* | *AFUA_1G16290* | ATP-dependent RNA helicase of the DEAD box family; required for 18S rRNA synthesis |
| *RRP1* | *YDR087C* | *AFUA_1G10990* | Essential evolutionarily conserved nucleolar protein necessary for biogenesis of 60S ribosomal subunits and processing of pre-rRNAs to mature rRNAs, associated with several distinct 66S pre-ribosomal particles |
| *RRP42* | *YDL111C* | *AFUA_2G13130* | Exosome non-catalytic core component; involved in 3'-5' RNA processing and degradation in both the nucleus and the cytoplasm; has similarity to E. coli RNase PH and to human hRrp42p (EXOSC7) |
| *RRP7* | *YCL031C* | *AFUA_7G02210* | Essential protein involved in rRNA processing and ribosome biogenesis |
| *SAK1* | *YER129W YGL179C* | *AFUA_5G05980* | Protein kinase, related to and functionally redundant with Elm1p and Sak1p for the phosphorylation and activation of Snf1p; functionally orthologous to LKB1, a mammalian kinase associated with Peutz-Jeghers cancer-susceptibility syndrome |
| *SET2* | *YJL168C* | *AFUA_5G06000* | SET and WW domain protein |
| *SGN1* | *YIR001C* | *AFUA_1G09490* | Cytoplasmic RNA-binding protein, contains an RNA recognition motif (RRM); may have a role in mRNA translation, as suggested by genetic interactions with genes encoding proteins involved in translational initiation |
| *SLM1* | *YIL105C YNL047C* | *AFUA_6G12290* | Phosphoinositide PI4,5P(2) binding protein, forms a complex with Slm2p; acts downstream of Mss4p in a pathway regulating actin cytoskeleton organization in response to stress; phosphorylated by the TORC2 complex |
| *SOL1* | *YCR073W-A YNR034W* | *AFUA_1G02980* | Protein with a possible role in tRNA export; shows similarity to 6-phosphogluconolactonase non-catalytic domains but does not exhibit this enzymatic activity; homologous to Sol1p, Sol3p, and Sol4p |
| *SPP2* | *YOR148C* | *AFUA_4G07550* | Essential protein that promotes the first step of splicing and is required for the final stages of spliceosome maturation; interacts with Prp2p, which may release Spp2p from the spliceosome following the first cleavage reaction |
| *SSM4* | *YIL030C* | *AFUA_2G08650* | RING finger membrane protein |
| *SUB2* | *YDL084W* | *AFUA_6G02630* | Component of the TREX complex required for nuclear mRNA export; member of the DEAD-box RNA helicase superfamily and is involved in early and late steps of spliceosome assembly; homolog of the human splicing factor hUAP56 |
| *TPD3* | *YAL016W* | *AFUA_1G05610* | Regulatory subunit A of the heterotrimeric protein phosphatase 2A (PP2A), which also contains regulatory subunit Cdc55p and either catalytic subunit Pph21p or Pph22p; required for cell morphogenesis and transcription by RNA polymerase III |
| *TPT1* | *YOL102C* | *AFUA_2G02890* | TRNA splicing 2' phosphotransferase 1 |
| *YHP1* | *YML027W YDR451C* | *AFUA_3G12160* | Homeodomain-containing transcriptional repressor, binds to Mcm1p and to early cell cycle boxes in the promoters of cell cycle-regulated genes expressed in M/G1 phase; expression is cell cycle-regulated; potential Cdc28p substrate |
| *YLH47* | *YPR125W* | *AFUA_3G08230* | Mitochondrial inner membrane protein exposed to the mitochondrial matrix, associates with mitochondrial ribosomes, NOT required for respiratory growth; homolog of human Letm1, a protein implicated in Wolf-Hirschhorn syndrome |
|  | *YPR013C YPR015C* | *AFUA_2G13770 AFUA_6G02690* | Putative protein of unknown function; overexpression causes a cell cycle delay or arrest |
| *ESS1* | *YJR017C* | *AFUA_2G08550* | Peptidyl-prolyl cis/trans isomerase |
| *LSM1* | *YJL124C* | *AFUA_2G11800* | Lsm protein; forms heteroheptameric complex involved in degradation of cytoplasmic mRNAs |
| *PTA1* | *YAL043C* | *AFUA_8G04420* | MRNA cleavage and polyadenylation specificity factor complex subunit (Pta1) |
| *YTH1* | *YPR107C* | *AFUA_1G02810* | Essential RNA-binding component of cleavage and polyadenylation factor, contains five zinc fingers; required for pre-mRNA 3'-end processing and polyadenylation |
| *ASF1* | *YJL115W* | *AFUA_3G11030* | Histone chaperone ASF1 |
| *ASG1* | *YIL130W* | *AFUA_3G11990* | C6 transcription factor (Mut3) |
| *BDF1* | *YLR399C YDL070W* | *AFUA_4G10540* | Transcription regulator BDF1 |
| *BTT1* | *YPL037C YDR252W* | *AFUA_6G02750* | Beta3 subunit of the heterotrimeric nascent polypeptide-associated complex which binds ribosomes via its beta-subunits in close proximity to nascent polypeptides; interacts with Caf130p of the CCR4-NOT complex; similar to human BTF3 |
| *CAF16* | *YFL028C* | *AFUA_6G05080* | ABC transporter |
| *CAF4* | *YJL112W YKR036C* | *AFUA_5G13140* | WD repeat-containing protein |
| *CHD1* | *YER164W* | *AFUA_1G10290* | Chromodomain helicase (Chd1) |
| *DOT5* | *YIL010W* | *AFUA_4G05915* | Disrupter of telomere silencing protein Dot5 |
| *ECM22* | *YDR034C YLR228C YDR213W* | *AFUA_2G10850 AFUA_5G12930* | Sterol regulatory element binding protein, induces transcription of sterol biosynthetic genes and of DAN/TIR gene products; Ecm22p homolog; relocates from intracellular membranes to perinuclear foci on sterol depletion |
| *ECM22* | *YLR228C YDR213W* | *AFUA_2G10830 AFUA_3G01040 AFUA_8G05750 AFUA_8G00980 AFUA_1G01960 AFUA_7G01960 AFUA_6G14150 AFUA_7G04740* | Sterol regulatory element binding protein, induces transcription of sterol biosynthetic genes and of DAN/TIR gene products; Ecm22p homolog; relocates from intracellular membranes to perinuclear foci on sterol depletion |
| *GAL83* | *YER027C YGL208W* | *AFUA_6G04500* | One of three beta subunits of the Snf1 serine/threonine protein kinase complex involved in the response to glucose starvation; null mutants exhibit accelerated aging; N-myristoylprotein localized to the cytoplasm and the plasma membrane |
| *GIS1* | *YER169W YDR096W* | *AFUA_1G12332* | Jumonji family transcription factor |
| *HAS1* | *YMR290C* | *AFUA_4G13330* | ATP-dependent RNA helicase; localizes to both the nuclear periphery and nucleolus; highly enriched in nuclear pore complex fractions; constituent of 66S pre-ribosomal particles |
| *HIR3* | *YJR140C* | *AFUA_3G12810* | Transcriptional corepressor of histone genes (Hir3), putative |
| *HPC2* | *YBR215W* | *AFUA_3G05910* | Hpc2p |
| *HST1* | *YOL068C YDL042C* | *AFUA_4G12120* | Conserved NAD+ dependent histone deacetylase of the Sirtuin family involved in regulation of lifespan; plays roles in silencing at HML, HMR, telomeres, and the rDNA locus; negatively regulates initiation of DNA replication |
| *HST2* | *YPL015C* | *AFUA_2G05900 AFUA_3G00520* | Cytoplasmic member of the silencing information regulator 2 family of NAD(+)-dependent protein deacetylases; modulates nucleolar and telomeric silencing; possesses NAD(+)-dependent histone deacetylase activity in vitro |
| *HST4* | *YDR191W* | *AFUA_1G10540* | SIR2 family histone deacetylase (Hst4) |
| *INO80* | *YGL150C* | *AFUA_5G06260* | ATPase, subunit of a complex containing actin and several actin-related proteins that has chromatin remodeling activity and 3' to 5' DNA helicase activity in vitro; has a role in modulating stress gene transcription |
| *ISW1* | *YBR245C* | *AFUA_4G13460* | SNF2 family helicase/ATPase |
| *JHD2* | *YJR119C* | *AFUA_5G03430* | PHD transcription factor (Rum1) |
| *KCS1* | *YDR017C* | *AFUA_5G13240* | Inositol hexakisphosphate and inositol heptakisphosphate kinase; generation of high energy inositol pyrophosphates by Kcs1p is required for many processes such as vacuolar biogenesis, stress response and telomere maintenance |
| *MAL13* | *YFL052W YGR288W YBR297W YPR196W* | *AFUA_2G04600 AFUA_7G06370 AFUA_8G07230 AFUA_1G17150 AFUA_4G10160* | MAL-activator protein, part of complex locus; nonfunctional in genomic reference strain S288C |
| *MET31* | *YPL038W YDR253C* | *AFUA_6G01910* | C2H2 finger domain protein |
| *MOB1* | *YIL106W* | *AFUA_2G12390* | Component of the mitotic exit network; associates with and is required for the activation and Cdc15p-dependent phosphorylation of the Dbf2p kinase; required for cytokinesis and cell separation; component of the CCR4 transcriptional complex |
| *MOT3* | *YMR070W* | *AFUA_5G06190* | Sexual development transcription factor SteA |
| *NIF3* | *YGL221C* | *AFUA_6G12480* | NGG1 interacting factor Nif3 |
| *OAF1* | *YOR363C YAL051W YCR106W YLL054C* | *AFUA_2G15340 AFUA_5G10310* | Autoregulatory oleate-specific transcriptional activator of peroxisome proliferation, contains Zn(2)-Cys(6) cluster domain, forms heterodimer with Oaf1p, binds oleate response elements (OREs), activates beta-oxidation genes |
| *PDC1* | *YDL080C YGR087C YLR044C YLR134W* | *AFUA_3G11070* | Minor isoform of pyruvate decarboxylase, key enzyme in alcoholic fermentation, decarboxylates pyruvate to acetaldehyde is glucose- and ethanol-dependent thiamine, involved in amino acid catabolism |
| *PHO23* | *YNL097C YHR090C YOR064C* | *AFUA_7G01870 AFUA_3G11940 AFUA_4G11660* | Subunit of histone acetyltransferase complex that acetylates histone PHD finger domain that histone, has similarity to the human tumor suppressor ING1 |
| *REB1* | *YBR049C YDR026C* | *AFUA_2G02350* | Protein of unknown function that may interact with ribosomes, based on co-purification experiments; Myb-like DNA-binding protein that may bind to the Ter region of rDNA; interacts physically with Fob1p |
| *RPC11* | *YDR045C* | *AFUA_1G06380* | RNA polymerase III subunit C11 |
| *RTT107* | *YHR154W* | *AFUA_4G12310* | Protein implicated in Mms22-dependent DNA repair during S phase, DNA damage induces phosphorylation by Mec1p at one or more SQ/TQ motifs; interacts with Mms22p and Slx4p; has four BRCT domains; has a role in regulation of Ty1 transposition |
| *SAS10* | *YDL153C* | *AFUA_1G11160* | Essential subunit of U3-containing Small Subunit processome complex involved in production of 18S rRNA and assembly of small ribosomal subunit; disrupts silencing when overproduced |
| *SAS3* | *YBL052C* | *AFUA_4G10910* | Histone acetyltransferase |
| *SSY1* | *YDR160W* | *AFUA_4G00240* | Component of the SPS plasma membrane amino acid sensor system (Ssy1p-Ptr3p-Ssy5p), which senses external amino acid concentration and transmits intracellular signals that result in regulation of expression of amino acid permease genes |
| *STB2* | *YMR053C YKL072W* | *AFUA_1G04480* | Protein that interacts with Sin3p in a two-hybrid assay and is part of a large protein complex with Sin3p and Stb1p |
| *STB5* | *YHR178W* | *AFUA_4G12570* | C6 transcription factor |
| *SWD1* | *YAR003W* | *AFUA_1G14670* | Subunit of the COMPASS complex, which methylates histone H3 on lysine 4 and is required in transcriptional silencing near telomeres; WD40 beta propeller superfamily member with similarity to mammalian Rbbp7 |
| *SWR1* | *YDR334W* | *AFUA_7G02370* | Swi2/Snf2-related ATPase that is the structural component of the SWR1 complex, which exchanges histone variant H2AZ for chromatin-bound histone H2A |
| *TAF11* | *YML015C* | *AFUA_5G03660* | Transcription initiation factor TFIID subunit beta, putative |
| *TAF12* | *YDR145W* | *AFUA_3G06090* | Transcription initiation factor TFIID subunit 12, putative |
| *TAF3* | *YPL011C* | *AFUA_2G14890* | Bromodomain associated domain protein |
| *THI20* | *YPR121W YOL055C YPL258C* | *AFUA_2G10740* | Hydroxymethylpyrimidine phosphate kinase, involved thiamine biosynthesis; member of a gene family with THI20 and THI22 activity |
| *TRM7* | *YBR061C* | *AFUA_6G08910* | 2'-O-ribose tRNA anticodon loop methyltransferase |
| *XBP1* | *YIL101C* | *AFUA_6G13680* | Transcriptional repressor that binds to promoter sequences of the cyclin genes, CYS3, and SMF2; expression is induced by stress or starvation during mitosis, and late in meiosis; member of the Swi4p/Mbp1p family; potential Cdc28p substrate |
| *YAF9* | *YNL107W* | *AFUA_4G09820* | Subunit of both the NuA4 histone H4 acetyltransferase complex and the SWR1 complex, may function to antagonize silencing near telomeres; interacts directly with Swc4p, has homology to human leukemogenic protein AF9, contains a YEATS domain |
|  | *YLR278C* | *AFUA_2G08240* | C6 transcription factor |
|  | *YER130C* | *AFUA_4G09080* | C2H2 transcription factor (Seb1) |
| *DOM34* | *YNL001W* | *AFUA_2G02960* | Translation factor pelota |
| *GCD14* | *YJL125C* | *AFUA_5G09620* | 1-methyladenosine tRNA methyltransferase subunit |
| *PTH1* | *YHR189W* | *AFUA_5G05940* | One of two mitochondrially-localized peptidyl-tRNA hydrolases; dispensable for respiratory growth on rich medium, but required for respiratory growth on minimal medium |
| *SHP1* | *YBL058W* | *AFUA_5G03610* | Cdc48-dependent protein degradation adaptor protein (Shp1) |
| *THO2* | *YNL139C* | *AFUA_5G07180* | Subunit of the THO complex, which is required for efficient transcription elongation and involved in transcriptional elongation-associated recombination; required for LacZ RNA expression from certain plasmids |
| *AIM10* | *YER087W* | *AFUA_1G10750* | Prolyl-tRNA synthetase |
| *DED81* | *YHR019C* | *AFUA_2G05650* | Cytoplasmic asparaginyl-tRNA synthetase |
| *DIA4* | *YHR011W* | *AFUA_3G12640* | Seryl-tRNA synthetase |
| *GUS1* | *YGL245W* | *AFUA_5G03560* | Glutamyl-tRNA synthetase |
| *MSR1* | *YHR091C YDR341C* | *AFUA_2G14030* | Arginyl-tRNA synthetase |
|  | *YHR020W* | *AFUA_2G16010* | Protein of unknown function that may interact with ribosomes, based on co-purification experiments; has similarity to proline-tRNA ligase; YHR020W is an essential gene |
|  | *YNL247W* | *AFUA_5G09610* | Cysteinyl-tRNA synthetase; may interact with ribosomes, based on co-purification experiments |
| *AOS1* | *YPR180W* | *AFUA_5G06100* | SUMO activating enzyme (AosA) |
| *APC2* | *YLR127C* | *AFUA_2G05210* | Anaphase-promoting complex subunit ApcB |
| *APC5* | *YOR249C* | *AFUA_3G06200* | Anaphase-promoting complex subunit Apc5 |
| *BSC5* | *YNR069C YML111W YMR275C* | *AFUA_6G13380* | Arrestin (or S-antigen), N-terminal domain protein |
| *CDC48* | *YDL126C* | *AFUA_2G17110* | ATPase in ER, nuclear membrane and cytosol with homology to mammalian p97; in a complex with Npl4p and Ufd1p participates in retrotranslocation of ubiquitinated proteins from the ER into the cytosol for degradation by the proteasome |
| *CDC53* | *YDL132W* | *AFUA_1G12960* | SCF ubiquitin ligase complex subunit CulA |
| *DOA1* | *YKL213C* | *AFUA_5G08370* | Polyubiquitin binding protein (Doa1/Ufd3) |
| *DOA4* | *YER144C YDR069C* | *AFUA_2G04720* | Putative ubiquitin-specific protease, closest paralog of Doa4p but has no functional overlap; concentrates at the bud neck |
| *DOC1* | *YGL240W* | *AFUA_1G15770* | Anaphase promoting complex subunit 10 (APC10) |
| *NAS6* | *YGR232W* | *AFUA_1G10700* | Proteasome regulatory particle subunit (Nas6) |
| *PRB1* | *YEL060C YOR003W* | *AFUA_5G09210* | Putative precursor to the subtilisin-like protease III |
| *PUP3* | *YER094C* | *AFUA_4G07420* | Beta 3 subunit of the 20S proteasome involved in ubiquitin-dependent catabolism; human homolog is subunit C10 |
| *RPN1* | *YHR027C* | *AFUA_5G11720* | Non-ATPase base subunit of the 19S regulatory particle of the 26S proteasome; may participate in the recognition of several ligands of the proteasome; contains a leucine-rich repeat domain, a site for protein-protein interactions |
| *RPN10* | *YHR200W* | *AFUA_2G15070* | Non-ATPase base subunit of the 19S regulatory particle of the 26S proteasome; N-terminus plays a role in maintaining the structural integrity of the RP; binds selectively to polyubiquitin chains; homolog of the mammalian S5a protein |
| *RPN11* | *YFR004W* | *AFUA_2G03400* | Metalloprotease subunit of the 19S regulatory particle of the 26S proteasome lid; couples the deubiquitination and degradation of proteasome substrates; involved, independent of catalytic activity, in fission of mitochondria and peroxisomes |
| *RPN2* | *YIL075C* | *AFUA_4G08480* | 26S proteasome regulatory subunit Rpn2 |
| *RPN3* | *YER021W* | *AFUA_3G06110* | Essential, non-ATPase regulatory subunit of the 26S proteasome lid, similar to the p58 subunit of the human 26S proteasome; temperature-sensitive alleles cause metaphase arrest, suggesting a role for the proteasome in cell cycle control |
| *RPN5* | *YDL147W* | *AFUA_3G06610* | Essential, non-ATPase regulatory subunit of the 26S proteasome lid, similar to mammalian p55 subunit and to another S. cerevisiae regulatory subunit, Rpn7p |
| *RPN6* | *YDL097C* | *AFUA_1G06300* | Essential, non-ATPase regulatory subunit of the 26S proteasome lid required for the assembly and activity of the 26S proteasome; the human homolog partially rescues Rpn6p depletion |
| *RPN7* | *YPR108W* | *AFUA_6G07760* | Essential, non-ATPase regulatory subunit of the 26S proteasome, similar to another S. cerevisiae regulatory subunit, Rpn5p, as well as to mammalian proteasome subunits |
| *RPN8* | *YOR261C* | *AFUA_1G07540* | Essential, non-ATPase regulatory subunit of the 26S proteasome; has similarity to the human p40 proteasomal subunit and to another S. cerevisiae regulatory subunit, Rpn11p |
| *RPT2* | *YDL007W* | *AFUA_5G07050* | One of six ATPases of the 19S regulatory particle of the 26S proteasome involved in the degradation of ubiquitinated substrates; required for normal peptide hydrolysis by the core 20S particle |
| *RPT3* | *YDR394W* | *AFUA_3G11390* | One of six ATPases of the 19S regulatory particle of the 26S proteasome involved in the degradation of ubiquitinated substrates; substrate of N-acetyltransferase B |
| *RPT5* | *YOR117W* | *AFUA_1G06170* | One of six ATPases of the 19S regulatory particle of the 26S proteasome involved in the degradation of ubiquitinated substrates; recruited to the GAL1-10 promoter region upon induction of transcription |
| *RPT6* | *YGL048C* | *AFUA_4G04660* | One of six ATPases of the 19S regulatory particle of the 26S proteasome involved in the degradation of ubiquitinated substrates; bound by ubiquitin-protein ligases Ubr1p and Ufd4p; localized mainly to the nucleus throughout the cell cycle |
| *UBC11* | *YOR339C* | *AFUA_6G13170* | Ubiquitin conjugating enzyme (UbcK) |
| *UBC13* | *YDR092W* | *AFUA_6G02420* | Ubiquitin conjugating enzyme (UbcM) |
| *UBP12* | *YJL197W* | *AFUA_2G14130* | Ubiquitin carboxyl-terminal hydrolase, ubiquitin-specific protease present in the nucleus and cytoplasm that cleaves ubiquitin from ubiquitinated proteins |
| *UBP13* | *YBL067C YER098W* | *AFUA_4G12910* | Putative ubiquitin carboxyl-terminal hydrolase, ubiquitin-specific protease that cleaves ubiquitin-protein fusions |
| *UBP14* | *YBR058C* | *AFUA_2G06330* | Ubiquitin-specific protease that specifically disassembles unanchored ubiquitin chains; involved in fructose-1,6-bisphosphatase degradation; similar to human isopeptidase T |
| *UBP8* | *YMR223W* | *AFUA_6G12710* | Ubiquitin-specific protease that is a component of the SAGA acetylation complex; required for SAGA-mediated deubiquitination of histone H2B |
| *UFD1* | *YGR048W* | *AFUA_1G02430* | Protein that interacts with Cdc48p and Npl4p, involved in recognition of polyubiquitinated proteins and their presentation to the 26S proteasome for degradation; involved in transporting proteins from the ER to the cytosol |
| *UFD2* | *YDL190C* | *AFUA_4G07340* | Ubiquitin chain assembly factor that cooperates with a ubiquitin-activating enzyme to conjugate ubiquitin to substrates; also functions as an E3 |
| *UMP1* | *YBR173C* | *AFUA_5G10740* | Short-lived chaperone required for correct maturation of the 20S proteasome; may inhibit premature dimerization of proteasome half-mers; degraded by proteasome upon completion of its assembly |
|  | *YFR006W* | *AFUA_2G07500* | Putative X-Pro aminopeptidase; green fluorescent protein (GFP)-fusion protein localizes to the cytoplasm; YFR006W is not an essential gene |
|  | *YHR113W* | *AFUA_3G08290* | Cytoplasmic aspartyl aminopeptidase; cleaves unblocked N-terminal acidic amino acid residues from peptide substrates; forms a 12 subunit homo-oligomeric complex; M18 metalloprotease family member; may interact with ribosomes |
| *ALG11* | *YNL048W* | *AFUA_1G06890* | Alpha-1,2-mannosyltransferase (Alg11) |
| *ALG2* | *YGL065C* | *AFUA_5G13210* | Alpha-1,2-mannosyltransferase (Alg2) |
| *ALG6* | *YOR002W* | *AFUA_3G07700* | Glucosyltransferase |
| *BAR1* | *YIL015W YIR039C YLR121C YLR120C YDR144C* | *AFUA_6G05350* | Aspartic protease, attached to the plasma membrane via a glycosylphosphatidylinositol anchor |
| *BET2* | *YPR176C* | *AFUA_7G04460* | Rab geranylgeranyltransferase, beta subunit |
| *BPL1* | *YDL141W* | *AFUA_2G09550* | Biotin:apoprotein ligase, covalently modifies proteins with the addition of biotin, required for acetyl-CoA carboxylase holoenzyme formation |
| *ELP3* | *YPL086C* | *AFUA_5G06140* | Histone acetyltransferase |
| *ERP5* | *YHR110W* | *AFUA_4G07390* | Endosomal cargo receptor (Erp5) |
| *ESA1* | *YOR244W* | *AFUA_2G05530* | Histone acetyltransferase (Esa1) |
| *GRX3* | *YER174C YDR098C* | *AFUA_2G14960* | Thioredoxin |
| *GUF1* | *YLR289W* | *AFUA_3G14350* | GTP binding protein Guf1 |
| *HOS2* | *YGL194C* | *AFUA_2G03810* | Histone deacetylase required for gene activation via specific deacetylation of lysines in H3 and H4 histone tails; subunit of the Set3 complex, a meiotic-specific repressor of sporulation specific genes that contains deacetylase activity |
| *HPA2* | *YEL066W YPR193C* | *AFUA_4G02980* | D-Amino acid N-acetyltransferase, catalyzes N-acetylation of D-amino acids through ordered bi-bi mechanism in which acetyl-CoA is first substrate bound and CoA is last product liberated; similar to Hpa2p, acetylates histones weakly in vitro |
| *KTR1* | *YBR205W YOR099W* | *AFUA_5G10760* | Alpha-1,2-mannosyltransferase involved in O- and N-linked protein glycosylation; type II membrane protein; member of the KRE2/MNT1 mannosyltransferase family |
| *KTR4* | *YBR199W* | *AFUA_5G02740* | Alpha-1,2-mannosyltransferase (Ktr4) |
| *KTR5* | *YIL085C YNL029C* | *AFUA_5G12160* | Alpha-1,2-mannosyltransferase (Kre5) |
| *MAF1* | *YDR005C* | *AFUA_2G01550* | Mitogen-activated protein kinase MAF1 |
| *MCX1* | *YBR227C* | *AFUA_1G02170* | ATP-dependent Clp protease |
| *MNN1* | *YGL257C YNR059W YER001W YIL014W* | *AFUA_6G14480* | Alpha-1,3-mannosyltransferase alpha-1,3-linked mannose residues to O-linked glycans O |
| *MNN4* | *YJR061W YKL201C* | *AFUA_1G03790 AFUA_1G12630* | Putative protein of unknown function; non-essential gene with similarity to Mnn4, a putative membrane protein involved in glycosylation; transcription repressed by Rm101p |
| *NAT3* | *YPR131C* | *AFUA_1G05180* | Catalytic subunit of the NatB N-terminal acetyltransferase, which catalyzes acetylation of the amino-terminal methionine residues of all proteins beginning with Met-Asp or Met-Glu and of some proteins beginning with Met-Asn or Met-Met |
| *NBP2* | *YDR162C* | *AFUA_2G03680* | High osmolarity glycerol pathway protein Nbp2 |
| *NPL6* | *YMR091C* | *AFUA_1G06410* | Component of the RSC chromatin remodeling complex; interacts with Rsc3p, Rsc30p, Ldb7p, and Htl1p to form a module important for a broad range of RSC functions; involved in nuclear protein import and maintenance of proper telomere length |
| *PAN2* | *YGL094C* | *AFUA_2G04650* | Essential subunit of the Pan2p-Pan3p poly(A)-ribonuclease complex, which acts to control poly(A) tail length and regulate the stoichiometry and activity of postreplication repair complexes |
| *PEX10* | *YDR265W* | *AFUA_7G04260* | Peroxisomal membrane E3 ubiquitin ligase required for for Ubc4p-dependent Pex5p ubiquitination and peroxisomal matrix protein import; contains zinc-binding RING domain; mutations in human homolog cause various peroxisomal disorders |
| *PMT1* | *YDL095W YDL093W* | *AFUA_3G06450* | Protein mannosyltransferase 1 |
| *RCE1* | *YMR274C* | *AFUA_6G04890* | CaaX prenyl proteinase Rce1 |
| *RDL1* | *YOR285W YOR286W* | *AFUA_1G03960* | Protein with rhodanese activity; contains a rhodanese-like domain similar to Rdl1p, Uba4p, Tum1p, and Ych1p; overexpression causes a cell cycle delay; null mutant displays elevated frequency of mitochondrial genome loss |
| *RIA1* | *YNL163C* | *AFUA_5G13520* | Cytoplasmic GTPase involved in biogenesis of the 60S ribosome; has similarity to translation elongation factor 2 (Eft1p and Eft2p) |
| *RIM13* | *YMR154C* | *AFUA_1G03450* | Calpain-like cysteine protease involved in proteolytic activation of Rim101p in response to alkaline pH; has similarity to A. nidulans palB |
| *RIM15* | *YFL033C* | *AFUA_2G15010* | Glucose-repressible protein kinase involved in signal transduction during cell proliferation in response to nutrients, specifically the establishment of stationary phase; identified as a regulator of IME2; substrate of Pho80p-Pho85p kinase |
| *SMT3* | *YDR510W* | *AFUA_1G10850* | Ubiquitin-like modifier SUMO |
| *STE23* | *YLR389C* | *AFUA_5G02010* | Metalloprotease involved, with homolog Axl1p, in N-terminal processing of pro-a-factor to the mature form; member of the insulin-degrading enzyme family |
| *SUA5* | *YGL169W* | *AFUA_2G09160* | Single-stranded telomeric DNA-binding protein, required for normal telomere length; null mutant lacks N6-threonylcarbamoyl adenosine modification in the anticodon loop of ANN-decoding tRNA; member of conserved YrdC/Sua5 family |
| *TRX1* | *YCR083W YGR209C YLR043C* | *AFUA_3G14970* | Cytoplasmic thioredoxin isoenzyme of the thioredoxin system which protects cells against oxidative and reductive stress, forms LMA1 complex with Pbi2p, acts as a cofactor for Tsa1p, required for ER-Golgi transport and vacuole inheritance |
| *UBA3* | *YPR066W* | *AFUA_2G13730* | Protein that acts together with Ula1p to activate Rub1p before its conjugation to proteins (neddylation), which may play a role in protein degradation; GFP-fusion protein localizes to the cytoplasm in a punctate pattern |
| *UBC12* | *YLR306W* | *AFUA_3G14430* | Enzyme that mediates the conjugation of Rub1p, a ubiquitin-like protein, to other proteins; related to E2 ubiquitin-conjugating enzymes |
| *UBC8* | *YEL012W* | *AFUA_6G09160* | Ubiquitin conjugating enzyme Ubc8 |
| *UBP6* | *YFR010W* | *AFUA_6G02380* | Ubiquitin-specific protease situated in the base subcomplex of the 26S proteasome, releases free ubiquitin from branched polyubiquitin chains; works in opposition to polyubiquitin elongation activity of Hul5p |
| *UGP1* | *YKL035W* | *AFUA_7G01830* | UDP-glucose pyrophosphorylase (UGPase), catalyses the reversible formation of UDP-Glc from glucose 1-phosphate and UTP, involved in a wide variety of metabolic pathways, expression modulated by Pho85p through Pho4p |
| *ULA1* | *YPL003W* | *AFUA_6G10600* | Protein that acts together with Uba3p to activate Rub1p before its conjugation to proteins (neddylation), which may play a role in protein degradation |
| *UPS2* | *YLR168C YDR185C* | *AFUA_4G05920* | Mitochondrial protein of unknown function; similar to Ups1p and Ups2p which are involved in regulation of mitochondrial cardiolipin and phosphatidylethanolamine levels; null is viable but interacts synthetically with ups1 and ups2 mutations |
| *VPS13* | *YLL040C* | *AFUA_4G11560* | Protein of unknown function; heterooligomeric or homooligomeric complex; peripherally associated with membranes; homologous to human COH1; involved in sporulation, vacuolar protein sorting and protein-Golgi retention |
| *VPS17* | *YOR132W* | *AFUA_5G07150* | Subunit of the membrane-associated retromer complex essential for endosome-to-Golgi retrograde protein transport; peripheral membrane protein that assembles onto the membrane with Vps5p to promote vesicle formation |
| *VPS36* | *YLR417W* | *AFUA_4G04100* | Component of the ESCRT-II complex; contains the GLUE domain which is involved in interactions with ESCRT-I and ubiquitin-dependent sorting of proteins into the endosome |
| *YIP1* | *YGR172C* | *AFUA_4G04630* | Integral membrane protein required for the biogenesis of ER-derived COPII transport vesicles; interacts with Yif1p and Yos1p; localizes to the Golgi, the ER, and COPII vesicles |
| *YND1* | *YER005W* | *AFUA_5G11010* | Apyrase with wide substrate specificity, involved in preventing the inhibition of glycosylation by hydrolyzing nucleoside tri- and diphosphates which are inhibitors of glycotransferases; partially redundant with Gda1p |
| *IMP3* | *YHR148W* | *AFUA_2G08320* | Component of the SSU processome, which is required for pre-18S rRNA processing, essential protein that interacts with Mpp10p and mediates interactions of Imp4p and Mpp10p with U3 snoRNA |
| *MRPL1* | *YDR116C* | *AFUA_5G03570* | Mitochondrial large ribosomal subunit protein L1, putative |
| *MRPS12* | *YNR036C* | *AFUA_5G10750* | Mitochondrial protein; may interact with ribosomes based on co-purification experiments; similar to E. coli and human mitochondrial S12 ribosomal proteins |
| *MRT4* | *YKL009W* | *AFUA_5G13470* | 60S acidic ribosomal protein P0 |
| *RLP24* | *YLR009W* | *AFUA_2G02710* | Essential protein with similarity to Rpl24Ap and Rpl24Bp, associated with pre-60S ribosomal subunits and required for ribosomal large subunit biogenesis |
| *RSM10* | *YDR041W* | *AFUA_6G05330* | Mitochondrial ribosomal protein of the small subunit, has similarity to E. coli S10 ribosomal protein; essential for viability, unlike most other mitoribosomal proteins |
| *SWS2* | *YNL081C* | *AFUA_4G07640* | Putative mitochondrial ribosomal protein of the small subunit, has similarity to E. coli S13 ribosomal protein; participates in controlling sporulation efficiency |
|  | *YDR115W* | *AFUA_4G07605* | Putative mitochondrial ribosomal protein of the large subunit, has similarity to E. coli L34 ribosomal protein; required for respiratory growth, as are most mitochondrial ribosomal proteins |
| *RIT1* | *YMR283C* | *AFUA_5G08280* | TRNA a64-2'-o-ribosylphosphate transferase |
| *TAD1* | *YGL243W* | *AFUA_1G05710* | TRNA-specific adenosine deaminase |
| *ADY2* | *YCR010C YNR002C* | *AFUA_2G04080* | Acetate transporter required for normal sporulation; phosphorylated in mitochondria |
| *AKR1* | *YOR034C YDR264C* | *AFUA_2G07670* | Palmitoyltransferase SidR |
| *CLA4* | *YOL113W YNL298W* | *AFUA_5G05900* | Member of the PAK family of serine/threonine protein kinases with similarity to Ste20p and Cla4p; involved in down-regulation of sterol uptake; proposed to be a downstream effector of Cdc42p during polarized growth |
| *DIE2* | *YGR227W* | *AFUA_2G11080* | Glucosyltransferase (Die2) |
| *FIG4* | *YNL325C* | *AFUA_2G16640* | Polyphosphoinositide phosphatase Fig4 |
| *GAL1* | *YBR020W YDR009W* | *AFUA_3G10300* | Galactokinase |
| *GLC8* | *YMR311C* | *AFUA_5G03700* | Glc8p |
| *GPA2* | *YER020W* | *AFUA_1G12930* | G protein complex alpha subunit GpaB |
| *HSE1* | *YHL002W* | *AFUA_2G04670* | SH3 domain protein |
| *INM1* | *YHR046C YDR287W* | *AFUA_3G04250* | Inositol monophosphatase |
| *ISC1* | *YER019W* | *AFUA_6G08370* | Sphingomyelinase family protein |
| *KEL1* | *YGR238C YHR158C* | *AFUA_2G02520* | Cell polarity protein (Tea1) |
| *KRE33* | *YNL132W* | *AFUA_1G05310* | Essential protein, required for biogenesis of the small ribosomal subunit; heterozygous mutant shows haploinsufficiency in K1 killer toxin resistance |
| *MSG5* | *YIL113W YNL053W* | *AFUA_2G02760* | Dual-specificity protein phosphatase; exists in 2 isoforms; required for maintenance of a low level of signaling through the cell integrity pathway, adaptive response to pheromone; regulates and is regulated by Slt2p; dephosphorylates Fus3p |
| *NPR2* | *YEL062W* | *AFUA_1G04580* | Component of an evolutionarily conserved Npr2/3 complex that mediates downregulation of TORC1 activity in response to amino acid limitation; homolog of human NPRL2; target of Grr1p (E3 ligase); required for growth on urea and proline |
| *PLC1* | *YPL268W* | *AFUA_1G13250* | Phospholipase C, hydrolyzes phosphatidylinositol 4,5-biphosphate to generate the signaling molecules inositol 1,4,5-triphosphate and 1,2-diacylglycerol (DAG); involved in regulating many cellular processes |
| *PSY2* | *YNL201C* | *AFUA_5G07830* | DUF625 domain protein |
| *RGA1* | *YOR127W YDR379W* | *AFUA_1G12680* | GTPase-activating protein for the polarity-establishment protein Cdc42p; implicated in control of septin organization, pheromone response, and haploid invasive growth; regulated by Pho85p and Cdc28p |
| *RGD1* | *YBR260C* | *AFUA_3G06280* | GTPase-activating protein for Rho3p and Rho4p, possibly involved in control of actin cytoskeleton organization |
| *RHB1* | *YCR027C* | *AFUA_5G05480* | Putative Rheb-related GTPase involved in regulating canavanine resistance and arginine uptake; member of the Ras superfamily of G-proteins |
| *ROM1* | *YGR070W YLR371W* | *AFUA_5G08550* | GDP/GTP exchange protein for Rho1p and Rho2p; mutations are synthetically lethal with mutations in rom1, which also encodes a GEP |
| *SGD1* | *YLR336C* | *AFUA_2G01980* | Essential nuclear protein, required for biogenesis of the small ribosomal subunit; has a possible role in the osmoregulatory glycerol response; putative homolog of human NOM1 which is implicated in acute myeloid leukemia |
| *SIP3* | *YHR155W YNL257C* | *AFUA_1G05420* | Mitochondrial protein with a potential role in promoting mitochondrial fragmentation during programmed cell death in response to high levels of alpha-factor mating pheromone or the drug amiodarone |
| *SKS1* | *YPL026C YDR247W* | *AFUA_3G10530* | Cytoplasmic serine/threonine protein kinase; identified as a high-copy suppressor of the synthetic lethality of a sis2 sit4 double mutant, suggesting a role in G1/S phase progression; homolog of Sks1p |
| *SMP3* | *YOR149C* | *AFUA_5G06050* | Alpha 1,2-mannosyltransferase involved in glycosyl phosphatidyl inositol biosynthesis; required for addition of the fourth, side branching mannose to the GPI core structure |
| *SNF8* | *YPL002C* | *AFUA_4G03870* | Component of the ESCRT-II complex, which is involved in ubiquitin-dependent sorting of proteins into the endosome; appears to be functionally related to SNF7; involved in glucose derepression |
| *STE11* | *YLR362W* | *AFUA_5G06420* | Signal transducing MEK kinase involved in pheromone response and pseudohyphal/invasive growth pathways where it phosphorylates Ste7p, and the high osmolarity response pathway, via phosphorylation of Pbs2p; regulated by Ste20p and Ste50p |
| *STE5* | *YDR103W* | *AFUA_6G06990* | Pheromone-response scaffold protein; binds Ste11p, Ste7p, and Fus3p kinases, forming a MAPK cascade complex that interacts with the plasma membrane and Ste4p-Ste18p; allosteric activator of Fus3p that facilitates Ste7p-mediated activation |
| *STE50* | *YCL032W* | *AFUA_2G17130* | Protein involved in mating response, invasive/filamentous growth, and osmotolerance, acts as an adaptor that links G protein-associated Cdc42p-Ste20p complex to the effector Ste11p to modulate signal transduction |
| *STE7* | *YDL159W* | *AFUA_3G05900* | Signal transducing MAP kinase kinase involved in pheromone response, where it phosphorylates Fus3p, and in the pseudohyphal/invasive growth pathway, through phosphorylation of Kss1p; phosphorylated by Ste11p, degraded by ubiquitin pathway |
| *STM1* | *YLR150W* | *AFUA_3G10920* | Protein required for optimal translation under nutrient stress; perturbs association of Yef3p with ribosomes; involved in TOR signaling; binds G4 quadruplex and purine motif triplex nucleic acid; helps maintain telomere structure |
| *STT4* | *YLR305C* | *AFUA_7G03760* | Phosphatidylinositol-4-kinase that functions in the Pkc1p protein kinase pathway; required for normal vacuole morphology, cell wall integrity, and actin cytoskeleton organization |
| *TAP42* | *YMR028W* | *AFUA_5G11780* | Essential protein involved in the TOR signaling pathway; physically associates with the protein phosphatase 2A and the SIT4 protein phosphatase catalytic subunits |
| *TEM1* | *YML064C* | *AFUA_6G10330* | GTP-binding protein of the ras superfamily involved in termination of M-phase; controls actomyosin and septin dynamics during cytokinesis |
| *TEP1* | *YNL128W* | *AFUA_2G11990* | Homolog of human tumor suppressor gene PTEN/MMAC1/TEP1 that has lipid phosphatase activity and is linked to the phosphatidylinositol signaling pathway; plays a role in normal sporulation |
| *TFS1* | *YLR178C YLR179C* | *AFUA_4G08120* | Protein of unknown function with similarity to Tfs1p; transcription is activated by paralogous proteins Yrm1p and Yrr1p along with proteins involved in multidrug resistance; GFP-tagged protein localizes to the cytoplasm and nucleus |
| *TOR1* | *YJR066W YKL203C* | *AFUA_2G10270* | PIK-related protein kinase and rapamycin target; subunit of TORC1, a complex that controls growth in response to nutrients by regulating translation, transcription, ribosome biogenesis, nutrient transport and autophagy; involved in meiosis |
| *UGA1* | *YGR019W* | *AFUA_5G06680* | Gamma-aminobutyrate transaminase involved in the 4-aminobutyrate and glutamate degradation pathways; required for normal oxidative stress tolerance and nitrogen utilization |
| *URE2* | *YNL229C* | *AFUA_3G10830 AFUA_4G14530 AFUA_7G05500* | Glutathione S-transferase |
| *VPS45* | *YGL095C* | *AFUA_6G04870* | Protein of the Sec1p/Munc-18 family, essential for vacuolar protein sorting; required for the function of Pep12p and the early endosome/late Golgi SNARE Tlg2p; essential for fusion of Golgi-derived vesicles with the prevacuolar compartment |
| *YPD1* | *YDL235C* | *AFUA_4G10280* | Phosphorelay intermediate protein, phosphorylated by the plasma membrane sensor Sln1p in response to osmotic stress and then in turn phosphorylates the response regulators Ssk1p in the cytosol and Skn7p in the nucleus |
| *YVH1* | *YIR026C* | *AFUA_4G07080* | Protein phosphatase involved in vegetative growth at low temperatures, sporulation, and glycogen accumulation; mutants are defective in 60S ribosome assembly; member of the dual-specificity family of protein phosphatases |
| *ALG12* | *YNR030W* | *AFUA_4G12900* | Alpha-1,6-mannosyltransferase subunit (Ecm39) |
| *ALG9* | *YNL219C* | *AFUA_1G13870* | Alpha-1,2-mannosyltransferase (Alg9) |
| *ASP1* | *YDR321W YLR155C YLR157C YLR158C YLR160C* | *AFUA_1G02780* | Asp3 |
| *BGL2* | *YGR282C* | *AFUA_1G11460* | 1,3-beta-glucanosyltransferase Bgt1 |
| *BUD4* | *YJR092W* | *AFUA_2G08470* | GTP binding protein (Bud4) |
| *CAX4* | *YGR036C* | *AFUA_6G04240* | PAP2 domain protein |
| *DAN1* | *YOR009W YER011W YOR010C YBR067C YIL011W YBL108C-A YJR150C YJR151C YBR301W YEL049W YGR294W YDR542W YHL046C YIL176C YJL223C YGL261C YAL068C YOL161C YLR461W YLL064C YNR076W YFL020C YKL224C YIR041W YCR104W YMR325W YOR394W YPL282C YLL025W YLR037C YAR020C* | *AFUA_4G03360* | Cell wall mannoprotein with similarity to Tir1p, Tir2p, Tir3p, and Tir4p; member of the seripauperin multigene family encoded mainly in subtelomeric regions; expressed under anaerobic conditions, completely repressed during aerobic growth |
| *DCG1* | *YIR030C* | *AFUA_1G13380* | Hydantoin racemase (Dcg1) |
| *ECM11* | *YDR446W* | *AFUA_2G10880* | Ecm11p |
| *ECM14* | *YHR132C* | *AFUA_2G08790* | Zinc carboxypeptidase |
| *ECM15* | *YBL001C* | *AFUA_2G04880 AFUA_4G09900* | Cell wall biogenesis protein Ecm15 |
| *ECM2* | *YBR065C* | *AFUA_4G03640* | Pre-RNA splicing factor Slt11 |
| *ECM23* | *YCR018C YPL021W* | *AFUA_3G05780* | Protein involved in the processing of pre-rRNA to mature rRNA; contains a C2/C2 zinc finger motif; srd1 mutation suppresses defects caused by the rrp1-1 mutation |
| *ECM29* | *YHL030W* | *AFUA_6G06540* | Proteasome component (Ecm29) |
| *ECM31* | *YBR176W* | *AFUA_6G09170* | 3-methyl-2-oxobutanoate hydroxymethyltransferase PanB |
| *ECM38* | *YLR299W* | *AFUA_7G04760* | Gamma-glutamyltranspeptidase |
| *ECM4* | *YGR154C YMR251W YKR076W* | *AFUA_2G15770* | Cell wall biogenesis protein/glutathione transferase (Gto1) |
| *ECM5* | *YMR176W* | *AFUA_5G03430* | PHD transcription factor (Rum1) |
| *EXG1* | *YLR300W YOR190W* | *AFUA_1G03600* | Sporulation-specific exo-1,3-beta-glucanase; contributes to ascospore thermoresistance |
| *GDH1* | *YOR375C YAL062W* | *AFUA_4G06620* | Glutamate/Leucine/Phenylalanine/Valine dehydrogenase, putative |
| *GPI10* | *YGL142C* | *AFUA_4G09130* | Mannosyltransferase |
| *HCA4* | *YJL033W* | *AFUA_6G11070* | DEAD box RNA helicase (Hca4) |
| *MET5* | *YJR137C* | *AFUA_2G15590* | Sulfite reductase |
| *PAH1* | *YMR165C* | *AFUA_1G14610* | Mg2+-dependent phosphatidate phosphatase, catalyzes the dephosphorylation of PA to yield diacylglycerol and Pi, responsible for de novo lipid synthesis; homologous to mammalian lipin 1 |
| *PAN3* | *YKL025C* | *AFUA_1G14310* | Essential subunit of the Pan2p-Pan3p poly(A)-ribonuclease complex, which acts to control poly(A) tail length and regulate the stoichiometry and activity of postreplication repair complexes |
| *PSA1* | *YDL055C* | *AFUA_4G11510* | GDP-mannose pyrophosphorylase (mannose-1-phosphate guanyltransferase), synthesizes GDP-mannose from GTP and mannose-1-phosphate in cell wall biosynthesis; required for normal cell wall structure |
| *QRI1* | *YDL103C* | *AFUA_7G02180* | UDP-N-acetylglucosamine pyrophosphorylase |
| *RGT2* | *YDL194W YDL138W* | *AFUA_6G03060 AFUA_2G14590 AFUA_4G00800* | Plasma membrane low glucose sensor that regulates glucose transport; contains 12 predicted transmembrane segments and a long C-terminal tail required for induction of hexose transporters; also senses fructose and mannose; similar to Rgt2p |
| *RNH1* | *YMR234W* | *AFUA_1G07180* | Ribonuclease H1; able to bind double-stranded RNAs and RNA-DNA hybrids; associates with RNAse polymerase I; the homolog of mammalian RNAse HII (the S. cerevisiae homolog of mammalian RNAse HI is RNH201) |
| *SKI6* | *YGR195W* | *AFUA_2G03740* | Exosome non-catalytic core component; involved in 3'-5' RNA processing and degradation in both the nucleus and the cytoplasm; has similarity to E. coli RNase PH and to human hRrp41p (EXOSC4) |
| *SKT5* | *YBL061C* | *AFUA_8G05620* | Activator of Chs3p (chitin synthase III), recruits Chs3p to the bud neck via interaction with Bni4p; has similarity to Shc1p, which activates Chs3p during sporulation |
| *SPE1* | *YKL184W* | *AFUA_4G08010* | Ornithine decarboxylase, catalyzes the first step in polyamine biosynthesis; degraded in a proteasome-dependent manner in the presence of excess polyamines; deletion decreases lifespan, and increases necrotic cell death and ROS generation |
| *SPE3* | *YPR069C* | *AFUA_1G13490* | Spermidine synthase |
| *AGA1* | *YNR044W YGR014W YDR420W YNL327W YKR102W YHR211W YAL063C YAR050W* | *AFUA_4G00500* | Flo9p |
| *ARK1* | *YIL095W YNL020C* | *AFUA_1G05930* | Serine/threonine protein kinase |
| *BFR1* | *YOR198C* | *AFUA_1G14120* | Nuclear segregation protein (Bfr1) |
| *BMH1* | *YER177W YDR099W* | *AFUA_2G03290* | 14-3-3 protein, minor isoform; controls proteome at post-transcriptional level, binds proteins and DNA, involved in regulation of many processes including exocytosis, vesicle transport, Ras/MAPK signaling, and rapamycin-sensitive signaling |
| *BNS1* | *YGR230W YHR152W* | *AFUA_5G06490* | Nucleolar protein of unknown function, positive regulator of mitotic exit; involved in regulating release of Cdc14p from the nucleolus in early anaphase, may play similar role in meiosis |
| *BOI1* | *YBL085W YER114C* | *AFUA_2G03360* | Polarized growth protein (Boi2) |
| *BRN1* | *YBL097W* | *AFUA_7G03890* | Condensin complex component cnd2 |
| *BUB2* | *YMR055C* | *AFUA_1G02940* | Mitotic check point protein (Bub2) |
| *BUD2* | *YKL092C* | *AFUA_6G12340* | GTPase activating protein (BUD2/CLA2) |
| *CDC123* | *YLR215C* | *AFUA_6G04050* | Cell cycle control protein Cdc123 |
| *CDC14* | *YFR028C* | *AFUA_3G12250* | Protein-tyrosine phosphatase |
| *CDC15* | *YAR019C* | *AFUA_4G06750* | Serine-threonine kinase SepH |
| *CDC28* | *YBR160W* | *AFUA_6G07980* | Catalytic subunit of the main cell cycle cyclin-dependent kinase and G2/M cyclins which direct the CDK to specific substrates |
| *CDC50* | *YCR094W YNR048W* | *AFUA_1G07740* | Protein that interacts specifically in vivo with phospholipid translocase Dnf3p; similar to Cdc50p, which is an essential interaction partner of the flippase Drs2p |
| *CDH1* | *YGL003C* | *AFUA_3G08280* | Cell cycle regulatory protein (Srw1) |
| *CKS1* | *YBR135W* | *AFUA_8G03920* | Cell cycle regulatory protein Cks/Suc1 |
| *CLB1* | *YGR108W YPR119W* | *AFUA_4G12160* | B-type cyclin involved in cell cycle progression; activates Cdc28p to promote the transition from G2 to M phase; accumulates during G2 and M, then targeted via a destruction box motif for ubiquitin-mediated degradation by the proteasome |
| *CLB3* | *YLR210W YDL155W* | *AFUA_2G16150* | B-type cyclin involved in cell cycle progression; activates Cdc28p to promote the G2/M transition; may be involved in DNA replication and spindle assembly; accumulates during S phase and G2, then targeted for ubiquitin-mediated degradation |
| *CLF1* | *YLR117C* | *AFUA_1G10200* | Cell cycle control protein (Cwf4) |
| *CLG1* | *YGL215W* | *AFUA_3G10040* | Cyclin-like protein (Clg1) |
| *CSH1* | *YBR161W YPL057C* | *AFUA_6G04680* | Probable catalytic subunit of a mannosylinositol phosphorylceramide synthase, forms a complex with probable regulatory subunit Csg2p; function in sphingolipid biosynthesis is overlapping with that of Csh1p |
| *DAN4* | *YGR023W YLR332W YHL028W YCR067C YNL327W YKR102W YHR211W YAL063C YAR050W YCR089W YJL078C YMR317W YJR151C YIR019C* | *AFUA_5G13970 AFUA_4G08650* | GPI-anchored cell surface glycoprotein |
| *DBF2* | *YGR092W YPR111W* | *AFUA_6G02840* | Serine/threonine protein kinase |
| *DCW1* | *YMR238W YKL046C* | *AFUA_3G00700 AFUA_4G00620* | Cell wall glycosyl hydrolase Dfg5 |
| *DFG10* | *YIL049W* | *AFUA_1G05470* | 3-oxo-5-alpha-steroid 4-dehydrogenase |
| *DIP2* | *YLR129W* | *AFUA_1G02680* | Small nucleolar ribonucleoprotein complex subunit Dip2, putative |
| *DOT1* | *YDR440W* | *AFUA_5G12110* | Dot1p |
| *DPP1* | *YDR284C* | *AFUA_6G10030* | Diacylglycerol pyrophosphate phosphatase, zinc-regulated vacuolar membrane-associated lipid phosphatase, dephosphorylates DGPP to phosphatidate and Pi, then PA to diacylglycerol; involved in lipid signaling and cell metabolism |
| *ECM33* | *YBR078W YDR055W YCL048W YDR522C* | *AFUA_4G06820* | Protein, redundant organization of the beta-glucan layer of the spore wall a spore wall |
| *ERV14* | *YBR210W YGL054C* | *AFUA_6G07290* | Endosomal cargo receptor (Erv14) |
| *GIN4* | *YCL024W YDR507C* | *AFUA_6G02300* | Serine/threonine protein kinase (Kcc4) |
| *GSG1* | *YDR108W* | *AFUA_2G16670* | Gsg1p |
| *HCS1* | *YKL017C* | *AFUA_2G01090* | DNA helicase |
| *HOF1* | *YMR032W* | *AFUA_3G10250* | Bud neck-localized, SH3 domain-containing protein required for cytokinesis; regulates actomyosin ring dynamics and septin localization; interacts with the formins, Bni1p and Bnr1p, and with Cyk3p, Vrp1p, and Bni5p |
| *HSL7* | *YBR133C* | *AFUA_5G11610* | Protein methyltransferase RmtC |
| *HYM1* | *YKL189W* | *AFUA_3G12480* | Component of the RAM signaling network that is involved in regulation of Ace2p activity and cellular morphogenesis, interacts with Kic1p and Sog2p, localizes to sites of polarized growth during budding and during the mating response |
| *IME2* | *YJL106W* | *AFUA_2G13140* | Meiosis induction protein kinase (Ime2) |
| *IPL1* | *YPL209C* | *AFUA_2G07550 AFUA_4G14740* | Aurora kinase subunit of the conserved chromosomal passenger complex (CPC; Ipl1p-Sli15p-Bir1p-Nbl1p), involved in regulating kinetochore-microtubule attachments; helps maintain condensed chromosomes during anaphase and early telophase |
| *JSN1* | *YPR042C YJR091C* | *AFUA_2G05610* | Member of the PUF protein family, which is defined by the presence of Pumilio homology domains that confer RNA binding activity; preferentially binds mRNAs encoding membrane-associated proteins |
| *LTE1* | *YAL024C* | *AFUA_3G12430* | Guanine nucleotide exchange factor |
| *LUC7* | *YDL087C* | *AFUA_6G09010* | Essential protein associated with the U1 snRNP complex; splicing factor involved in recognition of 5' splice site; contains two zinc finger motifs; N-terminal zinc finger binds pre-mRNA |
| *MAD2* | *YJL030W* | *AFUA_3G14340* | Component of the spindle-assembly checkpoint complex, which delays the onset of anaphase in cells with defects in mitotic spindle assembly; forms a complex with Mad1p |
| *MAK11* | *YKL021C* | *AFUA_4G07570* | 60S ribosome biogenesis protein Mak11 |
| *MEC1* | *YBR136W* | *AFUA_4G04760* | Genome integrity checkpoint protein and PI kinase superfamily member; signal transducer required for cell cycle arrest and transcriptional responses prompted by damaged or unreplicated DNA; monitors and participates in meiotic recombination |
| *MIH1* | *YMR036C* | *AFUA_6G08200* | Cell cycle control protein tyrosine phosphatase Mih1, putative |
| *MNN2* | *YBR015C YJL186W* | *AFUA_5G13090 AFUA_6G04450* | Alpha-1,2-mannosyltransferase, responsible for addition of alpha-1,2-linked mannose of the branches on the mannan backbone of oligosaccharides, localizes to an early Golgi compartment |
| *MPS1* | *YDL028C* | *AFUA_3G08100* | Dual-specificity kinase required for spindle pole body duplication and spindle checkpoint function; substrates include SPB proteins Spc42p, Spc110p, and Spc98p, mitotic exit network protein Mob1p, and checkpoint protein Mad1p |
| *MSB1* | *YOR188W* | *AFUA_3G08500* | Morphogenesis protein (Msb1) |
| *MUB1* | *YMR100W* | *AFUA_5G12230* | MYND domain protein (SamB) |
| *NDT80* | *YHR124W* | *AFUA_2G09890* | Meiosis-specific transcription factor required for exit from pachytene and for full meiotic recombination; activates middle sporulation genes; competes with Sum1p for binding to promoters containing middle sporulation elements (MSE) |
| *NFI1* | *YOR156C YDR409W* | *AFUA_6G05240* | SUMO/Smt3 ligase that promotes the attachment of sumo to proteins; binds Ubc9p and may bind septins; specifically required for sumoylation of septins in vivo; localized to the septin ring |
| *NUM1* | *YDR150W* | *AFUA_5G07710* | Nuclear migration protein (ApsA) |
| *OPT1* | *YJL212C* | *AFUA_2G15240 AFUA_6G10050 AFUA_5G13850 AFUA_6G10220* | Small oligopeptide transporter, OPT family |
| *OPY2* | *YPR075C* | *AFUA_3G14410* | Integral membrane protein that functions in the signaling branch of the high-osmolarity glycerol pathway; interacts with Ste50p; overproduction blocks cell cycle arrest in the presence of mating pheromone |
| *PAM1* | *YPL032C YDR251W* | *AFUA_6G02620* | Essential protein of unknown function; exhibits variable expression during colony morphogenesis; overexpression permits survival without protein phosphatase 2A, inhibits growth, and induces a filamentous phenotype |
| *PAP2* | *YOL115W YNL299W* | *AFUA_7G04130* | Non-canonical poly(A) polymerase, involved in nuclear RNA degradation as a component of the TRAMP complex; catalyzes polyadenylation of hypomodified tRNAs, and snoRNA and rRNA precursors; overlapping but non-redundant functions with Pap2p |
| *PCL1* | *YNL289W YDL179W YDL127W* | *AFUA_1G04750* | Cyclin, interacts with cyclin-dependent kinase Pho85p; member of the Pcl1,2-like subfamily, involved in the regulation of polarized growth and morphogenesis and progression through the cell cycle; localizes to sites of polarized cell growth |
| *PDS5* | *YMR076C* | *AFUA_2G16850* | Sister chromatid cohesion and DNA repair protein (BimD) |
| *PHB1* | *YGR132C* | *AFUA_1G13470* | Prohibitin complex subunit Phb1 |
| *PHB2* | *YGR231C* | *AFUA_2G09090* | Prohibitin |
| *PLP2* | *YOR281C* | *AFUA_2G02550* | Essential protein that interacts with the CCT complex to stimulate actin folding; has similarity to phosducins; null mutant lethality is complemented by mouse phosducin-like protein MgcPhLP |
| *PPH21* | *YDL188C YDL134C* | *AFUA_6G10830* | Catalytic subunit of protein phosphatase 2A (PP2A), functionally redundant with Pph22p; methylated at C terminus; forms alternate complexes with several regulatory subunits; involved in signal transduction and regulation of mitosis |
| *PPH3* | *YDR075W* | *AFUA_5G12010* | Catalytic subunit of an evolutionarily conserved protein phosphatase complex containing Psy2p and the regulatory subunit Psy4p; required for cisplatin resistance; involved in activation of Gln3p |
| *PPS1* | *YBR276C* | *AFUA_5G11690* | Protein tyrosine phosphatase Pps1 |
| *PRS1* | *YKL181W* | *AFUA_7G05670* | 5-phospho-ribosyl-1(alpha)-pyrophosphate synthetase, synthesizes PRPP, which is required for nucleotide, histidine, and tryptophan biosynthesis; one of five related enzymes, which are active as heteromultimeric complexes |
| *PTP3* | *YER075C* | *AFUA_4G04710* | Phosphotyrosine-specific protein phosphatase involved in the inactivation of mitogen-activated protein kinase during osmolarity sensing; dephosporylates Hog1p MAPK and regulates its localization; localized to the cytoplasm |
| *RAD24* | *YER173W* | *AFUA_8G02820* | Checkpoint protein, involved in the activation of the DNA damage and meiotic pachytene checkpoints; subunit of a clamp loader that loads Rad17p-Mec3p-Ddc1p onto DNA; homolog of human and S. pombe Rad17 protein |
| *RCK1* | *YGL158W YLR248W* | *AFUA_2G03490* | Protein kinase involved in the response to oxidative and osmotic stress; identified as suppressor of S. pombe cell cycle checkpoint mutations |
| *REX4* | *YOL080C* | *AFUA_2G05560* | Putative RNA exonuclease possibly involved in pre-rRNA processing and ribosome assembly |
| *RGP1* | *YDR137W* | *AFUA_2G12380* | Intracellular protein transport protein (Sat1), putative |
| *RHO3* | *YIL118W* | *AFUA_3G06690* | Non-essential small GTPase of the Rho/Rac subfamily of Ras-like proteins involved in the establishment of cell polarity; GTPase activity positively regulated by the GTPase activating protein Rgd1p |
| *SAP185* | *YJL098W YKR028W* | *AFUA_4G11012* | Protein that forms a complex with the Sit4p protein phosphatase and is required for its function; member of a family of similar proteins including Sap4p, Sap155p, and Sap190p |
| *SBE2* | *YHR103W YDR351W* | *AFUA_7G04580* | Protein involved in the transport of cell wall components from the Golgi to the cell surface; similar in structure and functionally redundant with Sbe2p; involved in bud growth |
| *SCH9* | *YHR205W* | *AFUA_1G06400* | Protein kinase involved in transcriptional activation of osmostress-responsive genes; regulates G1 progression, cAPK activity, nitrogen activation of the FGM pathway; involved in life span regulation; homologous to mammalian Akt/PKB |
| *SGV1* | *YPR161C* | *AFUA_5G05510* | Cyclin (Bur2p)-dependent protein kinase that functions in transcriptional regulation; phosphorylates the carboxy-terminal domain of Rpo21p and the C-terminal repeat domain of Spt5p; regulated by Cak1p |
| *SIT4* | *YDL047W* | *AFUA_6G11470* | TOR signaling pathway phosphatase SitA |
| *SNZ1* | *YMR096W YFL059W YNL333W* | *AFUA_5G08090* | Member of a stationary phase-induced gene family; transcription of SNZ2 is induced prior to diauxic shift, and also in the absence of thiamin in a Thi2p-dependent manner; forms a coregulated gene pair with SNO2; interacts with Thi11p |
| *SPO14* | *YKR031C* | *AFUA_3G05630* | Phospholipase D, catalyzes the hydrolysis of phosphatidylcholine, producing choline and phosphatidic acid; involved in Sec14p-independent secretion; required for meiosis and spore formation; differently regulated in secretion and meiosis |
| *SPO7* | *YAL009W* | *AFUA_1G03910* | Putative regulatory subunit of Nem1p-Spo7p phosphatase holoenzyme, regulates nuclear growth by controlling phospholipid biosynthesis, required for normal nuclear envelope morphology, premeiotic replication, and sporulation |
| *SPS1* | *YDR523C* | *AFUA_5G02220 AFUA_7G04330* | Putative protein serine/threonine kinase expressed at the end of meiosis and localized to the prospore membrane, required for correct localization of enzymes involved in spore wall synthesis |
| *SSF1* | *YHR066W YDR312W* | *AFUA_1G06230* | Constituent of 66S pre-ribosomal particles, required for ribosomal large subunit maturation; functionally redundant with Ssf2p; member of the Brix family |
| *SUR4* | *YLR372W* | *AFUA_5G02760* | Elongase, involved in fatty acid and sphingolipid biosynthesis; synthesizes very long chain 20-26-carbon fatty acids from C18-CoA primers; involved in regulation of sphingolipid biosynthesis |
| *SWE1* | *YJL187C* | *AFUA_2G07690* | Protein kinase that regulates the G2/M transition by inhibition of Cdc28p kinase activity; localizes to the nucleus and to the daughter side of the mother-bud neck; homolog of S. pombe Wee1p; potential Cdc28p substrate |
| *TOM1* | *YDR457W* | *AFUA_4G10780* | E3 ubiquitin ligase of the hect-domain class; has a role in mRNA export from the nucleus and may regulate transcriptional coactivators; involved in degradation of excess histones |
| *ULP2* | *YIL031W* | *AFUA_5G03200* | Peptidase that deconjugates Smt3/SUMO-1 peptides from proteins, plays a role in chromosome cohesion at centromeric regions and recovery from checkpoint arrest induced by DNA damage or DNA replication defects; potential Cdc28p substrate |
| *WHI2* | *YOR043W* | *AFUA_4G06130* | Protein required, with binding partner Psr1p, for full activation of the general stress response, possibly through Msn2p dephosphorylation; regulates growth during the diauxic shift; negative regulator of G1 cyclin expression |
| *WHI3* | *YDL224C YNL197C* | *AFUA_5G08330* | RNA binding protein that sequesters CLN3 mRNA in cytoplasmic foci; cytoplasmic retention factor for Cdc28p and associated cyclins; regulates cell fate and dose-dependently regulates the critical cell size required for passage through Start |
| *YAR1* | *YPL239W* | *AFUA_5G09560* | Cytoplasmic ankyrin-repeat containing protein of unknown function, proposed to link the processes of 40S ribosomal subunit biogenesis and adaptation to osmotic and oxidative stress; expression repressed by heat shock |
| *YPK1* | *YMR104C YKL126W* | *AFUA_2G10620* | Protein kinase with similarity to serine/threonine protein kinase Ypk1p; functionally redundant with YPK1 at the genetic level; participates in a signaling pathway required for optimal cell wall integrity; homolog of mammalian kinase SGK |
| *ZDS1* | *YML109W YMR273C* | *AFUA_6G07890* | Protein that interacts with silencing proteins at the telomere, involved in transcriptional silencing; has a role in localization of PKA subunit Bcy1p; implicated in mRNA nuclear export; involved in mitotic exit through regulation of Cdc14p |
|  | *YNL320W* | *AFUA_7G04660* | Putative protein of unknown function; the authentic, non-tagged protein is detected in highly purified mitochondria in high-throughput studies |
|  | *YMR262W* | *AFUA_1G03870* | Protein of unknown function; interacts weakly with Knr4p; YMR262W is not an essential gene |
| *CDC37* | *YDR168W* | *AFUA_4G10010* | Hsp90 co-chaperone Cdc37 |
| *ERJ5* | *YFR041C* | *AFUA_6G06610* | Endoplasmic reticulum DnaJ domain protein Erj5, putative |
| *FPR3* | *YLR449W YML074C* | *AFUA_6G08580* | FKBP-type peptidyl-prolyl isomerase |
| *GIM3* | *YNL153C* | *AFUA_1G15240* | Prefoldin subunit 4 |
| *GIM5* | *YML094W* | *AFUA_1G10740* | Prefoldin subunit 5 |
| *HLJ1* | *YMR161W* | *AFUA_4G07330* | Co-chaperone for Hsp40p, anchored in the ER membrane; with its homolog Ydj1p promotes ER-associated protein degradation of integral membrane substrates; similar to E. coli DnaJ |
| *HSP26* | *YBR072W YDR171W* | *AFUA_5G06820* | Hsp26p |
| *JJJ1* | *YNL227C* | *AFUA_3G05320* | Co-chaperone that stimulates the ATPase activity of Ssa1p, required for a late step of ribosome biogenesis; associated with the cytosolic large ribosomal subunit; contains a J-domain; mutation causes defects in fluid-phase endocytosis |
| *JJJ3* | *YJR097W* | *AFUA_4G09100* | DnaJ domain protein |
| *PFD1* | *YJL179W* | *AFUA_4G12360* | Prefoldin subunit 1 |
| *SBA1* | *YKL117W* | *AFUA_5G13920* | Co-chaperone that binds to and regulates Hsp90 family chaperones; important for pp60v-src activity in yeast; homologous to the mammalian p23 proteins and like p23 can regulate telomerase activity |
| *DIT2* | *YDR402C* | *AFUA_5G02620* | P450 family sporulation-specific N-formyltyrosine oxidase Dit2 |
| *JLP1* | *YLL057C* | *AFUA_3G07960 AFUA_8G02210* | Fe(II)-dependent sulfonate/alpha-ketoglutarate dioxygenase, involved in sulfonate catabolism for use as a sulfur source; contains sequence that resembles a J domain (typified by the E. coli DnaJ protein); induced by sulphur starvation |
| *ARE1* | *YCR048W YNR019W* | *AFUA_1G06040* | Acyl-CoA:sterol acyltransferase, isozyme of Are2p; endoplasmic reticulum enzyme that contributes the major sterol esterification activity in the absence of oxygen |
| *CDA1* | *YLR307W YLR308W* | *AFUA_3G07210 AFUA_4G09940 AFUA_6G10430* | Polysaccharide deacetylase family protein |
| *DIT1* | *YDR403W* | *AFUA_3G13690 AFUA_4G01420 AFUA_4G01370 AFUA_5G02660* | Pyoverdine/dityrosine biosynthesis family protein, putative |
| *LRG1* | *YDL240W* | *AFUA_2G15050* | Rho GTPase activator (Lrg11) |
| *MCD4* | *YKL165C* | *AFUA_4G03970* | GPI-anchor biosynthetic protein (Mcd4) |
| *MDS3* | *YER132C YGL197W* | *AFUA_4G08570* | Regulatory protein Ral2 |
| *RIM9* | *YMR063W* | *AFUA_3G07530* | Protein of unknown function, involved in the proteolytic activation of Rim101p in response to alkaline pH; has similarity to A. nidulans PalI; putative membrane protein |
| *CCH1* | *YGR217W* | *AFUA_1G11110* | Calcium channel subunit Cch1 |
| *CHO1* | *YER026C* | *AFUA_4G13680* | Phosphatidylserine synthase |
| *CRZ1* | *YNL027W* | *AFUA_1G06900* | C2H2 transcription factor Crz1 |
| *ENA1* | *YDR038C YDR039C YDR040C* | *AFUA_4G09440 AFUA_6G03690* | Sodium P-type ATPase |
| *ERG1* | *YGR175C* | *AFUA_5G07780* | Squalene monooxygenase Erg1 |
| *ERG6* | *YML008C* | *AFUA_4G03630* | Delta(24)-sterol C-methyltransferase, converts zymosterol to fecosterol in the ergosterol biosynthetic pathway by methylating position C-24; localized to both lipid particles and mitochondrial outer membrane |
| *HAM1* | *YJR069C* | *AFUA_5G05560* | Conserved protein with deoxyribonucleoside triphosphate pyrophosphohydrolase activity, mediates exclusion of noncanonical purines from deoxyribonucleoside triphosphate pools; mutant is sensitive to the base analog 6-N-hydroxylaminopurine |
| *KHA1* | *YJL094C* | *AFUA_8G06920* | K+ homeostasis protein Kha1 |
| *KTI12* | *YKL110C* | *AFUA_3G14100* | RNA polymerase II Elongator complex associated protein Kti12 |
| *NHA1* | *YLR138W* | *AFUA_2G17170* | Na+/H+ antiporter involved in sodium and potassium efflux through the plasma membrane; required for alkali cation tolerance at acidic pH |
| *NHX1* | *YDR456W* | *AFUA_5G06220* | Endosomal Na+/H+ exchanger, required for intracellular sequestration of Na+; required for osmotolerance to acute hypertonic shock |
| *PHO89* | *YBR296C* | *AFUA_3G03010* | Na+/Pi cotransporter, active in early growth phase; similar to phosphate transporters of Neurospora crassa; transcription regulated by inorganic phosphate concentrations and Pho4p |
| *PHO91* | *YNR013C* | *AFUA_1G04290* | Low-affinity phosphate transporter of the vacuolar membrane; deletion of pho84, pho87, pho89, pho90, and pho91 causes synthetic lethality; transcription independent of Pi and Pho4p activity; overexpression results in vigorous growth |
| *PIC2* | *YER053C* | *AFUA_2G12080* | Mitochondrial phosphate carrier, imports inorganic phosphate into mitochondria; functionally redundant with Mir1p but less abundant than Mir1p under normal conditions; expression is induced at high temperature |
| *PUG1* | *YLR046C YER185W YGR213C* | *AFUA_2G10840 AFUA_7G01970 AFUA_5G09900 AFUA_8G05740 AFUA_3G01030 AFUA_3G03310 AFUA_6G09550 AFUA_5G01310 AFUA_6G11800 AFUA_6G11810* | Membrane protein; member of the fungal lipid-translocating exporter family is |
| *QRI7* | *YDL104C* | *AFUA_7G05240* | Highly conserved mitochondrial protein, essential for stability of the mitochondrial genome; functional homolog of the essential E. coli ygjD gene; member of a family of proteins putatively involved in genome maintenance |
| *SLC1* | *YDL052C* | *AFUA_2G08600* | 1-acyl-sn-glycerol-3-phosphate acyltransferase, catalyzes the acylation of lysophosphatidic acid to form phosphatidic acid, a key intermediate in lipid metabolism; enzymatic activity detected in lipid particles and microsomes |
| *SPF1* | *YEL031W* | *AFUA_3G13790* | P-type ATPase, ion transporter of the ER membrane involved in ER function and Ca2+ homeostasis; required for regulating Hmg2p degradation; confers sensitivity to a killer toxin produced by Pichia farinosa KK1 |
| *SUL1* | *YBR294W YLR092W* | *AFUA_1G05020* | High affinity sulfate permease; sulfate uptake is mediated by specific sulfate transporters Sul1p and Sul2p, which control the concentration of endogenous activated sulfate intermediates |
|  | *YDR415C* | *AFUA_4G04210* | Putative protein of unknown function |
|  | *YPR003C* | *AFUA_3G13470* | Putative sulfate permease; physically interacts with Hsp82p; green fluorescent protein (GFP)-fusion protein localizes to the ER; YPR003C is not an essential gene |
|  | *YBR235W* | *AFUA_4G07770* | Putative ion transporter, similar to mammalian electroneutral Na(+)-(K+)-C1- cotransporter family; YBR235W is not an essential gene |
| *MAK32* | *YCR019W* | *AFUA_1G12750 AFUA_4G09500* | PfkB family carbohydrate kinase (Mak32) |
| *MKT1* | *YNL085W* | *AFUA_5G12250* | Posttranscriptional regulation nuclease (Mkt1), putative |
| *AAC1* | *YMR056C YBL030C YBR085W* | *AFUA_1G05390* | Major ADP/ATP carrier of the mitochondrial inner membrane, exchanges cytosolic ADP for mitochondrially synthesized ATP; also imports heme and ATP; phosphorylated; required for viability in many lab strains that carry a sal1 mutation |
| *AAT2* | *YLR027C* | *AFUA_2G09650* | Aspartate transaminase |
| *ABC1* | *YGL119W* | *AFUA_6G04380* | Molecular chaperone (ABC1) |
| *ABD1* | *YBR236C* | *AFUA_6G07690* | MRNA cap methyltransferase |
| *ABP1* | *YCR088W* | *AFUA_8G02850* | Actin binding protein |
| *ABP140* | *YOR239W* | *AFUA_6G07150* | Actin binding protein |
| *ABZ2* | *YMR289W* | *AFUA_2G01650* | Abz2p |
| *ACC1* | *YNR016C* | *AFUA_2G08670* | Acetyl-CoA carboxylase |
| *ACE2* | *YLR131C YDR146C* | *AFUA_3G11250* | C2H2 transcription factor (Swi5) |
| *ACF2* | *YLR144C YNR067C* | *AFUA_1G04260* | Daughter cell-specific secreted protein with similarity to glucanases, degrades cell wall from the daughter side causing daughter to separate from mother |
| *ACH1* | *YBL015W* | *AFUA_8G05580* | Acetyl-coA hydrolase Ach1 |
| *ACN9* | *YDR511W* | *AFUA_2G05090* | ACN9 family domain containing protein |
| *ACO1* | *YLR304C* | *AFUA_6G12930* | Mitochondrial aconitate hydratase |
| *ACP1* | *YKL192C* | *AFUA_1G06620* | Acyl carrier protein |
| *ACT1* | *YFL039C* | *AFUA_6G04740* | Actin Act1 |
| *ADA2* | *YDR448W* | *AFUA_2G10640* | SAGA complex subunit (Ada2) |
| *ADE12* | *YNL220W* | *AFUA_1G15450* | Adenylosuccinate synthetase AdB |
| *ADE3* | *YBR084W YGR204W* | *AFUA_3G08650* | C1 tetrahydrofolate synthase |
| *ADE6* | *YGR061C* | *AFUA_5G02720* | Phosphoribosylformylglycinamidine synthase |
| *ADE8* | *YDR408C* | *AFUA_2G10855* | Phosphoribosylglycinamide formyltransferase |
| *ADH4* | *YGL256W* | *AFUA_2G04520* | Fe-containing alcohol dehydrogenase |
| *ADI1* | *YMR009W* | *AFUA_6G04430* | Acireductone dioxygenease involved in the methionine salvage pathway; ortholog of human MTCBP-1; transcribed with YMR010W and regulated post-transcriptionally by RNase III cleavage; ADI1 mRNA is induced in heat shock conditions |
| *ADK1* | *YDR226W* | *AFUA_1G07530* | Adenylate kinase, required for purine metabolism; localized to the cytoplasm and the mitochondria; lacks cleavable signal sequence |
| *ADK2* | *YER170W* | *AFUA_1G03420* | Adenylate kinase 2 |
| *ADP1* | *YCR011C* | *AFUA_6G07280* | ABC transporter (Adp1) |
| *AFG1* | *YEL052W* | *AFUA_4G04130* | Mitochondrial ATPase (Afg1) |
| *AGE1* | *YDR524C* | *AFUA_7G03790* | ADP-ribosylation factor GTPase activating protein effector, involved in the secretory and endocytic pathways; contains C2C2H2 cysteine/histidine motif |
| *AGE2* | *YIL044C* | *AFUA_6G07830* | ADP-ribosylation factor GTPase activating protein effector, involved in Trans-Golgi-Network transport; contains C2C2H2 cysteine/histidine motif |
| *AHA1* | *YDR214W* | *AFUA_4G11330* | Aha1 domain family |
| *AHC1* | *YOR023C* | *AFUA_1G09530* | Ahc1p |
| *AHP1* | *YLR109W* | *AFUA_5G01440 AFUA_6G02280* | Allergen Asp F3 |
| *AIF1* | *YNR074C* | *AFUA_3G01290 AFUA_1G17180 AFUA_2G00230* | Pyridine nucleotide-disulphide oxidoreductase AMID-like |
| *AIM14* | *YGL160W YLR047C* | *AFUA_3G02980* | Metalloreductase Fre8 |
| *AIM17* | *YHL021C* | *AFUA_1G06180 AFUA_2G14970* | Gamma-butyrobetaine hydroxylase subfamily |
| *AIM18* | *YHR198C YHR199C* | *AFUA_1G06110* | Aim18p |
| *AIM2* | *YAL049C* | *AFUA_4G01130 AFUA_6G12740* | Dienelactone hydrolase family protein |
| *AIM22* | *YJL046W* | *AFUA_1G06950* | Lipoyltransferase and lipoate-protein ligase |
| *AIM24* | *YJR080C* | *AFUA_6G12860* | Mitochondrial protein Fmp26 |
| *AIM25* | *YJR100C* | *AFUA_5G04050* | Scramblase family protein |
| *AIM27* | *YKL207W* | *AFUA_8G05350* | DUF850 domain protein |
| *AIM3* | *YBR108W* | *AFUA_8G05290* | Aim3p |
| *AIM31* | *YML030W* | *AFUA_4G08130* | Mitochondrial hypoxia responsive domain protein |
| *AIM38* | *YNR018W* | *AFUA_1G12250* | Mitochondrial hypoxia responsive domain protein |
| *AIM45* | *YPR004C* | *AFUA_7G05470* | Electron transfer flavoprotein alpha subunit |
| *AIM6* | *YDL237W* | *AFUA_5G09530 AFUA_2G16060 AFUA_4G14200 AFUA_8G06060* | Aim6p |
| *AIM9* | *YER080W* | *AFUA_1G00750 AFUA_4G00650 AFUA_5G14880 AFUA_8G05975 AFUA_4G09260 AFUA_3G15220 AFUA_6G11540* | Phosphotransferase enzyme family protein |
| *AIP1* | *YMR092C* | *AFUA_2G06040* | Actin cortical patch component, interacts with the actin depolymerizing factor cofilin; required to restrict cofilin localization to cortical patches; contains WD repeats |
| *AIR1* | *YIL079C YDL175C* | *AFUA_4G13280* | Zinc knuckle domain protein |
| *ALA1* | *YOR335C* | *AFUA_8G03880* | Alanyl-tRNA synthetase |
| *ALD4* | *YER073W YOR374W* | *AFUA_6G11430 AFUA_7G01000* | Aldehyde dehydrogenase AldA |
| *ALE1* | *YOR175C* | *AFUA_6G04860* | MBOAT family protein |
| *ALF1* | *YNL148C* | *AFUA_1G07580* | Cell polarity protein (Alp11) |
| *ALG1* | *YBR110W* | *AFUA_6G14180* | Beta-1,4-mannosyltransferase (Alg1) |
| *ALG13* | *YGL047W* | *AFUA_4G13400* | Glycosyltransferase family 28 |
| *ALG14* | *YBR070C* | *AFUA_6G06940* | Glycosyltransferase family protein |
| *ALG3* | *YBL082C* | *AFUA_5G11990* | Alpha-1,3-mannosyltransferase (Alg3) |
| *ALG5* | *YPL227C* | *AFUA_5G08210* | Dolichyl-phosphate beta-glucosyltransferase |
| *ALG7* | *YBR243C* | *AFUA_2G11240* | UDP-N-acetyl-glucosamine-1-P transferase Alg7 |
| *ALG8* | *YOR067C* | *AFUA_2G16790* | Glucosyltransferase |
| *ALP1* | *YNL268W YEL063C YNL270C* | *AFUA_3G01560 AFUA_5G04260* | Amino acid permease (Can1) |
| *ALR1* | *YFL050C YOL130W* | *AFUA_5G05830* | CorA family metal ion transporter |
| *ALY1* | *YJL084C YKR021W* | *AFUA_1G12020* | Arrestin domain protein |
| *AMS1* | *YGL156W* | *AFUA_3G08200* | Alpha-mannosidase |
| *ANB1* | *YEL034W YJR047C* | *AFUA_1G04070* | Eukaryotic translation initiation factor eIF-5A |
| *ANP1* | *YEL036C* | *AFUA_2G15910* | Mannan polymerase II complex ANP1 subunit Anp1, putative |
| *ANT1* | *YPR128C* | *AFUA_1G03440* | Peroxisomal carrier protein |
| *APC1* | *YNL172W* | *AFUA_3G05950* | 20S cyclosome subunit (APC1/BimE) |
| *APC11* | *YDL008W* | *AFUA_7G01380* | Anaphase promoting complex subunit Apc11 |
| *APD1* | *YBR151W* | *AFUA_3G09110 AFUA_4G11477* | Sucrase/ferredoxin-like family protein |
| *APE3* | *YBR286W* | *AFUA_2G00220* | Aminopeptidase |
| *APL6* | *YGR261C* | *AFUA_5G11360* | AP-3 adaptor complex subunit beta |
| *APN1* | *YKL114C* | *AFUA_6G08110* | DNA lyase, endon uclease |
| *APP1* | *YNL094W* | *AFUA_6G10890* | Actin cytoskeleton organization protein App1 |
| *APT1* | *YLR118C* | *AFUA_6G02780* | Acyl-protein thioesterase responsible for depalmitoylation of Gpa1p; green fluorescent protein (GFP)-fusion protein localizes to both the cytoplasm and nucleus and is induced in response to the DNA-damaging agent MMS |
| *AQR1* | *YNL065W YIL121W YIL120W* | *AFUA_3G03190 AFUA_3G15380 AFUA_2G15320 AFUA_4G11060 AFUA_3G01940 AFUA_5G01520* | MFS multidrug transporter |
| *ARA2* | *YMR041C* | *AFUA_7G04140* | L-galactose dehydrogenase (L-GalDH) |
| *ARC1* | *YGL105W* | *AFUA_2G15940* | Cofactor for methionyl- and glutamyl-tRNA synthetases, putative |
| *ARC15* | *YIL062C* | *AFUA_3G10700* | Arp2/3 complex subunit Arc16 |
| *ARC18* | *YLR370C* | *AFUA_5G01860* | ARP2/3 complex subunit Arc18 |
| *ARC19* | *YKL013C* | *AFUA_6G02370* | ARP2/3 complex 20 kDa subunit (p20-ARC) |
| *ARC35* | *YNR035C* | *AFUA_1G02670* | ARP2/3 complex 34 kDa subunit |
| *ARC40* | *YBR234C* | *AFUA_6G06500* | Actin-related protein 2/3 complex subunit 1A |
| *ARD1* | *YHR013C* | *AFUA_1G09600* | N-acetyltransferase complex ARD1 subunit |
| *ARG1* | *YOL058W* | *AFUA_2G04310* | Argininosuccinate synthase |
| *ARG3* | *YJL088W* | *AFUA_4G07190* | Ornithine carbamoyltransferase |
| *ARG4* | *YHR018C* | *AFUA_3G07790* | Argininosuccinate lyase |
| *ARG5,6* | *YER069W* | *AFUA_6G02910* | Acetylglutamate kinase |
| *ARG7* | *YMR062C* | *AFUA_5G08120* | Arginine biosynthesis bifunctional protein ArgJ |
| *ARG8* | *YOL140W* | *AFUA_2G12470* | Acetylornithine aminotransferase, catalyzes the fourth step in the biosynthesis of the arginine precursor ornithine |
| *ARG81* | *YML099C* | *AFUA_8G02280* | C6 transcription factor |
| *ARG82* | *YDR173C* | *AFUA_5G11450* | Arginine metabolism regulation protein iii |
| *ARH1* | *YDR376W* | *AFUA_2G12610* | NADPH-adrenodoxin reductase Arh1 |
| *ARL1* | *YBR164C* | *AFUA_2G10980* | ADP-ribosylation factor |
| *ARO1* | *YDR127W* | *AFUA_1G13740* | Pentafunctional polypeptide (AroM) |
| *ARO2* | *YGL148W* | *AFUA_1G06940* | Chorismate synthase |
| *ARO3* | *YDR035W* | *AFUA_1G02110* | 3-deoxy-D-arabino-heptulosonate-7-phosphate synthase, catalyzes the first step in aromatic amino acid biosynthesis and is feedback-inhibited by phenylalanine or high concentration of tyrosine or tryptophan |
| *ARO4* | *YBR249C* | *AFUA_7G04070* | 3-deoxy-D-arabino-heptulosonate-7-phosphate synthase, catalyzes the first step in aromatic amino acid biosynthesis and is feedback-inhibited by tyrosine or high concentrations of phenylalanine or tryptophan |
| *ARO7* | *YPR060C* | *AFUA_5G13130* | Chorismate mutase |
| *ARO80* | *YDR421W* | *AFUA_5G13310 AFUA_2G00100 AFUA_2G04262* | C6 transcription factor Aro80 |
| *ARP2* | *YDL029W* | *AFUA_1G13330* | Arp2/3 complex subunit (Arp2) |
| *ARP3* | *YJR065C* | *AFUA_5G11560* | Arp2/3 complex subunit (Arp3) |
| *ARP4* | *YJL081C* | *AFUA_2G06110* | Chromatin remodeling and histone acetyltransferase complexes subunit putative |
| *ARP5* | *YNL059C* | *AFUA_4G03020* | Chromatin remodeling complex subunit (Arp5) |
| *ARP6* | *YLR085C* | *AFUA_4G04420* | Actin-related protein that binds nucleosomes; a component of the SWR1 complex, which exchanges histone variant H2AZ for chromatin-bound histone H2A |
| *ARP8* | *YOR141C* | *AFUA_2G10990* | Chromatin remodeling complex subunit (Arp8) |
| *ARP9* | *YMR033W* | *AFUA_3G02330* | Chromatin remodeling complex subunit (Arp9) |
| *ARR3* | *YPR201W* | *AFUA_1G05760 AFUA_1G16100 AFUA_5G15010* | Arsenite efflux transporter ArsB, puative |
| *ARV1* | *YLR242C* | *AFUA_2G11480* | Arv1p |
| *ARX1* | *YDR101C* | *AFUA_2G16820* | Curved DNA-binding protein (42 kDa protein) |
| *ASC1* | *YMR116C* | *AFUA_4G13170* | G-protein complex beta subunit CpcB |
| *ASE1* | *YOR058C* | *AFUA_2G16260* | Microtubule associated protein (Ase1) |
| *ASI1* | *YMR119W YNL008C* | *AFUA_6G10590* | Ubiquitin-protein ligase (Asi3) |
| *ASK1* | *YKL052C* | *AFUA_1G12670* | Ask1p |
| *ASR1* | *YPR093C* | *AFUA_2G16870* | PHD and RING finger domain protein |
| *ATE1* | *YGL017W* | *AFUA_2G13070* | Arginine-tRNA-protein transferase 1 |
| *ATG1* | *YGL180W* | *AFUA_4G09050* | Serine/threonine protein kinase (Pdd7p) |
| *ATG12* | *YBR217W* | *AFUA_6G09165* | Autophagy protein Apg12 |
| *ATG15* | *YCR068W* | *AFUA_2G10900* | Autophagy related lipase Atg15 |
| *ATG16* | *YMR159C* | *AFUA_5G12120* | Autophagy protein Apg16 |
| *ATG17* | *YLR423C* | *AFUA_2G14100* | Kinase activator (Atg17) |
| *ATG18* | *YFR021W* | *AFUA_5G11660* | Protein-vacuolar targeting protein Atg18 |
| *ATG2* | *YNL242W* | *AFUA_6G13200* | Autophagy regulatory protein Atg2 |
| *ATG26* | *YLR189C* | *AFUA_2G02220* | UDP-glucose:sterol glycosyltransferase |
| *ATG4* | *YNL223W* | *AFUA_3G05340* | Autophagy cysteine endopeptidase Atg4 |
| *ATG5* | *YPL149W* | *AFUA_6G07040* | Autophagy protein Apg5 |
| *ATG7* | *YHR171W* | *AFUA_2G06250* | Autophagy ubiquitin-activating enzyme ApgG |
| *ATG8* | *YBL078C* | *AFUA_1G07470* | Autophagic death protein Aut7/IDI-7 |
| *ATG9* | *YDL149W* | *AFUA_6G12350* | Autophagy protein Apg9 |
| *ATH1* | *YPR026W* | *AFUA_3G02280* | Acid trehalase required for utilization of extracellular trehalose |
| *ATM1* | *YMR301C* | *AFUA_6G12870* | ABC iron exporter Atm1 |
| *ATP1* | *YBL099W* | *AFUA_8G05320* | Alpha subunit of the F1 sector of mitochondrial F1F0 ATP synthase, which is a large, evolutionarily conserved enzyme complex required for ATP synthesis; phosphorylated |
| *ATP10* | *YLR393W* | *AFUA_1G10490* | F1F0 ATP synthase assembly protein Atp10 |
| *ATP11* | *YNL315C* | *AFUA_7G04670* | F1F0 ATP synthase assembly protein Atp11 |
| *ATP12* | *YJL180C* | *AFUA_7G02490* | Mitochondrial molecular chaperone (Atp12) |
| *ATP16* | *YDL004W* | *AFUA_1G03100* | ATP synthase delta chain, mitochondrial precursor, putative |
| *ATP17* | *YDR377W* | *AFUA_2G05510* | Mitochondrial F1F0 ATP synthase subunit F (Atp17), putative |
| *ATP18* | *YML081C-A* | *AFUA_2G02275* | Mitochondrial F1F0 ATP synthase subunit Atp18 |
| *ATP2* | *YJR121W* | *AFUA_5G10550* | ATP synthase F1, beta subunit |
| *ATP20* | *YPR020W* | *AFUA_1G16280* | Mitochondrial F1F0-ATP synthase g subunit |
| *ATP23* | *YNR020C* | *AFUA_2G07560* | Ku70-binding protein |
| *ATP25* | *YMR098C* | *AFUA_2G16740* | Atp25p |
| *ATP3* | *YBR039W* | *AFUA_1G03510* | ATP synthase gamma chain, mitochondrial precursor, putative |
| *ATP4* | *YPL078C* | *AFUA_8G05440* | Mitochondrial ATPase subunit ATP4 |
| *ATP5* | *YDR298C* | *AFUA_2G12400* | ATP synthase oligomycin sensitivity conferral protein, putative |
| *ATP7* | *YKL016C* | *AFUA_6G03810* | ATP synthase D chain, mitochondrial |
| *ATS1* | *YAL020C* | *AFUA_2G10700* | Alpha-tubulin suppressor protein Aats1 |
| *ATX1* | *YNL259C* | *AFUA_1G08880* | Iron/copper transporter Atx1 |
| *ATX2* | *YOR079C* | *AFUA_2G12050* | ZIP metal ion transporter |
| *AUR1* | *YKL004W* | *AFUA_3G09960* | Aureobasidin resistance protein Aur1 |
| *AVL9* | *YLR114C* | *AFUA_1G12950* | Avl9 protein |
| *AVO1* | *YOL078W* | *AFUA_2G12210* | Stress activated MAP kinase interacting protein, putative |
| *AVO2* | *YMR068W* | *AFUA_6G00580 AFUA_6G04960 AFUA_3G02830 AFUA_8G00260 AFUA_8G02140 AFUA_1G01020* | Domain and ankyrin repeat protein |
| *AXL2* | *YIL140W* | *AFUA_1G09270* | Transmembrane glycoprotein |
| *AYT1* | *YLL063C* | *AFUA_6G09620 AFUA_2G18020 AFUA_6G13990* | Acetyltransferase; catalyzes trichothecene 3-O-acetylation, suggesting a possible role in trichothecene biosynthesis |
| *AZF1* | *YOR113W* | *AFUA_6G05160* | C2H2 transcription factor (Azf1) |
| *AZR1* | *YPR198W YGR224W YCL069W YKR105C* | *AFUA_3G02520 AFUA_3G08530 AFUA_5G14490 AFUA_3G14720 AFUA_6G03320 AFUA_2G08230 AFUA_4G03920 AFUA_3G15250 AFUA_5G10340 AFUA_6G02220 AFUA_3G02720 AFUA_8G00940 AFUA_6G14640 AFUA_3G01520 AFUA_5G01540 AFUA_1G12620 AFUA_1G16910 AFUA_6G09710 AFUA_3G02110 AFUA_5G10140* | MFS multidrug transporter |
| *BAG7* | *YOR134W YDR389W* | *AFUA_2G01060* | GTPase activating protein for Rho1p, involved in signaling to the actin cytoskeleton, null mutations suppress tor2 mutations and temperature sensitive mutations in actin; potential Cdc28p substrate |
| *BAS1* | *YKR099W* | *AFUA_1G03210* | MYB family conidiophore development protein FlbD, putative |
| *BAT1* | *YHR208W YJR148W* | *AFUA_4G06160* | Branched-chain amino acid aminotransferase, cytosolic |
| *BBC1* | *YJL020C* | *AFUA_2G10320* | SH3 domain protein |
| *BCD1* | *YHR040W* | *AFUA_5G14010* | HIT finger domain protein |
| *BCK1* | *YJL095W* | *AFUA_3G11080* | MAP kinase kinase kinase (Bck1) |
| *BCP1* | *YDR361C* | *AFUA_5G13120* | Bcp1p |
| *BCS1* | *YDR375C* | *AFUA_3G13000* | Mitochondrial chaperone BCS1 |
| *BCY1* | *YIL033C* | *AFUA_3G10000* | CAMP-dependent protein kinase regulatory subunit PkaR |
| *BDP1* | *YNL039W* | *AFUA_6G04400* | Transcription factor TFIIIB component |
| *BEM1* | *YBR200W* | *AFUA_4G04120* | Protein kinase activator Bem1 |
| *BEM3* | *YPL115C* | *AFUA_6G06400* | Rho GTPase activator (Bem3) |
| *BET3* | *YKR068C* | *AFUA_7G02270* | TRAPP complex component Bet3 |
| *BET4* | *YJL031C* | *AFUA_4G10580* | Alpha subunit of Type II geranylgeranyltransferase required for vesicular transport between the endoplasmic reticulum and the Golgi; provides a membrane attachment moiety to Rab-like proteins Ypt1p and Sec4p |
| *BET5* | *YML077W* | *AFUA_5G05970* | TRAPP complex subunit (Bet5) |
| *BIK1* | *YCL029C* | *AFUA_8G04660* | Noc1p protein |
| *BIM1* | *YER016W* | *AFUA_3G11860* | Microtubule associated protein EB1 |
| *BIR1* | *YJR089W* | *AFUA_1G14070* | Chromosome segregation protein BIR1 |
| *BLM10* | *YFL007W* | *AFUA_4G11290* | Proteasome activator subunit 4 |
| *BMS1* | *YPL217C* | *AFUA_2G13570* | Ribosome biogenesis protein (Bms1) |
| *BNA6* | *YFR047C* | *AFUA_3G05730* | Nicotinate-nucleotide pyrophosphorylase |
| *BNA7* | *YDR428C* | *AFUA_1G09960* | Bna7p |
| *BNI1* | *YNL271C* | *AFUA_6G04940* | Cytokinesis protein SepA/Bni1 |
| *BNI4* | *YNL233W* | *AFUA_1G16680* | Bni4p |
| *BNI4* | *YNL233W* | *AFUA_1G14960* | Gelsolin repeat protein |
| *BOR1* | *YNL275W* | *AFUA_3G10880 AFUA_4G09170* | Anion exchange family protein |
| *BRE1* | *YDL074C* | *AFUA_6G04390* | Histone ubiquitinationc protein (Bre1) |
| *BRE2* | *YLR015W* | *AFUA_3G04120* | Histone-lysine N-methyltransferase (Bre2) |
| *BRE5* | *YNR051C* | *AFUA_5G04160 AFUA_6G11460* | Nonsense-mediated mRNA decay protein Upf3 |
| *BRF1* | *YGR246C* | *AFUA_3G12730* | Transcription factor TFIIIB complex subunit Brf1, putative |
| *BRR2* | *YER172C* | *AFUA_8G04740* | Pre-mRNA splicing helicase |
| *BRR6* | *YGL247W* | *AFUA_4G03180* | Nuclear envelope protein Brr6 |
| *BRX1* | *YOL077C* | *AFUA_1G02210* | 60S ribosome biogenesis protein Brx1 |
| *BSC6* | *YOL137W* | *AFUA_7G00280 AFUA_4G03590 AFUA_7G06830 AFUA_5G11980 AFUA_2G17270 AFUA_8G05090* | MFS transporter |
| *BSD2* | *YBR290W* | *AFUA_4G13740* | Metal homeostatis protein bsd2 |
| *BST1* | *YFL025C* | *AFUA_7G05540* | GPI maturation protein (Bst1) |
| *BUB1* | *YGR188C* | *AFUA_6G08120* | Checkpoint protein kinase (SldA) |
| *BUD13* | *YGL174W* | *AFUA_4G03160* | Cell cycle control protein (Cwf26) |
| *BUD14* | *YAR014C* | *AFUA_1G11920* | SH3 domain protein |
| *BUD20* | *YLR074C* | *AFUA_6G12920* | C2H2 finger domain protein |
| *BUD22* | *YMR014W* | *AFUA_6G08800* | Cellular morphogenesis protein (Bud22) |
| *BUD23* | *YCR047C* | *AFUA_6G06380* | Methyltransferase |
| *BUD27* | *YFL023W* | *AFUA_4G08910* | Bud27p |
| *BUD3* | *YCL014W* | *AFUA_5G11890* | Rho guanyl nucleotide exchange factor |
| *BUD31* | *YCR063W* | *AFUA_5G05610* | Cell cycle control protein Cwf14/Bud31 |
| *BUD32* | *YGR262C* | *AFUA_3G14290* | Protein kinase |
| *BUD6* | *YLR319C* | *AFUA_1G09640* | Actin- and formin-interacting protein, involved in actin cable nucleation and polarized cell growth; isolated as bipolar budding mutant; potential Cdc28p substrate |
| *BUR6* | *YER159C* | *AFUA_2G14250* | CBF/NF-Y family transcription factor |
| *BYE1* | *YKL005C* | *AFUA_6G09000* | PHD finger domain protein |
| *BZZ1* | *YHR114W* | *AFUA_2G01200* | Actin polymerization protein Bzz1 |
| *CAB1* | *YDR531W* | *AFUA_3G07180* | Pantothenate kinase |
| *CAB2* | *YIL083C* | *AFUA_3G07230* | Phosphopantothenate-cysteine ligase |
| *CAB3* | *YKL088W YOR054C YKR072C* | *AFUA_4G05960* | Negative regulatory subunit of protein phosphatase 1 Ppz1p and also a subunit of the phosphopantothenoylcysteine decarboxylase complex, which catalyzes the third step of coenzyme A biosynthesis |
| *CAB4* | *YGR277C* | *AFUA_4G08550* | Pantetheine-phosphate adenylyltransferase family protein |
| *CAB5* | *YDR196C* | *AFUA_5G02060* | Dephospho-CoA kinase |
| *CAC2* | *YML102W* | *AFUA_2G04030* | Chromatin assembly factor 1 subunit B |
| *CAD1* | *YML007W YDR423C* | *AFUA_2G14680 AFUA_6G09930* | AP-1-like basic leucine zipper transcriptional activator involved in stress responses, iron metabolism, and pleiotropic drug resistance; controls a set of genes involved in stabilizing proteins; binds consensus sequence TTACTAA |
| *CAF120* | *YLR187W YNL278W* | *AFUA_1G05130* | Protein of unknown function; green fluorescent protein (GFP)-fusion protein localizes to the cell periphery, cytoplasm, bud, and bud neck; potential Cdc28p substrate; similar to Caf120p and Skg4p |
| *CAF40* | *YNL288W* | *AFUA_4G12390* | Cell differentiation protein (Rcd1) |
| *CAM1* | *YPL048W YKL081W* | *AFUA_6G04570* | Translation elongation factor eEF-1 subunit gamma, putative |
| *CAP1* | *YKL007W* | *AFUA_6G10060* | Alpha subunit of the capping protein heterodimer which binds to the barbed ends of actin filaments preventing further polymerization; localized predominantly to cortical actin patches |
| *CAP2* | *YIL034C* | *AFUA_1G03060* | Beta subunit of the capping protein heterodimer which binds to the barbed ends of actin filaments preventing further polymerization; localized predominantly to cortical actin patches |
| *CAR1* | *YPL111W* | *AFUA_3G11430* | Arginase, responsible for arginine degradation, expression responds to both induction by arginine and nitrogen catabolite repression; disruption enhances freeze tolerance |
| *CAR2* | *YLR438W* | *AFUA_4G09140* | L-ornithine aminotransferase Car2 |
| *CAT2* | *YML042W* | *AFUA_2G12530* | Carnitine acetyl-CoA transferase present in both mitochondria and peroxisomes, transfers activated acetyl groups to carnitine to form acetylcarnitine which can be shuttled across membranes |
| *CAT5* | *YOR125C* | *AFUA_2G01890* | Ubiquinone biosynthesis protein Coq7 |
| *CAT8* | *YMR280C* | *AFUA_1G13510* | C6 transcription factor FacB/Cat8 |
| *CBC2* | *YPL178W* | *AFUA_2G08570* | Small subunit of nuclear cap-binding protein complex |
| *CBF1* | *YJR060W* | *AFUA_5G08020* | HLH DNA binding protein (Penr2) |
| *CBF5* | *YLR175W* | *AFUA_5G05710* | Pseudouridylate synthase family protein |
| *CBK1* | *YNL161W* | *AFUA_4G11890* | Serine/threonine protein kinase |
| *CBR1* | *YIL043C* | *AFUA_2G14060* | NADH-cytochrome b5 reductase |
| *CCA1* | *YER168C* | *AFUA_2G16660* | ATP (CTP):tRNA-specific tRNA nucleotidyltransferase; different forms targeted to the nucleus, cytosol, and mitochondrion are generated via the use of multiple transcriptional and translational start sites |
| *CCC1* | *YLR220W* | *AFUA_3G09970 AFUA_4G12530* | Vacuolar iron transporter Ccc1 |
| *CCC2* | *YDR270W* | *AFUA_4G12620* | Copper-transporting ATPase |
| *CCL1* | *YPR025C* | *AFUA_5G07030* | Cyclin Ccl1 |
| *CCP1* | *YKR066C* | *AFUA_4G09110 AFUA_6G13570* | Cytochrome c peroxidase Ccp1 |
| *CCR4* | *YAL021C* | *AFUA_4G12750* | Transcription factor |
| *CCS1* | *YMR038C* | *AFUA_2G09700* | Superoxide dismutase copper chaperone Lys7 |
| *CCT2* | *YIL142W* | *AFUA_1G01740* | T-complex protein 1, beta subunit |
| *CCT3* | *YJL014W* | *AFUA_3G13030* | T-complex protein 1, gamma subunit (Cct3) |
| *CCT4* | *YDL143W* | *AFUA_3G07830* | T-complex protein 1, delta subunit |
| *CCT5* | *YJR064W* | *AFUA_6G07540* | T-complex protein 1, epsilon subunit |
| *CCT6* | *YDR188W* | *AFUA_3G09590* | T-complex protein 1, zeta subunit |
| *CCT7* | *YJL111W* | *AFUA_1G06710* | T-complex protein 1, eta subunit |
| *CCT8* | *YJL008C* | *AFUA_4G09740* | T-complex protein 1, theta subunit |
| *CDC1* | *YDR182W* | *AFUA_2G14580* | Manganese ion homeostasis (Fr) |
| *CDC10* | *YCR002C* | *AFUA_1G08850* | Septin |
| *CDC11* | *YJR076C* | *AFUA_5G08540* | Septin AspA |
| *CDC12* | *YHR107C* | *AFUA_5G03080* | Septin |
| *CDC16* | *YKL022C* | *AFUA_5G02590* | 20S cyclosome subunit (Cut9/Cdc16) |
| *CDC20* | *YGL116W* | *AFUA_1G14730* | Cell division cycle protein Cdc20 |
| *CDC21* | *YOR074C* | *AFUA_4G04270* | Thymidylate synthase |
| *CDC23* | *YHR166C* | *AFUA_5G02440* | 20S cyclosome subunit (APC8) |
| *CDC24* | *YAL041W* | *AFUA_4G11450* | Rho guanyl nucleotide exchange factor |
| *CDC25* | *YLR310C* | *AFUA_2G16240* | Cell division control protein Cdc25 |
| *CDC27* | *YBL084C* | *AFUA_2G08620* | 20S cyclosome subunit (BimA/Nuc2/Cdc27) |
| *CDC3* | *YLR314C* | *AFUA_7G05370* | Septin AspB |
| *CDC31* | *YOR257W* | *AFUA_4G11160* | Calcium-binding component of the spindle pole body half-bridge, required for SPB duplication in mitosis and meiosis II; homolog of mammalian centrin; binds multiubiquitinated proteins and is involved in proteasomal protein degradation |
| *CDC33* | *YOL139C* | *AFUA_7G01480* | Cap binding protein |
| *CDC34* | *YDR054C* | *AFUA_5G09200* | Ubiquitin conjugating enzyme (UbcC) |
| *CDC36* | *YDL165W* | *AFUA_2G11130* | NOT2 family protein |
| *CDC39* | *YCR093W* | *AFUA_3G10240* | Ccr4-Not transcription complex subunit (NOT1) |
| *CDC4* | *YFL009W* | *AFUA_6G13030* | Cell division control protein Cdc4 |
| *CDC40* | *YDR364C* | *AFUA_6G07300* | MRNA splicing factor (Prp17) |
| *CDC42* | *YLR229C* | *AFUA_2G05740* | Rho GTPase ModA |
| *CDC43* | *YGL155W* | *AFUA_6G06710* | Beta subunit of geranylgeranyltransferase type I, catalyzes geranylgeranylation to the cysteine residue in proteins containing a C-terminal CaaX sequence ending in Leu or Phe; has substrates important for morphogenesis |
| *CDC45* | *YLR103C* | *AFUA_1G15310* | DNA replication initiation factor Cdc45 |
| *CDC5* | *YMR001C* | *AFUA_8G05680* | Serine/threonine protein kinase |
| *CDC55* | *YGL190C* | *AFUA_8G05560* | Protein phosphatase PP2A regulatory B subunit |
| *CDC6* | *YJL194W* | *AFUA_1G10720 AFUA_7G04310* | Origin recognition complex subunit Orc1 |
| *CDC60* | *YPL160W* | *AFUA_6G12630* | Leucyl-tRNA synthetase |
| *CDC7* | *YDL017W* | *AFUA_3G05540* | Cell cycle protein kinase |
| *CDC73* | *YLR418C* | *AFUA_2G11190* | Pol II transcription elongation factor subunit Cdc73, putative |
| *CDC8* | *YJR057W* | *AFUA_5G03460* | Thymidylate kinase |
| *CDC9* | *YDL164C* | *AFUA_2G09010* | DNA ligase Cdc9 |
| *CDS1* | *YBR029C* | *AFUA_1G07010* | Phosphatidate cytidylyltransferase |
| *CEG1* | *YGL130W* | *AFUA_2G05780* | Alpha subunit of the mRNA capping enzyme, a heterodimer involved in adding the 5' cap to mRNA; the mammalian enzyme is a single bifunctional polypeptide |
| *CEM1* | *YER061C* | *AFUA_2G05760* | Beta-ketoacyl synthase (Cem1) |
| *CEP3* | *YMR168C* | *AFUA_1G01560 AFUA_4G01470* | C6 finger domain protein |
| *CET1* | *YMR180C YPL228W* | *AFUA_4G08510* | Beta subunit of the mRNA capping enzyme, a heterodimer involved in adding the 5' cap to mRNA; the mammalian enzyme is a single bifunctional polypeptide |
| *CEX1* | *YOR112W* | *AFUA_1G09620* | Protein kinase family protein |
| *CFD1* | *YIL003W* | *AFUA_5G03600* | Nucleotide binding protein |
| *CFT1* | *YDR301W* | *AFUA_8G04040* | Cleavage and polyadenylation specificity factor subunit A |
| *CFT2* | *YLR115W* | *AFUA_3G09720* | Cleavage and polyadenylylation specificity factor, putative |
| *CGI121* | *YML036W* | *AFUA_2G03210* | Cgi121p |
| *CHA4* | *YFR057W YLR098C YOR337W* | *AFUA_6G07950 AFUA_1G16220 AFUA_5G12020 AFUA_2G10400 AFUA_5G01662 AFUA_7G01820 AFUA_5G09740 AFUA_6G01840 AFUA_3G03920 AFUA_4G03430* | C6 transcription factor |
| *CHC1* | *YGL206C* | *AFUA_4G07700* | Clathrin heavy chain |
| *CHK1* | *YBR274W* | *AFUA_6G13160* | Serine/threonine protein kinase |
| *CHL1* | *YPL008W* | *AFUA_3G05590* | DNA helicase |
| *CHL4* | *YDR254W* | *AFUA_4G06540* | CHL4 family chromosome segregation protein |
| *CHO2* | *YGR157W* | *AFUA_2G15970* | Phosphatidylethanolamine methyltransferase |
| *CHS1* | *YNL192W* | *AFUA_2G01870* | Chitin synthase I, requires activation from zymogenic form in order to catalyze the transfer of N-acetylglucosamine to chitin; required for repairing the chitin septum during cytokinesis; transcription activated by mating factor |
| *CHS2* | *YBR038W* | *AFUA_4G04180* | Chitin synthase II; catalyzes transfer of N-acetylglucosamine to chitin upon activation of zymogenic form; required for chitin synthesis in the primary septum during cytokinesis; localization regulated by Cdk1p during mitosis |
| *CHS3* | *YBR023C* | *AFUA_8G05630* | Chitin synthase III, catalyzes the transfer of N-acetylglucosamine to chitin; required for synthesis of the majority of cell wall chitin, the chitin ring during bud emergence, and spore wall chitosan |
| *CHS5* | *YLR330W* | *AFUA_6G02510* | Chitin biosynthesis protein (Chs5) |
| *CHS7* | *YHR142W* | *AFUA_1G12040* | Chitin biosynthesis protein (Chs7) |
| *CHZ1* | *YER030W* | *AFUA_1G07430* | Chz1p |
| *CIA1* | *YDR267C* | *AFUA_1G08930* | WD repeat protein |
| *CIC1* | *YHR052W* | *AFUA_7G05460* | Cic1p |
| *CIN1* | *YOR349W* | *AFUA_5G11940* | Tubulin-specific chaperone D |
| *CIT1* | *YCR005C YNR001C* | *AFUA_5G04230* | Citrate synthase (Cit1) |
| *CKA2* | *YOR061W* | *AFUA_8G04810* | Alpha' catalytic subunit of casein kinase 2, a Ser/Thr protein kinase with roles in cell growth and proliferation; the holoenzyme also contains CKA1, CKB1 and CKB2, the many substrates include transcription factors and all RNA polymerases |
| *CKB1* | *YGL019W* | *AFUA_4G06180* | Beta regulatory subunit of casein kinase 2, a Ser/Thr protein kinase with roles in cell growth and proliferation; the holoenzyme also contains CKA1, CKA2 and CKB2, the many substrates include transcription factors and all RNA polymerases |
| *CKB2* | *YOR039W* | *AFUA_1G09950* | Beta' regulatory subunit of casein kinase 2, a Ser/Thr protein kinase with roles in cell growth and proliferation; the holoenzyme also contains CKA1, CKA2 and CKB1, the many substrates include transcription factors and all RNA polymerases |
| *CLC1* | *YGR167W* | *AFUA_4G10020* | Clathrin light chain, subunit of the major coat protein involved in intracellular protein transport and endocytosis; thought to regulate clathrin function; two Clathrin heavy chains form the clathrin triskelion structural component |
| *CLD1* | *YGR110W* | *AFUA_1G03890* | Alpha/beta hydrolase |
| *CLP1* | *YOR250C* | *AFUA_5G11400* | MRNA cleavage factor complex II protein Clp1 |
| *CLU1* | *YMR012W* | *AFUA_3G10800* | Eukaryotic translation initiation factor 3 subunit CLU1/TIF31 |
| *CMC2* | *YBL059C-A* | *AFUA_1G02620* | Cmc2p |
| *CMD1* | *YBR109C* | *AFUA_4G10050* | Calmodulin; Ca++ binding protein that regulates Ca++ independent processes and Ca++ dependent processes (stress-activated pathways), targets include Nuf1p, Myo2p and calcineurin |
| *CMK1* | *YFR014C YOL016C* | *AFUA_2G13680* | Calmodulin-dependent protein kinase; may play a role in stress response, many CA++/calmodulan dependent phosphorylation substrates demonstrated in vitro, amino acid sequence similar to Cmk2p and mammalian Cam Kinase II |
| *CMP2* | *YLR433C YML057W* | *AFUA_5G09360* | Calcineurin A; one isoform of the catalytic subunit of calcineurin, a Ca++/calmodulin-regulated protein phosphatase which regulates Crz1p (a stress-response transcription factor), the other calcineurin subunit is CNB1 |
| *CMS1* | *YLR003C* | *AFUA_3G06320* | Cms1p |
| *CNB1* | *YKL190W* | *AFUA_6G04540* | Calcineurin B; the regulatory subunit of calcineurin, a Ca++/calmodulin-regulated type 2B protein phosphatase which regulates Crz1p (a stress-response transcription factor), the other calcineurin subunit is encoded by CNA1 and/or CMP1 |
| *CNE1* | *YAL058W* | *AFUA_4G12850* | Calnexin; integral membrane ER chaperone involved in folding and quality control of glycoproteins; chaperone activity is inhibited by Mpd1p, with which Cne1p interacts; 24% identical to mammalian calnexin; Ca+ binding not yet shown in yeast |
| *CNS1* | *YBR155W* | *AFUA_6G13600* | TPR repeat protein |
| *COA1* | *YIL157C* | *AFUA_4G05990* | Coa1p |
| *COF1* | *YLL050C* | *AFUA_5G10570* | Cofilin |
| *COG3* | *YER157W* | *AFUA_3G11090* | Golgi complex component Cog3 |
| *COG4* | *YPR105C* | *AFUA_2G05890* | Cog4p |
| *COG5* | *YNL051W* | *AFUA_3G09810* | Golgi transport complex component Cog5, putaitve |
| *COG6* | *YNL041C* | *AFUA_6G12470* | Golgi transport complex subunit Cog6 |
| *COQ1* | *YBR003W* | *AFUA_3G06120* | Hexaprenyl pyrophosphate synthetase Coq1 |
| *COQ10* | *YOL008W* | *AFUA_6G07220* | Coenzyme Q binding protein, functions in the delivery of Q6 to its proper location for electron transport during respiration; START domain protein with homologs in bacteria and eukaryotes |
| *COQ2* | *YNR041C* | *AFUA_4G05970* | Para-hydroxybenzoate-polyprenyltransferase Coq2, putative |
| *COQ3* | *YOL096C* | *AFUA_4G10370* | 3-demethylubiquinone-9 3-methyltransferase |
| *COQ5* | *YML110C* | *AFUA_6G08850* | 2-hexaprenyl-6-methoxy-1,4-benzoquinone methyltransferase |
| *COQ9* | *YLR201C* | *AFUA_6G12310* | Coq9p |
| *COT1* | *YMR243C YOR316C* | *AFUA_2G14570* | Vacuolar membrane zinc transporter, transports zinc from the cytosol into the vacuole for storage; also has a role in resistance to zinc shock resulting from a sudden influx of zinc into the cytoplasm |
| *COX10* | *YPL172C* | *AFUA_4G08340* | Heme a biosynthesis protein |
| *COX11* | *YPL132W* | *AFUA_2G12260* | Cytochrome c oxidase assembly protein Cox11 |
| *COX12* | *YLR038C* | *AFUA_2G13010* | Cytochrome c oxidase polypeptide vib |
| *COX13* | *YGL191W* | *AFUA_3G06190* | Cytochrome c oxidase subunit VIa |
| *COX15* | *YER141W* | *AFUA_6G07670* | Cytochrome c oxidase assembly protein cox15 |
| *COX17* | *YLL009C* | *AFUA_3G07690* | Copper metallochaperone that transfers copper to Sco1p and Cox11p for eventual delivery to cytochrome c oxidase; contains twin cysteine-x9-cysteine motifs |
| *COX18* | *YGR062C* | *AFUA_1G06880* | Mitochondrial export translocase Oxa2 |
| *COX19* | *YLL018C-A* | *AFUA_1G09757* | Cytochrome c oxidase assembly protein Cox19 |
| *COX4* | *YGL187C* | *AFUA_2G03010* | Cytochrome c subunit Vb |
| *COX5A* | *YIL111W YNL052W* | *AFUA_5G10560* | Cytochrome c oxidase subunit V |
| *COX6* | *YHR051W* | *AFUA_5G02750* | Cytochrome c oxidase subunit Va |
| *COX9* | *YDL067C* | *AFUA_3G14440* | Cytochrome c oxidase family protein |
| *COY1* | *YKL179C* | *AFUA_1G14240* | Golgi membrane protein (Coy1) |
| *CPA1* | *YOR303W* | *AFUA_5G06780* | Carbamoyl-phosphate synthase, small subunit |
| *CPA2* | *YJR109C* | *AFUA_2G10070* | Carbamoyl-phosphate synthase, large subunit |
| *CPR1* | *YDR155C* | *AFUA_3G07430* | Peptidyl-prolyl cis-trans isomerase/cyclophilin, putative |
| *CPR2* | *YHR057C YDR304C* | *AFUA_4G07650* | Peptidyl-prolyl cis-trans isomerase (CypB) |
| *CPR6* | *YLR216C* | *AFUA_2G02050* | Peptidyl-prolyl cis-trans isomerase Cpr7 |
| *CPS1* | *YJL172W* | *AFUA_3G07040* | Vacuolar carboxypeptidase Cps1 |
| *CPT1* | *YHR123W YNL130C* | *AFUA_3G06650* | Cholinephosphotransferase, required for phosphatidylcholine biosynthesis and for inositol-dependent regulation of EPT1 transcription |
| *CRG1* | *YER175C YHR209W* | *AFUA_2G01390* | S-adenosylmethionine-dependent methyltransferase, putative |
| *CRH1* | *YGR189C* | *AFUA_6G08510* | Chitin transglycosylase that functions in the transfer of chitin to beta(1-6) and beta(1-3) glucans in the cell wall; similar and functionally redundant to Utr2; localizes to sites of polarized growth; expression induced by cell wall stress |
| *CRM1* | *YGR218W* | *AFUA_1G08790* | Exportin KapK |
| *CRN1* | *YLR429W* | *AFUA_2G14270* | Coronin, cortical actin cytoskeletal component that associates with the Arp2p/Arp3p complex to regulate its activity; plays a role in regulation of actin patch assembly |
| *CSE1* | *YGL238W* | *AFUA_6G04010* | Chromosome segregation protein Cse1 |
| *CSF1* | *YLR087C* | *AFUA_2G13520* | Fermentation associated protein (Csf1) |
| *CSL4* | *YNL232W* | *AFUA_5G13530* | Exosome complex subunit Csl4 |
| *CSM3* | *YMR048W* | *AFUA_4G04745* | Replication fork protection component Swi3 |
| *CSN12* | *YJR084W* | *AFUA_1G04520 AFUA_6G14290* | Csn12p |
| *CSR1* | *YLR380W* | *AFUA_6G12690 AFUA_4G13930 AFUA_7G06760* | CRAL/TRIO domain protein |
| *CST26* | *YBR042C YDR018C* | *AFUA_5G05880* | Probable membrane protein with three predicted transmembrane domains; homologous to Ybr042cp, similar to C. elegans F55A11.5 and maize 1-acyl-glycerol-3-phosphate acyltransferase |
| *CTA1* | *YDR256C* | *AFUA_2G18030* | Catalase A, breaks down hydrogen peroxide in the peroxisomal matrix formed by acyl-CoA oxidase during fatty acid beta-oxidation |
| *CTF18* | *YMR078C* | *AFUA_7G05480* | Sister chromatid cohesion factor (Chl12) |
| *CTF4* | *YPR135W* | *AFUA_1G07320* | Chromatin-associated protein, required for sister chromatid cohesion; interacts with DNA polymerase alpha and may link DNA synthesis to sister chromatid cohesion |
| *CTF8* | *YHR191C* | *AFUA_4G10630* | Sister chromatid cohesion protein Ctf8 |
| *CTI6* | *YPL181W* | *AFUA_5G08840* | Transcriptional regulator (Cti6) |
| *CTK1* | *YKL139W* | *AFUA_5G03160* | Catalytic subunit of C-terminal domain kinase I (CTDK-I), which phosphorylates both RNA pol II subunit Rpo21p to affect transcription and pre-mRNA 3' end processing, and ribosomal protein Rps2p to increase translational fidelity |
| *CTK2* | *YJL006C* | *AFUA_1G07020* | Beta subunit of C-terminal domain kinase I (CTDK-I), which phosphorylates both RNA pol II subunit Rpo21p to affect transcription and pre-mRNA 3' end processing, and ribosomal protein Rps2p to increase translational fidelity |
| *CTP1* | *YBR291C* | *AFUA_3G05420* | Mitochondrial tricarboxylate transporter (Ctp), putative |
| *CTR2* | *YHR175W* | *AFUA_3G08180* | Ctr copper transporter family protein |
| *CTR3* | *YLR411W* | *AFUA_2G03730* | Ctr copper transporter family protein |
| *CTR86* | *YCR054C* | *AFUA_6G07580* | Essential cytoplasmic protein Ctr86 |
| *CTR9* | *YOL145C* | *AFUA_5G05870* | Component of the Paf1p complex that binds to and modulates the activity of RNA polymerases I and II; required for expression of a subset of genes, including cyclin genes; contains TPR repeats |
| *CTS1* | *YLR286C* | *AFUA_5G03760* | Class III chitinase ChiA1 |
| *CUE3* | *YGL110C* | *AFUA_2G02140* | CUE domain protein |
| *CUE5* | *YOR042W* | *AFUA_1G09250* | CUE domain protein |
| *CUP5* | *YEL027W* | *AFUA_3G12370* | Vacuolar ATP synthase 16 kDa proteolipid subunit, putative |
| *CUP9* | *YGL096W YPL177C* | *AFUA_4G10110* | Homeodomain-containing protein and putative transcription factor found associated with chromatin; target of SBF transcription factor; induced during meiosis and under cell-damaging conditions; similar to Cup9p transcription factor |
| *CUS1* | *YMR240C* | *AFUA_5G04420* | Splicing factor 3b, subunit 2, 145kD |
| *CWC2* | *YDL209C* | *AFUA_5G06760* | Cell cycle control protein (Cwf2) |
| *CWC22* | *YGR278W* | *AFUA_1G03010* | Cell cycle control protein (Cwf22) |
| *CWC23* | *YGL128C* | *AFUA_4G06920* | Component of a complex containing Cef1p, putatively involved in pre-mRNA splicing; has similarity to E. coli DnaJ and other DnaJ-like proteins and to S. pombe Cwf23p |
| *CWC24* | *YLR323C* | *AFUA_5G07720* | CCCH and RING finger protein (Znf183) |
| *CWC25* | *YNL245C* | *AFUA_2G10330* | Cwc25p |
| *CWH41* | *YGL027C* | *AFUA_6G04210* | Mannosyl-oligosaccharide glucosidase |
| *CWH43* | *YCR017C* | *AFUA_3G09740* | Integral plasma membrane protein |
| *CYB2* | *YML054C* | *AFUA_4G03120* | Mitochondrial cytochrome b2 |
| *CYC1* | *YEL039C YJR048W* | *AFUA_2G13110* | Cytochrome c |
| *CYC2* | *YOR037W* | *AFUA_6G04980* | Cytochrome c mitochondrial import factor (Cyc2), putative |
| *CYC3* | *YAL039C* | *AFUA_4G04600* | Cytochrome c heme lyase |
| *CYC8* | *YBR112C* | *AFUA_2G11840* | Transcriptional corepressor Cyc8 |
| *CYK3* | *YDL117W* | *AFUA_5G03400* | SH3 domain protein (Cyk3) |
| *CYM1* | *YDR430C* | *AFUA_4G07910* | Pitrilysin family metalloprotease (Cym1) |
| *CYR1* | *YJL005W* | *AFUA_6G08520* | Adenylate cyclase, required for cAMP production and cAMP-dependent protein kinase signaling; the cAMP pathway controls a variety of cellular processes, including metabolism, cell cycle, stress response, stationary phase, and sporulation |
| *CYS3* | *YAL012W* | *AFUA_8G04340* | Cystathionine gamma-lyase |
| *CYT1* | *YOR065W* | *AFUA_1G02070* | Cytochrome C1/Cyt1 |
| *CYT2* | *YKL087C* | *AFUA_4G07120* | CC1HL; cytochrome c1 heme lyase |
| *DAD1* | *YDR016C* | *AFUA_5G14070* | Dad1p |
| *DAK1* | *YFL053W YML070W* | *AFUA_5G12690* | Dihydroxyacetone kinase (DakA) |
| *DAL4* | *YBL042C YBR021W YIR028W* | *AFUA_1G13210* | Allantoin permease; expression sensitive to nitrogen catabolite repression and induced by allophanate, an intermediate in allantoin degradation |
| *DAL5* | *YJR152W* | *AFUA_2G08350 AFUA_3G02080* | Allantoate permease; ureidosuccinate permease; also transports dipeptides, though with lower affinity than for allantoate and ureidosuccinate; expression is constitutive but sensitive to nitrogen catabolite repression |
| *DAL7* | *YIR031C YNL117W* | *AFUA_6G03540* | Malate synthase, enzyme of the glyoxylate cycle, involved in utilization of non-fermentable carbon sources; expression is subject to carbon catabolite repression; localizes in peroxisomes during growth in oleic acid medium |
| *DAL80* | *YJL110C YKR034W* | *AFUA_2G13380* | GATA zinc finger protein and Dal80p homolog that negatively regulates nitrogen catabolic gene expression by competing with Gat1p for GATA site binding; function requires a repressive carbon source; dimerizes with Dal80p and binds to Tor1p |
| *DAL81* | *YIR023W* | *AFUA_3G08050* | C6 transcription factor (OTam) |
| *DAM1* | *YGR113W* | *AFUA_6G12700* | DASH complex component Dam1 |
| *DAP1* | *YPL170W* | *AFUA_3G10490* | DNA damage response protein (Dap1) |
| *DAP2* | *YHR028C* | *AFUA_3G07850* | Pheromone maturation dipeptidyl aminopeptidase DapB |
| *DBF4* | *YDR052C* | *AFUA_6G09180* | G1/S regulator NimO |
| *DBP1* | *YOR204W YPL119C* | *AFUA_4G07660* | ATP-dependent DEAD (Asp-Glu-Ala-Asp)-box RNA helicase, required for translation initiation of all yeast mRNAs; mutations in human DEAD-box DBY are a frequent cause of male infertility |
| *DBP2* | *YNL112W* | *AFUA_2G10750* | RNA helicase (Dbp) |
| *DBP3* | *YGL078C* | *AFUA_2G06310* | ATP-dependent RNA helicase |
| *DBP5* | *YOR046C* | *AFUA_2G01210* | Cytoplasmic ATP-dependent RNA helicase of the DEAD-box family involved in mRNA export from the nucleus; involved in translation termination |
| *DBP6* | *YNR038W* | *AFUA_1G16940* | DEAD/DEAH box helicase |
| *DBP9* | *YLR276C* | *AFUA_2G13980* | ATP-dependent RNA helicase of the DEAD-box family involved in biogenesis of the 60S ribosomal subunit |
| *DBR1* | *YKL149C* | *AFUA_6G10370* | RNA lariat debranching enzyme |
| *DCI1* | *YLR284C YOR180C* | *AFUA_6G04040* | Peroxisomal D3,D2-enoyl-CoA isomerase |
| *DCN1* | *YLR128W* | *AFUA_2G17070* | DUF298 domain protein |
| *DCR2* | *YLR361C* | *AFUA_4G12720* | Phosphoesterase |
| *DCS1* | *YLR270W YOR173W* | *AFUA_2G05580* | MRNA decapping hydrolase |
| *DDI1* | *YER143W* | *AFUA_7G06050* | DNA damage-inducible v-SNARE binding protein, contains a ubiquitin-associated domain, may act as a negative regulator of constitutive exocytosis, may play a role in S-phase checkpoint control |
| *DDP1* | *YOR163W* | *AFUA_2G13080* | Nudix/MutT family protein |
| *DEF1* | *YKL054C* | *AFUA_3G11740* | RNAPII degradation factor Def1 |
| *DEG1* | *YFL001W* | *AFUA_3G13350* | Pseudouridylate synthase 3 |
| *DEP1* | *YAL013W* | *AFUA_8G04410* | Dep1p |
| *DER1* | *YBR201W* | *AFUA_6G04670* | ER-associated proteolytic system protein Der1 |
| *DET1* | *YDR051C* | *AFUA_3G10050* | Acid phosphatase involved in the non-vesicular transport of sterols in both directions between the endoplasmic reticulum and plasma membrane; deletion confers sensitivity to nickel |
| *DFR1* | *YOR236W* | *AFUA_2G08890* | Dihydrofolate reductase |
| *DGA1* | *YOR245C* | *AFUA_7G03700 AFUA_4G14150* | Diacylglycerol acyltransferase type 2A |
| *DGK1* | *YOR311C* | *AFUA_3G13270* | Phosphatidate cytidylyltransferase |
| *DGR2* | *YMR102C YKL121W* | *AFUA_5G12330* | Protein of unknown function; transcription is activated by paralogous transcription factors Yrm1p and Yrr1p along with genes involved in multidrug resistance; mutant shows increased resistance to azoles; YMR102C is not an essential gene |
| *DHH1* | *YDL160C* | *AFUA_3G05430* | Cytoplasmic DExD/H-box helicase, stimulates mRNA decapping, coordinates distinct steps in mRNA function and decay, interacts with both the decapping and deadenylase complexes, may have a role in mRNA export and translation |
| *DIB1* | *YPR082C* | *AFUA_3G12290* | 17-kDa component of the U4/U6aU5 tri-snRNP, plays an essential role in pre-mRNA splicing, orthologue of hDIM1, the human U5-specific 15-kDa protein |
| *DIC1* | *YLR348C* | *AFUA_2G13020* | Mitochondrial dicarboxylate carrier |
| *DID2* | *YKR035W-A* | *AFUA_3G04060* | Class E protein of the vacuolar protein-sorting pathway; binds Vps4p and directs it to dissociate ESCRT-III complexes; forms a functional and physical complex with Ist1p; human ortholog may be altered in breast tumors |
| *DID4* | *YKL002W* | *AFUA_5G13410* | Class E Vps protein of the ESCRT-III complex, required for sorting of integral membrane proteins into lumenal vesicles of multivesicular bodies, and for delivery of newly synthesized vacuolar enzymes to the vacuole, involved in endocytosis |
| *DIM1* | *YPL266W* | *AFUA_7G04860* | Dimethyladenosine transferase |
| *DIP5* | *YPL265W* | *AFUA_2G08800* | Dicarboxylic amino acid permease, mediates high-affinity and high-capacity transport of L-glutamate and L-aspartate; also a transporter for Gln, Asn, Ser, Ala, and Gly |
| *DIS3* | *YOL021C* | *AFUA_4G12250* | Exosome complex exonuclease exoribonuclease (Rrp44), putative |
| *DJP1* | *YIR004W* | *AFUA_7G01230* | Cytosolic J-domain-containing protein, required for peroxisomal protein import and involved in peroxisome assembly, homologous to E. coli DnaJ |
| *DLD1* | *YDL174C* | *AFUA_1G17520 AFUA_3G06820 AFUA_1G00510 AFUA_7G02560* | D-lactate dehydrogenase (cytochrome) |
| *DLD2* | *YEL071W YDL178W* | *AFUA_5G02230* | D-lactate dehydrogenase, part of the retrograde regulon which consists of genes whose expression is stimulated by damage to mitochondria and reduced in cells grown with glutamate as the sole nitrogen source, located in the cytoplasm |
| *DLT1* | *YMR126C* | *AFUA_4G11467* | Thermatolerance membrane protein Dlt1 |
| *DMA1* | *YHR115C YNL116W* | *AFUA_5G13560* | FHA domain protein |
| *DMC1* | *YER179W* | *AFUA_7G02200* | Meiotic recombination protein (Dmc1) |
| *DML1* | *YMR211W* | *AFUA_1G15070* | MtDNA inheritance protein Dml1 |
| *DNA2* | *YHR164C* | *AFUA_4G12210* | DNA replication helicase Dna2 |
| *DNL4* | *YOR005C* | *AFUA_5G12050* | DNA ligase required for nonhomologous end-joining (NHEJ), forms stable heterodimer with required cofactor Lif1p, interacts with Nej1p; involved in meiosis, not essential for vegetative growth |
| *DNM1* | *YLL001W* | *AFUA_8G02840* | Dynamin-like GTPase Dnm1 |
| *DOP1* | *YDR141C* | *AFUA_2G05020* | Cellular morphogenesis regulator DopA |
| *DOS2* | *YDR068W* | *AFUA_2G04840* | BSD domain protein |
| *DOT6* | *YBL054W YER088C* | *AFUA_1G07560 AFUA_2G02990* | PAC motif binding protein involved in rRNA and ribosome biogenesis; subunit of the RPD3L histone deacetylase complex; Myb-like HTH transcription factor, similar to Dot6p; hypophosphorylated by rapamycin treatment in a Sch9p-dependent manne |
| *DPB11* | *YJL090C* | *AFUA_5G13380* | BRCT domain protein |
| *DPB2* | *YPR175W* | *AFUA_2G06190* | DNA polymerase epsilon subunit B |
| *DPB4* | *YDR121W* | *AFUA_1G10550* | CBF/NF-Y family transcription factor |
| *DPH1* | *YIL103W* | *AFUA_2G12440* | Diphthamide biosynthesis protein |
| *DPM1* | *YPR183W* | *AFUA_3G10400* | Dolichol phosphate mannose synthase of the ER membrane, catalyzes the formation of Dol-P-Man from Dol-P and GDP-Man; required for glycosyl phosphatidylinositol membrane anchoring, O mannosylation, and protein glycosylation |
| *DPS1* | *YLL018C* | *AFUA_2G02590* | Aspartyl-tRNA synthetase Dps1 |
| *DRS1* | *YLL008W* | *AFUA_1G14990* | ATP-dependent RNA helicase (Drs1) |
| *DSD1* | *YGL196W* | *AFUA_4G03722* | D-serine dehydratase (aka D-serine ammonia-lyase); converts D-serine to pyruvate and ammonia by a reaction dependent on pyridoxal 5'-phosphate and zinc; may play a role in D-serine detoxification; L-serine is not a substrate |
| *DSF2* | *YBR007C* | *AFUA_1G02760* | Deletion suppressor of mpt5 mutation |
| *DSK2* | *YMR276W* | *AFUA_6G13420* | Ubiquitin-like protein DskB |
| *DSS1* | *YMR287C* | *AFUA_7G01550* | 3'-5' exoribonuclease, component of the mitochondrial degradosome along with the ATP-dependent RNA helicase Suv3p; the degradosome associates with the ribosome and mediates turnover of aberrant or unprocessed RNAs |
| *DST1* | *YGL043W* | *AFUA_3G07670* | Transcription elongation factor S-II |
| *DTD1* | *YDL219W* | *AFUA_2G02060* | D-tyrosyl-tRNA(Tyr) deacylase |
| *DUG1* | *YFR044C* | *AFUA_3G05450* | Cys-Gly metallo-di-peptidase; forms a complex with Dug2p and Dug3p to degrade glutathione and other peptides containing a gamma-glu-X bond in an alternative pathway to GSH degradation by gamma-glutamyl transpeptidase (Ecm38p) |
| *DUG2* | *YBR281C* | *AFUA_2G04360* | WD repeat protein |
| *DUG3* | *YNL191W* | *AFUA_1G11980* | Glutamine amidotransferase |
| *DUN1* | *YDL101C* | *AFUA_7G00530 AFUA_7G03750* | Cell-cycle checkpoint serine-threonine kinase required for DNA damage-induced transcription of certain target genes, phosphorylation of Rad55p and Sml1p, and transient G2/M arrest after DNA damage; also regulates postreplicative DNA repair |
| *DUR3* | *YHL016C* | *AFUA_1G04870* | Urea transporter (Dur3) |
| *DUS1* | *YML080W* | *AFUA_1G16550* | Dihydrouridine synthase, member of a widespread family of conserved proteins including Smm1p, Dus3p, and Dus4p; modifies pre-tRNA(Phe) at U17 |
| *DUS3* | *YLR401C* | *AFUA_1G14770* | Dihydrouridine synthase, member of a widespread family of conserved proteins including Smm1p, Dus1p, and Dus4p; contains a consensus oleate response element in its promoter region |
| *DYN1* | *YKR054C* | *AFUA_5G11810* | Cytoplasmic heavy chain dynein, microtubule motor protein, required for anaphase spindle elongation; involved in spindle assembly, chromosome movement, and spindle orientation during cell division, targeted to microtubule tips by Pac1p |
| *DYN2* | *YDR424C* | *AFUA_1G04850* | Cytoplasmic light chain dynein, microtubule motor protein; proposed to be involved in the assembly of the nuclear pore complex |
| *EAF1* | *YDR359C* | *AFUA_4G07560* | Component of the NuA4 histone acetyltransferase complex; acts as a platform for assembly of NuA4 subunits into the native complex; required for initiation of pre-meiotic DNA replication, likely due to its requirement for expression of IME1 |
| *EAF3* | *YPR023C* | *AFUA_4G10660* | Histone acetylase complex subunit MRG15-2 |
| *EAF6* | *YJR082C* | *AFUA_3G08190* | Eaf6p |
| *EAP1* | *YKL204W* | *AFUA_5G10850 AFUA_5G01730* | MYB DNA-binding domain protein |
| *EAR1* | *YMR171C* | *AFUA_1G14910* | Endosomal SPRY domain protein |
| *EBP2* | *YKL172W* | *AFUA_5G12270* | RRNA processing protein (Ebp2) |
| *ECM10* | *YEL030W YJR045C* | *AFUA_2G09960* | Hsp70 family ATPase, constituent of the import motor component of the Translocase of the Inner Mitochondrial membrane (TIM23 complex); involved in protein translocation and folding; subunit of SceI endonuclease |
| *ECM16* | *YMR128W* | *AFUA_6G04330* | DEAH-box RNA helicase (Dhr1) |
| *ECO1* | *YFR027W* | *AFUA_3G06000* | Acetytransferase required for sister chromatid cohesion; modifies Smc3p at DNA replication forks during S-phase; modifies Mcd1p in response to double-strand DNA breaks during G2/M; acetylation of cohesin subunits antagonizes Rad61p |
| *EDC3* | *YEL015W* | *AFUA_5G13360* | YjeF_N domain protein |
| *EDE1* | *YBL047C* | *AFUA_1G02540* | EF hand domain protein |
| *EFB1* | *YAL003W* | *AFUA_1G11190* | Eukaryotic translation elongation factor 1 subunit Eef1-beta |
| *EFG1* | *YGR271C-A* | *AFUA_2G02870* | Nuclear protein involved in pre-rRNA processing, putative |
| *EFT1* | *YOR133W YDR385W* | *AFUA_2G13530* | Translation elongation factor EF-2 subunit |
| *EGD2* | *YHR193C* | *AFUA_6G03820* | Alpha subunit of the heteromeric nascent polypeptide-associated complex involved in protein sorting and translocation, associated with cytoplasmic ribosomes |
| *EHD3* | *YDR036C* | *AFUA_5G12790* | 3-hydroxyisobutyryl-CoA hydrolase, member of a family of enoyl-CoA hydratase/isomerases; non-tagged protein is detected in highly purified mitochondria in high-throughput studies; phosphorylated; mutation affects fluid-phase endocytosis |
| *ELA1* | *YNL230C* | *AFUA_2G08170* | RNA polymerase II transcription factor SIII subunit A |
| *ELC1* | *YPL046C* | *AFUA_6G10530* | Transcriptional elongation regulator Elc1/Elongin C, putative |
| *ELG1* | *YOR144C* | *AFUA_2G17190* | Elg1p |
| *ELP2* | *YGR200C* | *AFUA_6G05090* | RNA polymerase II Elongator subunit |
| *ELP4* | *YPL101W* | *AFUA_5G11130* | PAXNEB protein superfamily |
| *EMC1* | *YCL045C* | *AFUA_5G09270* | Emc1p |
| *EMC2* | *YJR088C* | *AFUA_7G03650* | Emc2p |
| *EMC4* | *YGL231C* | *AFUA_2G10350* | ER membrane DUF1077 domain protein |
| *EMG1* | *YLR186W* | *AFUA_3G06010* | RNA processing protein Emg1 |
| *EMI1* | *YDR512C* | *AFUA_1G07360* | Emi1p |
| *EMI5* | *YOL071W* | *AFUA_4G13180* | TPR repeat protein |
| *EMP46* | *YFL048C YLR080W* | *AFUA_2G16800* | Lectin family integral membrane protein |
| *EMP70* | *YLR083C YDR107C* | *AFUA_6G14250* | Protein with a role in cellular adhesion and filamentous growth; similar to Emp70p and Tmn3p; member of the evolutionarily conserved Transmembrane Nine family of proteins with nine membrane-spanning segments |
| *END3* | *YNL084C* | *AFUA_1G13020* | EH domain-containing protein involved in endocytosis, actin cytoskeletal organization and cell wall morphogenesis; forms a complex with Sla1p and Pan1p |
| *ENP1* | *YBR247C* | *AFUA_3G04110* | RRNA processing protein Bystin |
| *ENP2* | *YGR145W* | *AFUA_2G04780* | WD repeat protein |
| *ENT1* | *YLR206W YDL161W* | *AFUA_6G12570* | EH domain binding protein epsin 2 |
| *ENT3* | *YJR125C* | *AFUA_2G03650* | Golgi to endosome transport protein (Ent3) |
| *EPL1* | *YFL024C* | *AFUA_6G04530* | Component of NuA4, which is an essential histone H4/H2A acetyltransferase complex; homologous to Drosophila Enhancer of Polycomb |
| *ERB1* | *YMR049C* | *AFUA_1G09200* | Constituent of 66S pre-ribosomal particles, forms a complex with Nop7p and Ytm1p that is required for maturation of the large ribosomal subunit; required for maturation of the 25S and 5.8S ribosomal RNAs; homologous to mammalian Bop1 |
| *ERD1* | *YDR414C* | *AFUA_7G04250* | Protein-ER retention protein (Erd1) |
| *ERD2* | *YBL040C* | *AFUA_2G02980* | Protein-ER retention receptor |
| *ERF2* | *YLR246W* | *AFUA_3G06470* | DHHC zinc finger membrane protein |
| *ERG10* | *YPL028W* | *AFUA_8G04000* | Acetyl-CoA C-acetyltransferase (acetoacetyl-CoA thiolase), cytosolic enzyme that transfers an acetyl group from one acetyl-CoA molecule to another, forming acetoacetyl-CoA; involved in the first step in mevalonate biosynthesis |
| *ERG11* | *YHR007C* | *AFUA_4G06890 AFUA_7G03740* | 14-alpha sterol demethylase Cyp51A |
| *ERG12* | *YMR208W* | *AFUA_4G07780* | Mevalonate kinase |
| *ERG2* | *YMR202W* | *AFUA_1G04720* | C-8 sterol isomerase, catalyzes the isomerization of the delta-8 double bond to the delta-7 position at an intermediate step in ergosterol biosynthesis |
| *ERG20* | *YJL167W* | *AFUA_5G02450* | Farnesyl-pyrophosphate synthetase |
| *ERG25* | *YGR060W* | *AFUA_4G04820* | C-4 methyl sterol oxidase, catalyzes the first of three steps required to remove two C-4 methyl groups from an intermediate in ergosterol biosynthesis; mutants accumulate the sterol intermediate 4,4-dimethylzymosterol |
| *ERG27* | *YLR100W* | *AFUA_4G11500* | 3-keto sterol reductase, catalyzes the last of three steps required to remove two C-4 methyl groups from an intermediate in ergosterol biosynthesis; mutants are sterol auxotrophs |
| *ERG28* | *YER044C* | *AFUA_2G11550* | Endoplasmic reticulum membrane protein, may facilitate protein-protein interactions between the Erg26p dehydrogenase and the Erg27p 3-ketoreductase and/or tether these enzymes to the ER, also interacts with Erg6p |
| *ERG3* | *YLR056W* | *AFUA_2G00320 AFUA_6G05140* | C-5 sterol desaturase, catalyzes the introduction of a C-5(6) double bond into episterol, a precursor in ergosterol biosynthesis; mutants are viable, but cannot grow on non-fermentable carbon sources |
| *ERG5* | *YMR015C* | *AFUA_1G03950* | C-22 sterol desaturase, a cytochrome P450 enzyme that catalyzes the formation of the C-22(23) double bond in the sterol side chain in ergosterol biosynthesis; may be a target of azole antifungal drugs |
| *ERG7* | *YHR072W* | *AFUA_4G14770 AFUA_4G12040 AFUA_5G04080* | Oxidosqualene:lanosterol cyclase |
| *ERG8* | *YMR220W* | *AFUA_5G10680* | Phosphomevalonate kinase |
| *ERG9* | *YHR190W* | *AFUA_7G01220* | Farnesyl-diphosphate farnesyltransferase |
| *ERO1* | *YML130C* | *AFUA_8G05140* | Oxidoreductin |
| *ERP3* | *YDL018C* | *AFUA_5G03260* | Endosomal cargo receptor (Erp3) |
| *ERS1* | *YCR075C* | *AFUA_1G02850 AFUA_6G14530* | L-cystine transporter |
| *ERT1* | *YBR239C* | *AFUA_2G05830* | C6 finger domain protein |
| *ERV1* | *YGR029W* | *AFUA_3G08850* | FAD dependent sulfhydryl oxidase Erv1 |
| *ERV2* | *YPR037C* | *AFUA_7G04690* | FAD dependent sulfhydryl oxidase Erv2 |
| *ERV29* | *YGR284C* | *AFUA_1G11770* | COPII-coated vesicle protein SurF4/Erv29 |
| *ERV41* | *YML067C* | *AFUA_2G01530* | COPII-coated vesicle protein (Erv41) |
| *ERV46* | *YAL042W* | *AFUA_1G05120* | COPII-coated vesicle membrane protein Erv46 |
| *ESF1* | *YDR365C* | *AFUA_2G05420* | Pre-rRNA processing protein Esf1 |
| *ESF2* | *YNR054C* | *AFUA_2G16760* | Esf2p |
| *ESP1* | *YGR098C* | *AFUA_5G09710* | Separin |
| *EST2* | *YLR318W* | *AFUA_7G04590* | Telomerase reverse transcriptase |
| *ETP1* | *YHL010C* | *AFUA_4G10360* | RING and UBP finger domain protein |
| *ETR1* | *YBR026C* | *AFUA_3G04150* | 2-enoyl thioester reductase |
| *EUG1* | *YCL043C YDR518W* | *AFUA_2G06150* | Protein disulfide isomerase Pdi1 |
| *EXO5* | *YBR163W* | *AFUA_1G13850* | Exo5p |
| *EXO70* | *YJL085W* | *AFUA_2G11960* | Exocyst complex component Exo70 |
| *EXO84* | *YBR102C* | *AFUA_6G11370* | Essential protein with dual roles in spliceosome assembly and exocytosis; the exocyst complex mediates polarized targeting of secretory vesicles to active sites of exocytosis |
| *FAA2* | *YER015W* | *AFUA_5G04270* | AMP-binding enzyme |
| *FAB1* | *YFR019W* | *AFUA_6G07440* | 1-phosphatidylinositol-3-phosphate 5-kinase; vacuolar membrane kinase that generates phosphatidylinositol (3,5)P2, which is involved in vacuolar sorting and homeostasis |
| *FAD1* | *YDL045C* | *AFUA_6G11040* | FAD synthetase |
| *FAL1* | *YDR021W* | *AFUA_5G02410* | Eukaryotic translation initiation factor eIF-4A subunit |
| *FAP1* | *YNL023C* | *AFUA_7G04710* | NF-X1 finger transcription factor |
| *FAP7* | *YDL166C* | *AFUA_5G05570* | Essential NTPase required for small ribosome subunit synthesis, mediates processing of the 20S pre-rRNA at site D in the cytoplasm but associates only transiently with 43S preribosomes via Rps14p, may be the endonuclease for site D |
| *FAR10* | *YLR238W YDR200C* | *AFUA_2G02420* | Protein required for cytoplasm to vacuole targeting of proteins; forms a complex with Far3p and Far7p to Far11p involved in recovery from pheromone-induced cell cycle arrest; localizes to the cytoplasm and endoplasmic reticulum membrane |
| *FAR11* | *YNL127W* | *AFUA_6G04250* | Pheromone-dependent cell cycle arrest protein Far11, putative |
| *FAR8* | *YMR029C* | *AFUA_5G01780* | Cell differentiation and development protein Fsr1 |
| *FAS1* | *YKL182W* | *AFUA_3G04220* | Beta subunit of fatty acid synthetase, which catalyzes the synthesis of long-chain saturated fatty acids; contains acetyltransacylase, dehydratase, enoyl reductase, malonyl transacylase, and palmitoyl transacylase activities |
| *FAS2* | *YPL231W* | *AFUA_3G04210* | Alpha subunit of fatty acid synthetase, which catalyzes the synthesis of long-chain saturated fatty acids; contains the acyl-carrier protein domain and beta-ketoacyl reductase, beta-ketoacyl synthase and self-pantetheinylation activities |
| *FAT1* | *YBR041W* | *AFUA_6G03630 AFUA_6G07270* | Very-long-chain acyl-CoA synthetase family protein (CefD1) |
| *FBA1* | *YKL060C* | *AFUA_3G11690* | Fructose-bisphosphate aldolase, class II |
| *FBP1* | *YLR377C* | *AFUA_4G11310* | Fructose-1,6-bisphosphatase Fbp1 |
| *FBP26* | *YJL155C* | *AFUA_7G05800* | Fructose-2,6-bisphosphatase |
| *FCF1* | *YDR339C* | *AFUA_2G03340* | DUF652 domain protein |
| *FCF2* | *YLR051C* | *AFUA_6G05310* | Essential nucleolar protein involved in the early steps of 35S rRNA processing; interacts with Faf1p; member of a transcriptionally co-regulated set of genes called the RRB regulon |
| *FCJ1* | *YKR016W* | *AFUA_4G08030* | Fcj1p |
| *FCP1* | *YMR277W* | *AFUA_3G11410* | Carboxy-terminal domain phosphatase, essential for dephosphorylation of the repeated C-terminal domain of the RNA polymerase II large subunit (Rpo21p) |
| *FES1* | *YBR101C* | *AFUA_6G04750* | Hsp70 nucleotide exchange factor (Fes1) |
| *FET3* | *YMR058W* | *AFUA_5G03790* | Ferro-O2-oxidoreductase required for high-affinity iron uptake and involved in mediating resistance to copper ion toxicity, belongs to class of integral membrane multicopper oxidases |
| *FET4* | *YMR319C* | *AFUA_4G14640* | Low affinity iron transporter |
| *FIP1* | *YJR093C* | *AFUA_6G10690* | Cleavage and polyadenylylation specificity factor subunit Fip1 |
| *FIS1* | *YIL065C* | *AFUA_2G13320* | Mitochondrial membrane fission protein (Fis1) |
| *FKH1* | *YIL131C YNL068C* | *AFUA_3G11960* | Forkhead transcription factor Fkh1/2 |
| *FKH2* | *YNL068C* | *AFUA_5G05600* | Forkhead transcription factor (Sep1) |
| *FKS1* | *YGR032W YLR342W* | *AFUA_6G12400* | Catalytic subunit of 1,3-beta-glucan synthase, involved in formation of the inner layer of the spore wall; activity positively regulated by Rho1p and negatively by Smk1p; has similarity to an alternate catalytic subunit, Fks1p (Gsc1p) |
| *FLC1* | *YGL139W YPL221W YOR365C YAL053W* | *AFUA_2G17650 AFUA_4G13340* | Putative protein of unknown function; YOR365C is not an essential protein |
| *FLX1* | *YIL134W* | *AFUA_6G05170* | Mitochondrial folate carrier protein Flx1 |
| *FMN1* | *YDR236C* | *AFUA_2G05820* | Riboflavin kinase |
| *FMP21* | *YBR269C* | *AFUA_2G12460* | Fmp21p |
| *FMP25* | *YLR077W* | *AFUA_3G11100* | Mitochondrial protein Fmp25 |
| *FMP27* | *YLR454W* | *AFUA_2G16420* | Fmp27p |
| *FMP30* | *YPL103C* | *AFUA_3G13010* | Zn-dependent hydrolase/oxidoreductase family protein, putative |
| *FMP32* | *YFL046W* | *AFUA_6G06570* | Fmp32p |
| *FMP37* | *YGL080W* | *AFUA_4G07750* | UPF0041 domain protein |
| *FMP40* | *YPL222W* | *AFUA_1G07640* | YdiU domain protein |
| *FMP41* | *YNL168C* | *AFUA_3G08140* | Fumarylacetoacetate hydrolase family protein |
| *FMP43* | *YGR243W YHR162W* | *AFUA_6G02970* | Putative protein of unknown function; green fluorescent protein (GFP)-fusion protein localizes to the mitochondrion |
| *FMP52* | *YER004W* | *AFUA_6G08930* | Fmp52p |
| *FMT1* | *YBL013W* | *AFUA_6G06920* | Methionyl-tRNA formyltransferase family protein, putative |
| *FOX2* | *YKR009C* | *AFUA_4G03900* | Peroxisomal multifunctional beta-oxidation protein (MFP) |
| *FPK1* | *YCR091W YNR047W* | *AFUA_5G11520* | Serine/threonine protein kinase (Nrc-2) |
| *FPR1* | *YNL135C* | *AFUA_6G12170* | FKBP-type peptidyl-prolyl isomerase |
| *FPR2* | *YDR519W* | *AFUA_2G03870* | FKBP-type peptidyl-prolyl isomerase |
| *FRA1* | *YLL029W* | *AFUA_5G08050* | Aminopeptidase P |
| *FRA2* | *YGL220W* | *AFUA_6G12490* | BolA domain protein |
| *FRE1* | *YLR214W YLL051C YOR384W YNR060W YOR381W YKL220C* | *AFUA_1G17270 AFUA_8G01310* | Ferric reductase and cupric reductase, reduces siderophore-bound iron and oxidized copper prior to uptake by transporters; expression induced by low copper and iron levels |
| *FRE7* | *YOL152W* | *AFUA_6G02170* | Metalloreductase transmembrane component |
| *FRM2* | *YCL026C-B YCL026C-A* | *AFUA_4G09920 AFUA_5G09910* | Nitroreductase family protein |
| *FRQ1* | *YDR373W* | *AFUA_6G14240* | Calcium sensor (NCS-1) |
| *FRS1* | *YLR060W* | *AFUA_1G05620* | Phenylalanyl-tRNA synthetase, beta subunit |
| *FRS2* | *YFL022C* | *AFUA_2G03580* | Phenylalanyl-tRNA synthetase beta chain cytoplasmic |
| *FTR1* | *YER145C* | *AFUA_5G03800* | High-affinity iron transporter FtrA |
| *FUM1* | *YPL262W* | *AFUA_6G02470* | Fumarate hydratase |
| *FUN12* | *YAL035W* | *AFUA_1G03970* | Mitochondrial translation initiation factor IF-2, putative |
| *FUN19* | *YOR338W YAL034C* | *AFUA_1G07750* | Putative protein of unknown function; YOR338W transcription is regulated by Azf1p and its transcript is a specific target of the G protein effector Scp160p; identified as being required for sporulation in a high-throughput mutant screen |
| *FUN26* | *YAL022C* | *AFUA_4G09990* | Nucleoside transporter family |
| *FUN30* | *YAL019W* | *AFUA_3G08400* | SNF2 family helicase/ATPase |
| *FUR1* | *YHR128W* | *AFUA_5G05460* | Cytosine deaminase-uracil phosphoribosyltransferase fusion protein |
| *FUS3* | *YBL016W* | *AFUA_6G12820* | MAP kinase MpkB |
| *FYV10* | *YIL097W* | *AFUA_5G05720* | Negative regulation of gluconeogenesis |
| *FYV4* | *YHR059W* | *AFUA_7G03730* | Fyv4p |
| *FZO1* | *YBR179C* | *AFUA_5G13392* | Transmembrane GTPase Fzo1 |
| *GAA1* | *YLR088W* | *AFUA_6G12760* | GPI transamidase component (GAA1) |
| *GAB1* | *YLR459W* | *AFUA_4G08200* | GPI transamidase subunit, involved in attachment of glycosylphosphatidylinositol anchors to proteins; may have a role in recognition of the attachment signal or of the lipid portion of GPI |
| *GAL10* | *YBR019C* | *AFUA_5G10780* | UDP-glucose 4-epimerase |
| *GAL2* | *YDL245C YJR158W YEL069C YNR072W YFL011W YMR011W YJL214W YOL156W YJL219W YHR096C YHR094C YDR345C YLR081W YHR092C YDR342C YDR343C* | *AFUA_7G00950 AFUA_2G11520 AFUA_5G01160* | High-affinity glucose transporter of the major facilitator superfamily, expression induced by low levels of glucose and repressed by high levels of glucose |
| *GAL4* | *YPL248C* | *AFUA_1G11000 AFUA_3G06740* | DNA-binding transcription factor required for the activation of the GAL genes in response to galactose; repressed by Gal80p and activated by Gal3p |
| *GAL7* | *YBR018C* | *AFUA_2G11560* | Galactose-1-phosphate uridylyltransferase |
| *GAP1* | *YKR039W* | *AFUA_7G04290* | Amino acid permease (Gap1) |
| *GAR1* | *YHR089C* | *AFUA_4G13690* | SnoRNP protein (gar1) |
| *GAS1* | *YMR307W* | *AFUA_6G12410 AFUA_8G02130 AFUA_2G05340 AFUA_2G12850* | 1,3-beta-glucanosyltransferase |
| *GAS4* | *YOL132W* | *AFUA_6G11390* | 1,3-beta-glucanosyltransferase, involved with Gas2p in spore wall assembly; has similarity to Gas1p; localizes to the cell wall |
| *GAS5* | *YOL030W* | *AFUA_2G01170* | 1,3-beta-glucanosyltransferase, has similarity to Gas1p; localizes to the cell wall |
| *GAT1* | *YER040W YFL021W* | *AFUA_6G01970* | GATA transcriptional activator AreA |
| *GAT2* | *YMR136W* | *AFUA_3G13870* | GATA-type sexual development transcription factor NsdD |
| *GCD1* | *YOR260W* | *AFUA_1G16660* | Gamma subunit of the translation initiation factor eIF2B, the guanine-nucleotide exchange factor for eIF2; activity subsequently regulated by phosphorylated eIF2; first identified as a negative regulator of GCN4 expression |
| *GCD10* | *YNL062C* | *AFUA_5G01850* | Eukaryotic translation initiation factor 3, gamma subunit |
| *GCD11* | *YER025W* | *AFUA_4G07580* | Gamma subunit of the translation initiation factor eIF2, involved in the identification of the start codon; binds GTP when forming the ternary complex with GTP and tRNAi-Met |
| *GCD2* | *YGR083C* | *AFUA_5G13040* | Delta subunit of the translation initiation factor eIF2B, the guanine-nucleotide exchange factor for eIF2; activity subsequently regulated by phosphorylated eIF2; first identified as a negative regulator of GCN4 expression |
| *GCD6* | *YDR211W* | *AFUA_6G12530* | Catalytic epsilon subunit of the translation initiation factor eIF2B, the guanine-nucleotide exchange factor for eIF2; activity subsequently regulated by phosphorylated eIF2; first identified as a negative regulator of GCN4 expression |
| *GCD7* | *YLR291C* | *AFUA_1G09450* | Beta subunit of the translation initiation factor eIF2B, the guanine-nucleotide exchange factor for eIF2; activity subsequently regulated by phosphorylated eIF2; first identified as a negative regulator of GCN4 expression |
| *GCN1* | *YGL195W* | *AFUA_2G07960* | Translational activator |
| *GCN2* | *YDR283C* | *AFUA_5G06750* | Protein kinase (Gcn2) |
| *GCN20* | *YFR009W* | *AFUA_4G06070* | Translation initiation regulator (Gcn20) |
| *GCN3* | *YKR026C* | *AFUA_5G11340* | Alpha subunit of the translation initiation factor eIF2B, the guanine-nucleotide exchange factor for eIF2; activity subsequently regulated by phosphorylated eIF2; first identified as a positive regulator of GCN4 expression |
| *GCN4* | *YEL009C* | *AFUA_4G12470* | Basic leucine zipper transcriptional activator of amino acid biosynthetic genes in response to amino acid starvation; expression is tightly regulated at both the transcriptional and translational levels |
| *GCN5* | *YGR252W* | *AFUA_4G12650* | Histone acetyltransferase (Gcn5) |
| *GCS1* | *YDL226C* | *AFUA_5G07130* | ADP-ribosylation factor GTPase activating protein (ARF GAP), involved in ER-Golgi transport; shares functional similarity with Glo3p |
| *GCV1* | *YDR019C* | *AFUA_1G10780* | Glycine cleavage system T protein |
| *GCV2* | *YMR189W* | *AFUA_4G03760* | Glycine dehydrogenase |
| *GCV3* | *YAL044C* | *AFUA_1G12070* | Glycine cleavage system H protein |
| *GDA1* | *YEL042W* | *AFUA_1G12150* | Nucleoside diphosphatase Gda1 |
| *GDE1* | *YPL110C* | *AFUA_5G11590* | Glycerophosphocholine phosphodiesterase Gde1 |
| *GDH2* | *YDL215C* | *AFUA_2G06000* | NAD+ dependent glutamate dehydrogenase |
| *GDI1* | *YER136W* | *AFUA_2G11150* | GDP dissociation inhibitor, regulates vesicle traffic in secretory pathways by regulating the dissociation of GDP from the Sec4/Ypt/rab family of GTP binding proteins |
| *GDT1* | *YBR187W* | *AFUA_3G07080* | UPF0016 domain protein |
| *GEA1* | *YEL022W YJR031C* | *AFUA_5G11900* | Guanine nucleotide exchange factor (Gea2) |
| *GEF1* | *YJR040W* | *AFUA_2G08900 AFUA_2G12140 AFUA_5G10630* | Voltage-gated chloride channel (ClcA) |
| *GEM1* | *YAL048C* | *AFUA_6G07870* | Evolutionarily-conserved tail-anchored outer mitochondrial membrane GTPase which regulates mitochondrial morphology; cells lacking Gem1p contain collapsed, globular, or grape-like mitochondria; not required for pheromone-induced cell death |
| *GEP3* | *YOR205C* | *AFUA_6G12640* | Gep3p |
| *GEP4* | *YHR100C* | *AFUA_4G10230* | Gep4p |
| *GET1* | *YGL020C* | *AFUA_4G06340* | Retrograde vesicle-mediated transport protein Get1, putative |
| *GET3* | *YDL100C* | *AFUA_3G11350* | Arsenite translocating ATPase ArsA |
| *GET4* | *YOR164C* | *AFUA_6G05050* | DUF410 domain protein |
| *GFA1* | *YKL104C* | *AFUA_6G06340* | Glucosamine-fructose-6-phosphate aminotransferase |
| *GFD2* | *YCL036W YDR514C* | *AFUA_3G11950* | Putative protein of unknown function |
| *GGA1* | *YHR108W YDR358W* | *AFUA_1G06680* | VHS domain protein |
| *GGC1* | *YDL198C* | *AFUA_1G07450* | Mitochondrial GTP/GDP transporter Ggc1 |
| *GID7* | *YCL039W* | *AFUA_5G02400* | Catabolite degradation protein |
| *GID8* | *YMR135C* | *AFUA_2G05440* | Gid8p |
| *GIM4* | *YEL003W* | *AFUA_1G03830* | Prefoldin subunit 2 |
| *GIP3* | *YJL042W YOR227W YPL137C* | *AFUA_1G12900* | Cell wall biogenesis protein Mhp1 |
| *GIR2* | *YDR152W* | *AFUA_4G12100* | RWD domain protein (Gir2) |
| *GIT1* | *YCR098C* | *AFUA_6G07750* | MFS phospholipid transporter (Git1) |
| *GLC3* | *YEL011W* | *AFUA_5G10540* | Glycogen branching enzyme GbeA |
| *GLC7* | *YER133W* | *AFUA_1G04950* | Serine/threonine protein phosphatase PP1 |
| *GLE1* | *YDL207W* | *AFUA_1G11440* | Cytoplasmic nucleoporin required for polyadenylated RNA export but not for protein import; component of Nup82p nuclear pore subcomplex; contains a nuclear export signal |
| *GLE2* | *YER107C* | *AFUA_1G09020* | Component of the Nup82 subcomplex of the nuclear pore complex; required for polyadenylated RNA export but not for protein import; homologous to S. pombe Rae1p |
| *GLN1* | *YPR035W* | *AFUA_4G13120* | Glutamine synthetase |
| *GLN4* | *YOR168W* | *AFUA_2G01920* | Glutaminyl-tRNA synthetase |
| *GLO2* | *YOR040W YDR272W* | *AFUA_5G12840* | Cytoplasmic glyoxalase II, catalyzes the hydrolysis of S-D-lactoylglutathione into glutathione and D-lactate |
| *GLO3* | *YER122C* | *AFUA_2G09830* | ADP-ribosylation factor GTPase activating protein (ARF GAP), involved in ER-Golgi transport; shares functional similarity with Gcs1p |
| *GLR1* | *YPL091W* | *AFUA_1G15960* | Cytosolic and mitochondrial glutathione oxidoreductase, converts oxidized glutathione to reduced glutathione; mitochondrial but not cytosolic form has a role in resistance to hyperoxia |
| *GMH1* | *YKR030W* | *AFUA_3G05690* | Integral membrane protein |
| *GNA1* | *YFL017C* | *AFUA_6G02460* | Evolutionarily conserved glucosamine-6-phosphate acetyltransferase required for multiple cell cycle events including passage through START, DNA synthesis, and mitosis; involved in UDP-N-acetylglucosamine synthesis, forms GlcNAc6P from AcCoA |
| *GNT1* | *YOR320C* | *AFUA_8G02690* | AlphaN-acetylglucosamine transferase |
| *GOS1* | *YHL031C* | *AFUA_1G10497* | Vesicle transport v-SNARE protein Gos1 |
| *GOT1* | *YMR292W* | *AFUA_4G03040* | Got1 family protein |
| *GPD1* | *YOL059W YDL022W* | *AFUA_1G02150 AFUA_2G08250* | Glycerol 3-phosphate dehydrogenase (GfdA) |
| *GPH1* | *YPR160W* | *AFUA_1G12920* | Glycogen phosphorylase GlpV/Gph1 |
| *GPI1* | *YGR216C* | *AFUA_3G09860* | N-acetylglucosaminyl transferase component Gpi1 |
| *GPI12* | *YMR281W* | *AFUA_5G12550* | ER membrane protein involved in the second step of glycosylphosphatidylinositol anchor assembly, the de-N-acetylation of the N-acetylglucosaminylphosphatidylinositol intermediate; functional homolog of human PIG-Lp |
| *GPI13* | *YLL031C* | *AFUA_6G04290* | ER membrane localized phosphoryltransferase that adds phosphoethanolamine onto the third mannose residue of the glycosylphosphatidylinositol anchor precursor; similar to human PIG-O protein |
| *GPI14* | *YJR013W* | *AFUA_7G01300* | Glycosylphosphatidylinositol-alpha 1,4 mannosyltransferase I, involved in GPI anchor biosynthesis, requires Pbn1p for function; homolog of mammalian PIG-M |
| *GPI16* | *YHR188C* | *AFUA_6G09020* | GPI transamidase component Gpi16 |
| *GPI17* | *YDR434W* | *AFUA_6G07180* | GPI transamidase component PIG-S |
| *GPI18* | *YBR004C* | *AFUA_1G14140 AFUA_4G11280* | Functional ortholog of human PIG-V, which is a mannosyltransferase that transfers the second mannose in glycosylphosphatidylinositol biosynthesis; the authentic, non-tagged protein was localized to mitochondria |
| *GPI19* | *YDR437W* | *AFUA_5G01810* | Gpi19p |
| *GPI2* | *YPL076W* | *AFUA_3G07170* | Phosphatidylinositol:UDP-GlcNAc transferase PIG-C |
| *GPI8* | *YDR331W* | *AFUA_1G15130* | ER membrane glycoprotein subunit of the glycosylphosphatidylinositol transamidase complex that adds glycosylphosphatidylinositol anchors to newly synthesized proteins; human PIG-K protein is a functional homolog |
| *GPX1* | *YKL026C YBR244W YIR037W* | *AFUA_3G12270* | Glutathione peroxidase Hyr1 |
| *GRC3* | *YLL035W* | *AFUA_2G02190* | RNA processing protein Grc3 |
| *GRE3* | *YHR104W* | *AFUA_1G04820* | Aldose reductase involved in methylglyoxal, d-xylose, arabinose, and galactose metabolism; stress induced (osmotic, ionic, oxidative, heat shock, starvation and heavy metals); regulated by the HOG pathway |
| *GRH1* | *YDR517W* | *AFUA_6G08620* | Acetylated, cis-golgi localized protein involved in ER to Golgi transport; homolog of human GRASP65; forms a complex with the coiled-coil protein Bug1p; mutants are compromised for the fusion of ER-derived vesicles with Golgi membranes |
| *GRR1* | *YJR090C* | *AFUA_1G05970* | F-box protein component of the SCF ubiquitin-ligase complex; involved in carbon catabolite repression, glucose-dependent divalent cation transport, high-affinity glucose transport, morphogenesis, and sulfite detoxification |
| *GRS1* | *YBR121C* | *AFUA_5G05920* | Glycyl-tRNA synthetase |
| *GRX1* | *YCL035C YDR513W* | *AFUA_1G06100* | Cytoplasmic glutaredoxin, thioltransferase, glutathione-dependent disulfide oxidoreductase involved in maintaining redox state of target proteins, also exhibits glutathione peroxidase activity, expression induced in response to stress |
| *GSH1* | *YJL101C* | *AFUA_3G13900* | Gamma glutamylcysteine synthetase catalyzes the first step in glutathione biosynthesis; expression induced by oxidants, cadmium, and mercury |
| *GSP1* | *YLR293C YOR185C* | *AFUA_6G13300* | GTP binding protein involved in the maintenance of nuclear organization, RNA processing and transport; interacts with Kap121p, Kap123p and Pdr6p (karyophilin betas); Gsp1p homolog that is not required for viability |
| *GSY1* | *YFR015C YLR258W* | *AFUA_5G02480* | Glycogen synthase, similar to Gsy1p; expression induced by glucose limitation, nitrogen starvation, heat shock, and stationary phase; activity regulated by cAMP-dependent, Snf1p and Pho85p kinases as well as by the Gac1p-Glc7p phosphatase |
| *GTB1* | *YDR221W* | *AFUA_7G04110* | Glucosidase II beta subunit, forms a complex with alpha subunit Rot2p, involved in removal of two glucose residues from N-linked glycans during glycoprotein biogenesis in the ER |
| *GTR2* | *YGR163W* | *AFUA_1G16500* | Small monomeric GTPase (Gtr2) |
| *GTS1* | *YGL181W* | *AFUA_4G09120* | Arf3p GTPase Activating Protein that localizes to endocytic patches; gts1 mutations affect budding, cell size, heat tolerance, sporulation, life span, ultradian rhythms; localizes to nucleus and induces flocculation when overexpressed |
| *GTT1* | *YIR038C* | *AFUA_2G17300 AFUA_1G17010 AFUA_8G02500* | Glutathione S-transferase |
| *GTT2* | *YLL060C* | *AFUA_2G04240* | Glutathione S-transferase capable of homodimerization; functional overlap with Gtt2p, Grx1p, and Grx2p |
| *GUD1* | *YDL238C* | *AFUA_4G03770* | Guanine deaminase, a catabolic enzyme of the guanine salvage pathway producing xanthine and ammonia from guanine; activity is low in exponentially-growing cultures but expression is increased in post-diauxic and stationary-phase cultures |
| *GUP1* | *YGL084C YPL189W* | *AFUA_2G05040* | Glycerol:H+ symporter (Gup1) |
| *GUT2* | *YIL155C* | *AFUA_1G08810* | Glycerol-3-phosphate dehydrogenase, mitochondrial |
| *GVP36* | *YIL041W* | *AFUA_2G11475* | BAR domain-containing protein that localizes to both early and late Golgi vesicles; required for adaptation to varying nutrient concentrations, fluid-phase endocytosis, polarization of the actin cytoskeleton, and vacuole biogenesis |
| *GWT1* | *YJL091C* | *AFUA_1G14870* | GPI anchor biosynthesis protein Gwt1 |
| *GYL1* | *YMR192W YPL249C* | *AFUA_4G10600* | GTPase-activating protein for yeast Rab family members, involved in ER to Golgi trafficking; exhibits GAP activity toward Ypt1p that is stimulated by Gyl1p, also acts on Sec4p; interacts with Gyl1p, Rvs161p and Rvs167p |
| *GYP7* | *YDL234C* | *AFUA_6G03940* | GTPase-activating protein for yeast Rab family members including: Ypt7p (most effective), Ypt1p, Ypt31p, and Ypt32p (in vitro); involved in vesicle mediated protein trafficking |
| *GYP8* | *YFL027C* | *AFUA_1G04010* | GTPase-activating protein for yeast Rab family members; Ypt1p is the preferred in vitro substrate but also acts on Sec4p, Ypt31p and Ypt32p; involved in the regulation of ER to Golgi vesicle transport |
| *HAC1* | *YFL031W* | *AFUA_3G04070* | Basic leucine zipper transcription factor that regulates the unfolded protein response, via UPRE binding, and membrane biogenesis; ER stress-induced splicing pathway facilitates efficient Hac1p synthesis |
| *HAL9* | *YBR150C YOL089C* | *AFUA_6G01960 AFUA_6G02330* | Putative protein of unknown function; the authentic, non-tagged protein is detected in highly purified mitochondria in high-throughput studies |
| *HAP2* | *YGL237C* | *AFUA_2G14720* | CCAAT-binding transcription factor subunit HAPB |
| *HAP3* | *YBL021C* | *AFUA_1G03840* | CCAAT-binding factor complex subunit HapC |
| *HAP5* | *YOR358W* | *AFUA_6G05300* | CCAAT-binding factor complex subunit HapE |
| *HAT1* | *YPL001W* | *AFUA_2G12030* | Histone acetyltransferase type b catalytic subunit, putative |
| *HAT2* | *YEL056W* | *AFUA_5G03130* | Chromatin assembly factor 1 subunit C |
| *HBS1* | *YKR084C* | *AFUA_2G04630* | GTP binding protein with sequence similarity to the elongation factor class of G proteins, EF-1alpha and Sup35p; associates with Dom34p, and shares a similar genetic relationship with genes that encode ribosomal protein components |
| *HCM1* | *YCR065W* | *AFUA_5G05600* | Forkhead transcription factor that drives S-phase specific expression of genes involved in chromosome segregation, spindle dynamics, and budding; suppressor of calmodulin mutants with specific SPB assembly defects; telomere maintenance role |
| *HDA1* | *YNL021W* | *AFUA_5G01980* | Histone deacetylase HdaA |
| *HDA3* | *YPR179C* | *AFUA_5G03390* | Hda3p |
| *HEH2* | *YML034W YDR458C* | *AFUA_6G08530* | Inner nuclear membrane protein that functions in regulation of subtelomeric genes and is linked to TREX factors; SRC1 produces 2 splice variant proteins with different functions; possible role in chromatid segregation |
| *HEM1* | *YDR232W* | *AFUA_5G06270* | 5-aminolevulinate synthase, catalyzes the first step in the heme biosynthetic pathway; an N-terminal signal sequence is required for localization to the mitochondrial matrix; expression is regulated by Hap2p-Hap3p |
| *HEM12* | *YDR047W* | *AFUA_1G05060* | Uroporphyrinogen decarboxylase |
| *HEM13* | *YDR044W* | *AFUA_1G07480* | Coproporphyrinogen III oxidase, an oxygen requiring enzyme that catalyzes the sixth step in the heme biosynthetic pathway; transcription is repressed by oxygen and heme (via Rox1p and Hap1p) |
| *HEM14* | *YER014W* | *AFUA_6G08440* | Protoporphyrinogen oxidase |
| *HEM15* | *YOR176W* | *AFUA_5G07750* | Ferrochelatase, a mitochondrial inner membrane protein, catalyzes the insertion of ferrous iron into protoporphyrin IX, the eighth and final step in the heme biosynthetic pathway |
| *HEM2* | *YGL040C* | *AFUA_1G08760* | Aminolevulinate dehydratase, a homo-octameric enzyme, catalyzes the conversion of 5-aminolevulinate to porphobilinogen, the second step in heme biosynthesis; enzymatic activity is zinc-dependent; localizes to the cytoplasm and nucleus |
| *HEM3* | *YDL205C* | *AFUA_3G13120 AFUA_5G11760* | Porphobilinogen deaminase Hem3 |
| *HFD1* | *YMR110C* | *AFUA_4G13500* | Aldehyde dehydrogenase |
| *HFI1* | *YPL254W* | *AFUA_2G06060* | Adaptor protein required for structural integrity of the SAGA complex, a histone acetyltransferase-coactivator complex that is involved in global regulation of gene expression through acetylation and transcription functions |
| *HFM1* | *YGL251C* | *AFUA_6G13080* | DEAD/DEAH box DNA helicase (Mer3) |
| *HGH1* | *YGR187C* | *AFUA_3G04260* | DNA-binding protein HGH1 |
| *HHF1* | *YBR009C YNL030W* | *AFUA_1G13780* | Histone H4.1 |
| *HHO1* | *YPL127C* | *AFUA_3G06070* | Histone H1 |
| *HHT1* | *YBR010W YNL031C* | *AFUA_1G13790* | Histone H3 |
| *HIR1* | *YBL008W* | *AFUA_1G09790* | Histone transcription regulator Hir1 |
| *HIS3* | *YOR202W* | *AFUA_6G04700* | Imidazoleglycerol-phosphate dehydratase |
| *HKR1* | *YGR014W YDR420W* | *AFUA_4G04070* | Mucin family signaling protein Msb2 |
| *HMF1* | *YER057C YIL051C* | *AFUA_7G02340* | Mitochondrial protein involved in maintenance of the mitochondrial genome |
| *HMG1* | *YLR450W YML075C* | *AFUA_1G11230 AFUA_2G03700* | HMG-CoA reductase |
| *HMO1* | *YDR174W* | *AFUA_1G04550* | Chromatin associated high mobility group family member involved in genome maintenance; rDNA-binding component of the Pol I transcription system; associates with a 5'-3' DNA helicase and Fpr1p, a prolyl isomerase |
| *HMS1* | *YOR032C* | *AFUA_2G01260* | Basic helix-loop-helix protein with similarity to myc-family transcription factors; overexpression confers hyperfilamentous growth and suppresses the pseudohyphal filamentation defect of a diploid mep1 mep2 homozygous null mutant |
| *HMT1* | *YBR034C* | *AFUA_1G06190* | Histone H4 arginine methyltransferase RmtA |
| *HNM1* | *YGL077C* | *AFUA_5G02940* | Choline/ethanolamine transporter; involved in the uptake of nitrogen mustard and the uptake of glycine betaine during hypersaline stress; co-regulated with phospholipid biosynthetic genes and negatively regulated by choline and myo-inositol |
| *HNT2* | *YDR305C* | *AFUA_2G11700* | Dinucleoside triphosphate hydrolase; has similarity to the tumor suppressor FHIT and belongs to the histidine triad superfamily of nucleotide-binding proteins |
| *HNT3* | *YOR258W* | *AFUA_2G16130* | DNA 5' AMP hydrolase involved in DNA repair; member of the histidine triad superfamily of nucleotide-binding proteins; homolog of Aprataxin, a Hint related protein that is mutated in individuals with ataxia with oculomotor apraxia |
| *HOC1* | *YJR075W* | *AFUA_5G08580* | Alpha-1,6-mannosyltransferase involved in cell wall mannan biosynthesis; subunit of a Golgi-localized complex that also contains Anp1p, Mnn9p, Mnn11p, and Mnn10p; identified as a suppressor of a cell lysis sensitive pkc1-371 allele |
| *HOG1* | *YLR113W* | *AFUA_1G12940* | MAP kinase SakA |
| *HOL1* | *YNR055C* | *AFUA_5G00430 AFUA_2G02040 AFUA_8G04702* | MFS transporter |
| *HOP1* | *YIL072W* | *AFUA_6G13050* | Meiosis specific protein Hop1 |
| *HOS3* | *YPL116W* | *AFUA_4G04290* | Histone deacetylase HosB |
| *HOS4* | *YIL112W* | *AFUA_1G05490* | Histone deacetylase complex subunit (Hos4) |
| *HPF1* | *YIL169C YOL155C* | *AFUA_1G00250* | Putative protein of unknown function; serine/threonine rich and highly similar to YOL155C, a putative glucan alpha-1,4-glucosidase; transcript is induced in both high and low pH environments; YIL169C is a non-essential gene |
| *HRD1* | *YOL013C* | *AFUA_8G04840* | RING finger protein |
| *HRD3* | *YLR207W* | *AFUA_1G14690* | Ubiquitin-protein ligase Sel1/Ubx2 |
| *HRK1* | *YOR267C* | *AFUA_5G11840* | Protein kinase |
| *HRP1* | *YOL123W* | *AFUA_2G06090* | Heterogeneous nuclear ribonucleoprotein HRP1 |
| *HRQ1* | *YDR291W* | *AFUA_2G10820* | DEAD/DEAH box helicase |
| *HRR25* | *YPL204W* | *AFUA_2G02530* | Casein kinase I |
| *HRT1* | *YOL133W* | *AFUA_5G05790* | Ubiquitin ligase subunit HrtA |
| *HRT3* | *YLR097C* | *AFUA_2G09240* | F-box protein (Pof7) |
| *HSC82* | *YMR186W YPL240C* | *AFUA_5G04170* | Cytoplasmic chaperone of the Hsp90 family, redundant in function and nearly identical with Hsp82p, and together they are essential; expressed constitutively at 10-fold higher basal levels than HSP82 and induced 2-3 fold by heat shock |
| *HSF1* | *YGL073W* | *AFUA_5G01900* | Heat shock transcription factor Hsf1 |
| *HSH155* | *YMR288W* | *AFUA_2G13780* | Splicing factor 3B subunit 1 |
| *HSH49* | *YOR319W* | *AFUA_6G05180* | Splicing factor 3b subunit 4 |
| *HSL1* | *YKL101W* | *AFUA_6G02300* | Serine/threonine protein kinase (Kcc4) |
| *HSP10* | *YOR020C* | *AFUA_6G10700* | Chaperonin |
| *HSP104* | *YLL026W* | *AFUA_1G15270* | Heat shock protein that cooperates with Ydj1p and Ssa1p to refold and reactivate previously denatured, aggregated proteins; responsive to stresses including: heat, ethanol, and sodium arsenite; involved in [PSI+] propagation |
| *HSP30* | *YCR021C YBR054W YDR033W* | *AFUA_7G01430* | Putative protein of unknown function; the authentic, non-tagged protein is detected in a phosphorylated state in highly purified mitochondria in high-throughput studies; transcriptionally regulated by Haa1p |
| *HSP31* | *YDR533C YMR322C YOR391C YPL280W* | *AFUA_3G01210 AFUA_3G08490 AFUA_5G01430* | ThiJ/PfpI family protein |
| *HSP60* | *YLR259C* | *AFUA_2G09290* | Antigenic mitochondrial protein HSP60 |
| *HSP78* | *YDR258C* | *AFUA_1G11180* | Heat shock protein/chaperonin HSP78 |
| *HSV2* | *YGR223C* | *AFUA_5G08530* | Phosphatidylinositol 3,5-bisphosphate-binding protein, putative |
| *HTA1* | *YBL003C YDR225W* | *AFUA_3G05360* | Histone H2A, core histone protein required for chromatin assembly and chromosome function; one of two nearly identical subtypes (see also HTA2); DNA damage-dependent phosphorylation by Mec1p facilitates DNA repair; acetylated by Nat4p |
| *HTB1* | *YBL002W YDR224C* | *AFUA_3G05350* | Histone H2B, core histone protein required for chromatin assembly and chromosome function; nearly identical to HTB1; Rad6p-Bre1p-Lge1p mediated ubiquitination regulates transcriptional activation, meiotic DSB formation and H3 methylation |
| *HTD2* | *YHR067W* | *AFUA_5G05700 AFUA_6G07250* | Htd2p |
| *HTS1* | *YPR033C* | *AFUA_4G12920* | Histidyl-tRNA synthetase, mitochondrial precursor |
| *HTZ1* | *YOL012C* | *AFUA_5G01950* | H2AZ; H2A.F/Z |
| *HUB1* | *YNR032C-A* | *AFUA_3G13370* | Ubiquitin-like protein (HubA) |
| *HUF* | *YOR110W YNL108C* | *AFUA_1G15740* | Putative protein of unknown function with similarity to Tfc7p and prokaryotic phosphotransfer enzymes; null mutant shows alterations in glucose metabolism; GFP-fusion protein localizes to the cytoplasm and nucleus |
| *HUL4* | *YJR036C* | *AFUA_1G04640* | Ubiquitin-protein ligase (Hul4) |
| *HUL5* | *YGL141W* | *AFUA_1G04210* | IQ and HECT domain protein |
| *HUT1* | *YPL244C* | *AFUA_1G05440* | UDP-Glc/Gal endoplasmic reticulum nucleotide sugar transporter |
| *HXK1* | *YFR053C YGL253W* | *AFUA_2G05910* | Hexokinase isoenzyme 2 that catalyzes phosphorylation of glucose in the cytosol; predominant hexokinase during growth on glucose; functions in the nucleus to repress expression of HXK1 and GLK1 and to induce expression of its own gene |
| *IBA57* | *YJR122W* | *AFUA_5G12430* | Aminomethyl transferase |
| *ICE2* | *YIL090W* | *AFUA_1G08970* | Ice2p |
| *ICL1* | *YER065C* | *AFUA_4G13510* | Isocitrate lyase AcuD |
| *ICP55* | *YER078C* | *AFUA_6G09190* | Metallopeptidase family M24 |
| *IDH1* | *YNL037C* | *AFUA_6G06370* | NAD(+)-isocitrate dehydrogenase subunit I |
| *IDH2* | *YOR136W* | *AFUA_1G12800* | Isocitrate dehydrogenase, NAD-dependent |
| *IDI1* | *YPL117C* | *AFUA_6G11160* | Isopentenyl-diphosphate delta-isomerase |
| *IDP1* | *YDL066W* | *AFUA_3G08660* | Isocitrate dehydrogenase Idp1 |
| *IES1* | *YFL013C* | *AFUA_4G07670* | INO80 chromatin remodeling complex (Ies1) |
| *IES2* | *YNL215W* | *AFUA_2G12960* | PAPA-1-like conserved region protein |
| *IES6* | *YEL044W* | *AFUA_4G03030* | Ies6p |
| *IFM1* | *YOL023W* | *AFUA_1G06520* | Mitochondrial translation initiation factor |
| *IKI1* | *YHR187W* | *AFUA_6G04170* | Killer toxin sensitivity protein (Iki1) |
| *IKI3* | *YLR384C* | *AFUA_4G07140* | Killer toxin sensitivity protein (IKI3) |
| *IKS1* | *YJL057C* | *AFUA_4G08920* | Protein kinase |
| *ILS1* | *YBL076C* | *AFUA_1G13710* | Isoleucyl-tRNA synthetase ,cytoplasmic |
| *ILV1* | *YER086W* | *AFUA_4G08140* | Threonine dehydratase, biosynthetic |
| *ILV2* | *YMR108W* | *AFUA_3G10310* | Acetolactate synthase, catalyses the first common step in isoleucine and valine biosynthesis and is the target of several classes of inhibitors, localizes to the mitochondria; expression of the gene is under general amino acid control |
| *ILV3* | *YJR016C* | *AFUA_2G14210* | Dihydroxyacid dehydratase, catalyzes third step in the common pathway leading to biosynthesis of branched-chain amino acids |
| *ILV5* | *YLR355C* | *AFUA_3G14490* | Acetohydroxyacid reductoisomerase, mitochondrial protein involved in branched-chain amino acid biosynthesis, also required for maintenance of wild-type mitochondrial DNA and found in mitochondrial nucleoids |
| *ILV6* | *YCL009C* | *AFUA_4G07210* | Mitochondrial acetolactate synthase small subunit, putative |
| *IMG1* | *YCR046C* | *AFUA_5G09490* | Mitochondrial ribosomal protein |
| *IMG2* | *YCR071C* | *AFUA_3G11370* | Mitochondrial large ribosomal subunit L49 |
| *IMH1* | *YLR309C* | *AFUA_4G02990* | Vesicle-mediated transport protein (Imh1) |
| *IML1* | *YJR138W* | *AFUA_2G05390* | Dishevelled, Egl-10, and Pleckstrin domain protein |
| *IML2* | *YJL082W YKR018C* | *AFUA_1G10360* | Putative protein of unknown function; green fluorescent protein (GFP)-fusion protein localizes to the cytoplasm and nucleus |
| *IMP1* | *YMR150C* | *AFUA_5G12820* | Catalytic subunit of the mitochondrial inner membrane peptidase complex, required for maturation of mitochondrial proteins of the intermembrane space; complex contains Imp1p and Imp2p (both catalytic subunits), and Som1p |
| *IMP2* | *YMR035W* | *AFUA_3G13840* | Catalytic subunit of the mitochondrial inner membrane peptidase complex, required for maturation of mitochondrial proteins of the intermembrane space; complex contains Imp1p and Imp2p (both catalytic subunits), and Som1p |
| *IMP4* | *YNL075W* | *AFUA_1G13070* | Component of the SSU processome, which is required for pre-18S rRNA processing; interacts with Mpp10p; member of a superfamily of proteins that contain a sigma(70)-like motif and associate with RNAs |
| *INO1* | *YJL153C* | *AFUA_2G01010* | Myo-inositol-phosphate synthase |
| *INO4* | *YOL108C* | *AFUA_2G12310* | HLH transcription factor |
| *IOC4* | *YMR044W* | *AFUA_8G04570* | PWWP domain protein |
| *IPI1* | *YHR085W* | *AFUA_6G01990* | Essential component of the Rix1 complex that is required for processing of ITS2 sequences from 35S pre-rRNA; Rix1 complex associates with Mdn1p in pre-60S ribosomal particles |
| *IPI3* | *YNL182C* | *AFUA_3G06580* | Essential component of the Rix1 complex that is required for processing of ITS2 sequences from 35S pre-rRNA; highly conserved and contains WD40 motifs; Rix1 complex associates with Mdn1p in pre-60S ribosomal particles |
| *IPP1* | *YBR011C* | *AFUA_3G08380* | Cytoplasmic inorganic pyrophosphatase (PPase), homodimer that catalyzes the rapid exchange of oxygens from Pi with water, highly expressed and essential for viability, active-site residues show identity to those from E. coli PPase |
| *IQG1* | *YPL242C* | *AFUA_2G03640* | Essential protein required for determination of budding pattern, promotes localization of axial markers Bud4p and Cdc12p and functionally interacts with Sec3p, localizes to the contractile ring during anaphase, member of the IQGAP family |
| *IRC20* | *YLR247C* | *AFUA_6G06550* | SNF2 family helicase/ATPase |
| *IRC3* | *YDR332W* | *AFUA_7G05530* | DEAD/DEAH box helicase |
| *IRE1* | *YHR079C* | *AFUA_1G01720* | Protein kinase and ribonuclease Ire1 |
| *IRR1* | *YIL026C* | *AFUA_2G16080* | Nuclear cohesin complex subunit (Psc3) |
| *ISA1* | *YLL027W* | *AFUA_4G10690* | Iron-sulfur cluster assembly accessory protein Isa1, putative |
| *ISA2* | *YPR067W* | *AFUA_2G10370* | Iron-sulfur cluster assembly accessory protein Isa2, putative |
| *ISD11* | *YER048W-A* | *AFUA_3G06492* | Iron-sulfur cluster biosynthesis protein Isd11, putative |
| *ISM1* | *YPL040C* | *AFUA_7G01420* | Isoleucyl-tRNA synthetase |
| *ISN1* | *YOR155C* | *AFUA_4G00970* | Inosine 5'-monophosphate (IMP)-specific 5'-nucleotidase, catalyzes the breakdown of IMP to inosine, does not show similarity to known 5'-nucleotidases from other organisms |
| *ISR1* | *YPR106W* | *AFUA_3G08710* | Protein kinase domain-containing protein |
| *IST1* | *YNL265C* | *AFUA_1G13960* | DUF292 domian protein |
| *IST2* | *YBR086C* | *AFUA_4G02970 AFUA_4G03330* | Plasma membrane stress response protein (Ist2), putative |
| *ISY1* | *YJR050W* | *AFUA_5G08690* | Pre-mRNA splicing factor |
| *ITC1* | *YGL133W YPL216W* | *AFUA_2G16780* | Component of the ATP-dependent Isw2p-Itc1p chromatin remodeling complex, required for repression of a-specific genes, repression of early meiotic genes during mitotic growth, and repression of INO1 |
| *ITR1* | *YOL103W YDR497C* | *AFUA_2G07910* | Myo-inositol transporter |
| *ITT1* | *YML068W* | *AFUA_6G04230* | RING finger protein |
| *IZH1* | *YOL002C YDR492W* | *AFUA_3G10570* | Haemolysin-III channel protein Izh2 |
| *IZH3* | *YLR023C* | *AFUA_6G07160* | IZH family channel protein (Izh3) |
| *JAC1* | *YGL018C* | *AFUA_8G04610* | DnaJ domain protein |
| *JEM1* | *YJL073W* | *AFUA_1G05900 AFUA_3G05400* | DnaJ-like chaperone required for nuclear membrane fusion during mating, localizes to the ER membrane; exhibits genetic interactions with KAR2 |
| *JHD1* | *YER051W* | *AFUA_2G05970* | PHD finger and JmjC domain protein |
| *JID1* | *YPR061C* | *AFUA_3G11530* | Hsp40 co-chaperone Jid1 |
| *JIP4* | *YGR237C YOR019W YDR475C* | *AFUA_2G09500 AFUA_2G13230* | Protein of unknown function that may interact with ribosomes, based on co-purification experiments |
| *JIP5* | *YPR169W* | *AFUA_6G07080* | Essential protein required for biogenesis of the large ribosomal subunit; interacts with proteins involved in RNA processing, ribosome biogenesis, ubiquitination and demethylation; similar to WDR55, a human WD repeat protein |
| *JJJ2* | *YJL162C* | *AFUA_1G06020* | DnaJ domain protein |
| *JLP2* | *YMR132C* | *AFUA_1G08820* | DUF814 domain protein |
| *KAE1* | *YKR038C* | *AFUA_6G04510* | Highly conserved putative glycoprotease proposed to be involved in transcription as a component of the EKC protein complex with Bud32p, Cgi121p, Pcc1p, and Gon7p; also identified as a component of the KEOPS protein complex |
| *KAP104* | *YBR017C* | *AFUA_1G15900* | Importin beta-2 subunit |
| *KAP114* | *YGL241W* | *AFUA_2G15870* | Importin beta-5 subunit |
| *KAP120* | *YPL125W* | *AFUA_1G03590* | Importin 11 |
| *KAP122* | *YGL016W* | *AFUA_5G08060* | Importin 13 |
| *KAP123* | *YER110C* | *AFUA_6G09990* | Importin beta-4 subunit |
| *KAR2* | *YJL034W* | *AFUA_2G04620* | Hsp70 chaperone BiP/Kar2 |
| *KAR3* | *YPR141C* | *AFUA_2G14280* | Kinesin family protein (KlpA) |
| *KEL3* | *YPL263C* | *AFUA_2G04970* | Cytoplasmic protein of unknown function |
| *KEM1* | *YGL173C* | *AFUA_5G03120* | Evolutionarily-conserved 5'-3' exonuclease component of cytoplasmic processing bodies involved in mRNA decay; plays a role in microtubule-mediated processes, filamentous growth, ribosomal RNA maturation, and telomere maintenance |
| *KEX1* | *YGL203C* | *AFUA_1G08940* | Pheromone processing carboxypeptidase KexA |
| *KEX2* | *YNL238W* | *AFUA_4G12970* | Pheromone processing endoprotease KexB |
| *KGD1* | *YIL125W* | *AFUA_4G11650* | Component of the mitochondrial alpha-ketoglutarate dehydrogenase complex, which catalyzes a key step in the tricarboxylic acid cycle, the oxidative decarboxylation of alpha-ketoglutarate to form succinyl-CoA |
| *KGD2* | *YDR148C* | *AFUA_3G05370* | Dihydrolipoyl transsuccinylase, component of the mitochondrial alpha-ketoglutarate dehydrogenase complex, which catalyzes the oxidative decarboxylation of alpha-ketoglutarate to succinyl-CoA in the TCA cycle; phosphorylated |
| *KIN1* | *YLR096W YDR122W* | *AFUA_1G11080* | Serine/threonine protein kinase Kin1 |
| *KIN28* | *YDL108W* | *AFUA_7G03720* | Serine/threonine protein kinase (Kin28) |
| *KIN3* | *YAR018C* | *AFUA_6G02670* | G2-specific protein kinase NimA |
| *KIN4* | *YOR233W YPL141C* | *AFUA_1G14810* | Putative protein kinase; similar to Kin4p; green fluorescent protein (GFP)-fusion protein localizes to the cytoplasm; YPL141C is not an essential gene |
| *KIP1* | *YBL063W* | *AFUA_7G01400* | Kinesin-related motor protein required for mitotic spindle assembly, chromosome segregation, and 2 micron plasmid partitioning; functionally redundant with Cin8p for chromosomal but not plasmid functions |
| *KIP2* | *YPL155C* | *AFUA_7G03710* | Kinesin-related motor protein involved in mitotic spindle positioning, stabilizes microtubules by targeting Bik1p to the plus end; Kip2p levels are controlled during the cell cycle |
| *KIP3* | *YGL216W* | *AFUA_2G03150* | Kinesin-related motor protein involved in mitotic spindle positioning |
| *KNS1* | *YLL019C* | *AFUA_1G16780* | Protein kinase (Lkh1) |
| *KOG1* | *YHR186C* | *AFUA_2G02510* | TORC1 growth control complex subunit Kog1 |
| *KRE5* | *YOR336W* | *AFUA_2G02360* | UDP-glucose:glycoprotein glucosyltransferase |
| *KRE6* | *YGR143W YPR159W* | *AFUA_2G11870* | Protein involved in sphingolipid biosynthesis; type II membrane protein with similarity to Kre6p |
| *KRI1* | *YNL308C* | *AFUA_1G05410* | Essential nucleolar protein required for 40S ribosome biogenesis; physically and functionally interacts with Krr1p |
| *KRR1* | *YCL059C* | *AFUA_2G11380* | Essential nucleolar protein required for the synthesis of 18S rRNA and for the assembly of 40S ribosomal subunit |
| *KRS1* | *YDR037W* | *AFUA_6G07640* | Lysyl-tRNA synthetase |
| *KSH1* | *YNL024C-A* | *AFUA_1G09360* | Essential protein suggested to function early in the secretory pathway; inviability is suppressed by overexpression of Golgi protein Tvp23p; ortholog of human Kish |
| *KSP1* | *YHR082C* | *AFUA_5G06470* | Serine/threonine protein kinase |
| *KTI11* | *YBL071W-A* | *AFUA_4G10520* | CSL family zinc fnger-containing protein |
| *LAA1* | *YJL207C* | *AFUA_6G04080* | AP-1 accessory protein; colocalizes with clathrin to the late-Golgi apparatus; involved in TGN-endosome transport; physically interacts with AP-1; similar to the mammalian p200; may interact with ribosomes; YJL207C is a non-essential gene |
| *LAC1* | *YHL003C YKL008C* | *AFUA_6G10460* | Ceramide synthase component, involved in synthesis of ceramide from C26(acyl)-coenzyme A and dihydrosphingosine or phytosphingosine, functionally equivalent to Lac1p |
| *LAP4* | *YKL103C* | *AFUA_5G03990* | Vacuolar aspartyl aminopeptidase Lap4 |
| *LAS1* | *YKR063C* | *AFUA_5G12850* | Essential nuclear protein possibly involved in bud formation and morphogenesis; mutants require the SSD1-v allele for viability |
| *LAS17* | *YOR181W* | *AFUA_6G02540* | Actin assembly factor, activates the Arp2/3 protein complex that nucleates branched actin filaments; localizes with the Arp2/3 complex to actin patches; homolog of the human Wiskott-Aldrich syndrome protein (WASP) |
| *LAS21* | *YJL062W* | *AFUA_6G05260* | Integral plasma membrane protein involved in the synthesis of the glycosylphosphatidylinositol core structure; mutations affect cell wall integrity |
| *LAT1* | *YNL071W* | *AFUA_7G05720* | Dihydrolipoamide acetyltransferase component of pyruvate dehydrogenase complex, which catalyzes the oxidative decarboxylation of pyruvate to acetyl-CoA |
| *LCP5* | *YER127W* | *AFUA_6G14260* | Essential protein involved in maturation of 18S rRNA; depletion leads to inhibited pre-rRNA processing and reduced polysome levels; localizes primarily to the nucleolus |
| *LDB17* | *YDL146W* | *AFUA_6G12370* | Ldb17p |
| *LDB19* | *YOR322C* | *AFUA_5G12530* | Arrestin (or S-antigen), N-terminal domain protein |
| *LEE1* | *YPL054W* | *AFUA_3G05570* | Spindle poison sensitivity protein Scp3 |
| *LEU1* | *YGL009C* | *AFUA_2G11260* | Isopropylmalate isomerase, catalyzes the second step in the leucine biosynthesis pathway |
| *LEU2* | *YCL018W* | *AFUA_1G15780* | Beta-isopropylmalate dehydrogenase (IMDH), catalyzes the third step in the leucine biosynthesis pathway |
| *LEU3* | *YLR451W* | *AFUA_2G03460 AFUA_3G06290 AFUA_2G15260* | C6 transcription factor (Leu3) |
| *LHS1* | *YKL073W* | *AFUA_1G15050* | Hsp70 family chaperone Lhs1/Orp150 |
| *LIA1* | *YJR070C* | *AFUA_2G05490* | Deoxyhypusine hydroxylase, a HEAT-repeat containing metalloenzyme that catalyzes hypusine formation; binds to and is required for the modification of Hyp2p (eIF5A); complements S. pombe mmd1 mutants defective in mitochondrial positioning |
| *LIP2* | *YLR239C* | *AFUA_3G06680* | Lipoyltransferase |
| *LIP5* | *YOR196C* | *AFUA_3G06560* | Lipoic acid synthetase precursor |
| *LOS1* | *YKL205W* | *AFUA_5G09850* | TRNA exportin |
| *LOT6* | *YLR011W* | *AFUA_7G06600* | FMN-dependent NAD(P)H:quinone reductase, may be involved in quinone detoxification; expression elevated at low temperature; sequesters the Cin5p transcription factor in the cytoplasm in complex with the proteasome under reducing conditions |
| *LPD1* | *YFL018C* | *AFUA_2G02100* | Dihydrolipoamide dehydrogenase, the lipoamide dehydrogenase component of the pyruvate dehydrogenase and 2-oxoglutarate dehydrogenase multi-enzyme complexes |
| *LPX1* | *YOR084W* | *AFUA_3G13560 AFUA_1G01450 AFUA_4G12320* | Toxin biosynthesis protein |
| *LRO1* | *YNR008W* | *AFUA_3G09950* | Acyltransferase that catalyzes diacylglycerol esterification; one of several acyltransferases that contribute to triglyceride synthesis; putative homolog of human lecithin cholesterol acyltransferase |
| *LSB1* | *YGR136W YPR154W* | *AFUA_3G08620* | SH3 domain protein |
| *LSB3* | *YFR024C-A YHR016C* | *AFUA_6G07880* | Actin-binding protein involved in bundling of actin filaments and endocytosis of actin cortical patches; activity stimulated by Las17p; contains SH3 domain similar to Rvs167p |
| *LSB5* | *YCL034W* | *AFUA_6G09080* | VHS domain protein |
| *LSB6* | *YJL100W* | *AFUA_2G13370* | Phosphatidylinositol 4-kinase type II subunit alpha, putative |
| *LSG1* | *YGL099W* | *AFUA_5G06510* | Ribosome biogenesis GTPase Lsg1 |
| *LSM12* | *YHR121W* | *AFUA_6G08070* | Lsm12p |
| *LSM2* | *YBL026W* | *AFUA_2G15210* | Lsm protein; part of heteroheptameric complexes (Lsm2p-7p and either Lsm1p or 8p): cytoplasmic Lsm1p complex involved in mRNA decay; nuclear Lsm8p complex part of U6 snRNP and possibly involved in processing tRNA, snoRNA, and rRNA |
| *LSM3* | *YLR438C-A* | *AFUA_5G12570* | Lsm protein; part of heteroheptameric complexes (Lsm2p-7p and either Lsm1p or 8p): cytoplasmic Lsm1p complex involved in mRNA decay; nuclear Lsm8p complex part of U6 snRNP and possibly involved in processing tRNA, snoRNA, and rRNA |
| *LSM4* | *YER112W* | *AFUA_2G12020* | Lsm protein; part of heteroheptameric complexes (Lsm2p-7p and either Lsm1p or 8p): cytoplasmic Lsm1p complex involved in mRNA decay; nuclear Lsm8p complex part of U6 snRNP and possibly involved in processing tRNA, snoRNA, and rRNA |
| *LSM5* | *YER146W* | *AFUA_7G04280* | Lsm protein; part of heteroheptameric complexes (Lsm2p-7p and either Lsm1p or 8p): cytoplasmic Lsm1p complex involved in mRNA decay; nuclear Lsm8p complex part of U6 snRNP and possibly involved in processing tRNA, snoRNA, and rRNA |
| *LSM6* | *YDR378C* | *AFUA_6G06325* | Lsm protein; part of heteroheptameric complexes (Lsm2p-7p and either Lsm1p or 8p): cytoplasmic Lsm1p complex involved in mRNA decay; nuclear Lsm8p complex part of U6 snRNP and possibly involved in processing tRNA, snoRNA, and rRNA |
| *LSP1* | *YGR086C YPL004C* | *AFUA_6G07520* | Cell wall integrity signaling protein Lsp1/Pil1, putative |
| *LTP1* | *YPR073C* | *AFUA_2G01880* | Low molecular weight phosphotyrosine protein phosphatase |
| *LTV1* | *YKL143W* | *AFUA_5G06520* | Component of the GSE complex, which is required for proper sorting of amino acid permease Gap1p; required for ribosomal small subunit export from nucleus; required for growth at low temperature |
| *LYS1* | *YIR034C* | *AFUA_3G11710* | Saccharopine dehydrogenase Lys1 |
| *LYS14* | *YDR034C YDR207C* | *AFUA_4G09710 AFUA_6G07010 AFUA_3G02480 AFUA_3G15290* | C6 transcription factor |
| *LYS2* | *YBR115C* | *AFUA_4G11240* | Alpha aminoadipate reductase, catalyzes the reduction of alpha-aminoadipate to alpha-aminoadipate 6-semialdehyde, which is the fifth step in biosynthesis of lysine; activation requires posttranslational phosphopantetheinylation by Lys5p |
| *LYS20* | *YDL182W YDL131W* | *AFUA_4G10460* | Homocitrate synthase isozyme, catalyzes the condensation of acetyl-CoA and alpha-ketoglutarate to form homocitrate, which is the first step in the lysine biosynthesis pathway; highly similar to the other isozyme, Lys20p |
| *LYS4* | *YDR234W* | *AFUA_5G08890* | Homoaconitase, catalyzes the conversion of homocitrate to homoisocitrate, which is a step in the lysine biosynthesis pathway |
| *LYS9* | *YNR050C* | *AFUA_4G11340* | Saccharopine dehydrogenase Lys9 |
| *MAD1* | *YGL086W* | *AFUA_1G13580* | Coiled-coil protein involved in the spindle-assembly checkpoint; phosphorylated by Mps1p upon checkpoint activation which leads to inhibition of the activity of the anaphase promoting complex; forms a complex with Mad2p |
| *MAE1* | *YKL029C* | *AFUA_7G02420* | Malate dehydrogenase |
| *MAG1* | *YER142C* | *AFUA_4G06800* | 3-methyl-adenine DNA glycosylase involved in protecting DNA against alkylating agents; initiates base excision repair by removing damaged bases to create abasic sites that are subsequently repaired |
| *MAG2* | *YLR427W* | *AFUA_1G07150* | Cytoplasmic protein of unknown function; induced in response to mycotoxin patulin; ubiquitinated protein similar to the human ring finger motif protein RNF10; predicted to be involved in repair of alkylated DNA due to interaction with MAG1 |
| *MAK16* | *YAL025C* | *AFUA_6G04260* | Essential nuclear protein, constituent of 66S pre-ribosomal particles; required for maturation of 25S and 5.8S rRNAs; required for maintenance of M1 satellite double-stranded RNA of the L-A virus |
| *MAK21* | *YDR060W* | *AFUA_1G10760* | Constituent of 66S pre-ribosomal particles, required for large ribosomal subunit biogenesis; involved in nuclear export of pre-ribosomes; required for maintenance of dsRNA virus; homolog of human CAATT-binding protein |
| *MAK3* | *YPR051W* | *AFUA_3G09940* | Catalytic subunit of N-terminal acetyltransferase of the NatC type; required for replication of dsRNA virus |
| *MAK5* | *YBR142W* | *AFUA_6G08900* | Essential nucleolar protein, putative DEAD-box RNA helicase required for maintenance of M1 dsRNA virus; involved in biogenesis of large ribosomal subunits |
| *MAM3* | *YOL060C* | *AFUA_2G04430* | DUF21 and CBS domain protein (Mam3) |
| *MAM33* | *YIL070C* | *AFUA_3G09030* | Acidic protein of the mitochondrial matrix involved in oxidative phosphorylation; related to the human complement receptor gC1q-R |
| *MAP1* | *YLR244C* | *AFUA_6G07330 AFUA_8G00460* | Methionine aminopeptidase, type I |
| *MAP2* | *YBL091C* | *AFUA_2G01750 AFUA_4G06930 AFUA_8G00410* | Methionine aminopeptidase, type II |
| *MAS1* | *YLR163C* | *AFUA_1G14200* | Mitochondrial processing peptidase beta subunit, putative |
| *MAS2* | *YHR024C* | *AFUA_1G11870* | Mitochondrial processing peptidase alpha subunit, putative |
| *MBA1* | *YBR185C* | *AFUA_3G06270* | Mba1p |
| *MBF1* | *YOR298C-A* | *AFUA_3G08630* | Coactivator bridging factor 1 (Mbf1) |
| *MBP1* | *YDL056W* | *AFUA_3G13920* | APSES transcription factor |
| *MCA1* | *YOR197W* | *AFUA_1G06700 AFUA_3G14140* | Metacaspase CasA |
| *MCD1* | *YDL003W* | *AFUA_2G05850* | Essential subunit of the cohesin complex required for sister chromatid cohesion in mitosis and meiosis; apoptosis induces cleavage and translocation of a C-terminal fragment to mitochondria; expression peaks in S phase |
| *MCH1* | *YDL054C* | *AFUA_5G13230* | MFS monocarboxylic acid transporter |
| *MCM1* | *YMR043W* | *AFUA_6G02110* | MADS box transcription factor Mcm1 |
| *MCM10* | *YIL150C* | *AFUA_3G10280* | Essential chromatin-associated protein involved in the initiation of DNA replication; required for the association of the MCM2-7 complex with replication origins |
| *MCM2* | *YBL023C* | *AFUA_3G14010* | DNA replication licensing factor Mcm2 |
| *MCM3* | *YEL032W* | *AFUA_1G03920* | DNA replication licensing factor Mcm3 |
| *MCM4* | *YPR019W* | *AFUA_2G09060* | Essential helicase component of heterohexameric MCM2-7 complexes which bind pre-replication complexes on DNA and melt the DNA prior to replication; accumulates in the nucleus in G1; homolog of S. pombe Cdc21p |
| *MCM5* | *YLR274W* | *AFUA_5G02520* | Component of the hexameric MCM complex, which is important for priming origins of DNA replication in G1 and becomes an active ATP-dependent helicase that promotes DNA melting and elongation when activated by Cdc7p-Dbf4p in S-phase |
| *MCM6* | *YGL201C* | *AFUA_5G10890* | DNA replication licensing factor Mcm6 |
| *MCM7* | *YBR202W* | *AFUA_2G10140* | Component of the hexameric MCM complex, which is important for priming origins of DNA replication in G1 and becomes an active ATP-dependent helicase that promotes DNA melting and elongation when activated by Cdc7p-Dbf4p in S-phase |
| *MCR1* | *YKL150W* | *AFUA_1G04540* | NADH-cytochrome b5 reductase |
| *MDE1* | *YJR024C* | *AFUA_4G12840* | 5'-methylthioribulose-1-phosphate dehydratase; acts in the methionine salvage pathway; potential Smt3p sumoylation substrate; expression downregulated by caspofungin and deletion mutant is caspofungin resistant |
| *MDH1* | *YKL085W* | *AFUA_7G05740* | Malate dehydrogenase, NAD-dependent |
| *MDJ1* | *YFL016C* | *AFUA_2G11750* | Co-chaperone that stimulates the ATPase activity of the HSP70 protein Ssc1p; involved in protein folding/refolding in the mitochodrial matrix; required for proteolysis of misfolded proteins; member of the HSP40 family of chaperones |
| *MDM1* | *YML104C* | *AFUA_1G09040* | Intermediate filament protein, required for nuclear and mitochondrial transmission to daughter buds; contains a Phox homology domain and specifically binds phosphatidylinositol 3-phosphate (PtdIns-3-P) |
| *MDM10* | *YAL010C* | *AFUA_5G13460* | Mitochondrial protein |
| *MDM12* | *YOL009C* | *AFUA_1G14790* | Mitochondrial inheritance component mdm12 |
| *MDM20* | *YOL076W* | *AFUA_4G07460* | Cytoskeleton organisation protein (Dec1) |
| *MDM31* | *YHR194W* | *AFUA_6G06420* | Mitochondrion biogenesis protein (Mdm31) |
| *MDM34* | *YGL219C* | *AFUA_4G09960* | Mdm34p |
| *MDN1* | *YLR106C* | *AFUA_2G12150* | Huge dynein-related AAA-type ATPase (midasin), forms extended pre-60S particle with the Rix1 complex (Rix1p-Ipi1p-Ipi3p), may mediate ATP-dependent remodeling of 60S subunits and subsequent export from nucleoplasm to cytoplasm |
| *MDR1* | *YGR100W* | *AFUA_5G07440* | Cytoplasmic GTPase-activating protein for Ypt/Rab transport GTPases Ypt6p, Ypt31p and Sec4p; involved in recycling of internalized proteins and regulation of Golgi secretory function |
| *MED6* | *YHR058C* | *AFUA_6G04810* | RNA polymerase II transcription mediator complex subunit (Med6) |
| *MED7* | *YOL135C* | *AFUA_6G10020* | RNA polymerase II mediator complex protein (Med7), putative |
| *MED8* | *YBR193C* | *AFUA_4G08360* | RNA polymerase II mediator complex component Med8, putative |
| *MEF1* | *YLR069C* | *AFUA_4G08110* | Translation elongation factor G1 |
| *MEF2* | *YJL102W* | *AFUA_5G07140* | Translation elongation factor G2 |
| *MEK1* | *YOR351C* | *AFUA_5G07950* | Meiosis-specific serine/threonine protein kinase, functions in meiotic checkpoint, promotes recombination between homologous chromosomes by suppressing double strand break repair between sister chromatids |
| *MEP2* | *YNL142W* | *AFUA_1G10930* | Ammonium permease involved in regulation of pseudohyphal growth; belongs to a ubiquitous family of cytoplasmic membrane proteins that transport only ammonium (NH4+); expression is under the nitrogen catabolite repression regulation |
| *MES1* | *YGR264C* | *AFUA_1G09010* | Methionyl-tRNA synthetase |
| *MET13* | *YGL125W* | *AFUA_2G11300* | Major isozyme of methylenetetrahydrofolate reductase, catalyzes the reduction of 5,10-methylenetetrahydrofolate to 5-methyltetrahydrofolate in the methionine biosynthesis pathway |
| *MET17* | *YLR303W* | *AFUA_5G04250* | Homocysteine synthase CysD |
| *MET18* | *YIL128W* | *AFUA_8G05370* | DNA repair and TFIIH regulator, required for both nucleotide excision repair and RNA polymerase II transcription; involved in telomere maintenance |
| *MET2* | *YNL277W* | *AFUA_5G07210* | L-homoserine-O-acetyltransferase, catalyzes the conversion of homoserine to O-acetyl homoserine which is the first step of the methionine biosynthetic pathway |
| *MET22* | *YOL064C* | *AFUA_4G04200 AFUA_6G09070* | 3'(2'),5'-bisphosphate nucleotidase |
| *MET30* | *YIL046W* | *AFUA_2G14110* | F-box protein containing five copies of the WD40 motif, controls cell cycle function, sulfur metabolism, and methionine biosynthesis as part of the ubiquitin ligase complex; interacts with and regulates Met4p, localizes within the nucleus |
| *MET4* | *YNL103W* | *AFUA_4G06530 AFUA_6G07530* | Leucine-zipper transcriptional activator, responsible for the regulation of the sulfur amino acid pathway, requires different combinations of the auxiliary factors Cbf1p, Met28p, Met31p and Met32p |
| *MET6* | *YER091C* | *AFUA_4G07360* | Cobalamin-independent methionine synthase, involved in methionine biosynthesis and regeneration; requires a minimum of two glutamates on the methyltetrahydrofolate substrate, similar to bacterial metE homologs |
| *MEX67* | *YPL169C* | *AFUA_1G05110* | MRNA export factor mex67 |
| *MGA2* | *YIR033W YKL020C* | *AFUA_1G12550* | ER membrane protein involved in regulation of OLE1 transcription, acts with homolog Spt23p; inactive ER form dimerizes and one subunit is then activated by ubiquitin/proteasome-dependent processing followed by nuclear targeting |
| *MGE1* | *YOR232W* | *AFUA_2G13040* | Mitochondrial co-chaperone GrpE |
| *MGM1* | *YOR211C* | *AFUA_1G11970* | Mitochondrial dynamin GTPase (Msp1) |
| *MGM101* | *YJR144W* | *AFUA_2G09560* | Mitochondrial genome maintenance protein Mgm101, putative |
| *MGR2* | *YPL098C* | *AFUA_2G02430* | Mitochondrial genome maintenance protein Mgr2 |
| *MGR3* | *YMR115W YKL133C* | *AFUA_4G13160* | Putative protein of unknown function; has similarity to Mgr3p, but unlike MGR3, is not required for growth of cells lacking the mitochondrial genome (null mutation does not confer a petite-negative phenotype) |
| *MGS1* | *YNL218W* | *AFUA_4G11180* | AAA family ATPase |
| *MGT1* | *YDL200C* | *AFUA_2G02090* | DNA repair methyltransferase involved in protection against DNA alkylation damage |
| *MHT1* | *YMR321C YLL062C YPL273W* | *AFUA_5G01500* | S-adenosylmethionine-homocysteine methyltransferase, functions along with Mht1p in the conversion of S-adenosylmethionine to methionine to control the methionine/AdoMet ratio |
| *MIA40* | *YKL195W* | *AFUA_7G05420* | Essential protein of the mitochondrial intermembrane space (IMS); promotes retention of newly imported proteins; may do so by stabilizing client protein folding as part of a disulfide relay system or transferring metal to client proteins |
| *MIC17* | *YMR002W* | *AFUA_3G06370* | CHCH domain protein |
| *MID1* | *YNL291C* | *AFUA_5G05840* | Calcium channel subunit Mid1 |
| *MIF2* | *YKL089W* | *AFUA_1G07590* | Kinetochore protein with homology to human CENP-C, required for structural integrity of the spindle during anaphase spindle elongation, interacts with histones H2A, H2B, and H4, phosphorylated by Ipl1p |
| *MIG1* | *YGL035C* | *AFUA_2G11780* | C2H2 transcription factor (Crea) |
| *MIM1* | *YOL026C* | *AFUA_5G03630* | Mim1p |
| *MIP1* | *YOR330C* | *AFUA_5G12640* | DNA polymerase gamma |
| *MIR1* | *YJR077C* | *AFUA_1G02730 AFUA_1G15140* | Mitochondrial phosphate carrier protein (Mir1), putative |
| *MKK1* | *YOR231W YPL140C* | *AFUA_1G05800* | Mitogen-activated kinase kinase involved in protein kinase C signaling pathway that controls cell integrity; upon activation by Bck1p phosphorylates downstream target, Slt2p; functionally redundant with Mkk2p |
| *MLC2* | *YPR188C* | *AFUA_1G02930* | Calmodulin |
| *MLH1* | *YMR167W* | *AFUA_5G11700* | DNA mismatch repair protein Mlh1 |
| *MLP1* | *YIL149C YKR095W* | *AFUA_6G13120* | Filament-forming protein (Tpr/p270) |
| *MMM1* | *YLL006W* | *AFUA_6G04420* | ER integral membrane protein, component of the ERMES complex that links the ER to mitochondria and may promote inter-organellar calcium and phospholipid exchange as well as coordinating mitochondrial DNA replication and growth |
| *MMT1* | *YMR177W YPL224C* | *AFUA_5G09830* | Cation efflux family protein |
| *MND1* | *YGL183C* | *AFUA_4G09640* | GAJ protein |
| *MNI1* | *YIL110W* | *AFUA_8G04330* | Mni1p |
| *MNN10* | *YDR245W* | *AFUA_2G14910* | Alpha-1,6-mannosyltransferase subunit |
| *MNN11* | *YJL183W* | *AFUA_4G10750* | Alpha-1,6-mannosyltransferase subunit |
| *MNN9* | *YPL050C* | *AFUA_2G01450* | Alpha-1,6 mannosyltransferase subunit (Mnn9) |
| *MNP1* | *YGL068W* | *AFUA_4G09750* | 50S ribosomal protein L12 |
| *MNR2* | *YKL064W* | *AFUA_2G08070* | CorA family metal ion transporter |
| *MNS1* | *YJR131W* | *AFUA_6G06790* | Alpha-1,2-mannosidase involved in ER quality control; catalyzes the removal of one mannose residue from Man9GlcNAc to produce a single isomer of Man8GlcNAc in N-linked oligosaccharide biosynthesis; integral to ER membrane |
| *MOD5* | *YOR274W* | *AFUA_6G05070* | Delta 2-isopentenyl pyrophosphate:tRNA isopentenyl transferase, required for biosynthesis of the modified base isopentenyladenosine in mitochondrial and cytoplasmic tRNAs; gene is nuclear and encodes two isozymic forms |
| *MOG1* | *YJR074W* | *AFUA_6G12430* | Conserved nuclear protein that interacts with GTP-Gsp1p, which is a Ran homolog of the Ras GTPase family, and stimulates nucleotide release, involved in nuclear protein import, nucleotide release is inhibited by Yrb1p |
| *MOH1* | *YBL049W* | *AFUA_2G02820* | Yippee zinc-binding protein Moh1 |
| *MON1* | *YGL124C* | *AFUA_2G05010* | Mon1p |
| *MON2* | *YNL297C* | *AFUA_4G12070* | Endosomal peripheral membrane protein (Mon2) |
| *MOT1* | *YPL082C* | *AFUA_1G05830* | Essential abundant protein involved in regulation of transcription, removes Spt15p from DNA via its C-terminal ATPase activity, forms a complex with TBP that binds TATA DNA with high affinity but with altered specificity |
| *MOT2* | *YER068W* | *AFUA_6G08820* | CCR4-NOT core complex subunit Not4 |
| *MPD1* | *YOR288C* | *AFUA_1G05320* | Member of the protein disulfide isomerase family; interacts with and inhibits the chaperone activity of Cne1p; MPD1 overexpression in a pdi1 null mutant suppresses defects in Pdi1p functions such as carboxypeptidase Y maturation |
| *MPE1* | *YKL059C* | *AFUA_2G06220* | Essential conserved subunit of CPF (cleavage and polyadenylation factor), plays a role in 3' end formation of mRNA via the specific cleavage and polyadenylation of pre-mRNA, contains a putative RNA-binding zinc knuckle motif |
| *MPH1* | *YIR002C* | *AFUA_1G03050* | Member of the DEAH family of helicases, functions in an error-free DNA damage bypass pathway that involves homologous recombination, binds to flap DNA and stimulates activity of Rad27p and Dna2p; mutations confer a mutator phenotype |
| *MPP10* | *YJR002W* | *AFUA_4G05880* | Component of the SSU processome and 90S preribosome, required for pre-18S rRNA processing, interacts with and controls the stability of Imp3p and Imp4p, essential for viability; similar to human Mpp10p |
| *MRC1* | *YCL061C* | *AFUA_2G01910* | Mrc1p |
| *MRD1* | *YPR112C* | *AFUA_1G04840* | Essential conserved protein that is part of the 90S preribosome; required for production of 18S rRNA and small ribosomal subunit; contains five consensus RNA-binding domains |
| *MRE11* | *YMR224C* | *AFUA_6G11410* | Meiotic recombination protein Mre11 |
| *MRF1* | *YGL143C* | *AFUA_3G05410* | Mitochondrial translation release factor, involved in stop codon recognition and hydrolysis of the peptidyl-tRNA bond during mitochondrial translation; lack of MRF1 causes mitochondrial genome instability |
| *MRH4* | *YGL064C* | *AFUA_2G08480* | Mitochondrial ATP-dependent RNA helicase of the DEAD-box family, plays an essential role in mitochondrial function |
| *MRI1* | *YPR118W* | *AFUA_4G05830* | 5'-methylthioribose-1-phosphate isomerase; catalyzes the isomerization of 5-methylthioribose-1-phosphate to 5-methylthioribulose-1-phosphate in the methionine salvage pathway |
| *MRK1* | *YMR139W YDL079C* | *AFUA_6G05120* | Protein kinase required for signal transduction during entry into meiosis; promotes the formation of the Ime1p-Ume6p complex by phosphorylating Ime1p and Ume6p; shares similarity with mammalian glycogen synthase kinase 3-beta |
| *MRL1* | *YPR079W* | *AFUA_1G05730* | Membrane protein with similarity to mammalian mannose-6-phosphate receptors, possibly functions as a sorting receptor in the delivery of vacuolar hydrolases |
| *MRM1* | *YOR201C* | *AFUA_4G13450* | RNA methyltransferase, TrmH family family |
| *MRN1* | *YPL184C* | *AFUA_2G05670* | Differentiation regulator (Nrd1) |
| *MRP10* | *YDL045W-A* | *AFUA_1G04400* | Mitochondrial ribosomal protein of the small subunit; contains twin cysteine-x9-cysteine motifs |
| *MRP17* | *YKL003C* | *AFUA_1G12065* | Mitochondrial ribosomal protein of the small subunit; MRP17 exhibits genetic interactions with PET122, encoding a COX3-specific translational activator |
| *MRP2* | *YPR166C* | *AFUA_2G02270* | Mitochondrial ribosomal protein of the small subunit |
| *MRP20* | *YDR405W* | *AFUA_5G11740* | Mitochondrial ribosomal protein L23, putative |
| *MRP4* | *YHL004W* | *AFUA_1G06570* | Mitochondrial ribosomal protein of the small subunit |
| *MRP49* | *YKL167C* | *AFUA_6G12620* | Mitochondrial ribosomal protein of the large subunit, not essential for mitochondrial translation |
| *MRP51* | *YPL118W* | *AFUA_2G10290* | Mitochondrial ribosomal protein of the small subunit; MRP51 exhibits genetic interactions with mutations in the COX2 and COX3 mRNA 5'-untranslated leader sequences |
| *MRP7* | *YNL005C* | *AFUA_5G04030* | 50S ribosomal protein L27 |
| *MRPL10* | *YNL284C* | *AFUA_1G11940* | 50S ribosomal subunit protein L15 |
| *MRPL11* | *YDL202W* | *AFUA_4G11990* | Mrpl11p |
| *MRPL15* | *YLR312W-A* | *AFUA_5G09670* | RNase III domain protein |
| *MRPL16* | *YBL038W* | *AFUA_2G12230* | Mitochondrial large ribosomal subunit protein L16, putative |
| *MRPL17* | *YNL252C* | *AFUA_2G16970* | 50S ribosomal subunit L30 |
| *MRPL19* | *YNL185C* | *AFUA_5G11830* | Mitochondrial ribosomal protein L11 |
| *MRPL20* | *YKR085C* | *AFUA_3G05460* | 60S ribosomal protein L20 |
| *MRPL22* | *YNL177C* | *AFUA_2G01830* | Mitochondrial ribosomal protein of the large subunit |
| *MRPL23* | *YOR150W* | *AFUA_2G10510* | 50S ribosomal protein L13 |
| *MRPL24* | *YMR193W* | *AFUA_4G10740* | 50S ribosomal protein L24 |
| *MRPL25* | *YGR076C* | *AFUA_4G09000* | 60S ribosomal protein L25 |
| *MRPL27* | *YBR282W* | *AFUA_1G04230* | 50S ribosomal protein YmL27 |
| *MRPL3* | *YMR024W* | *AFUA_1G12730* | 60S ribosomal protein L3 |
| *MRPL33* | *YMR286W* | *AFUA_4G10480* | 50S ribosomal protein L30 |
| *MRPL35* | *YDR322W* | *AFUA_5G12810* | Mitochondrial large ribosomal subunit YmL35 |
| *MRPL36* | *YBR122C* | *AFUA_4G12810* | 50S ribosomal protein L36 |
| *MRPL37* | *YBR268W* | *AFUA_3G07920* | Mrpl37p |
| *MRPL38* | *YKL170W* | *AFUA_2G08520* | 50S ribosomal protein L14 |
| *MRPL4* | *YLR439W* | *AFUA_2G11140* | 50S ribosomal protein L4 |
| *MRPL40* | *YPL173W* | *AFUA_6G06890* | KOW motif domain protein |
| *MRPL49* | *YJL096W* | *AFUA_3G08080* | Aconitate hydratase |
| *MRPL51* | *YPR100W* | *AFUA_5G13490* | Mitochondrial ribosomal protein of the large subunit |
| *MRPL6* | *YHR147C* | *AFUA_6G09060* | 60S ribosomal protein L6 |
| *MRPL7* | *YDR237W* | *AFUA_5G06430* | 50S ribosomal subunit L7 |
| *MRPL8* | *YJL063C* | *AFUA_1G09410* | 50S ribosomal protein L17 |
| *MRPL9* | *YGR220C* | *AFUA_4G06000* | 50S ribosomal protein L3 |
| *MRPS16* | *YPL013C* | *AFUA_5G08350* | Mitochondrial ribosomal protein of the small subunit |
| *MRPS18* | *YNL306W* | *AFUA_1G09850* | 37S ribosomal protein S11 |
| *MRPS28* | *YDR337W* | *AFUA_3G13310* | Mitochondrial ribosomal protein of the small subunit |
| *MRPS35* | *YGR165W* | *AFUA_5G08380* | Mitochondrial ribosomal protein of the small subunit |
| *MRPS5* | *YBR251W* | *AFUA_5G11540* | Mitochondrial ribosomal protein of the small subunit |
| *MRPS8* | *YMR158W* | *AFUA_6G07360* | Mitochondrial ribosomal protein of the small subunit |
| *MRPS9* | *YBR146W* | *AFUA_6G04560* | Mitochondrial ribosomal protein of the small subunit |
| *MRS2* | *YOR334W* | *AFUA_6G02550* | Mitochondrial inner membrane Mg(2+) channel, required for maintenance of intramitochondrial Mg(2+) concentrations at the correct level to support splicing of group II introns |
| *MRS3* | *YJL133W YKR052C* | *AFUA_6G12550* | Iron transporter that mediates Fe2+ transport across the inner mitochondrial membrane; mitochondrial carrier family member, similar to and functionally redundant with Mrs4p; active under low-iron conditions; may transport other cations |
| *MRS6* | *YOR370C* | *AFUA_6G02130* | Rab geranylgeranyl transferase escort protein |
| *MSB3* | *YOL112W YNL293W* | *AFUA_3G12870* | GTPase-activating protein for Sec4p and several other Rab GTPases, regulates exocytosis via its action on Sec4p, also required for proper actin organization; similar to Msb4p; both Msb3p and Msb4p localize to sites of polarized growth |
| *MSC1* | *YML128C* | *AFUA_3G10480* | Meiotic sister chromatid recombination protein Ish1/Msc1 |
| *MSC2* | *YDR205W* | *AFUA_6G14170* | Member of the cation diffusion facilitator family, localizes to the endoplasmic reticulum and nucleus; mutations affect the cellular distribution of zinc and also confer defects in meiotic recombination between homologous chromatids |
| *MSD1* | *YPL104W* | *AFUA_4G08330* | Aspartyl-tRNA synthetase |
| *MSE1* | *YOL033W* | *AFUA_2G16280* | Glutamyl-tRNA synthetase |
| *MSF1* | *YPR047W* | *AFUA_2G13030* | Phenylalanyl-tRNA synthetase alpha subunit (PodG), putative |
| *MSH1* | *YHR120W* | *AFUA_8G04750* | DNA-binding protein of the mitochondria involved in repair of mitochondrial DNA, has ATPase activity and binds to DNA mismatches; has homology to E. coli MutS; transcription is induced during meiosis |
| *MSH2* | *YOL090W* | *AFUA_3G09850* | DNA mismatch repair protein Msh2 |
| *MSH3* | *YCR092C* | *AFUA_7G04480* | Mismatch repair protein, forms dimers with Msh2p that mediate repair of insertion or deletion mutations and removal of nonhomologous DNA ends, contains a PCNA binding motif required for genome stability |
| *MSH4* | *YFL003C* | *AFUA_1G02000* | DNA mismatch repair protein Msh4 |
| *MSH5* | *YDL154W* | *AFUA_1G11170* | DNA mismatch repair protein Msh5 |
| *MSH6* | *YDR097C* | *AFUA_4G08300* | DNA mismatch repair protein Msh6 |
| *MSL5* | *YLR116W* | *AFUA_3G10840* | Zinc knuckle transcription factor/splicing factor MSL5/ZFM1 |
| *MSM1* | *YGR171C* | *AFUA_4G07820* | Methionyl-tRNA synthetase |
| *MSP1* | *YGR028W* | *AFUA_4G03990* | Mitochondrial protein involved in sorting of proteins in the mitochondria; putative membrane-spanning ATPase |
| *MSS1* | *YMR023C* | *AFUA_6G04950* | Mitochondrial protein, forms a heterodimer complex with Mto1p that performs the 5-carboxymethylaminomethyl modification of the wobble uridine base in mitochondrial tRNAs; similar to human GTPBP3 |
| *MSS116* | *YDR194C* | *AFUA_1G15620* | DEAD-box protein required for efficient splicing of mitochondrial Group I and II introns; non-polar RNA helicase that also facilities strand annealing |
| *MSS4* | *YDR208W* | *AFUA_3G06080* | Multicopy suppressor of stt4 mutation |
| *MSS51* | *YLR203C* | *AFUA_7G02470* | MRNA processing protein (Mss51) |
| *MST1* | *YKL194C* | *AFUA_1G16210* | Mitochondrial threonyl-tRNA synthetase |
| *MSW1* | *YDR268W* | *AFUA_6G05340* | Tryptophanyl-tRNA synthetase |
| *MSY1* | *YPL097W* | *AFUA_4G08320* | Tyrosyl-tRNA synthetase, mitochondrial precursor (tyrrs) |
| *MTC1* | *YJL123C* | *AFUA_1G03680* | Mtc1p |
| *MTC5* | *YDR128W* | *AFUA_6G02410* | WD repeat protein |
| *MTC6* | *YHR151C* | *AFUA_2G12590* | Lectin C-type domain protein |
| *MTD1* | *YKR080W* | *AFUA_8G05330* | Methylenetetrahydrofolate dehydrogenase |
| *MTG1* | *YMR097C* | *AFUA_5G08680* | Mitochondrial GTPase (YlqF) |
| *MTG2* | *YHR168W* | *AFUA_1G15500* | GTP-binding protein Obg |
| *MTO1* | *YGL236C* | *AFUA_6G04440* | Mitochondrial protein, forms a heterodimer complex with Mss1p that performs the 5-carboxymethylaminomethyl modification of the wobble uridine base in mitochondrial tRNAs; required for respiration in paromomycin-resistant 15S rRNA mutants |
| *MTQ2* | *YDR140W* | *AFUA_2G05100* | Mtq2p |
| *MTR10* | *YOR160W* | *AFUA_7G05970* | MRNA transport regulator (Mtr10) |
| *MTR4* | *YJL050W* | *AFUA_4G07160* | ATP-dependent 3'-5' RNA helicase, involved in nuclear RNA processing and degredation both as a component of the TRAMP complex and in TRAMP independent processes; member of the Dead-box family of helicases |
| *MTW1* | *YAL034W-A* | *AFUA_1G16570* | Essential component of the MIND kinetochore complex which joins kinetochore subunits contacting DNA to those contacting microtubules; critical to kinetochore assembly |
| *MUD2* | *YKL074C* | *AFUA_7G05310* | Splicing factor u2af large subunit |
| *MUP1* | *YGR055W* | *AFUA_4G09040* | High affinity methionine permease, integral membrane protein with 13 putative membrane-spanning regions; also involved in cysteine uptake |
| *MUS81* | *YDR386W* | *AFUA_3G12750* | DNA repair protein Mus81 |
| *MVP1* | *YMR004W* | *AFUA_2G17180* | Sorting nexin Mvp1 |
| *MXR1* | *YER042W* | *AFUA_2G03140* | Methionine-S-sulfoxide reductase, involved in the response to oxidative stress; protects iron-sulfur clusters from oxidative inactivation along with MXR2; involved in the regulation of lifespan |
| *MYO1* | *YHR023W* | *AFUA_5G08700* | Myosin type II heavy chain |
| *MYO2* | *YOR326W YAL029C* | *AFUA_5G05550* | Class V myosin (Myo4) |
| *MYO3* | *YMR109W YKL129C* | *AFUA_8G05660* | Myosin I MyoA/Myo5 |
| *MZM1* | *YDR493W* | *AFUA_4G03645* | Mitochondrial complex I protein Fmp36 |
| *NAB2* | *YGL122C* | *AFUA_6G08450* | Nuclear polyadenylated RNA-binding protein Nab2, putative |
| *NAB3* | *YPL190C* | *AFUA_3G09770* | RNA-binding protein (Nab3) |
| *NAF1* | *YNL124W* | *AFUA_1G13880* | SnoRNP assembly factor Naf1 |
| *NAM2* | *YLR382C* | *AFUA_6G04910* | LeuRS; mitochondrial leucyl-tRNA synthetase |
| *NAM7* | *YMR080C* | *AFUA_1G13060* | ATP-dependent RNA helicase of the SFI superfamily involved in nonsense mediated mRNA decay; required for efficient translation termination at nonsense codons and targeting of NMD substrates to P-bodies; involved in telomere maintenance |
| *NAM9* | *YNL137C* | *AFUA_1G06980* | Mitochondrial ribosomal component of the small subunit |
| *NAN1* | *YPL126W* | *AFUA_7G02610* | WD repeat protein |
| *NAP1* | *YKR048C* | *AFUA_5G05540* | Nucleosome assembly protein Nap1 |
| *NAR1* | *YNL240C* | *AFUA_4G11960* | Component of the cytosolic iron-sulfur protein assembly machinery, required for maturation of cytosolic and nuclear FeS proteins and for normal resistance to oxidative stress; homologous to human Narf |
| *NAS2* | *YIL007C* | *AFUA_6G13310* | 26S proteasome non-ATPase regulatory subunit Nas2, putative |
| *NAT1* | *YDL040C* | *AFUA_4G11910* | N-terminal acetyltransferase catalytic subunit (NAT1), putative |
| *NAT2* | *YGR147C* | *AFUA_4G06010* | Peptide alpha-N-acetyltransferase Nat2 |
| *NAT4* | *YMR069W* | *AFUA_4G08210* | N alpha-acetyl-transferase, involved in acetylation of the N-terminal residues of histones H4 and H2A |
| *NBP35* | *YGL091C* | *AFUA_2G15960* | Essential iron-sulfur cluster binding protein localized in the cytoplasm; forms a complex with Cfd1p that is involved in iron-sulfur protein assembly in the cytosol; similar to P-loop NTPases |
| *NCA3* | *YIL123W YNL066W YJL116C YKR042W* | *AFUA_7G05450* | Cell wall protein involved in cell wall; member of the SUN family |
| *NCB2* | *YDR397C* | *AFUA_3G02340* | CBF/NF-Y family transcription factor |
| *NCL1* | *YBL024W* | *AFUA_1G14180* | Methyltransferase (Ncl1) |
| *NCP1* | *YHR042W* | *AFUA_6G10990* | NADP-cytochrome P450 reductase; involved in ergosterol biosynthesis; associated and coordinately regulated with Erg11p |
| *NCS2* | *YNL119W* | *AFUA_5G12400* | Ncs2p |
| *NCS6* | *YGL211W* | *AFUA_1G04710* | PP-loop ATPase superfamily protein |
| *NEM1* | *YHR004C* | *AFUA_1G09460* | NIF domain protein |
| *NFS1* | *YCL017C* | *AFUA_3G14240* | Cysteine desulfurase involved in iron-sulfur cluster biogenesis; required for the post-transcriptional thio-modification of mitochondrial and cytoplasmic tRNAs; essential protein located predominantly in mitochondria |
| *NGG1* | *YDR176W* | *AFUA_1G04590* | Transcriptional regulator Ngg1 |
| *NHP2* | *YDL208W* | *AFUA_1G13570* | Small nuclear ribonucleoprotein complex protein Nhp2, putative |
| *NHP6A* | *YBR089C-A YPR052C* | *AFUA_3G11610* | High-mobility group protein that binds to and remodels nucleosomes; involved in recruiting FACT and other chromatin remodelling complexes to the chromosomes; functionally redundant with Nhp6Ap; homologous to mammalian HMGB1 and HMGB2 |
| *NIC96* | *YFR002W* | *AFUA_4G04720* | Component of the nuclear pore complex, required for nuclear pore formation; forms a subcomplex with Nsp1p, Nup57p, and Nup49p |
| *NIP1* | *YMR309C* | *AFUA_4G03860* | Eukaryotic translation initiation factor 3 subunit EifCc |
| *NIP7* | *YPL211W* | *AFUA_2G17060* | 60S ribosome biogenesis protein Nip7 |
| *NIT1* | *YIL164C* | *AFUA_6G13450* | Nitrilase |
| *NMA1* | *YGR010W YLR328W* | *AFUA_6G08870* | Nicotinic acid mononucleotide adenylyltransferase, involved in pathways of NAD biosynthesis, including the de novo, NAD(+) salvage, and nicotinamide riboside salvage pathways |
| *NMA111* | *YNL123W* | *AFUA_6G13650* | Nuclear serine protease HtrA2/Nma111 |
| *NMD2* | *YHR077C* | *AFUA_7G05430* | Nonsense-mediated mRNA decay factor (Upf2) |
| *NMT1* | *YLR195C* | *AFUA_4G08070* | N-myristoyl transferase, catalyzes the cotranslational, covalent attachment of myristic acid to the N-terminal glycine residue of several proteins involved in cellular growth and signal transduction |
| *NNF1* | *YJR112W* | *AFUA_5G04140* | Essential component of the MIND kinetochore complex which joins kinetochore subunits contacting DNA to those contacting microtubules; required for accurate chromosome segregation |
| *NNF2* | *YGR089W* | *AFUA_4G12790* | Ubiquitination network signaling protein |
| *NNT1* | *YLR285W* | *AFUA_3G14110* | Nicotinamide N-methyltransferase Nnt1 |
| *NOB1* | *YOR056C* | *AFUA_5G04000* | Essential nuclear protein involved in proteasome maturation and synthesis of 40S ribosomal subunits; required for cleavage of the 20S pre-rRNA to generate the mature 18S rRNA |
| *NOC2* | *YOR206W* | *AFUA_1G16330* | Ribosome assembly protein Noc2 |
| *NOC3* | *YLR002C* | *AFUA_2G17050* | Nuclear export protein Noc3 |
| *NOC4* | *YPR144C* | *AFUA_8G05430* | Ribosome biogenesis protein Noc4 |
| *NOG1* | *YPL093W* | *AFUA_2G11510* | Nucleolar GTP-binding protein (Nog1) |
| *NOG2* | *YNR053C* | *AFUA_4G08930* | Nucleolar GTPase |
| *NOP1* | *YDL014W* | *AFUA_1G14220* | Fibrillarin |
| *NOP12* | *YOL041C* | *AFUA_8G04400* | RNA binding protein |
| *NOP14* | *YDL148C* | *AFUA_8G04220* | RRNA maturation protein (Nop14) |
| *NOP15* | *YNL110C* | *AFUA_4G06350* | Constituent of 66S pre-ribosomal particles, involved in 60S ribosomal subunit biogenesis; localizes to both nucleolus and cytoplasm |
| *NOP16* | *YER002W* | *AFUA_2G01480* | Constituent of 66S pre-ribosomal particles, involved in 60S ribosomal subunit biogenesis |
| *NOP2* | *YNL061W* | *AFUA_5G01760* | Nucleolar RNA methyltransferase (Nop2) |
| *NOP4* | *YPL043W* | *AFUA_4G06250* | Ribosome biogenesis (Nop4) |
| *NOP53* | *YPL146C* | *AFUA_2G05550* | Nop53p |
| *NOP56* | *YLR197W* | *AFUA_3G09600* | Essential evolutionarily-conserved nucleolar protein component of the box C/D snoRNP complexes that direct 2'-O-methylation of pre-rRNA during its maturation; overexpression causes spindle orientation defects |
| *NOP58* | *YOR310C* | *AFUA_3G13400* | Nucleolar protein nop5 |
| *NOP6* | *YDL213C* | *AFUA_3G06310* | RNA binding protein |
| *NOP7* | *YGR103W* | *AFUA_4G08190* | Component of several different pre-ribosomal particles; forms a complex with Ytm1p and Erb1p that is required for maturation of the large ribosomal subunit; required for exit from G0 and the initiation of cell proliferation |
| *NOP9* | *YJL010C* | *AFUA_3G11110* | Essential subunit of U3-containing 90S preribosome involved in production of 18S rRNA and assembly of small ribosomal subunit; also part of pre-40S ribosome and required for its export into cytoplasm; binds RNA and contains pumilio domain |
| *NOT3* | *YIL038C* | *AFUA_2G02070* | CCR4-NOT transcription complex, subunit 3 |
| *NPA3* | *YJR072C* | *AFUA_4G11820* | Essential, conserved, cytoplasmic ATPase; phosphorylated by the Pcl1p-Pho85p kinase complex |
| *NPC2* | *YDL046W* | *AFUA_2G11340* | Functional homolog of human NPC2/He1, which is a cholesterol-binding protein whose deficiency causes Niemann-Pick type C2 disease involving retention of cholesterol in lysosomes |
| *NPL3* | *YDR432W* | *AFUA_3G10100* | Pre-RNA splicing factor Srp2 |
| *NPP1* | *YCR026C YEL016C* | *AFUA_2G14770* | Type I phosphodiesterase / nucleotide pyrophosphatase family protein |
| *NPR3* | *YHL023C* | *AFUA_1G09760* | Component, with Npr2p, of an evolutionarily conserved complex that mediates downregulation of TOR Complex 1 activity in response to amino acid limitation; null mutant displays delayed meiotic DNA replication and double-strand break repair |
| *NPY1* | *YGL067W* | *AFUA_5G03360* | NADH diphosphatase (pyrophosphatase), hydrolyzes the pyrophosphate linkage in NADH and related nucleotides; localizes to peroxisomes |
| *NRD1* | *YNL251C* | *AFUA_5G04240* | RNA binding protein Nrd1 |
| *NRK1* | *YNL129W* | *AFUA_4G10840* | Nicotinamide riboside kinase, catalyzes the phosphorylation of nicotinamide riboside and nicotinic acid riboside in salvage pathways for NAD+ biosynthesis |
| *NRP1* | *YDL167C* | *AFUA_5G02160* | RNA binding protein (Arp) |
| *NSA2* | *YER126C* | *AFUA_3G08090* | 60S ribosomal protein Nsa2 |
| *NSE1* | *YLR007W* | *AFUA_3G02390* | Essential subunit of the Mms21-Smc5-Smc6 complex; nuclear protein required for DNA repair and growth; has a nonstructural role in the maintenance of chromosomes |
| *NSE4* | *YDL105W* | *AFUA_5G08660* | Nuclear protein Qri2/Nse4 |
| *NSG1* | *YHR133C YNL156C* | *AFUA_4G07680* | Nsg1p |
| *NSP1* | *YJL041W* | *AFUA_2G03310* | Essential component of the nuclear pore complex, which mediates nuclear import and export, found in both the Nup82 and Nic96 complexes |
| *NSR1* | *YGR159C* | *AFUA_3G07710* | Nucleolin protein Nsr1 |
| *NST1* | *YNL091W* | *AFUA_3G10350* | Stress response protein Nst1 |
| *NTA1* | *YJR062C* | *AFUA_5G08010* | Amidase, removes the amide group from N-terminal asparagine and glutamine residues to generate proteins with N-terminal aspartate and glutamate residues that are targets of ubiquitin-mediated degradation |
| *NTE1* | *YML059C* | *AFUA_4G03000* | Patatin-like serine hydrolase |
| *NTF2* | *YER009W* | *AFUA_3G10460* | Nuclear transport factor NTF-2 |
| *NUC1* | *YJL208C* | *AFUA_2G08750* | Major mitochondrial nuclease, has RNAse and DNA endo- and exonucleolytic activities; has roles in mitochondrial recombination, apoptosis and maintenance of polyploidy |
| *NUF2* | *YOL069W* | *AFUA_5G11350* | Component of the evolutionarily conserved kinetochore-associated Ndc80 complex (Ndc80p-Nuf2p-Spc24p-Spc25p); involved in chromosome segregation, spindle checkpoint activity and kinetochore clustering |
| *NUP1* | *YLR335W YOR098C* | *AFUA_3G12790 AFUA_2G10810* | Nucleoporin involved in nucleocytoplasmic transport, binds to either the nucleoplasmic or cytoplasmic faces of the nuclear pore complex depending on Ran-GTP levels; also has a role in chromatin organization |
| *NUP100* | *YMR047C YKL068W* | *AFUA_4G11070* | Nucleoporin SONB |
| *NUP120* | *YKL057C* | *AFUA_1G10400* | Nup120p |
| *NUP133* | *YKR082W* | *AFUA_4G05840* | Nuclear pore complex subunit Nup133 |
| *NUP157* | *YBL079W YER105C* | *AFUA_7G06020* | Abundant subunit of the nuclear pore complex (NPC), present on both sides of the NPC; has similarity to Nup170p; essential role, with Nup170p, in NPC assembly |
| *NUP159* | *YIL115C* | *AFUA_2G04920* | Nucleoporin, subunit of the nuclear pore complex that is found exclusively on the cytoplasmic side, member of the Nup82 subcomplex, required for mRNA export |
| *NUP188* | *YML103C* | *AFUA_1G13100* | Nucleoporin (Nup184) |
| *NUP192* | *YJL039C* | *AFUA_5G12670* | Essential structural subunit of the nuclear pore complex (NPC), localizes to the nuclear periphery of nuclear pores, homologous to human p205 |
| *NUP49* | *YGL172W* | *AFUA_6G10730* | Nucleoporin NUP49/NSP49 |
| *NUP57* | *YGR119C* | *AFUA_1G12280* | Nucleoporin, essential subunit of the nuclear pore complex (NPC), functions as the organizing center of an NPC subcomplex containing Nsp1p, Nup49p, Nup57p, and Nic96p |
| *NUP84* | *YDL116W* | *AFUA_1G10860* | Nuclear pore complex protein Nup107 |
| *NUS1* | *YDL193W* | *AFUA_8G02800* | Nus1p |
| *NUT1* | *YGL151W* | *AFUA_4G06600* | Component of the RNA polymerase II mediator complex, which is required for transcriptional activation and also has a role in basal transcription |
| *NUT2* | *YPR168W* | *AFUA_1G05940* | RNA polymerase II mediator complex protein Nut2, putative |
| *NYV1* | *YLR093C* | *AFUA_6G11270* | Synaptobrevin-like protein Sybl1 |
| *OAC1* | *YKL120W* | *AFUA_5G12360* | Mitochondrial inner membrane transporter, transports oxaloacetate, sulfate, thiosulfate, and isopropylmalate; member of the mitochondrial carrier family |
| *1-Oct* | *YKL134C* | *AFUA_6G08640* | Metallopeptidase Mip1 |
| *OGG1* | *YML060W* | *AFUA_7G05320* | Mitochondrial glycosylase/lyase that specifically excises 7,8-dihydro-8-oxoguanine residues located opposite cytosine or thymine residues in DNA, repairs oxidative damage to mitochondrial DNA, contributes to UVA resistance |
| *OLA1* | *YBR025C* | *AFUA_1G09800* | GTP-binding protein YchF |
| *OLE1* | *YGL055W* | *AFUA_7G05920* | Delta(9) fatty acid desaturase, required for monounsaturated fatty acid synthesis and for normal distribution of mitochondria |
| *OMA1* | *YKR087C* | *AFUA_4G09730* | Metalloendopeptidase of the mitochondrial inner membrane, involved in turnover of membrane-embedded proteins; member of a family of predicted membrane-bound metallopeptidases in prokaryotes and higher eukaryotes |
| *OMS1* | *YDR316W* | *AFUA_3G10140* | Ubiquinone/menaquinone biosynthesis-related protein |
| *OPI1* | *YHL020C* | *AFUA_5G09420* | Clock controled protein (Ccg-8) |
| *OPI10* | *YOL032W* | *AFUA_5G11100* | DUF775 domain protein |
| *OPI3* | *YJR073C* | *AFUA_1G09050* | Phospholipid methyltransferase |
| *ORC2* | *YBR060C* | *AFUA_5G08110* | Origin recognition complex subunit 2 |
| *ORC3* | *YLL004W* | *AFUA_8G04240* | Origin recognition complex subunit 3 |
| *ORC4* | *YPR162C* | *AFUA_2G11170* | Origin recognition complex subunit Orc4 |
| *ORM1* | *YGR038W YLR350W* | *AFUA_4G13270* | Evolutionarily conserved protein, similar to Orm2p, required for resistance to agents that induce unfolded protein response; Orm1p and Orm2p together control membrane biogenesis by coordinating lipid homeostasis with protein quality control |
| *ORT1* | *YOR130C* | *AFUA_8G02760* | Ornithine transporter of the mitochondrial inner membrane, exports ornithine from mitochondria as part of arginine biosynthesis; human ortholog is associated with hyperammonaemia-hyperornithinaemia-homocitrullinuria syndrome |
| *OSM1* | *YEL047C YJR051W* | *AFUA_7G05070 AFUA_8G05530* | Soluble fumarate reductase, required with isoenzyme Osm1p for anaerobic growth; may interact with ribosomes, based on co-purification experiments; authentic, non-tagged protein is detected in purified mitochondria in high-throughput studies |
| *OST1* | *YJL002C* | *AFUA_2G05790* | Alpha subunit of the oligosaccharyltransferase complex of the ER lumen, which catalyzes asparagine-linked glycosylation of newly synthesized proteins |
| *OST2* | *YOR103C* | *AFUA_1G03810* | Epsilon subunit of the oligosaccharyltransferase complex of the ER lumen, which catalyzes asparagine-linked glycosylation of newly synthesized proteins |
| *OST3* | *YOR085W* | *AFUA_2G06280* | Gamma subunit of the oligosaccharyltransferase complex of the ER lumen, which catalyzes asparagine-linked glycosylation of newly synthesized proteins; Ost3p is important for N-glycosylation of a subset of proteins |
| *OTU1* | *YFL044C* | *AFUA_3G05550* | Deubiquitylation enzyme that binds to the chaperone-ATPase Cdc48p; may contribute to regulation of protein degradation by deubiquitylating substrates that have been ubiquitylated by Ufd2p; member of the Ovarian Tumor family |
| *OTU2* | *YHL013C* | *AFUA_4G13560* | OTU-like cysteine protease |
| *OXA1* | *YER154W* | *AFUA_5G03640* | Mitochondrial inner membrane insertase, mediates the insertion of both mitochondrial- and nuclear-encoded proteins from the matrix into the inner membrane, interacts with mitochondrial ribosomes; conserved from bacteria to animals |
| *OXR1* | *YPL196W* | *AFUA_3G08740* | Oxidative stress response protein Oxr1 |
| *PAA1* | *YDR071C* | *AFUA_2G11500* | Acetyltransferase, GNAT family family |
| *PAB1* | *YER165W* | *AFUA_1G04190* | Polyadenylate-binding protein |
| *PAC10* | *YGR078C* | *AFUA_2G02230* | Prefoldin subunit 3 |
| *PAC2* | *YER007W* | *AFUA_4G11870* | Microtubule effector required for tubulin heterodimer formation, binds alpha-tubulin, required for normal microtubule function, null mutant exhibits cold-sensitive microtubules and sensitivity to benomyl |
| *PAF1* | *YBR279W* | *AFUA_2G11000* | Component of the Paf1p complex that binds to and modulates the activity of RNA polymerases I and II; required for expression of a subset of genes, including cell cycle-regulated genes; homolog of human PD2/hPAF1 |
| *PAM16* | *YJL104W* | *AFUA_7G04150* | Constituent of the import motor component of the Translocase of the Inner Mitochondrial membrane (TIM23 complex); forms a 1:1 subcomplex with Pam18p and inhibits its cochaperone activity; contains a J-like domain |
| *PAM17* | *YKR065C* | *AFUA_1G01870* | Constituent of the Translocase of the Inner Mitochondrial membrane or to interact with and modulate the core TIM23 complex |
| *PAM18* | *YLR008C* | *AFUA_2G02700* | Constituent of the import motor component of the Translocase of the Inner Mitochondrial membrane (TIM23 complex); essential J-protein cochaperone that stimulates Ssc1p ATPase activity to drive import; inhibited by Pam16p |
| *PAN1* | *YIR006C* | *AFUA_7G03870* | Actin cortical patch assembly protein Pan1 |
| *PAN5* | *YHR063C* | *AFUA_3G06390 AFUA_3G13550* | 2-dehydropantoate 2-reductase, part of the pantothenic acid pathway, structurally homologous to E. coli panE |
| *PAP1* | *YKR002W* | *AFUA_1G15340* | Poly(A) polymerase Pap |
| *PAT1* | *YCR077C* | *AFUA_5G10770* | Topisomerase II associated protein (Pat1) |
| *PBN1* | *YCL052C* | *AFUA_5G06810* | Essential component of glycosylphosphatidylinositol-mannosyltransferase I, required for the autocatalytic post-translational processing of the protease B precursor Prb1p, localizes to ER in lumenal orientation; homolog of mammalian PIG-X |
| *PBP1* | *YGR178C* | *AFUA_1G09630* | Component of glucose deprivation induced stress granules, involved in P-body-dependent granule assembly; similar to human ataxin-2; interacts with Pab1p to regulate mRNA polyadenylation; interacts with Mkt1p to regulate HO translation |
| *PBS2* | *YJL128C* | *AFUA_1G15950* | MAP kinase kinase that plays a pivotal role in the osmosensing signal-transduction pathway, activated under severe osmotic stress; plays a role in regulating Ty1 transposition |
| *PBY1* | *YBR094W* | *AFUA_3G10220* | Acid phosphatase |
| *PCA1* | *YBR295W* | *AFUA_1G16130* | Cadmium transporting P-type ATPase; may also have a role in copper and iron homeostasis; stabilized by Cd binding, which prevents ubiquitination; S288C and other lab strains contain a G970R mutation which eliminates Cd transport function |
| *PCD1* | *YLR151C* | *AFUA_3G08220* | NUDIX domain protein |
| *PCF11* | *YDR228C* | *AFUA_1G07510* | MRNA cleavage factor complex component Pcf11 |
| *PCH2* | *YBR186W* | *AFUA_1G02060* | Nucleolar component of the pachytene checkpoint, which prevents chromosome segregation when recombination and chromosome synapsis are defective; also represses meiotic interhomolog recombination in the rDNA |
| *PCK1* | *YKR097W* | *AFUA_6G07720* | Phosphoenolpyruvate carboxykinase AcuF |
| *PCL5* | *YHR071W* | *AFUA_6G02020* | Cyclin, interacts with and phosphorylated by Pho85p cyclin-dependent kinase (Cdk), induced by Gcn4p at level of transcription, specifically required for Gcn4p degradation, may be sensor of cellular protein biosynthetic capacity |
| *PCL6* | *YER059W YIL050W* | *AFUA_7G04640* | Cyclin-dependent protein kinase complex component, putative |
| *PCP1* | *YGR101W* | *AFUA_1G09150* | Mitochondrial serine protease required for the processing of various mitochondrial proteins and maintenance of mitochondrial DNA and morphology; belongs to the rhomboid-GlpG superfamily of intramembrane peptidases |
| *PCS60* | *YBR222C* | *AFUA_4G06850* | Coenzyme A synthetase |
| *PCT1* | *YGR202C* | *AFUA_1G09290* | Cholinephosphate cytidylyltransferase, also known as CTP:phosphocholine cytidylyltransferase, rate-determining enzyme of the CDP-choline pathway for phosphatidylcholine synthesis, inhibited by Sec14p, activated upon lipid-binding |
| *PDA1* | *YER178W* | *AFUA_1G06960* | E1 alpha subunit of the pyruvate dehydrogenase complex, catalyzes the direct oxidative decarboxylation of pyruvate to acetyl-CoA; phosphorylated; regulated by glucose |
| *PDB1* | *YBR221C* | *AFUA_3G04170* | E1 beta subunit of the pyruvate dehydrogenase complex, which is an evolutionarily-conserved multi-protein complex found in mitochondria |
| *PDE1* | *YGL248W* | *AFUA_1G14890* | Low-affinity cyclic AMP phosphodiesterase, controls glucose and intracellular acidification-induced cAMP signaling, target of the cAMP-protein kinase A pathway; glucose induces transcription and inhibits translation |
| *PDE2* | *YOR360C* | *AFUA_1G05230* | High-affinity cyclic AMP phosphodiesterase, component of the cAMP-dependent protein kinase signaling system, protects the cell from extracellular cAMP, contains readthrough motif surrounding termination codon |
| *PDH1* | *YPR002W* | *AFUA_6G03730* | Mitochondrial protein that participates in respiration, induced by diauxic shift; homologous to E. coli PrpD, may take part in the conversion of 2-methylcitrate to 2-methylisocitrate |
| *PDX1* | *YGR193C* | *AFUA_3G08270* | Dihydrolipoamide dehydrogenase of the mitochondrial pyruvate dehydrogenase complex, plays a structural role in the complex by binding and positioning E3 to the dihydrolipoamide acetyltransferase core |
| *PEF1* | *YGR058W* | *AFUA_3G08540* | Calcium binding modulator protein (Alg2) |
| *PEP12* | *YOR036W* | *AFUA_4G07110* | SNARE domain protein |
| *PEP3* | *YLR148W* | *AFUA_5G06450* | Component of CORVET tethering complex; vacuolar peripheral membrane protein that promotes vesicular docking/fusion reactions in conjunction with SNARE proteins, required for vacuolar biogenesis |
| *PEP4* | *YPL154C* | *AFUA_3G11400* | Aspartic endopeptidase Pep2 |
| *PEP5* | *YMR231W* | *AFUA_6G10410* | Component of CORVET tethering complex; peripheral vacuolar membrane protein required for protein trafficking and vacuole biogenesis; interacts with Pep7p |
| *PEP7* | *YDR323C* | *AFUA_3G13770* | Multivalent adaptor protein that facilitates vesicle-mediated vacuolar protein sorting by ensuring high-fidelity vesicle docking and fusion, which are essential for targeting of vesicles to the endosome; required for vacuole inheritance |
| *PEP8* | *YJL053W* | *AFUA_4G12060* | Vacuolar protein sorting-associated protein 26, putative |
| *PER1* | *YCR044C* | *AFUA_1G06200* | Mn2+ homeostasis protein (Per1) |
| *PET100* | *YDR079W* | *AFUA_6G04030* | Chaperone that specifically facilitates the assembly of cytochrome c oxidase, integral to the mitochondrial inner membrane; interacts with a subcomplex of subunits VII, VIIa, and VIII but not with the holoenzyme |
| *PET112* | *YBL080C* | *AFUA_3G11440* | Glutamyl-tRNA(Gln) amidotransferase, B subunit |
| *PET117* | *YER058W* | *AFUA_7G02030* | Cytochrome c oxidase assembly protein (Pet117), putative |
| *PET127* | *YOR017W* | *AFUA_6G04720* | Mitochondrial mRNA processing protein PET127 |
| *PET191* | *YJR034W* | *AFUA_5G08965* | Cytochrome c oxidase assembly protein (Pet191), putative |
| *PEX1* | *YKL197C* | *AFUA_2G10150* | AAA-peroxin that heterodimerizes with AAA-peroxin Pex6p and participates in the recycling of peroxisomal signal receptor Pex5p from the peroxisomal membrane to the cystosol; induced by oleic acid and upregulated during anaerobiosis |
| *PEX11* | *YOL147C* | *AFUA_6G07740* | Peroxisomal membrane protein required for peroxisome proliferation and medium-chain fatty acid oxidation; localization regulated by phosphorylation; transcription regulated by Adr1p and Pip2p-Oaf1p |
| *PEX12* | *YMR026C* | *AFUA_2G16730* | C3HC4-type RING-finger peroxisomal membrane peroxin required for peroxisome biogenesis and peroxisomal matrix protein import; forms translocation subcomplex with Pex2p and Pex10p; mutations in human homolog cause peroxisomal disorders |
| *PEX13* | *YLR191W* | *AFUA_8G05160* | Integral peroxisomal membrane required for the translocation of peroxisomal matrix proteins, interacts with the PTS1 signal recognition factor Pex5p and the PTS2 signal recognition factor Pex7p, forms a complex with Pex14p and Pex17p |
| *PEX14* | *YGL153W* | *AFUA_3G10190* | Peroxisomal membrane peroxin that is a central component of the peroxisomal protein import machinery; interacts with both PTS1 and PTS2 (Pex7p), peroxisomal matrix protein signal recognition factors and membrane receptor Pex13p |
| *PEX19* | *YDL065C* | *AFUA_3G10940* | Chaperone and import receptor for newly-synthesized class I peroxisomal membrane proteins (PMPs), binds PMPs in the cytoplasm and delivers them to the peroxisome for subsequent insertion into the peroxisomal membrane |
| *PEX29* | *YDR479C* | *AFUA_2G01510* | Peroxisomal integral membrane peroxin, involved in the regulation of peroxisomal size, number and distribution; genetic interactions suggest that Pex28p and Pex29p act at steps upstream of those mediated by Pex30p, Pex31p, and Pex32p |
| *PEX30* | *YGR004W YLR324W* | *AFUA_7G04160* | Peroxisomal integral membrane protein, involved in negative regulation of peroxisome number; partially functionally redundant with Pex31p; genetic interactions suggest action at a step downstream of steps mediated by Pex28p and Pex29p |
| *PEX4* | *YGR133W* | *AFUA_2G01130* | Peroxisomal ubiquitin conjugating enzyme required for peroxisomal matrix protein import and peroxisome biogenesis |
| *PEX5* | *YDR244W* | *AFUA_8G05240* | Peroxisomal membrane signal receptor for the C-terminal tripeptide signal sequence of peroxisomal matrix proteins, required for peroxisomal matrix protein import; also proposed to have PTS1-receptor independent functions |
| *PEX6* | *YNL329C* | *AFUA_3G08000* | AAA-peroxin that heterodimerizes with AAA-peroxin Pex1p and participates in the recycling of peroxisomal signal receptor Pex5p from the peroxisomal membrane to the cystosol |
| *PEX7* | *YDR142C* | *AFUA_1G15400* | Peroxisomal signal receptor for the N-terminal nonapeptide signal of peroxisomal matrix proteins; WD repeat protein; defects in human homolog cause lethal rhizomelic chondrodysplasia punctata (RCDP) |
| *PFA3* | *YNL326C* | *AFUA_2G16480* | Palmitoyltransferase for Vac8p, required for vacuolar membrane fusion; contains an Asp-His-His-Cys-cysteine rich domain; autoacylates; required for vacuolar integrity under stress conditions |
| *PFA4* | *YOL003C* | *AFUA_8G05830* | Palmitoyltransferase with autoacylation activity, required for palmitoylation of amino acid permeases containing a C-terminal Phe-Trp-Cys site; required for modification of Chs3p; member of the DHHC family of putative palmitoyltransferases |
| *PFK1* | *YGR240C YMR205C* | *AFUA_4G00960* | Alpha subunit of heterooctameric phosphofructokinase involved in glycolysis, indispensable for anaerobic growth, activated by fructose-2,6-bisphosphate and AMP, mutation inhibits glucose induction of cell cycle-related genes |
| *PFK26* | *YIL107C* | *AFUA_1G07220* | 6-phosphofructo-2-kinase 1 |
| *PFS2* | *YNL317W* | *AFUA_2G08390* | Integral subunit of the pre-mRNA cleavage and polyadenylation factor complex; plays an essential role in mRNA 3'-end formation by bridging different processing factors and thereby promoting the assembly of the processing complex |
| *PFY1* | *YOR122C* | *AFUA_4G03050* | Profilin Pfy1 |
| *PGC1* | *YPL206C* | *AFUA_2G00990* | Glycerophosphoryl diester phosphodiesterase family protein |
| *PGI1* | *YBR196C* | *AFUA_2G09790* | Glycolytic enzyme phosphoglucose isomerase, catalyzes the interconversion of glucose-6-phosphate and fructose-6-phosphate; required for cell cycle progression and completion of the gluconeogenic events of sporulation |
| *PGK1* | *YCR012W* | *AFUA_1G10350* | 3-phosphoglycerate kinase, catalyzes transfer of high-energy phosphoryl groups from the acyl phosphate of 1,3-bisphosphoglycerate to ADP to produce ATP; key enzyme in glycolysis and gluconeogenesis |
| *PGM1* | *YMR105C YKL127W* | *AFUA_3G11830* | Phosphoglucomutase PgmA |
| *PGS1* | *YCL004W* | *AFUA_5G06530* | CDP-diacylglycerol-glycerol-3-phosphate 3-phosphatidyltransferase |
| *PHA2* | *YNL316C* | *AFUA_5G05690* | Chorismate mutase/prephenate dehydratase |
| *PHD1* | *YMR016C YKL043W* | *AFUA_2G07900* | Nuclear protein that plays a regulatory role in the cyclic AMP signal transduction pathway; negatively regulates pseudohyphal differentiation; homologous to several transcription factors |
| *PHM7* | *YMR266W YOL084W* | *AFUA_5G01820 AFUA_5G10920* | Membrane protein of unknown function; overexpression suppresses NaCl sensitivity of sro7 mutant cells by restoring sodium pump localization to the plasma membrane |
| *PHM8* | *YER037W YGL224C* | *AFUA_2G13470* | Pyrimidine nucleotidase; overexpression suppresses the 6-AU sensitivity of transcription elongation factor S-II, as well as resistance to other pyrimidine derivatives |
| *PHO13* | *YDL236W* | *AFUA_3G08310* | Alkaline phosphatase specific for p-nitrophenyl phosphate; also has protein phosphatase activity |
| *PHO2* | *YDL106C* | *AFUA_4G10220* | Homeobox transcription factor; regulatory targets include genes involved in phosphate metabolism; binds cooperatively with Pho4p to the PHO5 promoter; phosphorylation of Pho2p facilitates interaction with Pho4p |
| *PHO4* | *YFR034C* | *AFUA_5G04190* | Basic helix-loop-helix transcription factor of the myc-family; binds cooperatively with Pho2p to the PHO5 promoter; function is regulated by phosphorylation at multiple sites and by phosphate availability |
| *PHO8* | *YDR481C* | *AFUA_2G03110* | Alkaline phosphatase Pho8 |
| *PHO80* | *YOL001W* | *AFUA_1G07070* | Cyclin, interacts with cyclin-dependent kinase Pho85p; regulates the response to nutrient levels and environmental conditions, including the response to phosphate limitation and stress-dependent calcium signaling |
| *PHO84* | *YML123C* | *AFUA_4G03610* | High-affinity inorganic phosphate transporter and low-affinity manganese transporter; regulated by Pho4p and Spt7p; mutation confers resistance to arsenate; exit from the ER during maturation requires Pho86p |
| *PHO85* | *YPL031C* | *AFUA_5G04130* | Cyclin-dependent kinase, with ten cyclin partners; involved in regulating the cellular response to nutrient levels and environmental conditions and progression through the cell cycle |
| *PHR1* | *YOR386W* | *AFUA_1G01600* | DNA photolyase involved in photoreactivation, repairs pyrimidine dimers in the presence of visible light; induced by DNA damage; regulated by transcriptional repressor Rph1p |
| *PHS1* | *YJL097W* | *AFUA_4G10270* | Essential 3-hydroxyacyl-CoA dehydratase of the ER membrane, involved in elongation of very long-chain fatty acids; evolutionarily conserved, similar to mammalian PTPLA and PTPLB; involved in sphingolipid biosynthesis and protein trafficking |
| *PIB1* | *YDR313C* | *AFUA_7G01620* | Pib1p |
| *PIB2* | *YGL023C* | *AFUA_1G17070* | FYVE domain protein |
| *PIF1* | *YML061C* | *AFUA_5G13370* | DNA helicase; exists in two forms; the nuclear form is involved in telomere formation and elongation; acts as a catalytic inhibitor of telomerase; the mitochondrial form is involved in repair and recombination of mitochondrial DNA |
| *PIM1* | *YBL022C* | *AFUA_2G11740* | ATP-dependent Lon protease, involved in degradation of misfolded proteins in mitochondria; required for biogenesis and maintenance of mitochondria |
| *PIN4* | *YBL051C* | *AFUA_5G01940* | R3H domain protein |
| *PIS1* | *YPR113W* | *AFUA_1G15790* | Phosphatidylinositol synthase, required for biosynthesis of phosphatidylinositol, which is a precursor for polyphosphoinositides, sphingolipids, and glycolipid anchors for some of the plasma membrane proteins |
| *PKC1* | *YBL105C* | *AFUA_5G11970* | Protein kinase c |
| *PKH1* | *YOL100W YDR490C* | *AFUA_3G12670* | Serine/threonine protein kinase |
| *PKP2* | *YGL059W* | *AFUA_5G03240* | Mitochondrial protein kinase that negatively regulates activity of the pyruvate dehydrogenase complex by phosphorylating the ser-133 residue of the Pda1p subunit; acts in concert with kinase Pkp1p and phosphatases Ptc5p and Ptc6p |
| *PLM2* | *YLR183C YDR501W* | *AFUA_7G05440* | Forkhead Associated domain containing protein and putative transcription factor found associated with chromatin; target of SBF transcription factor; induced in response to DNA damaging agents and deletion of telomerase; similar to TOS4 |
| *PLP1* | *YDR183W* | *AFUA_5G05760* | NTP binding protein |
| *PMA1* | *YGL008C YPL036W* | *AFUA_1G02480* | Plasma membrane H+-ATPase, pumps protons out of the cell; major regulator of cytoplasmic pH and plasma membrane potential; part of the P2 subgroup of cation-transporting ATPases |
| *PMC1* | *YGL006W* | *AFUA_1G10880 AFUA_3G10690 AFUA_7G01030* | Calcium-translocating P-type ATPase(PMCA-type),putative |
| *PMI40* | *YER003C* | *AFUA_1G13280* | Mannose-6-phosphate isomerase, catalyzes the interconversion of fructose-6-P and mannose-6-P; required for early steps in protein mannosylation |
| *PML1* | *YLR016C* | *AFUA_3G11540* | FHA domain protein SNIP1 |
| *PMP3* | *YDR276C* | *AFUA_3G12350 AFUA_5G03230* | Stress response RCI peptide |
| *PMR1* | *YGL167C* | *AFUA_2G05860* | High affinity Ca2+/Mn2+ P-type ATPase required for Ca2+ and Mn2+ transport into Golgi; involved in Ca2+ dependent protein sorting and processing; mutations in human homolog ATP2C1 cause acantholytic skin condition Hailey-Hailey disease |
| *PMS1* | *YNL082W* | *AFUA_2G13410* | ATP-binding protein required for mismatch repair in mitosis and meiosis; functions as a heterodimer with Mlh1p, binds double- and single-stranded DNA via its N-terminal domain, similar to E. coli MutL |
| *PMT2* | *YOR321W YAL023C* | *AFUA_1G07690* | Protein O-mannosyl transferase |
| *PMT4* | *YJR143C* | *AFUA_8G04500* | Mannosyltransferase PMTI |
| *PNC1* | *YGL037C* | *AFUA_2G03770* | Nicotinamidase that converts nicotinamide to nicotinic acid as part of the NAD(+) salvage pathway, required for life span extension by calorie restriction; PNC1 expression responds to all known stimuli that extend replicative life span |
| *PNG1* | *YPL096W* | *AFUA_2G04000* | Conserved peptide N-glycanase required for deglycosylation of misfolded glycoproteins during proteasome-dependent degradation; localizes to the cytoplasm and nucleus; activity is enhanced by interaction with Rad23p |
| *PNO1* | *YOR145C* | *AFUA_6G06430* | Essential nucleolar protein required for pre-18S rRNA processing, interacts with Dim1p, an 18S rRNA dimethyltransferase, and also with Nob1p, which is involved in proteasome biogenesis; contains a KH domain |
| *PNS1* | *YOR161C* | *AFUA_3G12970* | DUF580 domain protein Pns1 |
| *POA1* | *YBR022W* | *AFUA_1G16556* | Phosphatase that is highly specific for ADP-ribose 1''-phosphate, a tRNA splicing metabolite; may have a role in regulation of tRNA splicing |
| *POB3* | *YML069W* | *AFUA_7G05360* | Structure-specific recognition protein |
| *POL1* | *YNL102W* | *AFUA_2G10730* | Catalytic subunit of the DNA polymerase I alpha-primase complex, required for the initiation of DNA replication during mitotic DNA synthesis and premeiotic DNA synthesis |
| *POL12* | *YBL035C* | *AFUA_5G13020* | B subunit of DNA polymerase alpha-primase complex, required for initiation of DNA replication during mitotic and premeiotic DNA synthesis; also functions in telomere capping and length regulation |
| *POL2* | *YNL262W* | *AFUA_3G09560* | Catalytic subunit of DNA polymerase epsilon, a chromosomal DNA replication polymerase that exhibits processivity and proofreading exonuclease activity; also involved in DNA synthesis during DNA repair; interacts extensively with Mrc1p |
| *POL3* | *YDL102W* | *AFUA_2G16600* | Catalytic subunit of DNA polymerase delta; required for chromosomal DNA replication during mitosis and meiosis, intragenic recombination, repair of double strand DNA breaks, and DNA replication during nucleotide excision repair (NER) |
| *POL30* | *YBR088C* | *AFUA_1G04900* | Proliferating cell nuclear antigen (PCNA) |
| *POL4* | *YCR014C* | *AFUA_1G10480* | DNA polymerase IV, undergoes pair-wise interactions with Dnl4p-Lif1p and Rad27p to mediate repair of DNA double-strand breaks by non-homologous end joining (NHEJ); homologous to mammalian DNA polymerase beta |
| *POL5* | *YEL055C* | *AFUA_6G08170* | DNA Polymerase phi; has sequence similarity to the human MybBP1A and weak sequence similarity to B-type DNA polymerases, not required for chromosomal DNA replication; required for the synthesis of rRNA |
| *POM152* | *YMR129W* | *AFUA_3G05500* | Nuclear pore membrane glycoprotein; may be involved in duplication of nuclear pores and nuclear pore complexes during S-phase |
| *POP1* | *YNL221C* | *AFUA_6G02270* | Ribonuclease P complex subunit Pop1 |
| *POP2* | *YNR052C* | *AFUA_5G07370* | CCR4-NOT core complex subunit Caf1 |
| *POP4* | *YBR257W* | *AFUA_5G11150* | Ribonuclease P complex subunit Pop4 |
| *POR1* | *YIL114C YNL055C* | *AFUA_4G06910* | Mitochondrial porin (voltage-dependent anion channel), outer membrane protein required for the maintenance of mitochondrial osmotic stability and mitochondrial membrane permeability; phosphorylated |
| *POS5* | *YPL188W* | *AFUA_5G05890* | Mitochondrial NADH kinase, phosphorylates NADH; also phosphorylates NAD(+) with lower specificity; required for the response to oxidative stress |
| *POT1* | *YIL160C* | *AFUA_7G04080* | 3-ketoacyl-CoA thiolase with broad chain length specificity, cleaves 3-ketoacyl-CoA into acyl-CoA and acetyl-CoA during beta-oxidation of fatty acids |
| *POX1* | *YGL205W* | *AFUA_7G06090* | Fatty-acyl coenzyme A oxidase, involved in the fatty acid beta-oxidation pathway; localized to the peroxisomal matrix |
| *PPA1* | *YHR026W* | *AFUA_2G15560* | V-ATPase proteolipid subunit Ppa1 |
| *PPE1* | *YHR075C* | *AFUA_1G03080* | Ribosomal protein/carboxylic ester hydrolase (Ppe1), putative |
| *PPM2* | *YOL141W* | *AFUA_5G11670* | AdoMet-dependent tRNA methyltransferase also involved in methoxycarbonylation; required for the synthesis of wybutosine (yW), a modified guanosine found at the 3'-position adjacent to the anticodon of phe-tRNA; similarity to Ppm1p |
| *PPN1* | *YDR452W* | *AFUA_1G11490* | Vacuolar endopolyphosphatase |
| *PPR1* | *YLR014C* | *AFUA_1G15470* | C6 transcription factor (UaY) |
| *PPT1* | *YGR123C* | *AFUA_5G06700* | Serine/threonine protein phosphatase PPT1 |
| *PPX1* | *YHR201C* | *AFUA_5G03410* | Exopolyphosphatase, hydrolyzes inorganic polyphosphate into Pi residues; located in the cytosol, plasma membrane, and mitochondrial matrix |
| *PPZ1* | *YML016C YDR436W* | *AFUA_2G03950* | Serine/threonine protein phosphatase |
| *PRC1* | *YMR297W* | *AFUA_6G13540* | Carboxypeptidase CpyA/Prc1 |
| *PRD1* | *YCL057W* | *AFUA_7G05930* | Metallopeptidase MepB |
| *PRE1* | *YER012W* | *AFUA_4G07510* | Beta 4 subunit of the 20S proteasome; localizes to the nucleus throughout the cell cycle |
| *PRE10* | *YOR362C* | *AFUA_6G06350* | Alpha 7 subunit of the 20S proteasome |
| *PRE2* | *YPR103W* | *AFUA_6G08310* | Beta 5 subunit of the 20S proteasome, responsible for the chymotryptic activity of the proteasome |
| *PRE3* | *YJL001W* | *AFUA_7G04650* | Beta 1 subunit of the 20S proteasome, responsible for cleavage after acidic residues in peptides |
| *PRE4* | *YFR050C* | *AFUA_6G06450* | Beta 7 subunit of the 20S proteasome |
| *PRE5* | *YMR314W* | *AFUA_6G04790* | Alpha 6 subunit of the 20S proteasome |
| *PRE6* | *YOL038W* | *AFUA_5G02150* | Alpha 4 subunit of the 20S proteasome; may replace alpha 3 subunit under stress conditions to create a more active proteasomal isoform; GFP-fusion protein relocates from cytosol to the mitochondrial surface upon oxidative stress |
| *PRE7* | *YBL041W* | *AFUA_6G06440* | Beta 6 subunit of the 20S proteasome |
| *PRE8* | *YML092C* | *AFUA_7G05870* | Alpha 2 subunit of the 20S proteasome |
| *PRE9* | *YGR135W* | *AFUA_6G08960* | Alpha 3 subunit of the 20S proteasome, the only nonessential 20S subunit; may be replaced by the alpha 4 subunit under stress conditions to create a more active proteasomal isoform |
| *PRI1* | *YIR008C* | *AFUA_3G09020* | DNA primase subunit Pri1 |
| *PRI2* | *YKL045W* | *AFUA_1G03410* | DNA primase large subunit |
| *PRM1* | *YNL279W* | *AFUA_4G11210* | Pheromone-regulated multispanning membrane protein involved in membrane fusion during mating; predicted to have 5 transmembrane segments and a coiled coil domain; localizes to the shmoo tip; regulated by Ste12p |
| *PRM10* | *YJL108C* | *AFUA_5G07620 AFUA_3G13940 AFUA_3G01440 AFUA_4G03340* | DUF1212 domain membrane protein |
| *PRM6* | *YML047C YJR054W* | *AFUA_2G04400* | Pheromone-regulated protein, predicted to have 2 transmembrane segments; regulated by Ste12p during mating |
| *PRO3* | *YER023W* | *AFUA_3G02310* | Delta 1-pyrroline-5-carboxylate reductase, catalyzes the last step in proline biosynthesis |
| *PRP11* | *YDL043C* | *AFUA_4G11880* | Splicing factor 3a subunit 2 |
| *PRP16* | *YKR086W* | *AFUA_1G03820* | MRNA splicing factor RNA helicase (Prp16) |
| *PRP18* | *YGR006W* | *AFUA_1G16990* | MRNA splicing factor (Prp18) |
| *PRP19* | *YLL036C* | *AFUA_5G13510* | Cell cycle control protein (Cwf8) |
| *PRP21* | *YJL203W* | *AFUA_3G06440* | Pre-mRNA splicing factor |
| *PRP22* | *YER013W* | *AFUA_5G10830* | DEAH-box RNA-dependent ATPase/ATP-dependent RNA helicase, associates with lariat intermediates before the second catalytic step of splicing; mediates ATP-dependent mRNA release from the spliceosome and unwinds RNA duplexes |
| *PRP24* | *YMR268C* | *AFUA_2G01820* | Pre-mRNA splicing factor (Prp24) |
| *PRP28* | *YDR243C* | *AFUA_4G09020* | DEAD/DEAH box helicase |
| *PRP3* | *YDR473C* | *AFUA_1G13890* | U4/U6 small nuclear ribonucleoprotein |
| *PRP31* | *YGR091W* | *AFUA_1G10190* | Pre-mRNA splicing factor (Prp31) |
| *PRP39* | *YML046W* | *AFUA_4G09010* | MRNA splicing protein (Prp39) |
| *PRP4* | *YPR178W* | *AFUA_8G04590* | Pre-mRNA splicing factor |
| *PRP40* | *YKL012W* | *AFUA_1G10320* | Formin binding protein (FNB3) |
| *PRP43* | *YGL120C* | *AFUA_5G11620* | Pre-mRNA splicing factor RNA helicase (Prp43) |
| *PRP45* | *YAL032C* | *AFUA_5G03050* | Protein required for pre-mRNA splicing; associates with the spliceosome and interacts with splicing factors Prp22p and Prp46p; orthologous to human transcriptional coactivator SKIP and can activate transcription of a reporter gene |
| *PRP46* | *YPL151C* | *AFUA_1G10710* | Member of the NineTeen Complex that contains Prp19p and stabilizes U6 snRNA in catalytic forms of the spliceosome containing U2, U5, and U6 snRNAs |
| *PRP5* | *YBR237W* | *AFUA_1G10050* | DEAD/DEAH box RNA helicase |
| *PRP6* | *YBR055C* | *AFUA_2G06070* | MRNA splicing factor (Prp1/Zer1) |
| *PRP8* | *YHR165C* | *AFUA_2G03030* | Component of the U4/U6-U5 snRNP complex, involved in the second catalytic step of splicing; mutations of human Prp8 cause retinitis pigmentosa |
| *PRP9* | *YDL030W* | *AFUA_5G02420* | Splicing factor 3a subunit 3 |
| *PRR1* | *YKL116C* | *AFUA_2G14200* | Protein kinase |
| *PRT1* | *YOR361C* | *AFUA_1G02030* | Eukaryotic translation initiation factor 3 subunit EifCb |
| *PRX1* | *YBL064C* | *AFUA_4G08580 AFUA_8G07130* | Mitochondrial peroxiredoxin with thioredoxin peroxidase activity, has a role in reduction of hydroperoxides; reactivation requires Trr2p and glutathione; induced during respiratory growth and oxidative stress; phosphorylated |
| *PRY1* | *YJL078C YJL079C YKR013W* | *AFUA_1G02040* | Extracellular SCP domain protein Pry1 |
| *PSD1* | *YNL169C* | *AFUA_1G15760* | Phosphatidylserine decarboxylase of the mitochondrial inner membrane, converts phosphatidylserine to phosphatidylethanolamine |
| *PSD2* | *YGR170W* | *AFUA_3G13970* | Phosphatidylserine decarboxylase of the Golgi and vacuolar membranes, converts phosphatidylserine to phosphatidylethanolamine |
| *PSE1* | *YMR308C* | *AFUA_1G06790* | Karyopherin/importin that interacts with the nuclear pore complex; acts as the nuclear import receptor for specific proteins, including Pdr1p, Yap1p, Ste12p, and Aft1p |
| *PSF1* | *YDR013W* | *AFUA_4G12430* | PSF1 domain protein |
| *PSF2* | *YJL072C* | *AFUA_2G11090* | DNA replication complex GINS protein (Psf2) |
| *PSF3* | *YOL146W* | *AFUA_4G11620* | GINS complex subunit Psf3 |
| *PSH1* | *YOL054W* | *AFUA_2G15830* | Nuclear protein, putative RNA polymerase II elongation factor; isolated as Pob3p/Spt16p-binding protein |
| *PSK1* | *YOL045W YAL017W* | *AFUA_2G02850* | One of two PAS domain containing S/T protein kinases; regulates sugar flux and translation in response to an unknown metabolite by phosphorylating Ugp1p and Gsy2p and Caf20p, Tif11p and Sro9p (translation) |
| *PSO2* | *YMR137C* | *AFUA_2G15220* | Nuclease required for a post-incision step in the repair of DNA single and double-strand breaks that result from interstrand crosslinks produced by a variety of mono- and bi-functional psoralen derivatives; induced by UV-irradiation |
| *PSP1* | *YLR177W YDR505C* | *AFUA_1G04670* | Putative protein of unknown function; phosphorylated by Dbf2p-Mob1p in vitro; some strains contain microsatellite polymophisms at this locus; YLR177W is not an essential gene |
| *PSR1* | *YLL010C YLR019W* | *AFUA_1G04790* | Functionally redundant Psr1p homolog, a plasma membrane phosphatase involved in the general stress response; required with Psr1p and Whi2p for full activation of STRE-mediated gene expression, possibly through dephosphorylation of Msn2p |
| *PTC1* | *YDL006W* | *AFUA_5G13340* | Protein phosphatase 2C |
| *PTC2* | *YBL056W YER089C* | *AFUA_1G09280* | Protein phosphatase 2C |
| *PTC5* | *YOR090C* | *AFUA_1G06860* | Mitochondrial type 2C protein phosphatase involved in regulation of pyruvate dehydrogenase activity by dephosphorylating the ser-133 residue of the Pda1p subunit; acts in concert with kinases Pkp1p and Pkp2p and phosphatase Ptc6p |
| *PTC6* | *YCR079W* | *AFUA_1G15800* | Mitochondrial protein phosphatase of type 2C with similarity to mammalian PP1Ks; involved in mitophagy; null mutant is sensitive to rapamycin and has decreased phosphorylation of the Pda1 subunit of pyruvate dehydrogenase |
| *PTC7* | *YHR076W* | *AFUA_8G04580* | Ptc7p |
| *PTH2* | *YBL057C* | *AFUA_4G10430* | One of two mitochondrially-localized peptidyl-tRNA hydrolases; negatively regulates the ubiquitin-proteasome pathway via interactions with ubiquitin-like ubiquitin-associated proteins; dispensable for cell growth |
| *PTM1* | *YHL017W YKL039W* | *AFUA_5G12390* | Putative protein of unknown function; green fluorescent protein (GFP)-fusion protein co-localizes with clathrin-coated vesicles |
| *PTP1* | *YDL230W* | *AFUA_3G10970* | Phosphotyrosine-specific protein phosphatase that dephosphorylates a broad range of substrates in vivo, including Fpr3p; localized to the cytoplasm and the mitochondria |
| *PTR2* | *YKR093W* | *AFUA_7G01490* | Integral membrane peptide transporter, mediates transport of di- and tri-peptides; conserved protein that contains 12 transmembrane domains; PTR2 expression is regulated by the N-end rule pathway via repression by Cup9p |
| *PUB1* | *YNL016W* | *AFUA_1G12000* | Poly (A)+ RNA-binding protein, abundant mRNP-component protein that binds mRNA and is required for stability of many mRNAs; component of glucose deprivation induced stress granules, involved in P-body-dependent granule assembly |
| *PUF3* | *YLL013C* | *AFUA_6G04310* | Protein of the mitochondrial outer surface, links the Arp2/3 complex with the mitochore during anterograde mitochondrial movement; also binds to and promotes degradation of mRNAs for select nuclear-encoded mitochondrial proteins |
| *PUF6* | *YDR496C* | *AFUA_1G10440* | Puf6p |
| *PUN1* | *YLR414C YLR413W YKL187C* | *AFUA_2G10670 AFUA_1G09580 AFUA_6G07470* | Putative protein of unknown function; the authentic, non-tagged protein is detected in a phosphorylated state in highly purified mitochondria in high-throughput studies |
| *PUP1* | *YOR157C* | *AFUA_2G04910* | Beta 2 subunit of the 20S proteasome; endopeptidase with trypsin-like activity that cleaves after basic residues; synthesized as a proprotein before being proteolytically processed for assembly into 20S particle; human homolog is subunit Z |
| *PUP2* | *YGR253C* | *AFUA_2G11440* | Alpha 5 subunit of the 20S proteasome involved in ubiquitin-dependent catabolism; human homolog is subunit zeta |
| *PUS1* | *YPL212C* | *AFUA_2G08460* | TRNA pseudouridine synthase |
| *PUS4* | *YNL292W* | *AFUA_5G12310* | Pseudouridine synthase, catalyzes only the formation of pseudouridine-55 (Psi55), a highly conserved tRNA modification, in mitochondrial and cytoplasmic tRNAs; PUS4 overexpression leads to translational derepression of GCN4 (Gcd- phenotype) |
| *PUS7* | *YOR243C* | *AFUA_1G16730* | Pseudouridine synthase, catalyzes pseudouridylation at position 35 in U2 snRNA, position 50 in 5S rRNA, position 13 in cytoplasmic tRNAs, and position 35 in pre-tRNA(Tyr); conserved in archaea, vertebrates, and some bacteria |
| *PUT1* | *YLR142W* | *AFUA_3G02300 AFUA_6G08760* | Proline oxidase, nuclear-encoded mitochondrial protein involved in utilization of proline as sole nitrogen source; PUT1 transcription is induced by Put3p in the presence of proline and the absence of a preferred nitrogen source |
| *PUT2* | *YHR037W* | *AFUA_6G08750* | Delta-1-pyrroline-5-carboxylate dehydrogenase, nuclear-encoded mitochondrial protein involved in utilization of proline as sole nitrogen source; deficiency of the human homolog causes HPII, an autosomal recessive inborn error of metabolism |
| *PUT3* | *YKL015W* | *AFUA_2G05360 AFUA_3G00690 AFUA_6G14350* | C6 transcription factor |
| *PUT3* | *YKL015W* | *AFUA_1G16160 AFUA_6G12160* | C6 transcription factor |
| *PUT4* | *YOR348C* | *AFUA_7G01090* | Proline permease, required for high-affinity transport of proline; also transports the toxic proline analog azetidine-2-carboxylate (AzC); PUT4 transcription is repressed in ammonia-grown cells |
| *PWP1* | *YLR196W* | *AFUA_1G02630* | Protein with WD-40 repeats involved in rRNA processing; associates with trans-acting ribosome biogenesis factors; similar to beta-transducin superfamily |
| *PWP2* | *YCR057C* | *AFUA_5G03090* | Conserved 90S pre-ribosomal component essential for proper endonucleolytic cleavage of the 35 S rRNA precursor at A0, A1, and A2 sites; contains eight WD-repeats; PWP2 deletion leads to defects in cell cycle and bud morphogenesis |
| *PXA1* | *YPL147W* | *AFUA_1G04780 AFUA_1G12910* | Peroxisomal ABC transporter (PXA1) |
| *PXL1* | *YKR090W* | *AFUA_2G01000* | LIM domain-containing protein that localizes to sites of polarized growth, required for selection and/or maintenance of polarized growth sites, may modulate signaling by the GTPases Cdc42p and Rho1p; has similarity to metazoan paxillin |
| *PXR1* | *YGR280C* | *AFUA_7G03690* | G-patch RNA maturation protein (Gno1) |
| *PYC1* | *YBR218C YGL062W* | *AFUA_4G07710* | Pyruvate carboxylase |
| *PZF1* | *YPR186C* | *AFUA_1G05150* | C2H2 transcription factor (TFIIIA) |
| *QCR2* | *YPR191W* | *AFUA_5G04210* | Ubiquinol-cytochrome C reductase complex core protein 2 |
| *QCR6* | *YFR033C* | *AFUA_4G11390* | Ubiquinol-cytochrome c reductase complex 17 kd protein |
| *QCR7* | *YDR529C* | *AFUA_4G06790* | Ubiquinol-cytochrome c reductase complex 14 kDa protein |
| *QCR8* | *YJL166W* | *AFUA_1G13480* | Ubiquinol-cytochrome C reductase complex subunit UcrQ, putative |
| *QDR3* | *YBR043C* | *AFUA_4G13660* | Multidrug transporter of the major facilitator superfamily, required for resistance to quinidine, barban, cisplatin, and bleomycin |
| *QNS1* | *YHR074W* | *AFUA_5G03350* | Glutamine-dependent NAD(+) synthetase, essential for the formation of NAD(+) from nicotinic acid adenine dinucleotide |
| *RAD1* | *YPL022W* | *AFUA_6G02530* | DNA repair protein RAD1 |
| *RAD10* | *YML095C* | *AFUA_4G06260* | Mating-type switch/DNA repair protein Swi10/Rad10, putative |
| *RAD14* | *YMR201C* | *AFUA_5G01800* | Protein that recognizes and binds damaged DNA during nucleotide excision repair; subunit of Nucleotide Excision Repair Factor 1 (NEF1); contains zinc finger motif; homolog of human XPA protein |
| *RAD16* | *YBR114W* | *AFUA_7G03820* | Protein that recognizes and binds damaged DNA in an ATP-dependent manner during nucleotide excision repair; subunit of Nucleotide Excision Repair Factor 4 and the Elongin-Cullin-Socs ligase complex |
| *RAD18* | *YCR066W* | *AFUA_2G16700* | E3 ubiqutin ligase, forms heterodimer with Rad6p to monoubiquitinate PCNA-K164; heterodimer binds single-stranded DNA and has single-stranded DNA dependent ATPase activity; required for postreplication repair |
| *RAD2* | *YGR258C* | *AFUA_6G07500* | DNA excision repair protein Rad2 |
| *RAD23* | *YEL037C* | *AFUA_5G06040* | Protein with ubiquitin-like N terminus, subunit of Nuclear Excision Repair Factor 2 with Rad4p that recognizes and binds damaged DNA; enhances protein deglycosylation activity of Png1p; homolog of human HR23A and HR23B |
| *RAD26* | *YJR035W* | *AFUA_4G03840* | Protein involved in transcription-coupled nucleotide excision repair of UV-induced DNA lesions; recruitment to DNA lesions is dependent on an elongating RNA polymerase II; homolog of human CSB protein |
| *RAD27* | *YKL113C* | *AFUA_3G06060* | 5' to 3' exonuclease, 5' flap endonuclease, required for Okazaki fragment processing and maturation as well as for long-patch base-excision repair; member of the S. pombe RAD2/FEN1 family |
| *RAD3* | *YER171W* | *AFUA_1G13910* | 5' to 3' DNA helicase, involved in nucleotide excision repair and transcription; subunit of RNA polymerase II transcription initiation factor TFIIH; subunit of Nucleotide Excision Repair Factor 3 (NEF3); homolog of human XPD protein |
| *RAD4* | *YER162C* | *AFUA_2G04860 AFUA_2G11660* | Protein that recognizes and binds damaged DNA during nucleotide excision repair; subunit of Nuclear Excision Repair Factor 2 (NEF2); homolog of human XPC protein |
| *RAD5* | *YLR032W* | *AFUA_5G12600* | DNA helicase proposed to promote replication fork regression during postreplication repair by template switching; RING finger containing ubiquitin ligase; stimulates the synthesis of free and PCNA-bound polyubiquitin chains by Ubc13p-Mms2p |
| *RAD50* | *YNL250W* | *AFUA_4G12680* | DNA repair protein Rad50 |
| *RAD51* | *YER095W* | *AFUA_1G10410* | DNA repair protein RAD51 |
| *RAD52* | *YML032C* | *AFUA_4G06970* | Protein that stimulates strand exchange by facilitating Rad51p binding to single-stranded DNA; anneals complementary single-stranded DNA; involved in the repair of double-strand breaks in DNA during vegetative growth and meiosis |
| *RAD53* | *YPL153C* | *AFUA_2G14920* | Protein kinase, required for cell-cycle arrest in response to DNA damage; activated by trans autophosphorylation when interacting with hyperphosphorylated Rad9p; also interacts with ARS1 and plays a role in initiation of DNA replication |
| *RAD54* | *YGL163C* | *AFUA_6G12910* | DNA-dependent ATPase, stimulates strand exchange by modifying the topology of double-stranded DNA; involved in the recombinational repair of double-strand breaks in DNA during vegetative growth and meiosis; member of the SWI/SNF family |
| *RAD57* | *YDR004W* | *AFUA_1G12520* | Protein that stimulates strand exchange by stabilizing the binding of Rad51p to single-stranded DNA; involved in the recombinational repair of double-strand breaks in DNA during vegetative growth and meiosis; forms heterodimer with Rad55p |
| *RAD6* | *YGL058W* | *AFUA_6G14210* | Ubiquitin conjugating enzyme (UbcB) |
| *RAD7* | *YJR052W* | *AFUA_7G03830* | Protein that recognizes and binds damaged DNA in an ATP-dependent manner during nucleotide excision repair; subunit of Nucleotide Excision Repair Factor 4 and the Elongin-Cullin-Socs ligase complex |
| *RAD9* | *YDR217C* | *AFUA_5G04020* | DNA damage-dependent checkpoint protein, required for cell-cycle arrest in G1/S, intra-S, and G2/M; transmits checkpoint signal by activating Rad53p and Chk1p; hyperphosphorylated by Mec1p and Tel1p; potential Cdc28p substrate |
| *RAI1* | *YGL246C* | *AFUA_6G05060* | Nuclear protein that binds to and stabilizes the exoribonuclease Rat1p, required for pre-rRNA processing |
| *RAM1* | *YDL090C* | *AFUA_4G10330* | Beta subunit of the CAAX farnesyltransferase that prenylates the a-factor mating pheromone and Ras proteins; required for the membrane localization of Ras proteins and a-factor; homolog of the mammalian FTase beta subunit |
| *RAM2* | *YKL019W* | *AFUA_4G07800* | Alpha subunit of both the farnesyltransferase and type I geranylgeranyltransferase that catalyze prenylation of proteins containing a CAAX consensus motif; essential protein required for membrane localization of Ras proteins and a-factor |
| *RAP1* | *YNL216W* | *AFUA_6G07560* | DNA-binding protein involved in either activation or repression of transcription, depending on binding site context; also binds telomere sequences and plays a role in telomeric position effect and telomere structure |
| *RAS1* | *YOR101W YNL098C* | *AFUA_5G11230* | GTP-binding protein that regulates the nitrogen starvation response, sporulation, and filamentous growth; farnesylation and palmitoylation required for activity and localization to plasma membrane; homolog of mammalian Ras proto-oncogenes |
| *RAT1* | *YOR048C* | *AFUA_1G13730* | Nuclear 5' to 3' single-stranded RNA exonuclease, involved in RNA metabolism, including rRNA and snRNA processing as well as poly dependent and independent mRNA transcription termination |
| *RAV1* | *YJR033C* | *AFUA_1G13690* | WD repeat protein |
| *RAX1* | *YOR301W* | *AFUA_4G12640* | Protein involved in bud site selection during bipolar budding; localization requires Rax2p; has similarity to members of the insulin-related peptide superfamily |
| *RAX2* | *YLR084C* | *AFUA_7G05340* | N-glycosylated protein involved in the maintenance of bud site selection during bipolar budding; localization requires Rax1p; RAX2 mRNA stability is regulated by Mpt5p |
| *RBA50* | *YDR527W* | *AFUA_3G05940* | Protein involved in transcription; interacts with RNA polymerase II subunits Rpb2p, Rpb3, and Rpb11p; has similarity to human RPAP1 |
| *RBD2* | *YPL246C* | *AFUA_6G12750* | Possible rhomboid protease, has similarity to eukaryotic rhomboid proteases including Pcp1p |
| *RBG1* | *YAL036C* | *AFUA_1G05560* | Member of the DRG family of GTP-binding proteins; associates with translating ribosomes; interacts with Tma46p, Ygr250cp, Gir2p and Yap1p via two-hybrid |
| *RBG2* | *YGR173W* | *AFUA_5G06770* | Protein with similarity to mammalian developmentally regulated GTP-binding protein |
| *RBL2* | *YOR265W* | *AFUA_2G08190* | Protein involved in microtubule morphogenesis, required for protection from excess free beta-tubulin; proposed to be involved the folding of beta-tubulin; similar to mouse beta-tubulin cofactor A |
| *RBS1* | *YDL189W* | *AFUA_1G09400* | Protein of unknown function, identified as a high copy suppressor of psk1 psk2 mutations that confer temperature-sensitivity for galactose utilization; proposed to bind single-stranded nucleic acids via its R3H domain |
| *RCO1* | *YMR075W* | *AFUA_2G16810* | Essential subunit of the histone deacetylase Rpd3S complex; interacts with Eaf3p |
| *RCY1* | *YJL204C* | *AFUA_1G02050* | F-box protein involved in recycling plasma membrane proteins internalized by endocytosis; localized to sites of polarized growth |
| *RDI1* | *YDL135C* | *AFUA_5G11380* | Rho-gdp dissociation inhibitor |
| *RDR1* | *YOR380W* | *AFUA_8G07180* | C6 transcription factor |
| *RDS2* | *YPL133C* | *AFUA_4G01392* | C6 transcription factor |
| *RDS2* | *YPL133C* | *AFUA_2G12330* | Zn cluster transcription factor Rds2 |
| *REC8* | *YPR007C* | *AFUA_6G02900* | Meiosis-specific component of sister chromatid cohesion complex; maintains cohesion between sister chromatids during meiosis I; maintains cohesion between centromeres of sister chromatids until meiosis II; homolog of S. pombe Rec8p |
| *REE1* | *YJL217W* | *AFUA_2G05720 AFUA_2G16990* | Cytoplasmic protein involved in the regulation of enolase and the presence of galactose (via Gal4p); mRNA expression is also regulated by the cell cycle |
| *REG1* | *YDR028C* | *AFUA_8G02720* | Protein phosphatase type 1 complex subunit Hex2/Reg1, putative |
| *REH1* | *YBR267W YLR387C* | *AFUA_2G04440* | Cytoplasmic pre-60S factor; required for the correct recycling of shuttling factors Alb1, Arx1 and Tif6 at the end of the ribosomal large subunit biogenesis; involved in bud growth in the mitotic signaling network |
| *RER1* | *YCL001W* | *AFUA_2G10930* | Protein involved in retention of membrane proteins, including Sec12p, in the ER; localized to Golgi; functions as a retrieval receptor in returning membrane proteins to the ER |
| *RER2* | *YBR002C* | *AFUA_4G11350* | Cis-prenyltransferase involved in dolichol synthesis; participates in endoplasmic reticulum protein sorting |
| *RET1* | *YOR207C* | *AFUA_1G02460* | DNA-directed RNA polymerase III, beta subunit |
| *REV3* | *YPL167C* | *AFUA_3G06780* | Catalytic subunit of DNA polymerase zeta, involved in translesion synthesis during post-replication repair; required for mutagenesis induced by DNA damage; involved in double-strand break repair |
| *REX3* | *YLR107W* | *AFUA_3G12310* | RNA exonuclease; required for maturation of the RNA component of RNase MRP; functions redundantly with Rnh70p and Rex2p in processing of U5 snRNA and RNase P RNA; member of RNase D family of exonucleases |
| *RFA1* | *YAR007C* | *AFUA_2G06320* | Replication protein A 70 kDa DNA-binding subunit |
| *RFA2* | *YNL312W* | *AFUA_6G11130* | Possible replication factor-a protein |
| *RFC1* | *YOR217W* | *AFUA_2G12220* | DNA replication factor C subunit Rfc1 |
| *RFC2* | *YJR068W* | *AFUA_3G08300* | DNA replication factor C subunit Rfc2 |
| *RFC3* | *YNL290W* | *AFUA_5G01870* | DNA replication factor C subunit Rfc3 |
| *RFC4* | *YOL094C* | *AFUA_6G05040* | DNA replication factor C subunit Rfc4 |
| *RFC5* | *YBR087W* | *AFUA_2G12250* | DNA replication factor C subunit Rfc5 |
| *RFT1* | *YBL020W* | *AFUA_2G06290* | Essential integral membrane protein that is required for translocation of Man5GlcNac2-PP-Dol from the cytoplasmic side to the lumenal side of the ER membrane but is not the flippase; mutation is suppressed by expression of human p53 protein |
| *RFT1* | *YBL020W* | *AFUA_2G06300* | Essential integral membrane protein that is required for translocation of Man5GlcNac2-PP-Dol from the cytoplasmic side to the lumenal side of the ER membrane but is not the flippase; mutation is suppressed by expression of human p53 protein |
| *RFU1* | *YLR073C* | *AFUA_3G08730* | Protein that inhibits Doa4p deubiquitinating activity; contributes to ubiquitin homeostasis by regulating the conversion of free ubiquitin chains to ubiquitin monomers by Doa4p; GFP-fusion protein localizes to endosomes |
| *RFX1* | *YLR176C* | *AFUA_5G06120* | Major transcriptional repressor of DNA-damage-regulated genes, recruits repressors Tup1p and Cyc8p to their promoters; involved in DNA damage and replication checkpoint pathway; similar to a family of mammalian DNA binding RFX1-4 proteins |
| *RGD2* | *YFL047W* | *AFUA_7G04300* | GTPase-activating protein for Cdc42p and Rho5p |
| *RGR1* | *YLR071C* | *AFUA_2G06180* | RNA polymerase II holoenzyme/mediator complex component Rgr1 |
| *RGS2* | *YOR107W* | *AFUA_5G00900 AFUA_6G06860* | Negative regulator of glucose-induced cAMP signaling; directly activates the GTPase activity of the heterotrimeric G protein alpha subunit Gpa2p |
| *RGT1* | *YKL038W* | *AFUA_6G07800* | Glucose-responsive transcription factor that regulates expression of several glucose transporter genes in response to glucose; binds to promoters and acts both as a transcriptional activator and repressor |
| *RHO1* | *YPR165W* | *AFUA_6G06900* | GTP-binding protein of the rho subfamily of Ras-like proteins, involved in establishment of cell polarity; regulates protein kinase C and the cell wall synthesizing enzyme 1,3-beta-glucan synthase (Fks1p and Gsc2p) |
| *RHO2* | *YNL090W* | *AFUA_3G10340* | Non-essential small GTPase of the Rho/Rac subfamily of Ras-like proteins, involved in the establishment of cell polarity and in microtubule assembly |
| *RIM1* | *YCR028C-A* | *AFUA_5G07890* | SsDNA binding protein |
| *RIM101* | *YHL027W* | *AFUA_3G11970* | C2H2 transcription factor PacC |
| *RIM2* | *YBR192W* | *AFUA_3G06950* | Mitochondrial pyrimidine nucleotide transporter; imports pyrimidine nucleoside triphosphates and exports pyrimidine nucleoside monophosphates; member of the mitochondrial carrier family |
| *RIM20* | *YOR275C* | *AFUA_2G11760 AFUA_4G06400* | Protein involved in proteolytic activation of Rim101p in response to alkaline pH; PalA/AIP1/Alix family member; interaction with the ESCRT-III subunit Snf7p suggests a relationship between pH response and multivesicular body formation |
| *RIM21* | *YNL294C* | *AFUA_5G13270* | Component of the RIM101 pathway, has a role in cell wall construction and alkaline pH response; has similarity to A. nidulans PalH |
| *RIM4* | *YHL024W* | *AFUA_3G06230* | Putative RNA-binding protein required for the expression of early and middle sporulation genes |
| *RIM8* | *YGL045W* | *AFUA_4G09650* | Protein involved in proteolytic activation of Rim101p in response to alkaline pH; interacts with ESCRT-1 subunits Stp22p and Vps28p; essential for anaerobic growth; member of the arrestin-related trafficking adaptor family |
| *RIO1* | *YOR119C* | *AFUA_2G14090* | Essential serine kinase involved in cell cycle progression and processing of the 20S pre-rRNA into mature 18S rRNA |
| *RIO2* | *YNL207W* | *AFUA_5G11730* | Essential serine kinase involved in the processing of the 20S pre-rRNA into mature 18S rRNA; has similarity to Rio1p |
| *RIP1* | *YEL024W* | *AFUA_5G10610* | Ubiquinol-cytochrome c reductase iron-sulfur subunit precursor |
| *RIX7* | *YLL034C* | *AFUA_1G09210* | Putative ATPase of the AAA family, required for export of pre-ribosomal large subunits from the nucleus; distributed between the nucleolus, nucleoplasm, and nuclear periphery depending on growth conditions |
| *RKI1* | *YOR095C* | *AFUA_6G10610* | Ribose-5-phosphate ketol-isomerase, catalyzes the interconversion of ribose 5-phosphate and ribulose 5-phosphate in the pentose phosphate pathway; participates in pyridoxine biosynthesis |
| *RKM1* | *YHL039W YPL208W* | *AFUA_4G11040* | Putative protein of unknown function; contains a SET-domain; predicted to be involved in ribosome biogenesis; green fluorescent protein (GFP)-fusion protein localizes to the cytoplasm |
| *RKM4* | *YDR257C* | *AFUA_6G04520* | Ribosomal lysine methyltransferase specific for monomethylation of Rpl42ap and Rpl42bp (lysine 55); nuclear SET-domain containing protein |
| *RKR1* | *YMR247C* | *AFUA_1G13920* | Nuclear RING domain protein with functional connections to chromatin modification; may interact with ribosomes, based on co-purification experiments; YMR247C is not an essential gene |
| *RLF2* | *YPR018W* | *AFUA_5G03720* | Largest subunit of the Chromatin Assembly Complex with Cac2p and Msi1p that assembles newly synthesized histones onto recently replicated DNA; involved in the maintenance of transcriptionally silent chromatin |
| *RLM1* | *YBR182C YPL089C* | *AFUA_3G08520* | MADS-box transcription factor, component of the protein kinase C-mediated MAP kinase pathway involved in the maintenance of cell integrity; phosphorylated and activated by the MAP-kinase Slt2p |
| *RMD1* | *YDL001W* | *AFUA_2G12110* | Cytoplasmic protein required for sporulation |
| *RMD5* | *YDR255C* | *AFUA_1G04490* | Conserved protein that has an E3-like ubiquitin ligase activity necessary for polyubiquitination and degradation of the gluconeogenic enzyme fructose-1,6-bisphosphatase; also required for sporulation; has a degenerate RING finger domain |
| *RMD8* | *YFR048W* | *AFUA_4G10390* | Cytosolic protein required for sporulation |
| *RME1* | *YGR044C* | *AFUA_8G05010* | C2H2 finger domain protein |
| *RML2* | *YEL050C* | *AFUA_4G12170* | Mitochondrial ribosomal protein of the large subunit, has similarity to E. coli L2 ribosomal protein; fat21 mutant allele causes inability to utilize oleate and may interfere with activity of the Adr1p transcription factor |
| *RMP1* | *YLR145W* | *AFUA_6G05270* | Rmp1p |
| *RMR1* | *YGL250W* | *AFUA_1G15870* | Protein required for meiotic recombination and gene conversion; null mutant displays reduced PIS1 expression and growth defects on non-fermentable carbon sources and minimal media; GFP-fusion protein localizes to both cytoplasm and nucleus |
| *RMT2* | *YDR465C* | *AFUA_2G09080* | Arginine methyltransferase; ribosomal protein L12 is a substrate |
| *RNA1* | *YMR235C* | *AFUA_3G07680* | GTPase activating protein for Gsp1p, involved in nuclear transport |
| *RNA14* | *YMR061W* | *AFUA_3G11020* | Cleavage and polyadenylation factor I component involved in cleavage and polyadenylation of mRNA 3' ends; bridges interaction between Rna15p and Hrp1p in the CF I complex |
| *RNA15* | *YGL044C* | *AFUA_2G09100* | Cleavage and polyadenylation factor I component involved in cleavage and polyadenylation of mRNA 3' ends; interacts with the A-rich polyadenylation signal in complex with Rna14p and Hrp1p |
| *RNR2* | *YJL026W* | *AFUA_5G12350* | Ribonucleotide-diphosphate reductase (RNR), small subunit; the RNR complex catalyzes the rate-limiting step in dNTP synthesis and is regulated by DNA replication and DNA damage checkpoint pathways via localization of the small subunits |
| *RNT1* | *YMR239C* | *AFUA_5G04440* | RNAase III; involved in rDNA transcription and rRNA processing; also cleaves a stem-loop structure at the 3' end of U2 snRNA to ensure formation of the correct U2 3' end; involved in polyadenylation-independent transcription termination |
| *ROD1* | *YFR022W YOR018W* | *AFUA_6G07900* | Protein that binds the ubiquitin ligase Rsp5p via its 2 PY motifs; has similarity to Rod1p; mutation suppresses the temperature sensitivity of an mck1 rim11 double mutant; proposed to regulate the endocytosis of plasma membrane proteins |
| *ROG1* | *YDR444W YGL144C YDL109C* | *AFUA_3G04240* | Putative lipase; involved in lipid metabolism; YDL109C is not an essential gene |
| *ROT2* | *YBR229C* | *AFUA_5G03500* | Glucosidase II catalytic subunit required for normal cell wall synthesis; mutations in rot2 suppress tor2 mutations, and are synthetically lethal with rot1 mutations |
| *ROX1* | *YPR065W* | *AFUA_4G10820* | Heme-dependent repressor of hypoxic genes; contains an HMG domain that is responsible for DNA bending activity |
| *ROX3* | *YBL093C* | *AFUA_4G12550* | Rox3p |
| *RPA12* | *YJR063W* | *AFUA_2G05480* | DNA-directed RNA polymerase I 13.1 kDa polypeptide, putative |
| *RPA135* | *YPR010C* | *AFUA_6G08300* | DNA-directed RNA polymerase I subunit beta (Rpa2), putative |
| *RPA190* | *YOR341W* | *AFUA_1G13900* | DNA-directed RNA polymerase I subunit (Rpa190), putative |
| *RPA43* | *YOR340C* | *AFUA_7G02080* | RNA polymerase I subunit Rpa43 |
| *RPB10* | *YOR210W* | *AFUA_7G02620* | DNA-directed RNA polymerases N/8 kDa subunit superfamily |
| *RPB11* | *YOL005C* | *AFUA_7G03860* | DNA-directed RNA polymerase II subunit RPB11a |
| *RPB2* | *YOR151C* | *AFUA_7G01920* | DNA-dependent RNA polymerase II RPB140 |
| *RPB3* | *YIL021W* | *AFUA_2G13090* | RNA polymerase II subunit 3 |
| *RPB4* | *YJL140W* | *AFUA_3G08040* | Polymerase II polypeptide D |
| *RPB5* | *YBR154C* | *AFUA_1G14110* | RNA polymerase subunit |
| *RPB7* | *YDR404C* | *AFUA_8G05300* | RNA polymerase II subunit 7 |
| *RPB8* | *YOR224C* | *AFUA_5G11120* | DNA-directed RNA polymerases i, ii, and iii 145 kDa polypeptide |
| *RPB9* | *YGL070C* | *AFUA_2G08540* | DNA directed RNA polymerase II 15 kDa subunit |
| *RPC10* | *YHR143W-A* | *AFUA_4G11970* | Metallothionein-I gene transcription activator |
| *RPC19* | *YNL113W* | *AFUA_6G04610* | DNA-directed RNA polymerase I and III 14 KDA polypeptide |
| *RPC25* | *YKL144C* | *AFUA_8G04350* | DNA-directed RNA polymerase III subunit 22.9 kDa, putative |
| *RPC34* | *YNR003C* | *AFUA_7G01850* | DNA-directed RNA polymerase III subunit Rpc34 |
| *RPC37* | *YKR025W* | *AFUA_2G15600* | C37 |
| *RPC40* | *YPR110C* | *AFUA_2G13720* | DNA-directed RNA polymerase I and III subunit Rpc40, putative |
| *RPC53* | *YDL150W* | *AFUA_2G16170* | DNA-directed RNA polymerase III RPC4 |
| *RPC82* | *YPR190C* | *AFUA_5G04200* | DNA directed RNA polymerase III subunit Rpc82 |
| *RPD3* | *YNL330C* | *AFUA_2G03390* | Histone deacetylase; regulates transcription and silencing; plays a role in regulating Ty1 transposition |
| *RPE1* | *YJL121C* | *AFUA_2G15190* | D-ribulose-5-phosphate 3-epimerase, catalyzes a reaction in the non-oxidative part of the pentose-phosphate pathway; mutants are sensitive to oxidative stress |
| *RPF1* | *YHR088W* | *AFUA_5G05930* | Nucleolar protein involved in the assembly and export of the large ribosomal subunit; constituent of 66S pre-ribosomal particles; contains a sigma(70)-like motif, which is thought to bind RNA |
| *RPF2* | *YKR081C* | *AFUA_8G04790* | Essential protein involved in the processing of pre-rRNA and the assembly of the 60S ribosomal subunit; interacts with ribosomal protein L11; localizes predominantly to the nucleolus; constituent of 66S pre-ribosomal particles |
| *RPL10* | *YLR075W* | *AFUA_2G09210* | 60S ribosomal protein L10 |
| *RPL11A* | *YGR085C YPR102C* | *AFUA_4G07730* | 60S ribosomal protein L11 |
| *RPL12A* | *YEL054C YDR418W* | *AFUA_1G03390* | 60S ribosomal protein L12 |
| *RPL13A* | *YMR142C YDL082W* | *AFUA_4G04460* | 60S ribosomal protein L13 |
| *RPL14A* | *YHL001W YKL006W* | *AFUA_6G03830* | Ribosomal protein L14 |
| *RPL15A* | *YLR029C YMR121C* | *AFUA_1G04660* | 60S ribosomal protein L15 |
| *RPL16A* | *YIL133C YNL069C* | *AFUA_1G05990* | Ribosomal protein L16a |
| *RPL17A* | *YJL177W YKL180W* | *AFUA_1G14410* | 60S ribosomal protein L17 |
| *RPL18A* | *YOL120C YNL301C* | *AFUA_2G07380* | 60S ribosomal protein L18 |
| *RPL19A* | *YBL027W YBR084C-A* | *AFUA_2G07970* | 60S ribosomal protein L19 |
| *RPL1A* | *YGL135W YPL220W* | *AFUA_1G11710* | 60S ribosomal protein L1 |
| *RPL20A* | *YMR242C YOR312C* | *AFUA_1G04530* | Ribosomal L18ae protein family |
| *RPL21A* | *YBR191W YPL079W* | *AFUA_3G06960* | 60S ribosomal protein L21 |
| *RPL22A* | *YFL034C-A YLR061W* | *AFUA_3G12300* | 60S ribosomal protein L22 |
| *RPL23A* | *YBL087C YER117W* | *AFUA_2G03380* | Alkaline serine protease |
| *RPL24A* | *YGL031C YGR148C* | *AFUA_6G02440* | 60S ribosomal protein L24a |
| *RPL25* | *YOL127W* | *AFUA_5G05630* | 60S ribosomal protein L23 |
| *RPL26A* | *YGR034W YLR344W* | *AFUA_6G11260* | Ribosomal protein L26 |
| *RPL27A* | *YHR010W YDR471W* | *AFUA_1G06340* | 60S ribosomal protein L27e |
| *RPL28* | *YGL103W* | *AFUA_3G05600* | 60S ribosomal protein L27a |
| *RPL29* | *YFR032C-A* | *AFUA_1G03110* | 60S ribosomal protein L29 |
| *RPL2A* | *YFR031C-A YIL018W* | *AFUA_5G06360* | 60S ribosomal protein L8 |
| *RPL3* | *YOR063W* | *AFUA_2G11850* | 60S ribosomal protein L3 |
| *RPL30* | *YGL030W* | *AFUA_2G09200* | 60S ribosomal protein L30 |
| *RPL31A* | *YLR406C YDL075W* | *AFUA_6G13250* | 60S ribosomal protein L31e |
| *RPL32* | *YBL092W* | *AFUA_2G16370* | 60S ribosomal protein L32 |
| *RPL33A* | *YOR234C YPL143W* | *AFUA_3G08460* | 60S ribosomal protein L35Ae |
| *RPL34A* | *YER056C-A YIL052C* | *AFUA_2G03040* | Ribosomal protein L34 protein |
| *RPL35A* | *YDL191W YDL136W* | *AFUA_1G10510* | 60S ribosomal protein L35 |
| *RPL36A* | *YMR194W YPL249C-A* | *AFUA_4G07435* | 60S ribosomal protein L36 |
| *RPL37A* | *YLR185W YDR500C* | *AFUA_3G06760* | 60S ribosomal protein L37 |
| *RPL38* | *YLR325C* | *AFUA_4G07845* | 60S ribosomal protein L38 |
| *RPL40A* | *YIL148W YKR094C* | *AFUA_1G04040* | Ubiquitin UbiA |
| *RPL42A* | *YHR141C YNL162W* | *AFUA_2G08130* | 60S ribosomal protein L44 |
| *RPL43A* | *YPR043W YJR094W-A* | *AFUA_2G16880* | 60S ribosomal protein L37a |
| *RPL4A* | *YBR031W YDR012W* | *AFUA_5G03020* | 60S ribosomal protein L4 |
| *RPL5* | *YPL131W* | *AFUA_1G12890* | 60S ribosomal protein L5 |
| *RPL6A* | *YLR448W YML073C* | *AFUA_1G11130* | 60S ribosomal protein L6 |
| *RPL7A* | *YGL076C YPL198W* | *AFUA_4G03880* | 60S ribosomal protein L7 |
| *RPL8A* | *YHL033C YLL045C* | *AFUA_6G12990* | Cytosolic large ribosomal subunit protein L7A |
| *RPL9A* | *YGL147C YNL067W* | *AFUA_1G09100* | 60S ribosomal protein L9 |
| *RPN12* | *YFR052W* | *AFUA_3G08940* | Proteasome regulatory particle subunit (RpnL) |
| *RPN4* | *YDL020C* | *AFUA_1G13750* | C2H2 transcription factor (Rpn4) |
| *RPN9* | *YDR427W* | *AFUA_6G12770* | Non-ATPase regulatory subunit of the 26S proteasome, has similarity to putative proteasomal subunits in other species; null mutant is temperature sensitive and exhibits cell cycle and proteasome assembly defects |
| *RPO21* | *YDL140C* | *AFUA_1G14680* | DNA-dependent RNA polymerase II largest subunit, putative |
| *RPO26* | *YPR187W* | *AFUA_1G05160* | DNA-directed RNA polymerase I, II, and III subunit Rpb6 |
| *RPO31* | *YOR116C* | *AFUA_3G14370* | DNA-directed RNA polymerase III largest subunit, putative |
| *RPO41* | *YFL036W* | *AFUA_2G15610* | Mitochondrial RNA polymerase; single subunit enzyme similar to those of T3 and T7 bacteriophages; requires a specificity subunit encoded by MTF1 for promoter recognition |
| *RPP0* | *YLR340W* | *AFUA_1G05080* | 60S ribosomal protein P0 |
| *RPP1* | *YHR062C* | *AFUA_8G04820* | Ribonuclease P complex subunit Pop2 |
| *RPP1A* | *YDL130W YDL081C* | *AFUA_1G06830* | Ribosomal stalk protein P1 alpha, involved in the interaction between translational elongation factors and the ribosome; accumulation of P1 in the cytoplasm is regulated by phosphorylation and interaction with the P2 stalk component |
| *RPP2B* | *YDR382W* | *AFUA_2G10100* | 60S acidic ribosomal protein P2/allergen Asp F 8 |
| *RPS0A* | *YGR214W YLR048W* | *AFUA_3G13320* | 40S ribosomal protein S0 |
| *RPS10A* | *YMR230W YOR293W* | *AFUA_6G12660* | 40S ribosomal protein S10b |
| *RPS11A* | *YBR048W YDR025W* | *AFUA_2G04130* | 40S ribosomal protein S11 |
| *RPS12* | *YOR369C* | *AFUA_1G05500* | 40S ribosomal protein S12 |
| *RPS13* | *YDR064W* | *AFUA_7G05290* | 40S ribosomal protein S13 |
| *RPS14A* | *YCR031C YJL191W* | *AFUA_2G10440* | 40S ribosomal protein S11 |
| *RPS15* | *YOL040C* | *AFUA_2G10090* | 40S ribosomal protein S15 |
| *RPS16A* | *YMR143W YDL083C* | *AFUA_2G10500* | 40S ribosomal protein Rps16 |
| *RPS17A* | *YML024W YDR447C* | *AFUA_2G10300* | 40S ribosomal protein S17 |
| *RPS18A* | *YML026C YDR450W* | *AFUA_6G13550* | Ribosomal protein S13p/S18e |
| *RPS19A* | *YOL121C YNL302C* | *AFUA_1G05340* | 40S ribosomal protein S19 |
| *RPS1A* | *YLR441C YML063W* | *AFUA_5G05450* | 40S ribosomal protein S3Ae |
| *RPS2* | *YGL123W* | *AFUA_7G01460* | Ribosomal protein S5 |
| *RPS20* | *YHL015W* | *AFUA_2G02150* | 40S ribosomal protein S10a |
| *RPS21A* | *YJL136C YKR057W* | *AFUA_2G03590* | 40S ribosomal protein S21 |
| *RPS22A* | *YLR367W YJL190C* | *AFUA_1G15730* | 40S ribosomal protein S22 |
| *RPS23A* | *YGR118W YPR132W* | *AFUA_1G09440* | Ribosomal protein S23 (S12) |
| *RPS24A* | *YER074W YIL069C* | *AFUA_7G02140* | 37S ribosomal protein S24 |
| *RPS25A* | *YGR027C YLR333C* | *AFUA_1G16523* | 40S ribosomal protein S25 |
| *RPS26A* | *YER131W YGL189C* | *AFUA_1G06770* | 40S ribosomal protein S26 |
| *RPS27A* | *YHR021C YKL156W* | *AFUA_3G06640* | 40S ribosomal protein S27 |
| *RPS28A* | *YLR264W YOR167C* | *AFUA_7G04490* | Ribosomal protein S28e |
| *RPS29A* | *YLR388W YDL061C* | *AFUA_6G12720* | 40S ribosomal protein S29 |
| *RPS3* | *YNL178W* | *AFUA_1G05630* | 40S ribosomal protein S3 |
| *RPS30A* | *YLR287C-A YOR182C* | *AFUA_6G02450* | Ribosomal S30/ubiquitin fusion |
| *RPS31* | *YLR167W* | *AFUA_3G11260* | Ubiquitin (UbiC) |
| *RPS4A* | *YHR203C YJR145C* | *AFUA_3G06840* | 40S ribosomal protein S4 |
| *RPS5* | *YJR123W* | *AFUA_1G15020* | 40S ribosomal protein S5 |
| *RPS6A* | *YBR181C YPL090C* | *AFUA_4G10800* | Protein component of the small ribosomal subunit; identical to Rps6Ap and has similarity to rat S6 ribosomal protein |
| *RPS7A* | *YOR096W YNL096C* | *AFUA_3G10730* | 40S ribosomal protein S7e |
| *RPS8A* | *YBL072C YER102W* | *AFUA_1G04320* | 40S ribosomal protein S8e |
| *RPS9A* | *YBR189W YPL081W* | *AFUA_3G06970* | 40S ribosomal protein S9 |
| *RPT4* | *YOR259C* | *AFUA_6G06780* | One of six ATPases of the 19S regulatory particle of the 26S proteasome involved in the degradation of ubiquitinated substrates; required for spindle pole body duplication; localized mainly to the nucleus throughout the cell cycle |
| *RRB1* | *YMR131C* | *AFUA_6G10320* | Essential nuclear protein involved in early steps of ribosome biogenesis; physically interacts with the ribosomal protein Rpl3p |
| *RRD2* | *YPL152W* | *AFUA_1G13420* | Activator of the phosphotyrosyl phosphatase activity of PP2A,peptidyl-prolyl cis/trans-isomerase; regulates G1 phase progression, the osmoresponse, microtubule dynamics; subunit of the Tap42p-Pph21p-Rrd2p complex |
| *RRF1* | *YHR038W* | *AFUA_8G03940* | Mitochondrial ribosome recycling factor, essential for mitochondrial protein synthesis and for the maintenance of the respiratory function of mitochondria |
| *RRG7* | *YOR305W* | *AFUA_2G13340* | Protein of unknown function; green fluorescent protein phenylarsenoxide (GSAO); YOR305W is not an essential gene |
| *RRG9* | *YNL213C* | *AFUA_2G12370* | Protein of unknown function; null mutant lacks mitochondrial DNA and cannot grow on glycerol; the authentic, non-tagged protein is detected in highly purified mitochondria in high-throughput studies |
| *RRI1* | *YDL216C* | *AFUA_2G16250* | Catalytic subunit of the COP9 signalosome complex that acts as an isopeptidase in cleaving the ubiquitin-like protein Nedd8 from SCF ubiquitin ligases; metalloendopeptidase involved in the adaptation to pheromone signaling |
| *RRN3* | *YKL125W* | *AFUA_1G02590* | Protein required for transcription of rDNA by RNA polymerase I; transcription factor independent of DNA template; involved in recruitment of RNA polymerase I to rDNA |
| *RRN6* | *YBL014C* | *AFUA_1G08870* | Component of the core factor rDNA transcription factor complex; CF is required for transcription of 35S rRNA genes by RNA polymerase I and is composed of Rrn6p, Rrn7p, and Rrn11p |
| *RRN7* | *YJL025W* | *AFUA_1G04062* | Component of the core factor rDNA transcription factor complex; CF is required for transcription of 35S rRNA genes by RNA polymerase I and is composed of Rrn6p, Rrn7p, and Rrn11p |
| *RRP12* | *YPL012W* | *AFUA_2G11810* | Protein required for export of the ribosomal subunits; associates with the RNA components of the pre-ribosomes; contains HEAT-repeats |
| *RRP14* | *YKL082C* | *AFUA_1G06600* | Essential protein, constituent of 66S pre-ribosomal particles; interacts with proteins involved in ribosomal biogenesis and cell polarity; member of the SURF-6 family |
| *RRP15* | *YPR143W* | *AFUA_5G03870* | Nucleolar protein, constituent of pre-60S ribosomal particles; required for proper processing of the 27S pre-rRNA at the A3 and B1 sites to yield mature 5.8S and 25S rRNAs |
| *RRP3* | *YHR065C* | *AFUA_1G06220* | Protein involved in rRNA processing; required for maturation of the 35S primary transcript of pre-rRNA and for cleavage leading to mature 18S rRNA; homologous to eIF-4a, which is a DEAD box RNA-dependent ATPase with helicase activity |
| *RRP36* | *YOR287C* | *AFUA_6G04590* | Component of 90S preribosomes; involved in early cleavages of the 35S pre-rRNA and in production of the 40S ribosomal subunit |
| *RRP4* | *YHR069C* | *AFUA_3G05860* | Exosome non-catalytic core component; involved in 3'-5' RNA processing and degradation in both the nucleus and the cytoplasm; predicted to contain RNA binding domains; has similarity to human hRrp4p (EXOSC2) |
| *RRP40* | *YOL142W* | *AFUA_2G02340* | Exosome non-catalytic core component; involved in 3'-5' RNA processing and degradation in both the nucleus and the cytoplasm; predicted to contain both S1 and KH RNA binding domains; has similarity to human hRrp40p (EXOSC3) |
| *RRP45* | *YDR280W* | *AFUA_2G08860* | Exosome non-catalytic core component; involved in 3'-5' RNA processing and degradation in both the nucleus and the cytoplasm; has similarity to E. coli RNase PH and to human hRrp45p (PM/SCL-75, EXOSC9) |
| *RRP5* | *YMR229C* | *AFUA_2G16040* | RNA binding protein with preference for single stranded tracts of U's involved in synthesis of both 18S and 5.8S rRNAs; component of both the ribosomal small subunit processosome and the 90S preribosome |
| *RRP6* | *YOR001W* | *AFUA_3G09880* | Nuclear exosome exonuclease component; has 3'-5' exonuclease activity; involved in RNA processing, maturation, surveillance, degradation, tethering, and export; has similarity to E. coli RNase D and to human PM-Sc1 100 (EXOSC10) |
| *RRP8* | *YDR083W* | *AFUA_2G11450* | Nucleolar protein involved in rRNA processing, pre-rRNA cleavage at site A2; also involved in telomere maintenance; mutation is synthetically lethal with a gar1 mutation |
| *RRP9* | *YPR137W* | *AFUA_4G07500* | Protein involved in pre-rRNA processing, associated with U3 snRNP; component of small ribosomal subunit processosome; ortholog of the human U3-55k protein |
| *RRS1* | *YOR294W* | *AFUA_7G04430* | Essential protein that binds ribosomal protein L11 and is required for nuclear export of the 60S pre-ribosomal subunit during ribosome biogenesis; mouse homolog shows altered expression in Huntington's disease model mice |
| *RRT2* | *YBR246W* | *AFUA_3G08950* | Putative protein of unknown function; non-essential gene identified in a screen for mutants with increased levels of rDNA transcription; null mutants display a weak carboxypeptidase Y missorting/secretion phenotype |
| *RRT8* | *YOL047C YOL048C YAL018C* | *AFUA_1G06500 AFUA_2G02480* | Putative protein of unknown function; identified in a screen for mutants with increased levels of rDNA transcription; green fluorescent protein (GFP)-fusion protein localizes to lipid particles |
| *RSA4* | *YCR072C* | *AFUA_1G01990* | Ribosome biogenesis protein Rsa4 |
| *RSB1* | *YOR049C* | *AFUA_3G10770* | RTA1 domain protein |
| *RSC1* | *YGR056W YLR357W* | *AFUA_3G05560* | Component of the RSC chromatin remodeling complex; required for expression of mid-late sporulation-specific genes; contains two essential bromodomains, a bromo-adjacent homology domain, and an AT hook |
| *RSC8* | *YFR037C* | *AFUA_7G05510* | Component of the RSC chromatin remodeling complex; essential for viability and mitotic growth; homolog of SWI/SNF subunit Swi3p, but unlike Swi3p, does not activate transcription of reporters |
| *RSC9* | *YML127W* | *AFUA_6G07810* | Component of the RSC chromatin remodeling complex; DNA-binding protein involved in the synthesis of rRNA and in transcriptional repression and activation of genes regulated by the Target of Rapamycin pathway |
| *RSM18* | *YER050C* | *AFUA_2G06030* | Mitochondrial ribosomal protein of the small subunit, has similarity to E. coli S18 ribosomal protein |
| *RSM22* | *YKL155C* | *AFUA_2G02290* | Mitochondrial ribosomal protein of the small subunit; also predicted to be an S-adenosylmethionine-dependent methyltransferase |
| *RSM24* | *YDR175C* | *AFUA_4G13410* | Mitochondrial ribosomal protein of the small subunit |
| *RSM25* | *YIL093C* | *AFUA_4G07250* | Mitochondrial ribosomal protein of the small subunit |
| *RSM26* | *YJR101W* | *AFUA_6G07210* | Mitochondrial ribosomal protein of the small subunit |
| *RSM27* | *YGR215W* | *AFUA_5G04430* | Mitochondrial ribosomal protein of the small subunit |
| *RSM7* | *YJR113C* | *AFUA_1G04280* | Mitochondrial ribosomal protein of the small subunit, has similarity to E. coli S7 ribosomal protein |
| *RSP5* | *YER125W* | *AFUA_1G09500* | E3 ubiquitin ligase of the NEDD4 family; involved in regulating many cellular processes, including MVB sorting, heat shock response, transcription, and endocytosis; the human homolog is invovled in Liddle syndrome |
| *RSR1* | *YGR152C* | *AFUA_5G08950* | GTP-binding protein of the ras superfamily required for bud site selection, morphological changes in response to mating pheromone, and efficient cell fusion; localized to the plasma membrane; significantly similar to mammalian Rap GTPases |
| *RTC1* | *YOL138C* | *AFUA_2G03080* | Protein of unknown function; may interact with ribosomes, based on co-purification experiments; null mutation suppresses cdc13-1 temperature sensitivity |
| *RTC2* | *YBR147W YOL092W* | *AFUA_5G04100* | Protein of unknown function; identified in a screen for mutants with decreased levels of rDNA transcription; detected in highly purified mitochondria; null mutant suppresses cdc13-1; similar to a G-protein coupled receptor from S. pombe |
| *RTC3* | *YHR087W* | *AFUA_6G13330* | Protein of unknown function involved in RNA metabolism; has structural similarity to SBDS, the human protein mutated in Shwachman-Diamond Syndrome (the yeast SBDS ortholog = SDO1); null mutation suppresses cdc13-1 temperature sensitivity |
| *RTF1* | *YGL244W* | *AFUA_2G01900* | RNA polymerase II transcription elongation factor Rtf1p |
| *RTG2* | *YGL252C* | *AFUA_6G08350 AFUA_8G00730* | Retrograde regulation protein 2 |
| *RTN1* | *YDL204W YDR233C* | *AFUA_6G13670* | ER membrane protein that interacts with Sey1p to maintain ER morphology; interacts with exocyst subunit Sec6p, with Yip3p, and with Sbh1p; null mutant has an altered ER morphology; member of the RTNLA subfamily |
| *RTS1* | *YOR014W* | *AFUA_5G02560* | B-type regulatory subunit of protein phosphatase 2A (PP2A); homolog of the mammalian B' subunit of PP2A |
| *RTT10* | *YPL183C* | *AFUA_6G07490* | Cytoplasmic protein with a role in regulation of Ty1 transposition |
| *RTT103* | *YDR289C* | *AFUA_4G10030* | Protein that interacts with exonuclease Rat1p and Rai1p and plays a role in transcription termination by RNA polymerase II, has an RPR domain (carboxy-terminal domain interacting domain); also involved in regulation of Ty1 transposition |
| *RTT106* | *YNL206C* | *AFUA_6G06880* | Histone chaperone, involved in regulation of chromatin structure in both transcribed and silenced chromosomal regions; affects transcriptional elongation; has a role in regulation of Ty1 transposition |
| *RTT109* | *YLL002W* | *AFUA_5G09540* | DNA damage response protein Rtt109 |
| *RUD3* | *YOR216C* | *AFUA_1G08830* | Golgi matrix protein involved in the structural organization of the cis-Golgi; interacts genetically with COG3 and USO1 |
| *RVB1* | *YDR190C* | *AFUA_4G10730* | Essential protein involved in transcription regulation; component of chromatin remodeling complexes; required for assembly and function of the INO80 complex; also referred to as pontin; member of the RUVB-like protein family |
| *RVB2* | *YPL235W* | *AFUA_1G02410* | Essential protein involved in transcription regulation; component of chromatin remodeling complexes; required for assembly and function of the INO80 complex; also referred to as reptin; member of the RUVB-like protein family |
| *RVS161* | *YCR009C* | *AFUA_5G05950* | Amphiphysin-like lipid raft protein; interacts with Rvs167p and regulates polarization of the actin cytoskeleton, endocytosis, cell polarity, cell fusion and viability following starvation or osmotic stress |
| *RVS167* | *YDR388W* | *AFUA_3G14230* | Actin-associated protein, interacts with Rvs161p to regulate actin cytoskeleton, endocytosis, and viability following starvation or osmotic stress; homolog of mammalian amphiphysin |
| *RXT3* | *YDL076C* | *AFUA_2G12520* | Rxt3p |
| *SAC1* | *YKL212W* | *AFUA_4G08050* | Phosphatidylinositol phosphate phosphatase involved in hydrolysis of PtdIns[4]P; transmembrane protein localizes to ER and Golgi; involved in protein trafficking and processing, secretion, and cell wall maintenance |
| *SAC3* | *YDR159W* | *AFUA_5G08000* | Nuclear pore-associated protein, required for biogenesis of the small ribosomal subunit; forms a complex with Thp1p that is involved in transcription and in mRNA export from the nucleus |
| *SAC6* | *YDR129C* | *AFUA_2G07420* | Fimbrin, actin-bundling protein; cooperates with Scp1p in the organization and maintenance of the actin cytoskeleton |
| *SAD1* | *YFR005C* | *AFUA_4G07520* | Conserved zinc-finger domain protein involved in pre-mRNA splicing, required for assembly of U4 snRNA into the U4/U6 particle |
| *SAE3* | *YHR079C-A* | *AFUA_5G06080* | Meiosis specific protein involved in DMC1-dependent meiotic recombination, forms heterodimer with Mei5p; proposed to be an assembly factor for Dmc1p |
| *SAM37* | *YMR060C* | *AFUA_2G03840* | Component of the Sorting and Assembly Machinery of the mitochondrial outer membrane, which binds precursors of beta-barrel proteins and facilitates their outer membrane insertion; contributes to SAM complex stability |
| *SAM50* | *YNL026W* | *AFUA_4G04620* | Essential component of the Sorting and Assembly Machinery of the mitochondrial outer membrane, which binds precursors of beta-barrel proteins and facilitates their outer membrane insertion; homologous to bacterial Omp85 |
| *SAP1* | *YER047C* | *AFUA_6G12560* | Putative ATPase of the AAA family, interacts with the Sin1p transcriptional repressor in the two-hybrid system |
| *SAT4* | *YCR008W* | *AFUA_5G05960* | Serine/threonine protein kinase |
| *SAY1* | *YGR263C* | *AFUA_5G09800 AFUA_2G04300 AFUA_8G01280 AFUA_1G13270 AFUA_8G02590* | 6-hexanolactone hydrolase |
| *SBH2* | *YER019C-A* | *AFUA_1G04890* | Translocon protein Sec61beta |
| *SCC2* | *YDR180W* | *AFUA_2G05470* | Sister chromatid cohesion protein Mis4 |
| *SCJ1* | *YMR214W* | *AFUA_2G08300* | One of several homologs of bacterial chaperone DnaJ, located in the ER lumen where it cooperates with Kar2p to mediate maturation of proteins |
| *SCL1* | *YGL011C* | *AFUA_3G11300* | Alpha 1 subunit of the 20S proteasome involved in the degradation of ubiquitinated substrates; 20S proteasome is the core complex of the 26S proteasome; essential for growth; detected in the mitochondria |
| *SCM3* | *YDL139C* | *AFUA_8G05190* | Nonhistone component of centromeric chromatin that binds stoichiometrically to CenH3-H4 histones, required for kinetochore assembly; contains nuclear export signal (NES); required for G2/M progression and localization of Cse4p |
| *SCO1* | *YBR024W YBR037C* | *AFUA_3G07360* | Protein anchored to the mitochondrial inner membrane, similar to Sco1p and may have a redundant function with Sco1p in delivery of copper to cytochrome c oxidase; interacts with Cox2p |
| *SCP1* | *YOR367W* | *AFUA_4G13250* | Component of yeast cortical actin cytoskeleton, binds and cross links actin filaments; originally identified by its homology to calponin but the Scp1p domain structure is more similar to transgelin |
| *SCP160* | *YJL080C* | *AFUA_2G04700* | Essential RNA-binding G protein effector of mating response pathway, mainly associated with nuclear envelope and ER, interacts in mRNA-dependent manner with translating ribosomes via multiple KH domains, similar to vertebrate vigilins |
| *SCS2* | *YBL091C-A YER120W* | *AFUA_4G06950* | Integral ER membrane protein that regulates phospholipid metabolism via an interaction with the FFAT motif of Opi1p, also involved in telomeric silencing, disruption causes inositol auxotrophy above 34 degrees C, VAP homolog |
| *SCS7* | *YMR272C* | *AFUA_1G15820* | Fatty acid hydroxylase |
| *SCW10* | *YGR279C YMR305C* | *AFUA_6G12380* | Cell wall protein with similarity to glucanases; may play a role in conjugation during mating based on mutant phenotype and its regulation by Ste12p |
| *SCW11* | *YGL028C* | *AFUA_8G05610* | Cell wall protein with similarity to glucanases; may play a role in conjugation during mating based on its regulation by Ste12p |
| *SCY1* | *YGL083W* | *AFUA_4G06150* | Putative kinase, suppressor of GTPase mutant, similar to bovine rhodopsin kinase |
| *SDA1* | *YGR245C* | *AFUA_1G15030* | Highly conserved nuclear protein required for actin cytoskeleton organization and passage through Start, plays a critical role in G1 events, binds Nap1p, also involved in 60S ribosome biogenesis |
| *SDC1* | *YDR469W* | *AFUA_7G05270* | COMPASS complex subunit Sdc1 |
| *SDH2* | *YLL041C* | *AFUA_5G10370* | Iron-sulfur protein subunit of succinate dehydrogenase (Sdh1p, Sdh2p, Sdh3p, Sdh4p), which couples the oxidation of succinate to the transfer of electrons to ubiquinone as part of the TCA cycle and the mitochondrial respiratory chain |
| *SDO1* | *YLR022C* | *AFUA_6G13530* | Essential protein involved in 60S ribosome maturation; ortholog of the human protein responsible for autosomal recessive Shwachman-Bodian-Diamond Syndrome; highly conserved across archae and eukaryotes |
| *SDS22* | *YKL193C* | *AFUA_1G04800* | Conserved nuclear regulatory subunit of Glc7p type 1 protein serine-threonine phosphatase (PP1), functions positively with Glc7p to promote dephosphorylation of nuclear substrates required for chromosome transmission during mitosis |
| *SDS23* | *YBR214W YGL056C* | *AFUA_1G06660* | One of two S. cerevisiae homologs of the S. pombe Sds23 protein, which is implicated in APC/cyclosome regulation; involved in cell separation during budding; may play an indirect role in fluid-phase endocytosis |
| *SDS3* | *YIL084C* | *AFUA_3G13150* | Component of the Rpd3p/Sin3p deacetylase complex required for its structural integrity and catalytic activity, involved in transcriptional silencing and required for sporulation; cells defective in SDS3 display pleiotropic phenotypes |
| *SEC1* | *YDR164C* | *AFUA_5G10810* | Sec1 family superfamily |
| *SEC10* | *YLR166C* | *AFUA_8G02790* | Essential 100kDa subunit of the exocyst complex (Sec3p, Sec5p, Sec6p, Sec8p, Sec10p, Sec15p, Exo70p, and Exo84p), which has the essential function of mediating polarized targeting of secretory vesicles to active sites of exocytosis |
| *SEC11* | *YIR022W* | *AFUA_3G12840* | 18kDa catalytic subunit of the Signal Peptidase Complex which cleaves the signal sequence of proteins targeted to the endoplasmic reticulum |
| *SEC15* | *YGL233W* | *AFUA_6G05290* | Essential 113kDa subunit of the exocyst complex (Sec3p, Sec5p, Sec6p, Sec8p, Sec10p, Sec15p, Exo70p, and Exo84p), which mediates polarized targeting of vesicles to active sites of exocytosis; Sec15p associates with Sec4p and vesicles |
| *SEC3* | *YER008C* | *AFUA_1G04360* | Exocyst complex component Sec3 |
| *SEC39* | *YLR440C* | *AFUA_1G05810* | Component of the Dsl1p tethering complex that interacts with ER SNAREs Sec20p and Use1p; proposed to be involved in protein secretion; localizes to the ER and nuclear envelope |
| *SEC5* | *YDR166C* | *AFUA_1G12790* | Essential 107kDa subunit of the exocyst complex (Sec3p, Sec5p, Sec6p, Sec8p, Sec10p, Sec15p, Exo70p, and Exo84p), which has the essential function of mediating polarized targeting of secretory vesicles to active sites of exocytosis |
| *SEC53* | *YFL045C* | *AFUA_6G06580* | Phosphomannomutase, involved in synthesis of GDP-mannose and dolichol-phosphate-mannose; required for folding and glycosylation of secretory proteins in the ER lumen |
| *SEC59* | *YMR013C* | *AFUA_4G09060* | Dolichol kinase, catalyzes the terminal step in dolichyl monophosphate biosynthesis; required for viability and for normal rates of lipid intermediate synthesis and protein N-glycosylation |
| *SEC6* | *YIL068C* | *AFUA_4G10490* | Essential 88kDa subunit of the exocyst complex, which mediates polarized targeting of secretory vesicles to active sites of exocytosis; dimeric form of Sec6p interacts with Sec9p in vitro and inhibits t-SNARE assembly |
| *SEC61* | *YLR378C* | *AFUA_5G08130* | Essential subunit of Sec61 complex (Sec61p, Sbh1p, and Sss1p); forms a channel for SRP-dependent protein import and retrograde transport of misfolded proteins out of the ER; with Sec63 complex allows SRP-independent protein import into ER |
| *SEC62* | *YPL094C* | *AFUA_2G12580* | Essential subunit of Sec63 complex (Sec63p, Sec62p, Sec66p and Sec72p); with Sec61 complex, Kar2p/BiP and Lhs1p forms a channel competent for SRP-dependent and post-translational SRP-independent protein targeting and import into the ER |
| *SEC63* | *YOR254C* | *AFUA_1G14940* | Essential subunit of Sec63 complex (Sec63p, Sec62p, Sec66p and Sec72p); with Sec61 complex, Kar2p/BiP and Lhs1p forms a channel competent for SRP-dependent and post-translational SRP-independent protein targeting and import into the ER |
| *SEC65* | *YML105C* | *AFUA_1G16820* | Signal recognition particle 19 kDa protein |
| *SEC66* | *YBR171W* | *AFUA_8G04260* | Non-essential subunit of Sec63 complex (Sec63p, Sec62p, Sec66p and Sec72p); with Sec61 complex, Kar2p/BiP and Lhs1p forms a channel competent for SRP-dependent and post-translational SRP-independent protein targeting and import into the ER |
| *SEC8* | *YPR055W* | *AFUA_5G08040* | Essential 121kDa subunit of the exocyst complex (Sec3p, Sec5p, Sec6p, Sec8p, Sec10p, Sec15p, Exo70p, and Exo84p), which has the essential function of mediating polarized targeting of secretory vesicles to active sites of exocytosis |
| *SEC9* | *YGR009C* | *AFUA_2G13760* | Plasma membrane SNARE protein (Sec9) |
| *SED5* | *YLR026C* | *AFUA_3G07590* | ER-Golgi SNARE complex subunit (Sed5) |
| *SEE1* | *YIL064W* | *AFUA_2G02750* | Protein with a role in intracellular transport; has sequence similarity to S-adenosylmethionine-dependent methyltransferases of the seven beta-strand family |
| *SEH1* | *YGL100W* | *AFUA_2G11230* | Nuclear pore protein that is part of the evolutionarily conserved Nup84p complex (Nup84p, Nup85p, Nup120p, Nup145p, and Seh1p); homologous to Sec13p |
| *SEN1* | *YLR430W* | *AFUA_6G02060* | Presumed helicase required for RNA polymerase II transcription termination and processing of RNAs; homolog of Senataxin which causes Ataxia-Oculomotor Apraxia 2 and a dominant form of amyotrophic lateral sclerosis |
| *SEN2* | *YLR105C* | *AFUA_5G04010* | TRNA-splicing endonuclease subunit Sen2 |
| *SEN34* | *YAR008W* | *AFUA_5G11140* | TRNA-splicing endonuclease subunit sen34 |
| *SEN54* | *YPL083C* | *AFUA_6G10540* | TRNA splicing endonuclease subunit (Sen54) |
| *SES1* | *YDR023W* | *AFUA_5G05490* | SerRS; seryl-tRNA synthetase |
| *SET1* | *YHR119W* | *AFUA_6G06335* | SET domain protein |
| *SET3* | *YJL105W YKR029C* | *AFUA_2G11210* | Defining member of the SET3 histone deacetylase complex which is a meiosis-specific repressor of sporulation genes; necessary for efficient transcription by RNAPII; one of two yeast proteins that contains both SET and PHD domains |
| *SET5* | *YHR207C* | *AFUA_2G10080* | SET and MYND domain protein |
| *SEY1* | *YOR165W* | *AFUA_1G12180* | GTPase with a role in ER morphology; interacts physically and genetically with Yop1p and Rtn1p; possible functional ortholog of mammalian atlastins, defects in which cause a form of hereditary spastic paraplegia; homolog of Arabidopsis RHD3 |
| *SFA1* | *YDL168W* | *AFUA_2G01040* | Bifunctional enzyme containing both alcohol dehydrogenase and glutathione-dependent formaldehyde dehydrogenase activities, functions in formaldehyde detoxification and formation of long chain and complex alcohols, regulated by Hog1p-Sko1p |
| *SFB3* | *YHR098C* | *AFUA_3G09700* | Component of the Sec23p-Sfb3p heterodimer of the COPII vesicle coat, required for cargo selection during vesicle formation in ER to Golgi transport; homologous to Sec24p and Sfb2p |
| *SFC1* | *YJR095W* | *AFUA_2G16930* | Mitochondrial succinate-fumarate transporter, transports succinate into and fumarate out of the mitochondrion; required for ethanol and acetate utilization |
| *SFH1* | *YLR321C* | *AFUA_4G06120* | Component of the RSC chromatin remodeling complex; essential gene required for cell cycle progression and maintenance of proper ploidy; phosphorylated in the G1 phase of the cell cycle; Snf5p paralog |
| *SFH5* | *YJL145W* | *AFUA_5G03690* | Non-classical phosphatidylinositol transfer protein (PITP); exhibits PI- but not PC-transfer activity; localizes to the peripheral endoplasmic reticulum, cytosol and microsomes; similar to Sec14p |
| *SFI1* | *YLL003W* | *AFUA_1G13700* | Centrin duplication, localizes to the half-bridge of the SPB, required for progression through G(2)-M transition, has similarity to Xenopus laevis XCAP-C |
| *SFK1* | *YKL051W* | *AFUA_6G06640* | Plasma membrane protein that may act together with or upstream of Stt4p to generate normal levels of the essential phospholipid PI4P, at least partially mediates proper localization of Stt4p to the plasma membrane |
| *SFL1* | *YOR140W* | *AFUA_4G04502* | Flocculation suppression protein |
| *SFP1* | *YLR403W* | *AFUA_1G14750* | C2H2 transcription factor (Sfp1) |
| *SFT2* | *YBL102W* | *AFUA_1G09680* | Non-essential tetra-spanning membrane protein found mostly in the late Golgi, can suppress some sed5 alleles; may be part of the transport machinery, but precise function is unknown; similar to mammalian syntaxin 5 |
| *SGA1* | *YIL099W* | *AFUA_2G00690 AFUA_3G00610* | Intracellular sporulation-specific glucoamylase involved in glycogen degradation; induced during starvation of a/a diploids late in sporulation, but dispensable for sporulation |
| *SGF29* | *YCL010C* | *AFUA_1G13290* | Probable subunit of SAGA histone acetyltransferase complex |
| *SGF73* | *YGL066W* | *AFUA_2G05770* | SAGA complex component (Sgf73) |
| *SGM1* | *YJR134C* | *AFUA_1G10690* | Protein of unknown function, required for wild-type growth rate on galactose and mannose; localizes to COPI coated vesicles and the Golgi apparatus |
| *SGS1* | *YMR190C* | *AFUA_2G04960* | Nucleolar DNA helicase of the RecQ family involved in genome integrity maintenance; regulates chromosome synapsis and meiotic joint molecule/crossover formation; similar to human BLM and WRN proteins implicated in Bloom and Werner syndromes |
| *SGT1* | *YOR057W* | *AFUA_5G04090* | Cochaperone protein; regulates activity of adenylyl cyclase Cyr1p; involved in kinetochore complex assembly; associates with the SCF ubiquitin ligase complex; acts as a linker between Skp1p and HSP90 complexes |
| *SGT2* | *YOR007C* | *AFUA_1G09830* | Glutamine-rich cytoplasmic protein of unknown function; contains tetratricopeptide repeats, which often mediate protein-protein interactions; has similarity to human SGT, which is a cochaperone that negatively regulates Hsp70 |
| *SHE1* | *YBL031W* | *AFUA_4G04320* | Mitotic spindle protein that interacts with components of the Dam1 complex, its effector Sli15p, and microtubule-associated protein Bim1p; also localizes to nuclear microtubules and to the bud neck in a ring-shaped structure |
| *SHE9* | *YDR393W* | *AFUA_5G10600* | Mitochondrial inner membrane protein required for normal mitochondrial morphology, may be involved in fission of the inner membrane; forms a homo-oligomeric complex |
| *SHM1* | *YBR263W* | *AFUA_2G07810* | Mitochondrial serine hydroxymethyltransferase, converts serine to glycine plus 5,10 methylenetetrahydrofolate; involved in generating precursors for purine, pyrimidine, amino acid, and lipid biosynthesis; reverse reaction generates serine |
| *SHM2* | *YLR058C* | *AFUA_3G09320* | Cytosolic serine hydroxymethyltransferase, converts serine to glycine plus 5,10 methylenetetrahydrofolate; major isoform involved in generating precursors for purine, pyrimidine, amino acid, and lipid biosynthesis |
| *SHO1* | *YER118C* | *AFUA_5G08420* | High osmolarity signaling protein Sho1 |
| *SHR3* | *YDL212W* | *AFUA_4G09660* | Endoplasmic reticulum packaging chaperone, required for incorporation of amino acid permeases into COPII coated vesicles for transport to the cell surface |
| *SHY1* | *YGR112W* | *AFUA_3G06340* | Mitochondrial inner membrane protein required for assembly of cytochrome c oxidase (complex IV); associates with complex IV assembly intermediates and complex III/complex IV supercomplexes; similar to human SURF1 involved in Leigh Syndrome |
| *SIN3* | *YOL004W* | *AFUA_8G05570* | Component of the Sin3p-Rpd3p histone deacetylase complex, involved in transcriptional repression and activation of diverse processes, including mating-type switching and meiosis; involved in the maintenance of chromosomal integrity |
| *SIN4* | *YNL236W* | *AFUA_1G10525* | RNA polymerase II Mediator complex subunit Sin4, putative |
| *SIP5* | *YMR140W* | *AFUA_8G04180* | Protein of unknown function; interacts with both the Reg1p/Glc7p phosphatase and the Snf1p kinase |
| *SIS1* | *YNL007C* | *AFUA_5G07340* | DnaJ domain protein Psi |
| *SIT1* | *YEL065W* | *AFUA_7G06060* | Ferrioxamine B transporter, member of the ARN family of transporters that specifically recognize siderophore-iron chelates; transcription is induced during iron deprivation and diauxic shift; potentially phosphorylated by Cdc28p |
| *SIW14* | *YNL032W* | *AFUA_4G07000* | Tyrosine phosphatase family protein |
| *SKI2* | *YLR398C* | *AFUA_2G10000* | Ski complex component and putative RNA helicase, mediates 3'-5' RNA degradation by the cytoplasmic exosome; null mutants have superkiller phenotype of increased viral dsRNAs and are synthetic lethal with mutations in 5'-3' mRNA decay |
| *SKI3* | *YPR189W* | *AFUA_3G08810* | Ski complex component and TPR protein, mediates 3'-5' RNA degradation by the cytoplasmic exosome; null mutants have superkiller phenotype of increased viral dsRNAs and are synthetic lethal with mutations in 5'-3' mRNA decay |
| *SKN7* | *YHR206W* | *AFUA_6G12522* | Nuclear response regulator and transcription factor, part of a branched two-component signaling system; required for optimal induction of heat-shock genes in response to oxidative stress; involved in osmoregulation |
| *SKO1* | *YNL167C* | *AFUA_3G11330* | Basic leucine zipper transcription factor of the ATF/CREB family, forms a complex with Tup1p and Ssn6p to both activate and repress transcription; cytosolic and nuclear protein involved in osmotic and oxidative stress responses |
| *SKP1* | *YDR328C* | *AFUA_5G06060* | Evolutionarily conserved kinetochore protein that is part of multiple protein complexes, including the SCF ubiquitin ligase complex, the CBF3 complex that binds centromeric DNA, and the RAVE complex that regulates assembly of the V-ATPase |
| *SKY1* | *YMR216C* | *AFUA_4G03140* | SR protein kinase involved in regulating proteins involved in mRNA metabolism and cation homeostasis; similar to human SRPK1 |
| *SLA1* | *YBL007C* | *AFUA_8G04520* | Cytoskeletal protein binding protein required for assembly of the cortical actin cytoskeleton; interacts with proteins regulating actin dynamics and proteins required for endocytosis; found in the nucleus and cell cortex; has 3 SH3 domains |
| *SLA2* | *YNL243W* | *AFUA_3G06140* | Cytoskeleton assembly control protein Sla2 |
| *SLD5* | *YDR489W* | *AFUA_6G08210* | GINS DNA replication complex subunit Sld5 |
| *SLF1* | *YCL037C YDR515W* | *AFUA_1G09770* | Cytoplasmic RNA-binding protein that associates with translating ribosomes; involved in heme regulation of Hap1p as a component of the HMC complex, also involved in the organization of actin filaments; contains a La motif |
| *SLH1* | *YGR271W* | *AFUA_4G03070* | Putative RNA helicase related to Ski2p, involved in translation inhibition of non-poly(A) mRNAs; required for repressing propagation of dsRNA viruses |
| *SLM5* | *YCR024C* | *AFUA_3G09630* | Asparaginyl-tRNA synthetase Slm5 |
| *SLN1* | *YIL147C* | *AFUA_2G00660* | Histidine kinase osmosensor that regulates a MAP kinase cascade; transmembrane protein with an intracellular kinase domain that signals to Ypd1p and Ssk1p, thereby forming a phosphorelay system similar to bacterial two-component regulators |
| *SLP1* | *YOR154W* | *AFUA_5G06480* | Integral membrane protein of unknown function; member of the SUN-like family of proteins; genetic interactions suggest a role in folding of ER membrane proteins |
| *SLT2* | *YHR030C* | *AFUA_4G13720* | Serine/threonine MAP kinase involved in regulating the maintenance of cell wall integrity and progression through the cell cycle; regulated by the PKC1-mediated signaling pathway |
| *SLU7* | *YDR088C* | *AFUA_3G06770* | RNA splicing factor, required for ATP-independent portion of 2nd catalytic step of spliceosomal RNA splicing; interacts with Prp18p; contains zinc knuckle domain |
| *SLX1* | *YBR228W* | *AFUA_5G03450* | GIY-YIG catalytic domain containing protein |
| *SLY1* | *YDR189W* | *AFUA_3G14320* | Hydrophilic protein involved in vesicle trafficking between the ER and Golgi; SM family protein that binds the tSNARE Sed5p and stimulates its assembly into a trans-SNARE membrane-protein complex |
| *SMB1* | *YER029C* | *AFUA_4G07740* | Small nuclear ribonucleoprotein SmB |
| *SMC1* | *YFL008W* | *AFUA_3G08260* | Cohesin complex subunit (Psm1) |
| *SMC2* | *YFR031C* | *AFUA_2G11110* | Nuclear condensin complex subunit Smc2 |
| *SMC3* | *YJL074C* | *AFUA_2G14080* | Chromosome segregation protein SudA |
| *SMC4* | *YLR086W* | *AFUA_2G02170* | Nuclear condensin complex subunit Smc4 |
| *SMD1* | *YGR074W* | *AFUA_2G05110* | Small nuclear ribonucleoprotein SmD1 |
| *SMD2* | *YLR275W* | *AFUA_5G12910* | Small nuclear ribonucleoprotein SmD2 |
| *SMD3* | *YLR147C* | *AFUA_4G10240* | Small nuclear ribonucleoprotein SmD3 |
| *SME1* | *YOR159C* | *AFUA_7G05980* | Small nuclear ribonucleoprotein SmE |
| *SMF1* | *YHR050W YOL122C* | *AFUA_4G10990* | Divalent metal ion transporter involved in manganese homeostasis; has broad specificity for di-valent and tri-valent metals; post-translationally regulated by levels of metal ions; member of the Nramp family of metal transport proteins |
| *SMI1* | *YGR229C* | *AFUA_5G05770* | Protein involved in the regulation of cell wall synthesis; proposed to be involved in coordinating cell cycle progression with cell wall integrity |
| *SMM1* | *YNR015W* | *AFUA_3G08390* | Dihydrouridine synthase, member of a family of dihydrouridine synthases including Dus1p, Smm1p, Dus3p, and Dus4p; modifies uridine residues at position 20 of cytoplasmic tRNAs |
| *SMX2* | *YFL017W-A* | *AFUA_8G04960* | Small nuclear ribonucleoprotein SmG |
| *SMX3* | *YPR182W* | *AFUA_2G15500* | Small nuclear ribonucleoprotein SmF |
| *SMY2* | *YBR172C YPL105C* | *AFUA_2G13290* | Protein of unknown function involved in COPII vesicle formation; interacts with the Sec23p/Sec24p subcomplex; overexpression suppresses the temperature sensitivity of a myo2 mutant; has similarity to S. pombe Mpd2 |
| *SNA2* | *YDR525W-A YJL151C* | *AFUA_5G10590* | Integral membrane protein localized to vacuolar intralumenal vesicles, computational analysis of large-scale protein-protein interaction data suggests a possible role in either cell wall synthesis or protein-vacuolar targeting |
| *SNF1* | *YDR477W* | *AFUA_2G01700* | AMP-activated serine/threonine protein kinase found in a complex containing Snf4p and members of the Sip1p/Sip2p/Gal83p family; required for transcription of glucose-repressed genes, thermotolerance, sporulation, and peroxisome biogenesis |
| *SNF4* | *YGL115W* | *AFUA_5G12990* | Activating gamma subunit of the AMP-activated Snf1p kinase complex (contains Snf1p and a Sip1p/Sip2p/Gal83p family member); activates glucose-repressed genes, represses glucose-induced genes; role in sporulation, and peroxisome biogenesis |
| *SNF5* | *YBR289W* | *AFUA_2G13480* | Snf5p |
| *SNF5* | *YBR289W* | *AFUA_2G16840* | SWI-SNF complex subunit (Snf5) |
| *SNF7* | *YLR025W* | *AFUA_1G06420* | One of four subunits of the endosomal sorting complex required for transport III pathway; recruited from the cytoplasm to endosomal membranes |
| *SNG1* | *YGR197C YJR015W* | *AFUA_4G03620* | Putative protein of unknown function; localizes to the endoplasmic reticulum and cytoplasm; predicted to encode a membrane transporter based on phylogenetic analysis; YJR015W is a non-essential gene |
| *SNO1* | *YMR095C YFL060C YNL334C* | *AFUA_2G08580* | Protein of unknown function, nearly identical to Sno2p; expression is induced before the diauxic shift and also in the absence of thiamin |
| *SNP1* | *YIL061C* | *AFUA_5G13480* | U1 small nuclear ribonucleoprotein 70 kDa |
| *SNT1* | *YCR033W* | *AFUA_5G01730* | MYB DNA-binding domain protein |
| *SNT2* | *YGL131C* | *AFUA_1G05240* | DNA binding protein with similarity to the S. pombe Snt2 protein; computational analysis suggests a role in regulation of expression of genes encoding amine transporters |
| *SNU13* | *YEL026W* | *AFUA_2G05950* | RNA binding protein, part of U3 snoRNP involved in rRNA processing, part of U4/U6-U5 tri-snRNP involved in mRNA splicing, similar to human 15.5K protein |
| *SNU23* | *YDL098C* | *AFUA_1G14060* | Component of U4/U6.U5 snRNP involved in mRNA splicing via spliceosome |
| *SNU66* | *YOR308C* | *AFUA_4G10420* | Component of the U4/U6.U5 snRNP complex involved in pre-mRNA splicing via spliceosome; also required for pre-5S rRNA processing and may act in concert with Rnh70p; has homology to human SART-1 |
| *SNX3* | *YOR357C* | *AFUA_3G06880* | Sorting nexin Snx3 |
| *SNX4* | *YJL036W* | *AFUA_4G12950* | Vacuolar targeting protein Atg24 |
| *SNX41* | *YDR425W* | *AFUA_2G14160* | Autophagy protein Atg20 |
| *SOD1* | *YJR104C* | *AFUA_5G09240* | Cytosolic copper-zinc superoxide dismutase; some mutations are analogous to those that cause ALS in humans |
| *SOD2* | *YHR008C* | *AFUA_4G11580* | Mitochondrial superoxide dismutase, protects cells against oxygen toxicity; phosphorylated |
| *SOF1* | *YLL011W* | *AFUA_1G06290* | Essential protein required for biogenesis of 40S ribosomal subunit; has similarity to the beta subunit of trimeric G-proteins and the splicing factor Prp4p |
| *SOG2* | *YOR353C* | *AFUA_4G07260* | Key component of the RAM signaling network, required for proper cell morphogenesis and cell separation after mitosis |
| *SOK1* | *YDR006C* | *AFUA_4G07280* | Protein whose overexpression suppresses the growth defect of mutants lacking protein kinase A activity; involved in cAMP-mediated signaling; localized to the nucleus; similar to the mouse testis-specific protein PBS13 |
| *SPA2* | *YLL021W* | *AFUA_2G03710* | Component of the polarisome, which functions in actin cytoskeletal organization during polarized growth; acts as a scaffold for Mkk1p and Mpk1p cell wall integrity signaling components; potential Cdc28p substrate |
| *SPB1* | *YCL054W* | *AFUA_5G12100* | AdoMet-dependent methyltransferase involved in rRNA processing and 60S ribosomal subunit maturation; methylates G2922 in the tRNA docking site of the large subunit rRNA and in the absence of snR52, U2921; suppressor of PAB1 mutants |
| *SPB4* | *YFL002C* | *AFUA_3G13280* | Putative ATP-dependent RNA helicase, nucleolar protein required for synthesis of 60S ribosomal subunits at a late step in the pathway; sediments with 66S pre-ribosomes in sucrose gradients |
| *SPC1* | *YJR010C-A* | *AFUA_5G05800* | Microsomal signal peptidase Spc12 |
| *SPC105* | *YGL093W* | *AFUA_2G04140* | Chromosome segregation protein Spc105 |
| *SPC19* | *YDR201W* | *AFUA_2G02280* | Essential subunit of the Dam1 complex (aka DASH complex), couples kinetochores to the force produced by MT depolymerization thereby aiding in chromosome segregation; also localized to nuclear side of spindle pole body |
| *SPC2* | *YML055W* | *AFUA_8G05340* | Signal peptidase complex component |
| *SPC24* | *YMR117C* | *AFUA_2G13330* | Component of the evolutionarily conserved kinetochore-associated Ndc80 complex (Ndc80p-Nuf2p-Spc24p-Spc25p); involved in chromosome segregation, spindle checkpoint activity and kinetochore clustering |
| *SPC3* | *YLR066W* | *AFUA_5G03220* | Microsomal signal peptidase subunit (gp23) |
| *SPC97* | *YHR172W* | *AFUA_2G11430* | Component of the microtubule-nucleating Tub4p complex; interacts with Spc110p at the spindle pole body inner plaque and with Spc72p at the SPB outer plaque |
| *SPC98* | *YNL126W* | *AFUA_3G11320* | Component of the microtubule-nucleating Tub4p complex; interacts with Spc110p at the spindle pole body inner plaque and with Spc72p at the SPB outer plaque |
| *SPE2* | *YOL052C* | *AFUA_5G03670* | S-adenosylmethionine decarboxylase, required for the biosynthesis of spermidine and spermine; cells lacking Spe2p require spermine or spermidine for growth in the presence of oxygen but not when grown anaerobically |
| *SPN1* | *YPR133C* | *AFUA_1G12260* | Protein that interacts with Spt6p and copurifies with Spt5p and RNA polymerase II, probable transcriptional elongation factor; metazoan homologs contain an acidic N terminus; mutations in the gene confer an Spt- phenotype |
| *SPO11* | *YHL022C* | *AFUA_5G04070* | Meiosis-specific protein that initiates meiotic recombination by catalyzing the formation of double-strand breaks in DNA via a transesterification reaction; required for homologous chromosome pairing and synaptonemal complex formation |
| *SPO71* | *YDR104C* | *AFUA_3G13830* | Meiosis-specific protein of unknown function, required for spore wall formation during sporulation; dispensable for both nuclear divisions during meiosis |
| *SPO73* | *YER046W* | *AFUA_3G09930* | Meiosis-specific protein of unknown function, required for spore wall formation during sporulation; dispensible for both nuclear divisions during meiosis |
| *SPP1* | *YPL138C* | *AFUA_3G12030* | PHD transcription factor |
| *SPP382* | *YLR424W* | *AFUA_4G11570* | Essential protein that forms a dimer with Ntr2p; also forms a trimer, with Ntr2p and Prp43p, that is involved in spliceosome disassembly; found also in a multisubunit complex with the splicing factor Clf1p; suppressor of prp38-1 mutation |
| *SPS19* | *YNL202W* | *AFUA_5G07470* | Oxidoreductase, short-chain dehydrogenase/reductase family |
| *SPT10* | *YJL127C* | *AFUA_1G06720* | Putative histone acetylase, sequence-specific activator of histone genes, binds specifically and highly cooperatively to pairs of UAS elements in core histone promoters, functions at or near the TATA box |
| *SPT14* | *YPL175W* | *AFUA_1G16950* | Phosphatidylinositol:UDP-GlcNAc transferase subunit PIG-A |
| *SPT15* | *YER148W* | *AFUA_3G10120* | RNA polymerase I and III transcription factor complex component Tbp |
| *SPT16* | *YGL207W* | *AFUA_1G07720* | Transcription elongation complex subunit (Cdc68) |
| *SPT20* | *YOL148C* | *AFUA_1G16580* | Spt20p |
| *SPT21* | *YMR179W* | *AFUA_5G12900* | Protein required for normal transcription at several loci including HTA2-HTB2 and HHF2-HHT2, but not required at the other histone loci; functionally related to Spt10p; involved in telomere maintenance |
| *SPT3* | *YDR392W* | *AFUA_1G14030* | SAGA-like transcriptional regulatory complex subunit Spt3 |
| *SPT4* | *YGR063C* | *AFUA_5G06690* | Protein involved in the regulating Pol I and Pol II transcription, pre-mRNA processing, kinetochore function, and gene silencing; forms a complex with Spt5p |
| *SPT5* | *YML010W* | *AFUA_4G08500* | Protein involved in regulating Pol I and Pol II transcription and pre-mRNA processing; forms a complex with Spt4p; contains a C-terminal repeat domain that is a target for phosphorylation by Sgv1p |
| *SPT6* | *YGR116W* | *AFUA_3G05890* | Transcription elongation factor SPT6 |
| *SPT7* | *YBR081C* | *AFUA_3G11000* | Transcriptional activator spt7 |
| *SPT8* | *YLR055C* | *AFUA_5G09080* | Transcription factor (SPT8) |
| *SQS1* | *YNL224C* | *AFUA_3G05330* | R3H and G-patch domain protein |
| *SQT1* | *YIR012W* | *AFUA_5G07390* | Essential protein involved in a late step of 60S ribosomal subunit assembly or modification; contains multiple WD repeats; interacts with Qsr1p in a two-hybrid assay |
| *SRB4* | *YER022W* | *AFUA_4G00950* | RNA polymerase II mediator complex component SRB4, putative |
| *SRB5* | *YGR104C* | *AFUA_6G06830* | RNA polymerase II mediator complex subunit Srb5, putative |
| *SRB7* | *YDR308C* | *AFUA_2G13350* | RNA polymerase II transcription mediator complex subunit Srb7 |
| *SRB8* | *YCR081W* | *AFUA_3G06250* | RNA polymerase II mediator complex component Srb8, putative |
| *SRM1* | *YGL097W* | *AFUA_4G04740* | Nucleotide exchange factor for Gsp1p, localizes to the nucleus, required for nucleocytoplasmic trafficking of macromolecules; suppressor of the pheromone response pathway; potentially phosphorylated by Cdc28p |
| *SRO7* | *YBL106C YPR032W* | *AFUA_3G11040* | Effector of Rab GTPase Sec4p, forms a complex with Sec4p and t-SNARE Sec9p; involved in exocytosis and docking and fusion of post-Golgi vesicles with plasma membrane; homolog of Sro77p and Drosophila lgl tumor suppressor |
| *SRP1* | *YNL189W* | *AFUA_2G16090* | Karyopherin alpha homolog, forms a dimer with karyopherin beta Kap95p to mediate import of nuclear proteins, binds the nuclear localization signal of the substrate during import; may also play a role in regulation of protein degradation |
| *SRP101* | *YDR292C* | *AFUA_6G03850* | Signal recognition particle receptor - alpha subunit; contain GTPase domains; involved in SRP-dependent protein targeting; interacts with Srp102p |
| *SRP102* | *YKL154W* | *AFUA_2G07600* | Signal recognition particle receptor beta subunit; involved in SRP-dependent protein targeting; anchors Srp101p to the ER membrane |
| *SRP54* | *YPR088C* | *AFUA_5G03880* | Signal recognition particle subunit (homolog of mammalian SRP54); contains the signal sequence-binding activity of SRP, interacts with the SRP RNA, and mediates binding of SRP to signal receptor; contains GTPase domain |
| *SRP68* | *YPL243W* | *AFUA_1G03940* | Core component of the signal recognition particle ribonucleoprotein complex that functions in targeting nascent secretory proteins to the endoplasmic reticulum membrane |
| *SRP72* | *YPL210C* | *AFUA_4G10180* | Core component of the signal recognition particle ribonucleoprotein complex that functions in targeting nascent secretory proteins to the endoplasmic reticulum membrane |
| *SRS2* | *YJL092W* | *AFUA_2G03910* | DNA helicase and DNA-dependent ATPase involved in DNA repair, needed for proper timing of commitment to meiotic recombination and transition from Meiosis I to II; blocks trinucleotide repeat expansion; affects genome stability |
| *SRV2* | *YNL138W* | *AFUA_1G12760* | CAP subunit of adenylyl cyclase complex; N-terminus binds adenylyl cyclase and facilitates activation by RAS; C-terminus binds ADP-actin monomers, facilitating regulation of actin dynamics and cell morphogenesis |
| *SSA1* | *YLL024C YAL005C YBL075C YER103W* | *AFUA_1G07440* | ATPase involved in protein folding and the response to stress; plays a role in SRP-dependent cotranslational protein-membrane targeting and translocation; member of the heat shock protein 70 family; localized to the cytoplasm |
| *SSB1* | *YDL229W YNL209W* | *AFUA_8G03930* | Cytoplasmic ATPase that is a ribosome-associated molecular chaperone, functions with J-protein partner Zuo1p; may be involved in folding of newly-made polypeptide chains; member of the HSP70 family; interacts with phosphatase subunit Reg1p |
| *SSD1* | *YDR293C* | *AFUA_1G11420* | Protein with a role in maintenance of cellular integrity, interacts with components of the TOR pathway; ssd1 mutant of a clinical S. cerevisiae strain displays elevated virulence |
| *SSE1* | *YBR169C YPL106C* | *AFUA_1G12610* | ATPase that is a component of the heat shock protein Hsp90 chaperone complex; binds unfolded proteins; member of the heat shock protein 70 family; localized to the cytoplasm |
| *SSK1* | *YLR006C* | *AFUA_5G08390* | Cytoplasmic response regulator, part of a two-component signal transducer that mediates osmosensing via a phosphorelay mechanism; dephosphorylated form is degraded by the ubiquitin-proteasome system; potential Cdc28p substrate |
| *SSK2* | *YCR073C YNR031C* | *AFUA_1G10940* | MAP kinase kinase kinase of the HOG1 mitogen-activated signaling pathway; interacts with Ssk1p, leading to autophosphorylation and activation of Ssk2p which phosphorylates Pbs2p; also mediates actin cytoskeleton recovery from osmotic stress |
| *SSL2* | *YIL143C* | *AFUA_5G03320* | Component of the holoenzyme form of RNA polymerase transcription factor TFIIH, has DNA-dependent ATPase/helicase activity and is required, with Rad3p, for unwinding promoter DNA; involved in DNA repair; homolog of human ERCC3 |
| *SSN2* | *YDR443C* | *AFUA_2G04790* | Ssn2p |
| *SSN3* | *YPL042C* | *AFUA_3G13990* | Cyclin-dependent protein kinase, component of RNA polymerase II holoenzyme; involved in phosphorylation of the RNA polymerase II C-terminal domain; involved in glucose repression |
| *SSN8* | *YNL025C* | *AFUA_2G15790* | Cyclin-like component of the RNA polymerase II holoenzyme, involved in phosphorylation of the RNA polymerase II C-terminal domain; involved in glucose repression and telomere maintenance |
| *SSO1* | *YMR183C YPL232W* | *AFUA_7G01510* | Plasma membrane t-SNARE involved in fusion of secretory vesicles at the plasma membrane and in vesicle fusion during sporulation; forms a complex with Sec9p that binds v-SNARE Snc2p; syntaxin homolog; functionally redundant with Sso2p |
| *SSP120* | *YLR250W* | *AFUA_5G11640* | Protein of unknown function; green fluorescent protein (GFP)-fusion protein localizes to the cytoplasm in a punctate pattern |
| *SSS1* | *YDR086C* | *AFUA_3G06901* | Protein translocation complex subunit Sss1 |
| *SST2* | *YLR452C* | *AFUA_2G11180* | GTPase-activating protein for Gpa1p, regulates desensitization to alpha factor pheromone; also required to prevent receptor-independent signaling of the mating pathway; member of the RGS family |
| *SSU1* | *YPL092W* | *AFUA_4G09410 AFUA_3G14640 AFUA_1G13360 AFUA_7G01790* | C4-dicarboxylate transporter/malic acid transport protein |
| *SSU72* | *YNL222W* | *AFUA_2G03760* | Transcription/RNA-processing factor Ssu72 |
| *SSZ1* | *YHR064C* | *AFUA_2G02320* | Hsp70 protein that interacts with Zuo1p to form a ribosome-associated complex that binds the ribosome via the Zuo1p subunit; also involved in pleiotropic drug resistance via sequential activation of PDR1 and PDR5; binds ATP |
| *STB4* | *YMR019W* | *AFUA_4G01010* | Protein that binds Sin3p in a two-hybrid assay; contains a Zn(II)2Cys6 zinc finger domain characteristic of DNA-binding proteins; computational analysis suggests a role in regulation of expression of genes encoding transporters |
| *STE12* | *YHR084W* | *AFUA_5G06190* | Sexual development transcription factor SteA |
| *STE14* | *YDR410C* | *AFUA_2G08420* | Farnesyl cysteine-carboxyl methyltransferase, mediates the carboxyl methylation step during C-terminal CAAX motif processing of a-factor and RAS proteins in the endoplasmic reticulum, localizes to the ER membrane |
| *STE18* | *YJR086W* | *AFUA_1G05210* | G protein gamma subunit, forms a dimer with Ste4p to activate the mating signaling pathway, forms a heterotrimer with Gpa1p and Ste4p to dampen signaling; C-terminus is palmitoylated and farnesylated, which are required for normal signaling |
| *STE2* | *YFL026W* | *AFUA_3G14330* | Receptor for alpha-factor pheromone; seven transmembrane-domain GPCR that interacts with both pheromone and a heterotrimeric G protein to initiate the signaling response that leads to mating between haploid a and alpha cells |
| *STE20* | *YHL007C* | *AFUA_2G04680* | Cdc42p-activated signal transducing kinase of the PAK family; involved in pheromone response, pseudohyphal/invasive growth, vacuole inheritance, down-regulation of sterol uptake; GBB motif binds Ste4p |
| *STE24* | *YJR117W* | *AFUA_4G07590* | Highly conserved zinc metalloprotease that functions in two steps of a-factor maturation, C-terminal CAAX proteolysis and the first step of N-terminal proteolytic processing; contains multiple transmembrane spans |
| *STE3* | *YKL178C* | *AFUA_5G07880* | Receptor for a factor pheromone, transcribed in alpha cells and required for mating by alpha cells, couples to MAP kinase cascade to mediate pheromone response; ligand bound receptors are endocytosed and recycled to the plasma membrane; GPC |
| *STE4* | *YOR212W* | *AFUA_5G12210* | G protein beta subunit, forms a dimer with Ste18p to activate the mating signaling pathway, forms a heterotrimer with Gpa1p and Ste18p to dampen signaling; may recruit Rho1p to the polarized growth site during mating; contains WD40 repeats |
| *STE6* | *YKL209C* | *AFUA_4G08800* | Plasma membrane ATP-binding cassette transporter required for the export of a-factor, catalyzes ATP hydrolysis coupled to a-factor transport; contains 12 transmembrane domains and two ATP binding domains; expressed only in MATa cells |
| *STH1* | *YIL126W* | *AFUA_5G06330* | ATPase component of the RSC chromatin remodeling complex; required for expression of early meiotic genes; essential helicase-related protein homologous to Snf2p |
| *STI1* | *YOR027W* | *AFUA_7G01860* | Hsp90 cochaperone, interacts with the Ssa group of the cytosolic Hsp70 chaperones; activates the ATPase activity of Ssa1p; homolog of mammalian Hop protein |
| *STL1* | *YDR536W* | *AFUA_8G05710* | Glycerol proton symporter of the plasma membrane, subject to glucose-induced inactivation, strongly but transiently induced when cells are subjected to osmotic shock |
| *STO1* | *YMR125W* | *AFUA_6G05130* | Large subunit of the nuclear mRNA cap-binding protein complex, interacts with Npl3p to carry nuclear poly(A)+ mRNA to cytoplasm; also involved in nuclear mRNA degradation and telomere maintenance; orthologous to mammalian CBP80 |
| *STS1* | *YIR011C* | *AFUA_4G07170* | Protein that interacts with the karyopherin Srp1p; may have a role with Srp1p in ubiquitin-mediated protein degradation |
| *STT3* | *YGL022W* | *AFUA_8G04430* | Oligosaccharyl transferase subunit (Stt3) |
| *STU1* | *YBL034C* | *AFUA_1G12700* | Component of the mitotic spindle that binds to interpolar microtubules via its association with beta-tubulin (Tub2p); required for interpolar microtubules to provide an outward force on the spindle poles |
| *STU2* | *YLR045C* | *AFUA_6G12980* | Microtubule-associated protein of the XMAP215/Dis1 family; regulates microtubule dynamics during spindle orientation and metaphase chromosome alignment; interacts with spindle pole body component Spc72p |
| *SUA7* | *YPR086W* | *AFUA_3G10620* | Transcription initiation protein |
| *SUB1* | *YMR039C* | *AFUA_2G07460* | RNA polymerase II transcriptional coactivator |
| *SUC2* | *YIL162W* | *AFUA_2G01240* | Invertase, sucrose hydrolyzing enzyme; a secreted, glycosylated form is regulated by glucose repression, and an intracellular, nonglycosylated enzyme is produced constitutively |
| *SUI1* | *YNL244C* | *AFUA_3G06260* | Translation initiation factor SUI1 |
| *SUI2* | *YJR007W* | *AFUA_3G13480* | Alpha subunit of the translation initiation factor eIF2, involved in the identification of the start codon; phosphorylation of Ser51 is required for regulation of translation by inhibiting the exchange of GDP for GTP |
| *SUI3* | *YPL237W* | *AFUA_3G08600* | Beta subunit of the translation initiation factor eIF2, involved in the identification of the start codon; proposed to be involved in mRNA binding |
| *SUP35* | *YDR172W* | *AFUA_2G04820* | Translation release factor eRF3 |
| *SUP45* | *YBR143C* | *AFUA_5G05680* | Peptide chain release factor eRF/aRF, subunit 1 |
| *SUR2* | *YDR297W* | *AFUA_1G16850* | Sphinganine C4-hydroxylase, catalyses the conversion of sphinganine to phytosphingosine in sphingolipid biosyntheis |
| *SUR7* | *YML052W* | *AFUA_2G02310* | Plasma membrane protein that localizes to furrow-like invaginations (MCC patches); component of eisosomes; associated with endocytosis, along with Pil1p and Lsp1p; sporulation and plasma membrane sphingolipid content are altered in mutants |
| *SUV3* | *YPL029W* | *AFUA_5G10820* | ATP-dependent RNA helicase, component of the mitochondrial degradosome along with the RNase Dss1p; the degradosome associates with the ribosome and mediates turnover of aberrant or unprocessed RNAs |
| *SVF1* | *YDR346C* | *AFUA_5G11820* | Protein with a potential role in cell survival pathways, required for the diauxic growth shift; expression in mammalian cells increases survival under conditions inducing apoptosis |
| *SWA2* | *YDR320C* | *AFUA_5G09630* | Auxilin-like protein involved in vesicular transport; clathrin-binding protein required for uncoating of clathrin-coated vesicles |
| *SWC4* | *YGR002C* | *AFUA_4G07380* | Component of the Swr1p complex that incorporates Htz1p into chromatin; component of the NuA4 histone acetyltransferase complex |
| *SWC5* | *YBR231C* | *AFUA_1G16610* | Protein of unknown function, component of the SWR1 complex, which exchanges histone variant H2AZ for chromatin-bound histone H2A |
| *SWD2* | *YKL018W* | *AFUA_5G12370* | Subunit of the COMPASS complex, which methylates histone H3 on lys 4 and is involved in telomeric silencing; subunit of CPF (cleavage and polyadenylation factor), a complex involved in RNAP II transcription termination |
| *SWF1* | *YDR126W* | *AFUA_6G07570* | Palmitoyltransferase that acts on the SNAREs Snc1p, Syn8p, Tlg1p and likely on all SNAREs; member of a family of putative palmitoyltransferases containing an Asp-His-His-Cys-cysteine rich domain; may have a role in vacuole fusion |
| *SWI1* | *YPL016W* | *AFUA_3G11380* | Subunit of the SWI/SNF chromatin remodeling complex, which regulates transcription by remodeling chromosomes; required for transcription of many genes, including ADH1, ADH2, GAL1, HO, INO1 and SUC2; can form the prion (SWI+) |
| *SWI6* | *YLR182W* | *AFUA_7G05620* | APSES transcription factor (MbpA) |
| *SWP1* | *YMR149W* | *AFUA_4G08660* | Delta subunit of the oligosaccharyl transferase glycoprotein complex, which is required for N-linked glycosylation of proteins in the endoplasmic reticulum |
| *SWT21* | *YNL187W* | *AFUA_6G10740* | Protein involved in mRNA splicing; contains a consensus nuclear export signal sequence similar to the consensus sequence recognized by Crm1p; interacts genetically with Prp40p and Tgs1p; contains WD40 repeats |
| *SYF1* | *YDR416W* | *AFUA_5G11910* | Member of the NineTeen Complex that contains Prp19p and stabilizes U6 snRNA in catalytic forms of the spliceosome containing U2, U5, and U6 snRNAs; null mutant has splicing defect and arrests in G2/M; homologs in human and C. elegans |
| *SYG1* | *YIL047C* | *AFUA_5G09320* | Plasma membrane protein of unknown function; truncation and overexpression suppresses lethality of G-alpha protein deficiency |
| *SYM1* | *YLR251W* | *AFUA_5G01170 AFUA_7G01470* | Protein required for ethanol metabolism; induced by heat shock and localized to the inner mitochondrial membrane; homologous to mammalian peroxisomal membrane protein Mpv17 |
| *SYN8* | *YAL014C* | *AFUA_2G15820* | Endosomal SNARE related to mammalian syntaxin 8 |
| *SYP1* | *YCR030C* | *AFUA_1G13590* | Protein of unknown function that is involved in endocytic site formation; may regulate assembly and disassembly of the septin ring; colocalizes and interacts with septin subunits; potential role in actin cytoskeletal organization |
| *SYT1* | *YPR095C* | *AFUA_2G08780* | Guanine nucleotide exchange factor for Arf proteins; involved in vesicular transport; suppressor of ypt3 mutations; member of the Sec7-domain family |
| *TAD3* | *YLR316C* | *AFUA_1G07080* | Subunit of tRNA-specific adenosine-34 deaminase, forms a heterodimer with Tad2p that converts adenosine to inosine at the wobble position of several tRNAs |
| *TAE1* | *YBR261C* | *AFUA_5G01020* | Putative S-adenosylmethionine-dependent methyltransferase of the seven beta-strand family; has a role in protein synthesis; green fluorescent protein (GFP)-fusion protein localizes to the cytoplasm; YBR261C is not an essential gene |
| *TAF1* | *YGR274C* | *AFUA_6G08600* | Transcription factor TFIID complex 145 kDa subunit, putative |
| *TAF10* | *YDR167W* | *AFUA_5G11460* | TFIID and and SAGA complex TAF10 subunit |
| *TAF13* | *YML098W* | *AFUA_1G09350* | Transcription initiation factor TFIID subunit 13, putative |
| *TAF14* | *YPL129W* | *AFUA_5G12190* | Transcription initiation factor subunit (TAF30), putative |
| *TAF2* | *YCR042C* | *AFUA_8G04950* | Transcription initiation factor TFIID subunit TSM1/127kD |
| *TAF5* | *YBR198C* | *AFUA_1G03070* | Transcription initiation factor TFIID subunit |
| *TAF6* | *YGL112C* | *AFUA_5G03680* | Transcription initiation factor TFIID complex 60 kDa subunit |
| *TAF7* | *YMR227C* | *AFUA_2G14880* | Transcription initiation factor TFIID subunit 7, putative |
| *TAF9* | *YMR236W* | *AFUA_1G14600* | Transcription initiation factor TFIID, 31kD subunit, putative |
| *TAH1* | *YCR060W* | *AFUA_2G11040* | HSP90 cofactor; interacts with Hsp82p, Pih1p, Rvb1 and Rvb2, contains a single TPR domain with at least two TPR motifs |
| *TAM41* | *YGR046W* | *AFUA_1G14100* | Mitochondrial protein involved in protein import into the mitochondrial matrix; maintains the functional integrity of the TIM23 protein translocator complex; viability of null mutant is strain-dependent; mRNA is targeted to the bud |
| *TAN1* | *YGL232W* | *AFUA_3G11060* | Putative tRNA acetyltransferase, RNA-binding protein required for the formation of the modified nucleoside N(4)-acetylcytidine in serine and leucine tRNAs but not required for the same modification in 18S rRNA |
| *TAO3* | *YIL129C* | *AFUA_6G11010* | Component of the RAM signaling network that is involved in regulation of Ace2p activity and cellular morphogenesis, interacts with protein kinase Cbk1p and also with Kic1p |
| *TAZ1* | *YPR140W* | *AFUA_2G13960* | Lyso-phosphatidylcholine acyltransferase, required for normal phospholipid content of mitochondrial membranes; may remodel acyl groups of cardiolipin in the inner membrane; human ortholog tafazzin is implicated in Barth syndrome |
| *TBF1* | *YPL128C* | *AFUA_5G11470* | MYB DNA binding protein (Tbf1) |
| *TCB1* | *YOR086C YNL087W* | *AFUA_7G01840* | Bud-specific protein with a potential role in membrane trafficking; GFP-fusion protein migrates from the cell surface to intracellular vesicles near vacuole; contains 3 calcium and lipid binding domains; mRNA is targeted to the bud |
| *TCP1* | *YDR212W* | *AFUA_2G16020* | Alpha subunit of chaperonin-containing T-complex, which mediates protein folding in the cytosol; involved in actin cytoskeleton maintenance; overexpression in neurons suppresses formation of pathogenic conformations of huntingtin protein |
| *TDH1* | *YJL052W YGR192C YJR009C* | *AFUA_5G01030 AFUA_5G01970* | Glyceraldehyde-3-phosphate dehydrogenase, isozyme 1, involved in glycolysis and gluconeogenesis; tetramer that catalyzes the reaction of glyceraldehyde-3-phosphate to 1,3 bis-phosphoglycerate; detected in the cytoplasm and cell wall |
| *TDP1* | *YBR223C* | *AFUA_2G11070* | Tyrosyl-DNA phosphodiesterase |
| *TEC1* | *YBR083W* | *AFUA_1G04830* | Transcription factor AbaA |
| *TED1* | *YIL039W* | *AFUA_2G03370* | Conserved phosphoesterase domain-containing protein that acts together with Emp24p/Erv25p in cargo exit from the ER; deletion confers sensitivity to 4-(N-(S-glutathionylacetyl)amino) phenylarsenoxide (GSAO) |
| *TEF1* | *YBR118W YPR080W* | *AFUA_1G06390* | Translation elongation factor EF-1 alpha subunit , putative |
| *TEL1* | *YBL088C* | *AFUA_5G12660* | Protein kinase primarily involved in telomere length regulation; contributes to cell cycle checkpoint control in response to DNA damage; functionally redundant with Mec1p; homolog of human ataxia telangiectasia gene |
| *TEL2* | *YGR099W* | *AFUA_2G13740* | Essential DNA-binding protein specific to single-stranded yeast telomeric DNA repeats, required for telomere length regulation and telomere position effect |
| *TES1* | *YJR019C* | *AFUA_1G15170 AFUA_8G06680* | Peroxisomal acyl-CoA thioesterase likely to be involved in fatty acid oxidation rather than fatty acid synthesis; conserved protein also found in human peroxisomes; TES1 mRNA levels increase during growth on fatty acids |
| *TFA1* | *YKL028W* | *AFUA_2G08450* | TFIIE large subunit, involved in recruitment of RNA polymerase II to the promoter, activation of TFIIH, and promoter opening |
| *TFA2* | *YKR062W* | *AFUA_6G13130* | TFIIE small subunit, involved in RNA polymerase II transcription initiation |
| *TFB1* | *YDR311W* | *AFUA_4G11690* | Subunit of TFIIH and nucleotide excision repair factor 3 complexes, required for nucleotide excision repair, target for transcriptional activators |
| *TFB2* | *YPL122C* | *AFUA_4G04360* | Subunit of TFIIH and nucleotide excision repair factor 3 complexes, involved in transcription initiation, required for nucleotide excision repair, similar to 52 kDa subunit of human TFIIH |
| *TFB4* | *YPR056W* | *AFUA_5G11580* | Subunit of TFIIH complex, involved in transcription initiation, similar to 34 kDa subunit of human TFIIH; interacts with Ssl1p |
| *TFB5* | *YDR079C-A* | *AFUA_3G14550* | Component of the RNA polymerase II general transcription and DNA repair factor TFIIH; involved in transcription initiation and in nucleotide-excision repair; homolog of Chlamydomonas reinhardtii REX1-S protein involved in DNA repair |
| *TFC1* | *YBR123C* | *AFUA_5G02550* | RNA polymerase III transcription factor subunit, putative |
| *TFC3* | *YAL001C* | *AFUA_2G16462* | TFIIIC transcription initiation factor complex subunits Tfc3 |
| *TFC4* | *YGR047C* | *AFUA_1G04980* | RNA polymerase III transcription factor TFIIIC subunit (Tfc4) |
| *TFC6* | *YDR362C* | *AFUA_3G10380* | Transcription factor TFIIIC complex subunit Tfc6, putative |
| *TFG1* | *YGR186W* | *AFUA_3G04230* | TFIIF largest subunit; involved in both transcription initiation and elongation of RNA polymerase II; homologous to human RAP74 |
| *TFG2* | *YGR005C* | *AFUA_8G05490* | TFIIF middle subunit; involved in both transcription initiation and elongation of RNA polymerase II; homologous to human RAP30 |
| *TFP1* | *YDL185W* | *AFUA_5G02370* | Subunit A of the eight-subunit V1 peripheral membrane domain of the vacuolar H+-ATPase; protein precursor undergoes self-catalyzed splicing to yield the extein Tfp1p and the intein Vde (PI-SceI), which is a site-specific endonuclease |
| *TFP3* | *YPL234C* | *AFUA_5G08560* | Vacuolar ATPase proteolipid subunit c |
| *TGL4* | *YOR081C YKR089C* | *AFUA_4G08440* | Multifunctional triacylglycerol lipase, steryl ester hydrolase, and Ca2+-independent phospholipase A2; catalyzes acyl-CoA dependent acylation of LPA to PA; required with Tgl3p for timely bud formation; phosphorylated and activated by Cdc28p |
| *TGS1* | *YPL157W* | *AFUA_6G08610* | RNA methylase family protein |
| *THG1* | *YGR024C* | *AFUA_1G14630* | TRNAHis guanylyltransferase Thg1 |
| *THI2* | *YBR240C* | *AFUA_1G01850* | C6 finger domain protein |
| *THI74* | *YML018C YDR438W* | *AFUA_5G12140* | Putative protein of unknown function; green fluorescent protein (GFP)-fusion protein localizes to the membrane of the vacuole; physical interaction with Atg27p suggests a possible role in autophagy; YML018C is not an essential gene |
| *THR4* | *YCR053W* | *AFUA_3G08980* | Threonine synthase Thr4 |
| *THS1* | *YIL078W* | *AFUA_4G13700* | Threonyl-tRNA synthetase |
| *TID3* | *YIL144W* | *AFUA_3G10180* | Component of the evolutionarily conserved kinetochore-associated Ndc80 complex (Ndc80p-Nuf2p-Spc24p-Spc25p); conserved coiled-coil protein involved in chromosome segregation, spindle checkpoint activity, kinetochore assembly and clustering |
| *TIF1* | *YJL138C YKR059W* | *AFUA_3G08160* | Eukaryotic translation initiation factor 4 |
| *TIF11* | *YMR260C* | *AFUA_6G02520* | Eukaryotic translation initiation factor eIF-1A subunit |
| *TIF3* | *YPR163C* | *AFUA_2G16400* | Translation initiation factor 4B |
| *TIF34* | *YMR146C* | *AFUA_3G08640* | Eukaryotic translation initiation factor 3 subunit 2i, putative |
| *TIF35* | *YDR429C* | *AFUA_2G09870* | Eukaryotic translation initiation factor 3 subunit EifCg |
| *TIF4631* | *YGL049C YGR162W* | *AFUA_2G09490* | Eukaryotic translation initiation factor subunit eIF-4F |
| *TIF5* | *YPR041W* | *AFUA_2G08990* | Eukaryotic translation initiation factor 5 |
| *TIF6* | *YPR016C* | *AFUA_5G06010* | Constituent of 66S pre-ribosomal particles, has similarity to human translation initiation factor 6 (eIF6); may be involved in the biogenesis and or stability of 60S ribosomal subunits |
| *TIM10* | *YHR005C-A* | *AFUA_1G04470* | Essential protein of the mitochondrial intermembrane space, forms a complex with Tim9p that delivers hydrophobic proteins to the TIM22 complex for insertion into the inner membrane |
| *TIM13* | *YGR181W* | *AFUA_2G12190* | Mitochondrial intermembrane space protein, forms a complex with Tim8p that delivers a subset of hydrophobic proteins to the TIM22 complex for insertion into the inner membrane |
| *TIM17* | *YJL143W* | *AFUA_2G04270* | Essential subunit of the Translocase of the Inner Mitochondrial membrane (TIM23 complex); with Tim23p, contributes to the architecture and function of the import channel; may link the import motor to the core TIM23 complex |
| *TIM21* | *YGR033C* | *AFUA_2G09820* | Nonessential subunit of the Translocase of the Inner Mitochondrial membrane and with respiratory enzymes; may regulate TIM23 complex activity |
| *TIM22* | *YDL217C* | *AFUA_5G02200* | Essential core component of the mitochondrial TIM22 complex involved in insertion of polytopic proteins into the inner membrane; forms the channel through which proteins are imported |
| *TIM23* | *YNR017W* | *AFUA_2G14190* | Essential component of the Translocase of the Inner Mitochondrial membrane (TIM23 complex); involved in protein import into mitochondrial matrix and inner membrane; with Tim17p, contributes to architecture and function of the import channel |
| *TIM44* | *YIL022W* | *AFUA_1G09840* | Essential component of the Translocase of the Inner Mitochondrial membrane to the translocation channel (Tim23p-Tim17p core complex) |
| *TIM50* | *YPL063W* | *AFUA_2G03420* | Essential component of the Translocase of the Inner Mitochondrial membrane (TIM23 complex); acts as receptor for the TIM23 complex guiding incoming precursors from the TOM complex; may control the gating of the Tim23p-Tim17p channel |
| *TIM54* | *YJL054W* | *AFUA_4G11900* | Component of the mitochondrial TIM22 complex involved in insertion of polytopic proteins into the inner membrane |
| *TIM9* | *YEL020W-A* | *AFUA_2G01050* | Essential protein of the mitochondrial intermembrane space, forms a complex with Tim10p that delivers hydrophobic proteins to the TIM22 complex for insertion into the inner membrane |
| *TIP20* | *YGL145W* | *AFUA_3G11130* | Peripheral membrane protein required for fusion of COPI vesicles with the ER; prohibits back-fusion of COPII vesicles with the ER; forms a tethering complex with Sec39p and Dsl1p that interacts with ER SNAREs Sec20p and Use1p |
| *TIP41* | *YPR040W* | *AFUA_2G07540* | Protein that interacts physically and genetically with Tap42p, which regulates protein phosphatase 2A; component of the TOR signaling pathway |
| *TKL1* | *YBR117C YPR074C* | *AFUA_1G13500* | Transketolase TktA |
| *TLG1* | *YDR468C* | *AFUA_5G02900* | Essential t-SNARE that forms a complex with Tlg2p and Vti1p and mediates fusion of endosome-derived vesicles with the late Golgi; binds the docking complex VFT through interaction with Vps51p |
| *TMA16* | *YOR252W* | *AFUA_3G09040* | Protein of unknown function that associates with ribosomes |
| *TMA17* | *YDL110C* | *AFUA_1G09160* | Protein of unknown function that associates with ribosomes; heterozygous deletion demonstrated increases in chromosome instability in a rad9 deletion background; protein abundance is decreased upon intracellular iron depletion |
| *TMA19* | *YKL056C* | *AFUA_1G16840* | Protein that associates with ribosomes; homolog of translationally controlled tumor protein; green fluorescent protein (GFP)-fusion protein localizes to the cytoplasm and relocates to the mitochondrial outer surface upon oxidative stress |
| *TMA20* | *YER007C-A* | *AFUA_1G09810* | Protein of unknown function that associates with ribosomes and has a putative RNA binding domain; interacts with Tma22p; null mutant exhibits translation defects; has homology to human oncogene MCT-1 |
| *TMA22* | *YJR014W* | *AFUA_8G02730* | Protein of unknown function; associates with ribosomes and has a putative RNA binding domain; interacts with Tma20p; similar to human GRAP and human DRP1, which interacts with human Tma20p homolog MCT-1 |
| *TMA23* | *YMR269W* | *AFUA_1G14640* | Nucleolar protein of unknown function implicated in ribosome biogenesis; TMA23 may be a fungal-specific gene as no homologs have been yet identified in higher eukaryotes |
| *TMA29* | *YMR226C* | *AFUA_5G11240* | NADP(+)-dependent dehydrogenase; acts on serine, L-allo-threonine, and other 3-hydroxy acids; green fluorescent protein fusion protein localizes to the cytoplasm and nucleus; may interact with ribosomes, based on co-purification experiments |
| *TMA46* | *YOR091W* | *AFUA_5G13930* | Protein of unknown function that associates with ribosomes; interacts with GTPase Rbg1p |
| *TMS1* | *YDR105C* | *AFUA_2G13400* | Membrane protein TMS1 |
| *TOA1* | *YOR194C* | *AFUA_1G14740* | TFIIA large subunit; involved in transcriptional activation, acts as antirepressor or as coactivator; homologous to largest and second largest subunits of human and Drosophila TFIIA |
| *TOA2* | *YKL058W* | *AFUA_2G15680* | TFIIA small subunit; involved in transcriptional activation, acts as antirepressor or as coactivator; homologous to smallest subunit of human and Drosophila TFIIA |
| *TOF1* | *YNL273W* | *AFUA_4G04390* | Subunit of a replication-pausing checkpoint complex that acts at the stalled replication fork to promote sister chromatid cohesion after DNA damage, facilitating gap repair of damaged DNA; interacts with the MCM helicase |
| *TOK1* | *YJL093C* | *AFUA_1G14250 AFUA_3G07540* | Outward-rectifier potassium channel of the plasma membrane with two pore domains in tandem, each of which forms a functional channel permeable to potassium; carboxy tail functions to prevent inner gate closures; target of K1 toxin |
| *TOM20* | *YGR082W* | *AFUA_6G11380* | Component of the TOM complex responsible for recognition and initial import steps for all mitochondrially directed proteins; acts as a receptor for incoming precursor proteins |
| *TOM22* | *YNL131W* | *AFUA_1G11860* | Component of the TOM complex responsible for initial import of mitochondrially directed proteins; acts as a receptor for precursor proteins and mediates interaction between TOM and TIM complexes |
| *TOM40* | *YMR203W* | *AFUA_6G05110* | Component of the TOM complex responsible for recognition and initial import steps for all mitochondrially directed proteins; constitutes the core element of the protein conducting pore |
| *TOM70* | *YHR117W YNL121C* | *AFUA_2G01660* | Mitochondrial outer membrane protein with similarity to Tom70p; probable minor component of the TOM complex responsible for recognition and import of mitochondrially directed proteins |
| *TOP1* | *YOL006C* | *AFUA_1G03500* | Topoisomerase I |
| *TOP2* | *YNL088W* | *AFUA_6G13690* | Essential type II topoisomerase, relieves torsional strain in DNA by cleaving and re-sealing the phosphodiester backbone of both positively and negatively supercoiled DNA; cleaves complementary strands; localizes to axial cores in meiosis |
| *TOP3* | *YLR234W* | *AFUA_2G02660* | DNA Topoisomerase III, conserved protein that functions in a complex with Sgs1p and Rmi1p to relax single-stranded negatively-supercoiled DNA preferentially, involved in telomere stability and regulation of mitotic recombination |
| *TPA1* | *YER049W* | *AFUA_2G14810* | Poly(rA)-binding protein involved in translation termination efficiency, mRNA poly(A) tail length and mRNA stability; interacts with Sup45p and Pab1p; similar to prolyl 4-hydroxylases; binds Fe(III) and 2-oxoglutarate |
| *TPI1* | *YDR050C* | *AFUA_5G13450* | Triosephosphate isomerase |
| *TPK1* | *YPL203W YJL164C YKL166C* | *AFUA_2G12200* | CAMP-dependent protein kinase catalytic subunit PkaC1 |
| *TPM1* | *YIL138C YNL079C* | *AFUA_7G04210* | Tropomyosin |
| *TPO1* | *YLL028W* | *AFUA_2G12500 AFUA_5G01350 AFUA_5G09780 AFUA_4G06050 AFUA_5G01630* | MFS multidrug transporter |
| *TPO2* | *YGR138C YPR156C* | *AFUA_4G01140* | Polyamine transport protein specific for spermine; localizes to the plasma membrane; transcription of TPO2 is regulated by Haa1p; member of the major facilitator superfamily |
| *TPP1* | *YMR156C* | *AFUA_6G06820* | DNA 3'-phosphatase that functions in repair of endogenous damage of double-stranded DNA, activity is specific for removal of 3' phosphates at strand breaks; has similarity to the l-2-haloacid dehalogenase superfamily |
| *TPS1* | *YBR126C* | *AFUA_2G04010 AFUA_6G12950* | Synthase subunit of trehalose-6-phosphate synthase/phosphatase complex, which synthesizes the storage carbohydrate trehalose; also found in a monomeric form; expression is induced by the stress response and repressed by the Ras-cAMP pathway |
| *TPS2* | *YDR074W* | *AFUA_3G05650* | Phosphatase subunit of the trehalose-6-phosphate synthase/phosphatase complex, which synthesizes the storage carbohydrate trehalose; expression is induced by stress conditions and repressed by the Ras-cAMP pathway |
| *TPS3* | *YML100W YMR261C* | *AFUA_2G04020 AFUA_7G03940* | Alpha,alpha-trehalose phosphate synthase subunit, putative |
| *TRA1* | *YHR099W* | *AFUA_5G02570* | Subunit of SAGA and NuA4 histone acetyltransferase complexes; interacts with acidic activators which leads to transcription activation; similar to human TRRAP, which is a cofactor for c-Myc mediated oncogenic transformation |
| *TRI1* | *YMR233W YOR295W* | *AFUA_1G02020* | Subunit of UAF (upstream activation factor), which is an RNA polymerase I specific transcription stimulatory factor composed of Uaf30p, Rrn5p, Rrn9p, Rrn10p, histones H3 and H4; deletion decreases cellular growth rate |
| *TRK1* | *YJL129C YKR050W* | *AFUA_5G02290* | Component of the Trk1p-Trk2p potassium transport system; 180 kDa high affinity potassium transporter; phosphorylated in vivo and interacts physically with the phosphatase Ppz1p, suggesting Trk1p acitivy is regulated by phosphorylation |
| *TRL1* | *YJL087C* | *AFUA_1G09690* | TRNA ligase |
| *TRM1* | *YDR120C* | *AFUA_3G04200* | N2,N2-dimethylguanosine tRNA methyltransferase |
| *TRM10* | *YOL093W* | *AFUA_3G08760* | TRNA m(1)G methyltransferase domain containing protein |
| *TRM11* | *YOL124C* | *AFUA_4G12140* | Catalytic subunit of an adoMet-dependent tRNA methyltransferase complex in tRNAs; contains a THUMP domain and a methyltransferase domain |
| *TRM112* | *YNR046W* | *AFUA_6G06330* | Subunit of tRNA methyltransferase complexes in combination with Trm9p and Trm11p; subunit of complex with Mtq2p that methylates Sup45p in the ternary complex eRF1-eRF3-GTP; deletion confers resistance to zymocin |
| *TRM12* | *YML005W* | *AFUA_5G11650* | S-adenosylmethionine-dependent methyltransferase of the seven beta-strand family; required for wybutosine formation in phenylalanine-accepting tRNA |
| *TRM2* | *YKR056W* | *AFUA_5G11550* | TRNA(m5U54)methyltransferase |
| *TRM44* | *YPL030W* | *AFUA_6G02570* | Trm44p |
| *TRM8* | *YDL201W* | *AFUA_4G12280* | Subunit of a tRNA methyltransferase complex composed of Trm8p and Trm82p that catalyzes 7-methylguanosine modification of tRNA |
| *TRM82* | *YDR165W* | *AFUA_1G05070* | Subunit of a tRNA methyltransferase complex composed of Trm8p and Trm82p that catalyzes 7-methylguanosine modification of tRNA |
| *TRM9* | *YML014W* | *AFUA_1G07570* | Trm9p |
| *TRP1* | *YDR007W* | *AFUA_1G13090* | Phosphoribosylanthranilate isomerase that catalyzes the third step in tryptophan biosynthesis; in 2004, the sequence of TRP1 from strain S228C was updated by changing the previously annotated internal STOP to serine (TCA) |
| *TRP2* | *YER090W* | *AFUA_6G12580* | Anthranilate synthase, catalyzes the initial step of tryptophan biosynthesis, forms multifunctional hetero-oligomeric anthranilate synthase:indole-3-glycerol phosphate synthase enzyme complex with Trp3p |
| *TRP3* | *YKL211C* | *AFUA_1G13090* | Bifunctional enzyme exhibiting both indole-3-glycerol-phosphate synthase and anthranilate synthase activities, forms multifunctional hetero-oligomeric anthranilate synthase:indole-3-glycerol phosphate synthase enzyme complex with Trp2p |
| *TRP4* | *YDR354W* | *AFUA_4G11980* | Anthranilate phosphoribosyl transferase of the tryptophan biosynthetic pathway, catalyzes the phosphoribosylation of anthranilate, subject to the general control system of amino acid biosynthesis |
| *TRP5* | *YGL026C* | *AFUA_2G13250* | Bifunctional tryptophan synthase TRPB |
| *TRS120* | *YDR407C* | *AFUA_6G04840* | One of 10 subunits of the transport protein particle complex of the cis-Golgi which mediates vesicle docking and fusion; involved in endoplasmic reticulum to Golgi membrane traffic |
| *TRS130* | *YMR218C* | *AFUA_1G12540* | One of 10 subunits of the transport protein particle complex of the cis-Golgi which mediates vesicle docking and fusion; involved in ER to Golgi membrane traffic; mutation activates transcription of OCH1 |
| *TRS20* | *YBR254C* | *AFUA_2G08720* | One of 10 subunits of the transport protein particle complex of the cis-Golgi which mediates vesicle docking and fusion; mutations in the human homolog cause the spondyloepiphyseal dysplasia tarda disorder |
| *TRS23* | *YDR246W* | *AFUA_2G15080* | One of 10 subunits of the transport protein particle complex of the cis-Golgi which mediates vesicle docking and fusion; involved in endoplasmic reticulum to Golgi membrane traffic; human homolog is TRAPPC4 |
| *TRS31* | *YDR472W* | *AFUA_5G12880* | One of 10 subunits of the transport protein particle complex of the cis-Golgi which mediates vesicle docking and fusion; involved in endoplasmic reticulum to Golgi membrane traffic |
| *TRS33* | *YOR115C* | *AFUA_6G05220* | One of 10 subunits of the transport protein particle complex of the cis-Golgi which mediates vesicle docking and fusion; involved in endoplasmic reticulum to Golgi membrane traffic |
| *TRZ1* | *YKR079C* | *AFUA_1G16470* | TRNA processing endoribonuclease Trz1 |
| *TSC10* | *YBR265W* | *AFUA_1G11150* | 3-ketosphinganine reductase, catalyzes the second step in phytosphingosine synthesis, essential for growth in the absence of exogenous dihydrosphingosine or phytosphingosine, member of short chain dehydrogenase/reductase protein family |
| *TSC11* | *YER093C* | *AFUA_2G10460* | Subunit of TORC2 (Tor2p-Lst8p-Avo1-Avo2-Tsc11p-Bit61p), a membrane-associated complex that regulates actin cytoskeletal dynamics during polarized growth and cell wall integrity; involved in sphingolipid metabolism; contains a RasGEFN domain |
| *TSC13* | *YDL015C* | *AFUA_4G10760* | Enoyl reductase that catalyzes the last step in each cycle of very long chain fatty acid elongation, localizes to the ER, highly enriched in a structure marking nuclear-vacuolar junctions, coimmunoprecipitates with elongases Fen1p and Sur4p |
| *TSR1* | *YDL060W* | *AFUA_2G12880* | Protein required for processing of 20S pre-rRNA in the cytoplasm, associates with pre-40S ribosomal particles |
| *TSR2* | *YLR435W* | *AFUA_2G04800* | Protein with a potential role in pre-rRNA processing |
| *TSR3* | *YOR006C* | *AFUA_1G06690* | Protein required for correct processing of the 20S pre-rRNA at site D to generate mature 18S rRNA; green fluorescent protein (GFP)-fusion protein localizes to both the cytoplasm and the nucleus |
| *TSR4* | *YOL022C* | *AFUA_3G09340* | Cytoplasmic protein required for correct processing of the 20S pre-rRNA at site D to generate mature 18S rRNA; essential gene in S288C background but not in CEN.PK2 |
| *TTI1* | *YKL033W* | *AFUA_2G09000* | Putative protein of unknown function; subunit of the ASTRA complex which is part of the chromatin remodeling machinery; similar to S. pombe Tti1p; detected in highly purified mitochondria in high-throughput studies |
| *TUB1* | *YML124C YML085C* | *AFUA_1G02550* | Alpha-tubulin; associates with beta-tubulin to form tubulin dimer, which polymerizes to form microtubules; expressed at lower level than Tub1p |
| *TUB2* | *YFL037W* | *AFUA_1G10910 AFUA_7G00250* | Beta-tubulin; associates with alpha-tubulin to form tubulin dimer, which polymerizes to form microtubules |
| *TUB4* | *YLR212C* | *AFUA_1G13390* | Gamma-tubulin, involved in nucleating microtubules from both the cytoplasmic and nuclear faces of the spindle pole body |
| *TUF1* | *YOR187W* | *AFUA_1G12170* | Mitochondrial translation elongation factor Tu; comprises both GTPase and guanine nucleotide exchange factor activities, while these activities are found in separate proteins in S. pombe and humans |
| *TUL1* | *YKL034W* | *AFUA_1G12080* | Golgi-localized RING-finger ubiquitin ligase (E3), involved in ubiquitinating and sorting membrane proteins that contain polar transmembrane domains to multivesicular bodies for delivery to the vacuole for quality control purposes |
| *TUM1* | *YOR251C* | *AFUA_7G01370 AFUA_8G01800* | Mitochondrial protein, required for formation of the 2-thio group of the 5-methoxycarbonylmethyl-2-thiouridine modified base in some tRNAs; has similarity to mammalian thiosulfate sulfurtransferase (rhodanese) |
| *TUP1* | *YCR084C* | *AFUA_6G05150* | General repressor of transcription, forms complex with Cyc8p, involved in the establishment of repressive chromatin structure through interactions with histones H3 and H4, appears to enhance expression of some genes |
| *TUS1* | *YLR425W* | *AFUA_5G07430* | Guanine nucleotide exchange factor that functions to modulate Rho1p activity as part of the cell integrity signaling pathway; multicopy suppressor of tor2 mutation and ypk1 ypk2 double mutation; potential Cdc28p substrate |
| *TVP15* | *YDR100W* | *AFUA_5G11270* | Integral membrane protein localized to late Golgi vesicles along with the v-SNARE Tlg2p |
| *TVP18* | *YMR071C* | *AFUA_3G10600* | Integral membrane protein localized to late Golgi vesicles along with the v-SNARE Tlg2p; may interact with ribosomes, based on co-purification experiments |
| *TVP23* | *YDR084C* | *AFUA_4G13040* | Integral membrane protein localized to late Golgi vesicles along with the v-SNARE Tlg2p; green fluorescent protein (GFP)-fusion protein localizes to the cytoplasm in a punctate pattern |
| *TVP38* | *YKR088C* | *AFUA_4G13010* | Integral membrane protein localized to late Golgi vesicles along with the v-SNARE Tlg2p; required for asymmetric localization of Kar9p during mitosis; GFP-fusion protein localizes to the cytoplasm in a punctate pattern |
| *TWF1* | *YGR080W* | *AFUA_2G16920* | Actin monomer binding protein |
| *TYR1* | *YBR166C* | *AFUA_2G10450* | Prephenate dehydrogenase involved in tyrosine biosynthesis, expression is dependent on phenylalanine levels |
| *TYS1* | *YGR185C* | *AFUA_5G10640* | Cytoplasmic tyrosyl-tRNA synthetase, required for cytoplasmic protein synthesis; interacts with positions 34 and 35 of the tRNATyr anticodon; mutations in human ortholog YARS are associated with Charcot-Marie-Tooth neuropathies |
| *TYW1* | *YPL207W* | *AFUA_6G11510* | Protein required for the synthesis of wybutosine, a modified guanosine found at the 3'-position adjacent to the anticodon of phenylalanine tRNA which supports reading frame maintenance by stabilizing codon-anticodon interactions |
| *TYW3* | *YGL050W* | *AFUA_6G02350* | DUF207 domain protein |
| *UBA1* | *YKL210W* | *AFUA_2G15760* | Poly(A)+ RNA transport protein (UbaA) |
| *UBA2* | *YDR390C* | *AFUA_6G10510* | Nuclear protein that acts as a heterodimer with Aos1p to activate Smt3p before its conjugation to proteins (sumoylation), which may play a role in protein targeting; essential for viability |
| *UBC1* | *YDR177W* | *AFUA_5G07040* | Ubiquitin conjugating enzyme (UbcA) |
| *UBC4* | *YBR082C YDR059C* | *AFUA_3G06030* | Ubiquitin conjugating enzyme (UbcD) |
| *UBC6* | *YER100W* | *AFUA_4G04612* | Ubiquitin-conjugating enzyme Ubc6 |
| *UBC7* | *YMR022W* | *AFUA_5G04060* | Ubiquitin conjugating enzyme (UbcH) |
| *UBC9* | *YDL064W* | *AFUA_4G06830* | SUMO-conjugating enzyme involved in the Smt3p conjugation pathway; nuclear protein required for S- and M-phase cyclin degradation and mitotic control; involved in proteolysis mediated by the anaphase-promoting complex cyclosome (APCC) |
| *UBI4* | *YLL039C* | *AFUA_4G10350* | Polyubiquitin UbiD/Ubi4 |
| *UBP1* | *YDL122W* | *AFUA_5G13620* | Ubiquitin-specific protease that removes ubiquitin from ubiquitinated proteins; cleaves at the C terminus of ubiquitin fusions irrespective of their size; capable of cleaving polyubiquitin chains |
| *UBP15* | *YMR304W* | *AFUA_5G03250* | Ubiquitin-specific protease that may play a role in ubiquitin precursor processing |
| *UBP16* | *YPL072W* | *AFUA_3G11700* | Deubiquitinating enzyme anchored to the outer mitochondrial membrane, probably not important for general mitochondrial functioning, but may perform a more specialized function at mitochondria |
| *UBP2* | *YOR124C* | *AFUA_6G12270* | Ubiquitin-specific protease that removes ubiquitin from ubiquitinated proteins; interacts with Rsp5p and is required for MVB sorting of membrane proteins; can cleave polyubiquitin and has isopeptidase activity |
| *UBP3* | *YER151C* | *AFUA_1G07160* | Ubiquitin-specific protease that interacts with Bre5p to co-regulate anterograde and retrograde transport between endoplasmic reticulum and Golgi compartments; inhibitor of gene silencing; cleaves ubiquitin fusions but not polyubiquitin |
| *UBR1* | *YGR184C* | *AFUA_6G08420* | E3 ubiquitin ligase (N-recognin), forms heterodimer with Rad6p to ubiquitinate substrates in the N-end rule pathway; regulates peptide transport via Cup9p ubiquitination; mutation in human UBR1 causes Johansson-Blizzard Syndrome (JBS) |
| *UBX3* | *YDL091C* | *AFUA_1G14320* | UBX domain-containing protein that interacts with Cdc48p, green fluorescent protein (GFP)-fusion protein localizes to the cytoplasm in a punctate pattern |
| *UBX5* | *YDR330W* | *AFUA_7G04320* | UBX domain-containing protein that interacts with Cdc48p |
| *UBX6* | *YBR273C YJL048C* | *AFUA_3G06360* | UBX domain-containing protein that interacts with Cdc48p, transcription is repressed when cells are grown in media containing inositol and choline |
| *UFD4* | *YKL010C* | *AFUA_6G08880* | Ubiquitin-protein ligase that interacts with Rpt4p and Rpt6p, two subunits of the 19S particle of the 26S proteasome; cytoplasmic E3 involved in the degradation of ubiquitin fusion proteins |
| *UFE1* | *YOR075W* | *AFUA_2G09670* | SNARE protein (Ufe1) |
| *UGA4* | *YDL210W* | *AFUA_4G03370 AFUA_5G06230* | Permease that serves as a gamma-aminobutyrate transport protein involved in the utilization of GABA as a nitrogen source; catalyzes the transport of putrescine and delta-aminolevulinic acid (ALA); localized to the vacuolar membrane |
| *UGO1* | *YDR470C* | *AFUA_5G03910* | Protein of unknown function; outer membrane component of the mitochondrial fusion machinery; Ugo1p bind directly to Fzo1p and Mgm1p and thereby link these two GTPases during mitochondrial fusion |
| *UIP5* | *YKR044W* | *AFUA_2G12180* | Protein of unknown function that interacts with Ulp1p, a Ubl (ubiquitin-like protein)-specific protease for Smt3p protein conjugates |
| *ULP1* | *YPL020C* | *AFUA_5G14040* | Ubl (ubiquitin-like protein)-specific protease that cleaves Smt3p protein conjugates; specifically required for cell cycle progression; associates with nucleoporins and may interact with septin rings during telophase |
| *ULS1* | *YOR191W* | *AFUA_7G08480 AFUA_1G00630 AFUA_6G13290* | SWI/SNF family DNA-dependent ATPase |
| *UNG1* | *YML021C* | *AFUA_2G06140* | Uracil DNA N-glycosylase Ung1 |
| *URA2* | *YJL130C* | *AFUA_6G11310* | Bifunctional carbamoylphosphate synthetase (CPSase)-aspartate transcarbamylase (ATCase), catalyzes the first two enzymatic steps in the de novo biosynthesis of pyrimidines; both activities are subject to feedback inhibition by UTP |
| *URA6* | *YKL024C* | *AFUA_7G03990* | Uridylate kinase Ura6 |
| *URB1* | *YKL014C* | *AFUA_5G10840* | Nucleolar protein required for the normal accumulation of 25S and 5.8S rRNAs, associated with the 27SA2 pre-ribosomal particle; proposed to be involved in the biogenesis of the 60S ribosomal subunit |
| *URB2* | *YJR041C* | *AFUA_1G13200* | Nucleolar protein required for normal metabolism of the rRNA primary transcript, proposed to be involved in ribosome biogenesis |
| *URC2* | *YDR520C* | *AFUA_4G10120* | Putative Zn(II)2Cys6 motif containing transcription factor; non-essential gene identified in a screen for mutants with increased levels of rDNA transcription; similar to S. kluyveri Urc2p involved in uracil catabolism |
| *USO1* | *YDL058W* | *AFUA_1G13720* | Essential protein involved in the vesicle-mediated ER to Golgi transport step of secretion; binds membranes and functions during vesicle docking to the Golgi; required for assembly of the ER-to-Golgi SNARE complex |
| *UTP10* | *YJL109C* | *AFUA_6G13370* | Nucleolar protein, component of the small subunit processome containing the U3 snoRNA that is involved in processing of pre-18S rRNA |
| *UTP11* | *YKL099C* | *AFUA_1G14580* | Subunit of U3-containing Small Subunit processome complex involved in production of 18S rRNA and assembly of small ribosomal subunit |
| *UTP13* | *YLR222C* | *AFUA_4G07540* | Nucleolar protein, component of the small subunit processome containing the U3 snoRNA that is involved in processing of pre-18S rRNA |
| *UTP14* | *YML093W* | *AFUA_1G04240* | Subunit of U3-containing Small Subunit processome complex involved in production of 18S rRNA and assembly of small ribosomal subunit |
| *UTP15* | *YMR093W* | *AFUA_2G01780* | Nucleolar protein, component of the small subunit processome containing the U3 snoRNA that is involved in processing of pre-18S rRNA |
| *UTP18* | *YJL069C* | *AFUA_2G05930* | Possible U3 snoRNP protein involved in maturation of pre-18S rRNA, based on computational analysis of large-scale protein-protein interaction data |
| *UTP20* | *YBL004W* | *AFUA_6G08240* | Component of the small-subunit processome, which is involved in the biogenesis of the 18S rRNA |
| *UTP21* | *YLR409C* | *AFUA_3G04310* | Subunit of U3-containing 90S preribosome and Small Subunit processome complexes involved in production of 18S rRNA and assembly of small ribosomal subunit; synthetic defect with STI1 Hsp90 cochaperone; human homolog linked to glaucoma |
| *UTP22* | *YGR090W* | *AFUA_3G05490* | Possible U3 snoRNP protein involved in maturation of pre-18S rRNA, based on computational analysis of large-scale protein-protein interaction data |
| *UTP23* | *YOR004W* | *AFUA_1G02610* | Essential nucleolar protein that is a component of the SSU processome involved in 40S ribosomal subunit biogenesis; has homology to PINc domain protein Fcf1p, although the PINc domain of Utp23p is not required for function |
| *UTP4* | *YDR324C* | *AFUA_2G03930* | Subunit of U3-containing 90S preribosome and Small Subunit processome complexes involved in production of 18S rRNA and assembly of small ribosomal subunit; member of t-Utp subcomplex involved with transcription of 35S rRNA transcript |
| *UTP5* | *YDR398W* | *AFUA_4G10550* | Subunit of U3-containing Small Subunit processome complex involved in production of 18S rRNA and assembly of small ribosomal subunit |
| *UTP6* | *YDR449C* | *AFUA_3G09750* | Nucleolar protein, component of the small subunit processome containing the U3 snoRNA that is involved in processing of pre-18S rRNA |
| *UTP7* | *YER082C* | *AFUA_2G12890* | Nucleolar protein, component of the small subunit processome containing the U3 snoRNA that is involved in processing of pre-18S rRNA |
| *UTR1* | *YEL041W YJR049C* | *AFUA_2G01350 AFUA_5G12870* | ATP-NADH kinase; phosphorylates both NAD and NADH |
| *UTR2* | *YEL040W* | *AFUA_2G03120* | Chitin transglycosylase that functions in the transfer of chitin to beta(1-6) and beta(1-3) glucans in the cell wall; similar to and functionally redundant with Crh1; glycosylphosphatidylinositol (GPI)-anchored protein localized to bud neck |
| *VAC14* | *YLR386W* | *AFUA_6G12890* | Protein involved in regulated synthesis of PtdIns(3,5)P(2), in control of trafficking of some proteins to the vacuole lumen via the MVB, and in maintenance of vacuole size and acidity; interacts with Fig4p; activator of Fab1p |
| *VAC7* | *YNL054W* | *AFUA_2G08080* | Integral vacuolar membrane protein involved in vacuole inheritance and morphology; activates Fab1p kinase activity under basal conditions and also after hyperosmotic shock |
| *VAC8* | *YEL013W* | *AFUA_5G13540* | Phosphorylated and palmitoylated vacuolar membrane protein that interacts with Atg13p, required for the cytoplasm-to-vacuole targeting pathway; interacts with Nvj1p to form nucleus-vacuole junctions |
| *VAM6* | *YDL077C* | *AFUA_7G05790* | Vacuolar protein that plays a critical role in the tethering steps of vacuolar membrane fusion by facilitating guanine nucleotide exchange on small guanosine triphosphatase Ypt7p |
| *VAM7* | *YGL212W* | *AFUA_2G02610* | Component of the vacuole SNARE complex involved in vacuolar morphogenesis; SNAP-25 homolog; functions with a syntaxin homolog Vam3p in vacuolar protein trafficking |
| *VAS1* | *YGR094W* | *AFUA_8G04800* | Mitochondrial and cytoplasmic valyl-tRNA synthetase |
| *VBA2* | *YBR293W* | *AFUA_2G15140* | Permease of basic amino acids in the vacuolar membrane |
| *VCX1* | *YDL128W* | *AFUA_1G04270* | Vacuolar H+/Ca2+ exchanger involved in control of cytosolic Ca2+ concentration; has similarity to sodium/calcium exchangers, including the bovine Na+/Ca2+,K+ antiporter |
| *VID27* | *YNL212W* | *AFUA_3G08750* | Cytoplasmic protein of unknown function; possibly involved in vacuolar protein degradation; not essential for proteasome-dependent degradation of fructose-1,6-bisphosphatase (FBPase); null mutants exhibit normal growth |
| *VID30* | *YGL227W* | *AFUA_7G02110* | Protein involved in proteasome-dependent catabolite degradation of fructose-1,6-bisphosphatase (FBPase); binds FBPase; shifts the balance of nitrogen metabolism toward glutamate production; localizes to the nucleus and the cytoplasm |
| *VIP1* | *YLR410W* | *AFUA_3G04300* | Inositol hexakisphosphate and inositol heptakisphosphate kinase; generation of IP7 by Vip1p is important for phosphate signaling; likely involved in cortical actin cytoskeleton function, by analogy with S. pombe ortholog asp1 |
| *VMA13* | *YPR036W* | *AFUA_1G10670* | Subunit H of the eight-subunit V1 peripheral membrane domain of the vacuolar H+-ATPase (V-ATPase), an electrogenic proton pump found throughout the endomembrane system; serves as an activator or a structural stabilizer of the V-ATPase |
| *VMA2* | *YBR127C* | *AFUA_2G13240* | Subunit B of the eight-subunit V1 peripheral membrane domain of the vacuolar H+-ATPase (V-ATPase), an electrogenic proton pump found throughout the endomembrane system; contains nucleotide binding sites; also detected in the cytoplasm |
| *VMA21* | *YGR105W* | *AFUA_3G08410* | Integral membrane protein that is required for vacuolar H+-ATPase function, although not an actual component of the V-ATPase complex; functions in the assembly of the V-ATPase; localized to the yeast endoplasmic reticulum (ER) |
| *VMA4* | *YOR332W* | *AFUA_6G02090* | Subunit E of the eight-subunit V1 peripheral membrane domain of the vacuolar H+-ATPase (V-ATPase), an electrogenic proton pump found throughout the endomembrane system; required for the V1 domain to assemble onto the vacuolar membrane |
| *VMA5* | *YKL080W* | *AFUA_1G10810* | Subunit C of the eight-subunit V1 peripheral membrane domain of vacuolar H+-ATPase (V-ATPase), an electrogenic proton pump found throughout the endomembrane system; required for the V1 domain to assemble onto the vacuolar membrane |
| *VMA6* | *YLR447C* | *AFUA_3G13390* | Subunit d of the five-subunit V0 integral membrane domain of vacuolar H+-ATPase (V-ATPase), an electrogenic proton pump found in the endomembrane system; stabilizes VO subunits; required for V1 domain assembly on the vacuolar membrane |
| *VMA7* | *YGR020C* | *AFUA_2G11330* | Subunit F of the eight-subunit V1 peripheral membrane domain of vacuolar H+-ATPase (V-ATPase), an electrogenic proton pump found throughout the endomembrane system; required for the V1 domain to assemble onto the vacuolar membrane |
| *VMA8* | *YEL051W* | *AFUA_5G11920* | Subunit D of the eight-subunit V1 peripheral membrane domain of the vacuolar H+-ATPase (V-ATPase), an electrogenic proton pump found throughout the endomembrane system; plays a role in the coupling of proton transport and ATP hydrolysis |
| *VNX1* | *YNL321W* | *AFUA_4G04670* | Low affinity vacuolar membrane localized monovalent cation/H+ antiporter; member of the calcium exchanger family; potential Cdc28p substrate |
| *VPH1* | *YOR270C* | *AFUA_4G11300* | Subunit a of vacuolar-ATPase V0 domain, one of two isoforms (Vph1p and Stv1p); Vph1p is located in V-ATPase complexes of the vacuole while Stv1p is located in V-ATPase complexes of the Golgi and endosomes |
| *VPS1* | *YKR001C* | *AFUA_5G02360* | Dynamin-like GTPase required for vacuolar sorting; also involved in actin cytoskeleton organization, late Golgi-retention of some proteins, regulating peroxisome biogenesis |
| *VPS16* | *YPL045W* | *AFUA_5G13600* | Subunit of the vacuole fusion and protein sorting HOPS complex and the CORVET tethering complex; part of the Class C Vps complex essential for membrane docking and fusion at Golgi-to-endosome and endosome-to-vacuole protein transport stages |
| *VPS20* | *YMR077C* | *AFUA_1G09220* | Myristoylated subunit of ESCRTIII, the endosomal sorting complex required for transport of transmembrane proteins into the multivesicular body pathway to the lysosomal/vacuolar lumen; cytoplasmic protein recruited to endosomal membranes |
| *VPS24* | *YKL041W* | *AFUA_5G13890* | One of four subunits of the endosomal sorting complex required for transport III pathway |
| *VPS25* | *YJR102C* | *AFUA_4G12290* | Component of the ESCRT-II complex, which is involved in ubiquitin-dependent sorting of proteins into the endosome |
| *VPS27* | *YNR006W* | *AFUA_2G04740* | Endosomal protein that forms a complex with Hse1p; required for recycling Golgi proteins, forming lumenal membranes and sorting ubiquitinated proteins destined for degradation; has Ubiquitin Interaction Motifs which bind ubiquitin (Ubi4p) |
| *VPS28* | *YPL065W* | *AFUA_1G16320* | Component of the ESCRT-I complex (Stp22p, Srn2p, Vps28p, and Mvb12p), which is involved in ubiquitin-dependent sorting of proteins into the endosome; conserved C-terminal domain interacts with ESCRT-III subunit Vps20p |
| *VPS29* | *YHR012W* | *AFUA_1G09480* | Endosomal protein that is a subunit of the membrane-associated retromer complex essential for endosome-to-Golgi retrograde transport; forms a subcomplex with Vps35p and Vps26p that selects cargo proteins for endosome-to-Golgi retrieval |
| *VPS3* | *YDR495C* | *AFUA_1G14260* | Component of CORVET tethering complex; cytoplasmic protein required for the sorting and processing of soluble vacuolar proteins, acidification of the vacuolar lumen, and assembly of the vacuolar H+-ATPase |
| *VPS33* | *YLR396C* | *AFUA_2G13750* | ATP-binding protein that is a subunit of the HOPS complex and the CORVET tethering complex; essential for membrane docking and fusion at both the Golgi-to-endosome and endosome-to-vacuole stages of protein transport |
| *VPS35* | *YJL154C* | *AFUA_3G10360* | Endosomal subunit of membrane-associated retromer complex required for retrograde transport; receptor that recognizes retrieval signals on cargo proteins, forms subcomplex with Vps26p and Vps29p that selects cargo proteins for retrieval |
| *VPS4* | *YPR173C* | *AFUA_3G09360* | AAA-ATPase involved in multivesicular body protein sorting, ATP-bound Vps4p localizes to endosomes and catalyzes ESCRT-III disassembly and membrane release; ATPase activity is activated by Vta1p; regulates cellular sterol metabolism |
| *VPS41* | *YDR080W* | *AFUA_3G11200* | Vacuolar membrane protein that is a subunit of the homotypic vacuole fusion and vacuole protein sorting complex; essential for membrane docking and fusion at the Golgi-to-endosome and endosome-to-vacuole stages of protein transport |
| *VPS5* | *YOR069W* | *AFUA_4G12830* | Nexin-1 homolog required for localizing membrane proteins from a prevacuolar/late endosomal compartment back to the late Golgi apparatus; structural component of the retromer membrane coat complex; forms a retromer subcomplex with Vps17p |
| *VPS52* | *YDR484W* | *AFUA_1G04080* | Component of the GARP complex, Vps51p-Vps52p-Vps53p-Vps54p, which is required for the recycling of proteins from endosomes to the late Golgi; involved in localization of actin and chitin |
| *VPS53* | *YJL029C* | *AFUA_1G05100* | Component of the GARP complex, Vps51p-Vps52p-Vps53p-Vps54p, which is required for the recycling of proteins from endosomes to the late Golgi; required for vacuolar protein sorting |
| *VPS54* | *YDR027C* | *AFUA_5G02510* | Component of the GARP complex, Vps51p-Vps52p-Vps53p-Vps54p, which is required for the recycling of proteins from endosomes to the late Golgi; potentially phosphorylated by Cdc28p |
| *VPS55* | *YJR044C* | *AFUA_6G04780* | Late endosomal protein involved in late endosome to vacuole trafficking; functional homolog of human obesity receptor gene-related protein (OB-RGRP) |
| *VPS60* | *YDR486C* | *AFUA_4G11000* | Cytoplasmic and vacuolar membrane protein involved in late endosome to vacuole transport; required for normal filament maturation during pseudohyphal growth; may function in targeting cargo proteins for degradation; interacts with Vta1p |
| *VPS62* | *YGR141W YPR157W* | *AFUA_2G05980* | Putative protein of unknown function; induced by treatment with 8-methoxypsoralen and UVA irradiation |
| *VPS66* | *YPR139C* | *AFUA_2G14220* | Cytoplasmic protein of unknown function involved in vacuolar protein sorting |
| *VPS68* | *YOL129W* | *AFUA_6G04760* | Vacuolar membrane protein of unknown function involved in vacuolar protein sorting; also detected in the mitochondria |
| *VPS70* | *YJR126C* | *AFUA_1G03740* | Protein of unknown function involved in vacuolar protein sorting |
| *VPS71* | *YML041C* | *AFUA_2G05030* | Nucleosome-binding component of the SWR1 complex, which exchanges histone variant H2AZ for chromatin-bound histone H2A; required for vacuolar protein sorting |
| *VPS74* | *YDR372C* | *AFUA_1G06630* | Protein required for Golgi localization of glycosyltransferases; binds the cytosolic domains of Golgi glycosyltransferases; binding to PtdIns4P required for Golgi targeting and function; tetramer formation required for function |
| *VPS9* | *YML097C* | *AFUA_4G12490* | A guanine nucleotide exchange factor involved in vesicle-mediated vacuolar protein transport; specifically stimulates the intrinsic guanine nucleotide exchange activity of Vps21p/Rab5: similar to mammalian ras inhibitors; binds ubiquitin |
| *VRG4* | *YGL225W* | *AFUA_5G05740* | Golgi GDP-mannose transporter; regulates Golgi function and glycosylation in Golgi |
| *VTC1* | *YER072W* | *AFUA_1G09540* | Subunit of the vacuolar transporter chaperone complex involved in membrane trafficking, vacuolar polyphosphate accumulation, microautophagy and non-autophagic vacuolar fusion |
| *VTC2* | *YFL004W YPL019C* | *AFUA_1G07250* | Subunit of the vacuolar transporter chaperone complex involved in membrane trafficking, vacuolar polyphosphate accumulation, microautophagy and non-autophagic vacuolar fusion |
| *VTC4* | *YJL012C* | *AFUA_2G09040* | Vacuolar membrane polyphosphate polymerase; subunit of the vacuolar transporter chaperone complex involved in synthesis and transfer of polyP to the vacuole; regulates membrane trafficking; role in non-autophagic vacuolar fusion |
| *VTS1* | *YOR359W* | *AFUA_1G04990* | Post-transcriptional gene regulator, flap-structured DNA-binding and RNA-binding protein; shows genetic interactions with Vti1p, a v-SNARE involved in cis-Golgi membrane traffic; stimulates Dna2p endonuclease activity; contains a SAM domain |
| *WAR1* | *YML076C* | *AFUA_1G03780 AFUA_7G01640 AFUA_3G02700 AFUA_8G00950* | Homodimeric Zn2Cys6 zinc finger transcription factor; binds to a weak acid response element to induce transcription of PDR12 and FUN34, encoding an acid transporter and a putative ammonia transporter, respectively |
| *WBP1* | *YEL002C* | *AFUA_5G08970* | Beta subunit of the oligosaccharyl transferase glycoprotein complex; required for N-linked glycosylation of proteins in the endoplasmic reticulum |
| *WRS1* | *YOL097C* | *AFUA_2G01640* | Tryptophanyl-tRNA synthetase |
| *WSC4* | *YHL028W* | *AFUA_5G09020* | ER membrane protein involved in the translocation of soluble secretory proteins and insertion of membrane proteins into the ER membrane; may also have a role in the stress response but has only partial functional overlap with WSC1-3 |
| *WSS1* | *YHR134W* | *AFUA_1G02700* | Sumoylated protein of unknown function, identified based on genetic interactions with SMT3; UV-sensitive mutant phenotype and genetic interactions suggest a role in the DNA damage response, processing stalled or collapsed replication forks |
| *WWM1* | *YFL010C* | *AFUA_5G04290* | WW domain containing protein of unknown function; binds to Mca1p, a caspase-related protease that regulates H2O2-induced apoptosis; overexpression causes G1 phase growth arrest and clonal death that is suppressed by overexpression of MCA1 |
| *XPT1* | *YJR133W* | *AFUA_4G04550* | Xanthine-guanine phosphoribosyl transferase, required for xanthine utilization and for optimal utilization of guanine |
| *YAE1* | *YJR067C* | *AFUA_3G07330* | Protein of unknown function, essential for growth under standard conditions but not under anaerobic conditions |
| *YAH1* | *YPL252C* | *AFUA_5G03900* | Ferredoxin of the mitochondrial matrix required for formation of cellular iron-sulfur proteins; involved in heme A biosynthesis; homologous to human adrenodoxin |
| *YAK1* | *YJL141C* | *AFUA_4G03850* | Serine-threonine protein kinase that is part of a glucose-sensing system involved in growth control in response to glucose availability; translocates from the cytoplasm to the nucleus and phosphorylates Pop2p in response to a glucose signal |
| *YAP1801* | *YGR241C YHR161C* | *AFUA_2G04110* | Protein involved in clathrin cage assembly; binds Pan1p and clathrin; homologous to Yap1801p, member of the AP180 protein family |
| *YAP3* | *YHL009C* | *AFUA_2G02540* | Basic leucine zipper transcription factor |
| *YAT1* | *YAR035W* | *AFUA_1G12340* | Outer mitochondrial carnitine acetyltransferase, minor ethanol-inducible enzyme involved in transport of activated acyl groups from the cytoplasm into the mitochondrial matrix; phosphorylated |
| *YBL103C-A* | *YBL104C* | *AFUA_2G02880* | Putative protein of unknown function, promoter contains multiple GCN4 binding sites |
| *YBP1* | *YBR216C YGL060W* | *AFUA_2G16510* | Central kinetochore associated protein that mediates mitotic progression; interacts with several central kinetochore proteins and the centromeric histone Cse4p; role in resistance to oxidative stress; similarity to Ybp1p and Slk19p |
| *YBR075W* | *YBR074W* | *AFUA_1G05960* | Peptidase family M28 family |
| *YCF1* | *YDR135C* | *AFUA_5G07970* | Vacuolar glutathione S-conjugate transporter of the ATP-binding cassette family, has a role in detoxifying metals such as cadmium, mercury, and arsenite; also transports unconjugated bilirubin; similar to human cystic fibrosis protein CFTR |
| *YCG1* | *YDR325W* | *AFUA_5G02170* | Subunit of the condensin complex; required for establishment and maintenance of chromosome condensation, chromosome segregation and chromatin binding of the condensin complex; required for clustering of tRNA genes at the nucleolus |
| *YCH1* | *YGR203W* | *AFUA_2G09750* | Phosphatase with sequence similarity to Cdc25p, Arr2p and Mih1p; member of the single-domain rhodanese homology superfamily; green fluorescent protein (GFP)-fusion protein localizes to both the cytoplasm and the nucleus |
| *YCK1* | *YHR135C YNL154C* | *AFUA_6G06870* | Palmitoylated plasma membrane-bound casein kinase I isoform; shares redundant functions with Yck2p in morphogenesis, proper septin assembly, endocytic trafficking; provides an essential function overlapping with that of Yck2p |
| *YCL011C-A* | *YCL012C* | *AFUA_6G05190* | Putative protein of unknown function; orthologs are present in S. bayanus, S. paradoxus and Ashbya gossypii; YCL012C is not an essential gene |
| *YCR062W* | *YCR061W* | *AFUA_6G12730* | Protein of unknown function; green fluorescent protein (GFP)-fusion protein localizes to the cytoplasm in a punctate pattern; induced by treatment with 8-methoxypsoralen and UVA irradiation |
| *YCS4* | *YLR272C* | *AFUA_3G05530* | Subunit of the condensin complex; required for establishment and maintenance of chromosome condensation, chromosome segregation, chromatin binding of condensin, tRNA gene clustering at the nucleolus, and silencing at the mating type locus |
| *YDC1* | *YBR183W YPL087W* | *AFUA_4G12330* | Alkaline dihydroceramidase, involved in sphingolipid metabolism; preferentially hydrolyzes dihydroceramide to a free fatty acid and dihydrosphingosine; has a minor reverse activity |
| *YDJ1* | *YNL064C* | *AFUA_1G05040* | Protein chaperone involved in regulation of the HSP90 and HSP70 functions; involved in protein translocation across membranes; member of the DnaJ family |
| *YEA4* | *YEL004W* | *AFUA_8G02830* | Uridine diphosphate-N-acetylglucosamine transporter required for cell wall chitin synthesis; localized to the ER |
| *YEN1* | *YER041W* | *AFUA_3G13260* | Holliday junction resolvase; localization is cell-cycle dependent and regulated by Cdc28p phosphorylation; homolog of human GEN1 and has similarity to S. cerevisiae endonuclease Rth1p |
| *YET3* | *YDL072C* | *AFUA_1G14780* | Protein of unknown function; YET3 null mutant decreases the level of secreted invertase; homolog of human BAP31 protein |
| *YFH1* | *YDL120W* | *AFUA_4G10510* | Mitochondrial matrix iron chaperone; oxidizes and stores iron; interacts with Isu1p to promote Fe-S cluster assembly; mutation results in multiple Fe/S-dependent enzyme deficiencies; human frataxin homolog is mutated in Friedrich's ataxia |
| *YFH7* | *YFR007W* | *AFUA_2G14310 AFUA_4G06710* | Putative kinase with similarity to the phosphoribulokinase/uridine kinase/bacterial pantothenate kinase subfamily of P-loop kinases |
| *YHB1* | *YGR234W* | *AFUA_4G03410 AFUA_8G06080* | Nitric oxide oxidoreductase, flavohemoglobin involved in nitric oxide detoxification; plays a role in the oxidative and nitrosative stress responses |
| *YHC3* | *YJL059W* | *AFUA_2G12480* | Vacuolar membrane protein involved in the ATP-dependent transport of arginine into the vacuole and possibly in balancing ion homeostasis; homolog of human CLN3 involved in Batten disease (juvenile onset neuronal ceroid lipofuscinosis) |
| *YHI9* | *YHR029C* | *AFUA_4G04380* | Protein of unknown function; null mutant is defective in unfolded protein response; possibly involved in a membrane regulation metabolic pathway; member of the PhzF superfamily, though most likely not involved in phenazine production |
| *YHK8* | *YHR048W* | *AFUA_2G05840* | Presumed antiporter of the DHA1 family of multidrug resistance transporters; contains 12 predicted transmembrane spans; expression of gene is up-regulated in cells exhibiting reduced susceptibility to azoles |
| *YHM2* | *YMR241W* | *AFUA_5G04220* | Carrier protein that exports citrate from and imports oxoglutarate into the mitochondrion, causing net export of NADPH reducing equivalents; also associates with mt nucleoids and has a role in replication and segregation of the mt genome |
| *YIF1* | *YNL263C* | *AFUA_6G03840* | Integral membrane protein required for the fusion of ER-derived COPII transport vesicles with the Golgi; interacts with Yip1p and Yos1p; localizes to the Golgi, the ER, and COPII vesicles |
| *YIH1* | *YCR059C* | *AFUA_5G03000* | Protein that inhibits activation of Gcn2p, an eIF2 alpha subunit protein kinase, by competing for Gcn1p binding, thus impacting gene expression in response to starvation; has sequence and functional similarity to the mouse IMPACT gene |
| *YIP3* | *YNL044W* | *AFUA_6G06620* | Protein localized to COPII vesicles, proposed to be involved in ER to Golgi transport; interacts with members of the Rab GTPase family and Yip1p; also interacts with Rtn1p |
| *YIP4* | *YGL198W* | *AFUA_2G10800* | Protein that interacts with Rab GTPases, localized to late Golgi vesicles; computational analysis of large-scale protein-protein interaction data suggests a possible role in vesicle-mediated transport |
| *YIP5* | *YGL161C* | *AFUA_1G04020* | Protein that interacts with Rab GTPases, localized to late Golgi vesicles; computational analysis of large-scale protein-protein interaction data suggests a possible role in vesicle-mediated transport |
| *YJU2* | *YKL095W* | *AFUA_5G02580* | Essential protein required for pre-mRNA splicing; associates transiently with the spliceosomal NTC and acts after Prp2p to promote the first catalytic reaction of splicing |
| *YJU3* | *YKL094W* | *AFUA_3G14990* | Serine hydrolase with sequence similarity to monoglyceride lipase (MGL), localizes to lipid particles |
| *YKE2* | *YLR200W* | *AFUA_3G09350* | Subunit of the heterohexameric Gim/prefoldin protein complex involved in the folding of alpha-tubulin, beta-tubulin, and actin |
| *YKE4* | *YIL023C* | *AFUA_2G01460* | ZIP Zinc transporter, putaitve |
| *YKG9* | *YKL069W* | *AFUA_2G01360* | Methionine-R-sulfoxide reductase, reduces the R enantiomer of free Met-SO, in contrast to Ycl033Cp which reduces Met-R-SO in a peptide linkage; has a role in protection against oxidative stress |
| *YKT6* | *YKL196C* | *AFUA_4G09520* | Vesicle membrane protein with acyltransferase activity; involved in trafficking to and within the Golgi, endocytic trafficking to the vacuole, and vacuolar fusion; membrane localization due to prenylation at the carboxy-terminus |
| *YKU70* | *YMR284W* | *AFUA_5G07740* | Subunit of the telomeric Ku complex (Yku70p-Yku80p), involved in telomere length maintenance, structure and telomere position effect; relocates to sites of double-strand cleavage to promote nonhomologous end joining during DSB repair |
| *YKU80* | *YMR106C* | *AFUA_2G02620* | Subunit of the telomeric Ku complex (Yku70p-Yku80p), involved in telomere length maintenance, structure and telomere position effect; relocates to sites of double-strand cleavage to promote nonhomologous end joining during DSB repair |
| *YMC1* | *YBR104W YPR058W* | *AFUA_1G06530* | Mitochondrial protein, putative inner membrane transporter with a role in oleate metabolism and glutamate biosynthesis; member of the mitochondrial carrier family; has similarity with Ymc1p |
| *YMD8* | *YML038C* | *AFUA_6G08100* | Putative nucleotide sugar transporter, has similarity to Vrg4p |
| *YME1* | *YPR024W* | *AFUA_4G11530* | Catalytic subunit of the mitochondrial inner membrane i-AAA protease complex, which is responsible for degradation of unfolded or misfolded mitochondrial gene products; mutation causes an elevated rate of mitochondrial turnover |
| *YME2* | *YMR302C* | *AFUA_1G07350* | Integral inner mitochondrial membrane protein with a role in maintaining mitochondrial nucleoid structure and number; mutants exhibit an increased rate of mitochondrial DNA escape; shows some sequence similarity to exonucleases |
| *YML6* | *YML025C* | *AFUA_5G12800* | Mitochondrial ribosomal protein of the large subunit, has similarity to E. coli L4 ribosomal protein and human mitoribosomal MRP-L4 protein; essential for viability, unlike most other mitoribosomal proteins |
| *YMR1* | *YJR110W* | *AFUA_1G05640* | Phosphatidylinositol 3-phosphate phosphatase; involved in various protein sorting pathways, including CVT targeting and endosome to vacuole transport; has similarity to the conserved myotubularin dual specificity phosphatase family |
| *YNK1* | *YKL067W* | *AFUA_5G03490* | Nucleoside diphosphate kinase, catalyzes the transfer of gamma phosphates from nucleoside triphosphates, usually ATP, to nucleoside diphosphates by a mechanism that involves formation of an autophosphorylated enzyme intermediate |
| *YOR1* | *YGR281W* | *AFUA_4G09150* | Plasma membrane ATP-binding cassette transporter, multidrug transporter mediates export of many different organic anions including oligomycin; similar to human cystic fibrosis transmembrane receptor (CFTR) |
| *YOR29-03* | *YOR052C* | *AFUA_1G13400* | Nuclear protein of unknown function; expression induced by nitrogen limitation in a GLN3, GAT1-independent manner and by weak acid |
| *YOR29-10* | *YOR059C* | *AFUA_3G12320* | Lipase/serine esterase |
| *YOS9* | *YDR057W* | *AFUA_8G04530* | ER quality-control lectin; integral subunit of the HRD ligase; binds to glycans with terminal alpha-1,6 linked mannose on misfolded N-glycosylated proteins and participates in targeting proteins to ERAD; member of the OS-9 protein family |
| *YPR090W* | *YPR089W* | *AFUA_3G12450* | Protein of unknown function; exhibits genetic interaction with ERG11 and protein-protein interaction with Hsp82p |
| *YPT1* | *YFL038C* | *AFUA_5G04320* | Rab family small GTPase, involved in the ER-to-Golgi step of the secretory pathway; complex formation with the Rab escort protein Mrs6p is required for prenylation of Ypt1p by protein geranylgeranyltransferase type II (Bet2p-Bet4p) |
| *YPT6* | *YLR262C* | *AFUA_2G15570* | GTPase, Ras-like GTP binding protein involved in the secretory pathway, required for fusion of endosome-derived vesicles with the late Golgi, maturation of the vacuolar carboxypeptidase Y; has similarity to the human GTPase, Rab6 |
| *YRA1* | *YDR381W* | *AFUA_5G13860* | RNA binding protein required for export of poly(A)+ mRNA from the nucleus; proposed to couple mRNA export with 3' end processing via its interactions with Mex67p and Pcf11p; functionally redundant with Yra2p, another REF family member |
| *YRB1* | *YDR002W* | *AFUA_5G12180* | Ran GTPase binding protein; involved in nuclear protein import and RNA export, ubiquitin-mediated protein degradation during the cell cycle; shuttles between the nucleus and cytoplasm; is essential; homolog of human RanBP1 |
| *YRB2* | *YIL063C* | *AFUA_2G10810* | Protein of unknown function involved in nuclear processes of the Ran-GTPase cycle; involved in nuclear protein export; contains Ran Binding Domain and FxFG repeats; interacts with Srm1p, GTP-Gsp1p, Rna1p and Crm1p; is not essential |
| *YRB30* | *YGL164C* | *AFUA_2G04830* | RanGTP-binding protein, inhibits RanGAP1 (Rna1p)-mediated GTP hydrolysis of RanGTP (Gsp1p); shares similarity to proteins in other fungi but not in higher eukaryotes |
| *YSF3* | *YNL138W-A* | *AFUA_1G05700* | Component of the SF3b subcomplex of the U2 snRNP, essential protein required for for splicing and for assembly of SF3b |
| *YSH1* | *YLR277C* | *AFUA_1G16800* | Putative endoribonuclease, subunit of the mRNA cleavage and polyadenylation specificity complex; required for 3' processing, splicing, and transcriptional termination of mRNAs and snoRNAs |
| *YSP2* | *YHR080C YDR326C* | *AFUA_5G07100* | Protein of unknown function that may interact with ribosomes, based on co-purification experiments; the authentic, non-tagged protein is detected in highly purified mitochondria in high-throughput studies |
| *YTA12* | *YMR089C* | *AFUA_2G02680* | Component, with Afg3p, of the mitochondrial inner membrane m-AAA protease that mediates degradation of misfolded or unassembled proteins and is also required for correct assembly of mitochondrial enzyme complexes |
| *YTA7* | *YGR270W* | *AFUA_8G04270* | Protein that localizes to chromatin and has a role in regulation of histone gene expression; has a bromodomain-like region that interacts with the N-terminal tail of histone H3, and an ATPase domain; potentially phosphorylated by Cdc28p |
| *YTM1* | *YOR272W* | *AFUA_4G07630* | Constituent of 66S pre-ribosomal particles, forms a complex with Nop7p and Erb1p that is required for maturation of the large ribosomal subunit; has seven C-terminal WD repeats |
| *YUH1* | *YJR099W* | *AFUA_2G05590 AFUA_5G11950* | Ubiquitin C-terminal hydrolase that cleaves ubiquitin-protein fusions to generate monomeric ubiquitin; hydrolyzes the peptide bond at the C-terminus of ubiquitin; also the major processing enzyme for the ubiquitin-like protein Rub1p |
| *YVC1* | *YOR087W* | *AFUA_3G13490* | Vacuolar cation channel, mediates release of Ca(2+) from the vacuole in response to hyperosmotic shock |
| *ZAP1* | *YJL056C* | *AFUA_1G10080* | C2H2 transcription factor |
| *ZIM17* | *YNL310C* | *AFUA_6G08230* | Heat shock protein with a zinc finger motif; essential for protein import into mitochondria; may act with Pam18p to facilitate recognition and folding of imported proteins by Ssc1p in the mitochondrial matrix |
| *ZPR1* | *YGR211W* | *AFUA_6G10470* | Essential protein with two zinc fingers, present in the nucleus of growing cells but relocates to the cytoplasm in starved cells via a process mediated by Cpr1p; binds to translation elongation factor eEF-1 (Tef1p) |
| *ZPS1* | *YOL154W* | *AFUA_4G09580* | Putative GPI-anchored protein; transcription is induced under low-zinc conditions, as mediated by the Zap1p transcription factor, and at alkaline pH |
| *ZRG17* | *YNR039C* | *AFUA_1G12090* | Endoplasmic reticulum protein of unknown function, transcription is induced under conditions of zinc deficiency; mutant phenotype suggests a role in uptake of zinc |
| *ZRT1* | *YGL255W* | *AFUA_1G01550* | High-affinity zinc transporter of the plasma membrane, responsible for the majority of zinc uptake; transcription is induced under low-zinc conditions by the Zap1p transcription factor |
| *ZRT2* | *YLR130C* | *AFUA_2G03860* | Low-affinity zinc transporter of the plasma membrane; transcription is induced under low-zinc conditions by the Zap1p transcription factor |
| *ZRT3* | *YKL175W* | *AFUA_2G08740* | Vacuolar membrane zinc transporter, transports zinc from storage in the vacuole to the cytoplasm when needed; transcription is induced under conditions of zinc deficiency |
| *ZTA1* | *YBR046C* | *AFUA_5G11430* | NADPH-dependent quinone reductase, GFP-tagged protein localizes to the cytoplasm and nucleus; has similarity to E. coli quinone oxidoreductase and to human zeta-crystallin |
| *ZUO1* | *YGR285C* | *AFUA_4G03650* | Ribosome associated DnaJ chaperone Zuotin |
| *ZWF1* | *YNL241C* | *AFUA_3G08470* | Glucose-6-phosphate dehydrogenase (G6PD), catalyzes the first step of the pentose phosphate pathway; involved in adapting to oxidatve stress; homolog of the human G6PD which is deficient in patients with hemolytic anemia |
|  | *YKL171W* | *AFUA_1G11930* | Putative protein kinase; implicated in proteasome function; epitope-tagged protein localizes to the cytoplasm |
|  | *YPL236C* | *AFUA_8G04670* | Serine/threonine protein kinase |
|  | *YBR028C* | *AFUA_3G10080* | Putative protein kinase, possible substrate of cAMP-dependent protein kinase (PKA) |
|  | *YPL150W* | *AFUA_6G06720* | Putative protein kinase of unknown cellular role |
|  | *YOR378W* | *AFUA_5G09940* | Putative protein of unknown function; belongs to the DHA2 family of drug:H+ antiporters; YOR378W is not an essential gene |
|  | *YPL247C* | *AFUA_4G08530* | Putative protein of unknown function; green fluorescent protein (GFP)-fusion protein localizes to the cytoplasm and nucleus; similar to the petunia WD repeat protein an11; overexpression causes a cell cycle delay or arrest |
|  | *YOR246C* | *AFUA_2G04560 AFUA_6G02770* | Protein with similarity to oxidoreductases, found in lipid particles; required for replication of Brome mosaic virus in S. cerevisiae, a model system for studying replication of positive-strand RNA viruses in their natural hosts |
|  | *YLR426W YDL114W* | *AFUA_1G06280* | Putative protein of unknown function; the authentic, non-tagged protein is detected in highly purified mitochondria in high-throughput studies; proposed to be involved in resistance to mechlorethamine and streptozotocin |
|  | *YKL071W* | *AFUA_5G14120* | Putative protein of unknown function; expression induced in cells treated with the mycotoxin patulin, and also the quinone methide triterpene celastrol; green fluorescent protein (GFP)-fusion protein localizes to the cytoplasm |
|  | *YNL181W* | *AFUA_5G10790* | Oxidoreductase, short chain dehydrogenase/reductase family |
|  | *YNL134C YCR102C YLR460C* | *AFUA_6G03360 AFUA_1G17730 AFUA_6G10120 AFUA_8G01430* | Zinc-binding oxidoreductase ToxD |
|  | *YPR022C* | *AFUA_7G05960* | Putative protein of unknown function; green fluorescent protein (GFP)-fusion protein localizes to both the cytoplasm and the nucleus and is induced in response to the DNA-damaging agent MMS |
|  | *YPR127W* | *AFUA_1G10270* | Protein of unknown function, differentially expressed during alcoholic fermentation; expression activated by transcription factor YRM1/YOR172W; green fluorescent protein (GFP)-fusion protein localizes to both the cytoplasm and the nucleus |
|  | *YER152C* | *AFUA_1G14490 AFUA_6G00370* | Protein with 2-aminoadipate transaminase activity; shares amino acid similarity with the aminotransferases Aro8p and Aro9p; YER152C is not an essential gene |
|  | *YNL217W* | *AFUA_5G08620* | Ser/Thr protein phosphatase family |
|  | *YBR062C* | *AFUA_6G07590* | Ybr062cp |
|  | *YGR130C* | *AFUA_5G06740* | Putative protein of unknown function; GFP-fusion protein localizes to the cytoplasm; possible component of the eisosome; specifically phosphorylated in vitro by mammalian diphosphoinositol pentakisphosphate (IP7) |
|  | *YLR419W* | *AFUA_2G16140* | Putative helicase with limited sequence similarity to human Rb protein; the authentic, non-tagged protein is detected in highly purified mitochondria in high-throughput studies; YLR419W is not an essential gene |
|  | *YOL098C* | *AFUA_4G07610* | Zinc metalloprotease |
|  | *YGR210C* | *AFUA_1G13540* | Putative protein of unknown function; green fluorescent protein (GFP)-fusion protein localizes to the cytoplasm |
|  | *YMR315W* | *AFUA_2G16380* | Protein with NADP(H) oxidoreductase activity; transcription is regulated by Stb5p in response to NADPH depletion induced by diamide; promoter contains a putative Stb5p binding site |
|  | *YMR010W* | *AFUA_5G03510* | Putative protein of unknown function; green fluorescent protein (GFP)-fusion protein localizes to the cytoplasm; YMR010W is not an essential gene; YMR010W mRNA is transcribed with ADI1 |
|  | *YDR090C* | *AFUA_5G08410* | PQ loop repeat protein |
|  | *YJR129C* | *AFUA_7G02130* | Putative protein of unknown function; predicted S-adenosylmethionine-dependent methyltransferase of the seven beta-strand family; green fluorescent protein (GFP)-fusion protein localizes to the cytoplasm |
|  | *YNL024C* | *AFUA_4G05890* | Putative protein of unknown function with seven beta-strand methyltransferase motif; green fluorescent protein (GFP)-fusion protein localizes to the cytoplasm; YNL024C is not an essential gene |
|  | *YBR271W* | *AFUA_1G16310* | Putative S-adenosylmethionine-dependent methyltransferase of the seven beta-strand family; green fluorescent protein (GFP)-fusion protein localizes to the cytoplasm; predicted to be involved in ribosome biogenesis |
|  | *YNR064C* | *AFUA_4G07410 AFUA_3G08960 AFUA_4G03260* | Epoxide hydrolase |
|  | *YCR023C* | *AFUA_1G13970 AFUA_4G08740 AFUA_1G03730 AFUA_1G15490 AFUA_8G04150* | MFS multidrug transporter |
|  | *YKR017C* | *AFUA_6G12540* | Putative protein of unknown function; contains a RING finger motif |
|  | *YLR241W* | *AFUA_1G02130* | Putative protein of unknown function, may be involved in detoxification |
|  | *YDL144C* | *AFUA_3G00740* | Putative protein of unknown function; green fluorescent protein (GFP)-fusion protein localizes to the cytoplasm and nucleus; YDL144C is not an essential gene |
|  | *YOR292C* | *AFUA_1G10340* | Putative protein of unknown function; green fluorescent protein (GFP)-fusion protein localizes to the vacuole; YOR292C is not an essential gene |
|  | *YGL082W YPL191C* | *AFUA_5G01840* | Putative protein of unknown function; predicted prenylation/proteolysis target of Afc1p and Rce1p; green fluorescent protein (GFP)-fusion protein localizes to the cytoplasm and nucleus; YGL082W is not an essential gene |
|  | *YJL049W* | *AFUA_2G16692* | Putative protein of unknown function; YJL049W is a non-essential gene |
|  | *YMR253C YPL264C* | *AFUA_1G09310* | Putative protein of unknown function; green fluorescent protein (GFP)-fusion protein localizes to the cytoplasm in a punctate pattern; YMR253C is not an essential gene |
|  | *YLR143W* | *AFUA_4G09070* | Putative protein of unknown function; green fluorescent protein (GFP)-tagged protein localizes to the cytoplasm; YLR143W is not an essential gene |
|  | *YOR296W* | *AFUA_7G02350* | Putative protein of unknown function; green fluorescent protein (GFP)-fusion protein localizes to the cytoplasm; expressed during copper starvation; YOR296W is not an essential gene |
|  | *YGR125W* | *AFUA_4G12440* | Putative protein of unknown function; deletion mutant has decreased rapamycin resistance but normal wormannin resistance; green fluorescent protein (GFP)-fusion protein localizes to the vacuole |
|  | *YBL095W* | *AFUA_6G02390 AFUA_1G10800 AFUA_6G08890* | Putative protein of unknown function; the authentic, non-tagged protein is detected in highly purified mitochondria in high-throughput studies |
|  | *YLR253W* | *AFUA_4G06760* | Putative protein of unknown function; the authentic, non-tagged protein is detected in highly purified mitochondria in high-throughput studies; transcription is periodic during the metabolic cycle; YLR253W is not an essential gene |
|  | *YPL109C* | *AFUA_4G04590* | Putative protein of unknown function; the authentic, non-tagged protein is detected in highly purified mitochondria in high-throughput studies |
|  | *YPL260W* | *AFUA_1G06820* | Putative substrate of cAMP-dependent protein kinase (PKA); green fluorescent protein (GFP)-fusion protein localizes to the cytoplasm and nucleus; YPL260W is not an essential gene |
|  | *YLR352W* | *AFUA_4G11700* | Putative protein of unknown function with similarity to F-box proteins; interacts with Skp1p and Cdc53p; YLR352W is not an essential gene |
|  | *YDR306C* | *AFUA_2G02570* | F-box protein of unknown function; interacts with Sgt1p via a Leucine-Rich Repeat domain |
|  | *YBR287W* | *AFUA_1G15940* | Protein of unknown function; green fluorescent protein (GFP)-fusion protein localizes to the ER; YBR287W is not an essential gene |
|  | *YLL056C* | *AFUA_8G06600* | Putative protein of unknown function, transcription is activated by paralogous transcription factors Yrm1p and Yrr1p and genes involved in pleiotropic drug resistance (PDR); expression is induced in cells treated with the mycotoxin patulin |
|  | *YJL163C* | *AFUA_5G07760* | Tetracycline-efflux transporter |
|  | *YNL022C* | *AFUA_1G17090* | Putative cytosine 5-methyltransferase, contains seven beta-strand methyltransferase motif similar to NOP2/YNL061W; green fluorescent protein (GFP)-fusion protein localizes to the nucleus; predicted to be involved in ribosome biogenesis |
|  | *YDR124W* | *AFUA_5G07980 AFUA_5G14920 AFUA_3G08250 AFUA_6G13630* | Putative protein of unknown function; non-essential gene; expression is strongly induced by alpha factor |
|  | *YLR243W* | *AFUA_1G10640* | Putative protein of unknown function; YLR243W is an essential gene |
|  | *YOR262W* | *AFUA_6G10630* | Protein of unknown function required for establishment of sister chromatid cohesion; contains an ATP/GTP binding site motif; similar to YLR243W and is highly conserved across species and homologous to human gene GPN2/ATPBD1B |
|  | *YDR282C* | *AFUA_3G11210* | Putative protein of unknown function |
|  | *YGL242C* | *AFUA_1G06370* | Putative protein of unknown function; deletion mutant is viable |
|  | *YDR049W* | *AFUA_5G07960* | C2H2 finger and ankyrin domain protein |
|  | *YJR154W* | *AFUA_3G14710 AFUA_8G00230* | Putative protein of unknown function; green fluorescent protein (GFP)-fusion protein localizes to the cytoplasm |
|  | *YLR126C* | *AFUA_4G03700 AFUA_5G11090* | Putative protein of unknown function with similarity to glutamine amidotransferase proteins; has Aft1p-binding motif in the promoter; may be involved in copper and iron homeostasis; YLR126C is not an essential protein |
|  | *YGR093W* | *AFUA_6G08250* | Putative protein of unconfirmed function; green fluorescent protein (GFP)-fusion protein localizes to the nucleus |
|  | *YKL033W-A* | *AFUA_3G03460 AFUA_6G10760* | Putative protein of unknown function; similar to uncharacterized proteins from other fungi |
|  | *YNL035C* | *AFUA_1G14650* | Putative protein of unknown function with similarity to proteins containing WD-40 domains; green fluorescent protein (GFP)-fusion protein localizes to the nucleus; YNL035C is not an essential gene |
|  | *YDR336W* | *AFUA_6G13010* | Putative protein of unknown function; sumoylated under stress conditions in a genome wide study; YDR336W is not an essential gene |
|  | *YPL199C* | *AFUA_1G13150* | Putative protein of unknown function, predicted to be palmitoylated |
|  | *YIR042C* | *AFUA_7G00850* | Putative protein of unknown function; YIR042C is a non-essential gene |
|  | *YLL032C* | *AFUA_2G13490* | Protein of unknown function that may interact with ribosomes, based on co-purification experiments; green fluorescent protein (GFP)-fusion protein localizes to the cytoplasm; YLL032C is not an essential gene |
|  | *YMR196W* | *AFUA_2G12410* | Putative protein of unknown function; green fluorescent protein (GFP)-fusion protein localizes to the cytoplasm; YMR196W is not an essential gene |
|  | *YGR273C YMR295C* | *AFUA_3G08450 AFUA_8G02120* | Protein of unknown function that associates with ribosomes; green fluorescent protein (GFP)-fusion protein localizes to the cell periphery and bud; YMR295C is not an essential gene |
|  | *YHR097C YDR348C* | *AFUA_3G14020* | Protein of unknown function; green fluorescent protein (GFP)-fusion protein localizes to the cell periphery and bud neck; potential Cdc28p substrate |
|  | *YNL040W* | *AFUA_8G07110* | Putative protein of unknown function with strong similarity to alanyl-tRNA synthases from Eubacteria; green fluorescent protein (GFP)-fusion protein localizes to the cytoplasm; YNL040W is not an essential gene |
|  | *YMR034C* | *AFUA_6G11840* | Putative transporter, member of the SLC10 carrier family; identified in a transposon mutagenesis screen as a gene involved in azole resistance; YMR034C is not an essential gene |
|  | *YGR054W* | *AFUA_3G05970* | Eukaryotic initiation factor 2A; associates specifically with both 40S subunits and 80 S ribosomes, and interacts genetically with both eIF5b and eIF4E; homologous to mammalian eIF2A |
|  | *YLR281C* | *AFUA_6G04020* | Putative protein of unknown function; green fluorescent protein (GFP)-fusion protein localizes to mitochondria; YLR281C is not an essential gene |
|  | *YHR132W-A YNL157W* | *AFUA_1G08960* | Putative protein of unknown function; green fluorescent protein (GFP)-fusion protein localizes to the cytoplasm and nucleus; protein abundance is decreased upon intracellular iron depletion |
|  | *YNR029C* | *AFUA_2G11720* | Putative protein of unknown function, deletion confers reduced fitness in saline |
|  | *YEL023C* | *AFUA_2G14400 AFUA_3G12130* | Putative protein of unknown function; expression is increased greatly during sporulation; YEL023C is not an essential gene |
|  | *YMR155W* | *AFUA_2G13390* | Putative protein of unknown function; identified as interacting with Hsp82p in a high-throughput two-hybrid screen |
|  | *YGR031W* | *AFUA_1G02390 AFUA_8G07300* | Putative protein of unknown function; the authentic, non-tagged protein is detected in highly purified mitochondria in high-throughput studies |
|  | *YBR096W* | *AFUA_3G00570 AFUA_1G12200 AFUA_8G02315* | Putative protein of unknown function; green fluorescent protein (GFP)-fusion protein localizes to the ER |
|  | *YEL007W YHR177W* | *AFUA_3G09640 AFUA_6G04490* | Putative protein of unknown function; overexpression causes a cell cycle delay or arrest |
|  | *YNL010W* | *AFUA_4G12730 AFUA_7G06850* | Putative protein of unknown function with similarity to phosphoserine phosphatases; green fluorescent protein (GFP)-fusion protein localizes to the cytoplasm and nucleus; homozygous diploid mutant shows an increase in glycogen accumulation |
|  | *YGR266W* | *AFUA_1G09130 AFUA_4G12980* | Protein of unknown function, predicted to contain a single transmembrane domain; localized to both the mitochondrial outer membrane and the plasma membrane |
|  | *YMR210W* | *AFUA_6G04640* | Putative acyltransferase with similarity to Eeb1p and Eht1p, has a minor role in medium-chain fatty acid ethyl ester biosynthesis; may be involved in lipid metabolism and detoxification |
|  | *YKR070W* | *AFUA_3G12330 AFUA_4G11720* | Putative protein of unknown function; the authentic, non-tagged protein is detected in highly purified mitochondria in high-throughput studies |
|  | *YDR338C* | *AFUA_6G11140* | MATE efflux family protein subfamily |
|  | *YGL085W* | *AFUA_1G03430* | Putative protein of unknown function, has homology to Staphylococcus aureus nuclease; green fluorescent protein (GFP)-fusion protein localizes to mitochondria and is induced in response to the DNA-damaging agent MMS |
|  | *YNL092W* | *AFUA_1G15210* | Putative S-adenosylmethionine-dependent methyltransferase of the seven beta-strand family; YNL092W is not an essential gene |
|  | *YHR138C* | *AFUA_1G07500* | Putative protein of unknown function; has similarity to Pbi2p; double null mutant lacking Pbi2p and Yhr138p exhibits highly fragmented vacuoles |
|  | *YML020W* | *AFUA_5G12090* | Putative protein of unknown function |
|  | *YBR204C* | *AFUA_1G03540* | Serine hydrolase; YBR204C is not an essential gene |
|  | *YNL305C* | *AFUA_5G09310* | Putative protein of unknown function; green fluorescent protein (GFP)-fusion protein localizes to the vacuole; YNL305C is not an essential gene |
|  | *YJR111C* | *AFUA_5G13110* | Putative protein of unknown function; green fluorescent protein (GFP)-fusion protein localizes to the mitochondria |
|  | *YIR007W* | *AFUA_3G08820* | Putative protein of unknown function; green fluorescent protein (GFP)-fusion protein localizes to the cytoplasm; YIR007W is a non-essential gene |
|  | *YPR098C* | *AFUA_2G02630* | Protein of unknown function, localized to the mitochondrial outer membrane |
|  | *YHR009C* | *AFUA_6G04220* | Putative protein of unknown function; not an essential gene |
|  | *YLR456W YPR172W* | *AFUA_3G06670* | Putative protein of unknown function; null mutant displays increased resistance to antifungal agents gliotoxin, cycloheximide and H2O2 |
|  | *YLL023C* | *AFUA_7G05380* | Protein of unknown function; highly conserved across species and orthologous to human TMEM33; may interact with ribosomes, based on co-purification experiments; GFP-fusion protein localizes to the endoplasmic reticulum |
|  | *YPL067C* | *AFUA_2G03780 AFUA_2G14070* | Putative protein of unknown function; green fluorescent protein (GFP)-fusion protein localizes to the cytoplasm; YPL067C is not an essential gene |
|  | *YOR389W YPL277C* | *AFUA_2G01940* | Putative protein of unknown function; localized to the membranes; gene expression regulated by copper levels |
|  | *YLR108C YDR132C* | *AFUA_1G17100* | Protein of unknown function; green fluorescent protein (GFP)-fusion protein localizes to the nucleus; YLR108C is not an esssential gene |
|  | *YGL159W* | *AFUA_1G04440 AFUA_6G08780* | Putative protein of unknown function; deletion mutant has no detectable phenotype |
|  | *YJR116W* | *AFUA_1G06320* | Putative protein of unknown function |
|  | *YOR390W YPL279C* | *AFUA_2G16210* | Putative protein of unknown function |
|  | *YOR022C* | *AFUA_2G07430* | Protein with similarity to bovine phospholipase A1; the authentic, non-tagged protein is detected in highly purified mitochondria in high-throughput studies |
|  | *YKR051W* | *AFUA_2G13512* | Putative protein of unknown function |
|  | *YBR220C* | *AFUA_3G07290* | Putative protein of unknown function; YBR220C is not an essential gene |
|  | *YDR109C* | *AFUA_4G04680* | FGGY-family carbohydrate kinase |
|  | *YBR242W YGL101W* | *AFUA_2G11680* | Putative protein of unknown function; green fluorescent protein (GFP)-fusion protein localizes to the cytoplasm and nucleus; YBR242W is not an essential gene |
|  | *YIL067C* | *AFUA_5G02970 AFUA_5G08630* | Uncharacterized protein of unknown function |
|  | *YHR202W* | *AFUA_3G04160* | Putative protein of unknown function; green fluorescent protein (GFP)-fusion protein localizes to the vacuole, while HA-tagged protein is found in the soluble fraction, suggesting cytoplasmic localization |
|  | *YFL034W* | *AFUA_1G03760* | Putative integral membrane protein that interacts with Rpp0p, which is a component of the ribosomal stalk |
|  | *YMR027W* | *AFUA_5G06710* | Putative protein of unknown function; green fluorescent protein (GFP)-fusion protein localizes to the nucleus and cytoplasm; YMR027W is not an essential gene |
|  | *YOL057W* | *AFUA_4G06140* | Dipeptidyl-peptidase III; cleaves dipeptides from the amino terminus of target proteins; highly active on synthetic substrate Arg-Arg-2-naphthylamide; mammalian ortholog may be a biomarker for some cancers |
|  | *YMR221C* | *AFUA_4G03740 AFUA_7G01670* | Putative protein of unknown function; the authentic, non-tagged protein is detected in highly purified mitochondria in high-throughput studies; physical interaction with Atg27p suggests a possible role in autophagy |
|  | *YBL028C* | *AFUA_6G05250* | Protein of unknown function that may interact with ribosomes; green fluorescent protein (GFP)-fusion protein localizes to the nucleolus; predicted to be involved in ribosome biogenesis |
|  | *YML003W* | *AFUA_1G09870* | Putative protein of unknown function |
|  | *YPR010C-A* | *AFUA_2G13000* | Putative protein of unknown function; conserved among Saccharomyces sensu stricto species |
|  | *YBL029C-A* | *AFUA_1G04860* | Protein of unknown function; green fluorescent protein (GFP)-fusion protein localizes to the cell periphery; has potential orthologs in Saccharomyces species and in Yarrowia lipolytica |
|  | *YLR050C* | *AFUA_7G06040* | Putative protein of unknown function; green fluorescent protein (GFP)-fusion protein localizes to the endoplasmic reticulum; YLR050C is not an essential gene |
|  | *YJR056C* | *AFUA_4G13470* | Putative protein of unknown function; green fluorescent protein (GFP)-fusion protein localizes to both the cytoplasm and the nucleus |
|  | *YPL041C* | *AFUA_5G03485* | Protein of unknown function involved in maintenance of proper telomere length |
|  | *YLR173W* | *AFUA_4G07200* | Putative protein of unknown function |
|  | *YIL102C-A* | *AFUA_1G03020* | Putative protein of unknown function, identified based on comparisons of the genome sequences of six Saccharomyces species |
|  | *YGR042W* | *AFUA_5G07680* | Putative protein of unknown function; involved in maintenance of proper telomere length; green fluorescent protein (GFP)-fusion protein localizes to both the cytoplasm and the nucleus |
|  | *YDR374C* | *AFUA_7G03780* | YT521-B-like splicing factor |
|  | *YMR074C* | *AFUA_3G06420* | Protein with homology to human PDCD5, which is involved in programmed cell death; N-terminal region forms a conserved triple-helix bundle structure; overexpression promotes H2O2-induced apoptosis; YMR074C is not an essential gene |
|  | *YLR132C* | *AFUA_8G05110* | Essential protein of unknown function; fluorescent protein (GFP or YFP)-tagged protein localizes to mitochondria, cytoplasm and nucleus |
|  | *YOL086W-A* | *AFUA_6G10965* | Putative protein of unknown function; mutant in a srs2 mutant background displays MMS hypersensitivity; ortholog of human MHF1, a component of the Fanconi anemia complex that is involved in maintaining genome stability |
|  | *YLR137W* | *AFUA_2G09930* | Putative S-adenosylmethionine-dependent methyltransferase |
|  | *YKL162C* | *AFUA_8G05210* | Putative protein of unknown function; green fluorescent protein (GFP)-fusion protein localizes to the mitochondrion |
|  | *YOR228C* | *AFUA_3G04190* | Protein of unknown function, localized to the mitochondrial outer membrane |
|  | *YHR192W* | *AFUA_4G02920* | Putative protein of unknown function; green fluorescent protein (GFP)-fusion protein localizes to the cytoplasm and is induced in response to the DNA-damaging agent MMS |
|  | *YDR262W* | *AFUA_8G05360* | Putative protein of unknown function; green fluorescent protein (GFP)-fusion protein localizes to the vacuole and is induced in response to the DNA-damaging agent MMS; gene expression increases in response to Zymoliase treatment |
|  | *YDR286C* | *AFUA_1G11535* | Putative protein of unknown function; predicted to have thiol-disulfide oxidoreductase active site |
|  | *YBL107C* | *AFUA_4G11920* | Putative protein of unknown function; green fluorescent protein (GFP)-fusion protein localizes to the cytoplasm; YBL107C is not an essential gene |
|  | *YNL260C* | *AFUA_1G09000* | Putative protein of unknown function with similarity to a human protein overexpressed in oral cancers; essential gene with defects in anaerobic and filamentous growth; localizes to the nucleus and cytoplasm |
|  | *YOR238W* | *AFUA_3G14300* | Putative protein of unknown function; green fluorescent protein (GFP)-fusion protein localizes to the cytoplasm |
|  | *YHR045W* | *AFUA_5G09510* | Putative protein of unknown function; possible role in iron metabolism and/or amino acid and carbohydrate metabolism; green fluorescent protein (GFP)-fusion protein localizes to the endoplasmic reticulum |
|  | *YNL193W* | *AFUA_5G04150* | Putative protein of unknown function; exhibits a two-hybrid interaction with Yhr151cp in a large-scale analysis |
|  | *YBR137W* | *AFUA_2G04610* | Protein of unknown function; localized to the cytoplasm; binds to Replication Protein A (RPA); YBR137W is not an essential gene |
|  | *YLR271W* | *AFUA_8G04200* | Putative protein of unknown function; green fluorescent protein (GFP)-fusion protein localizes to the cytoplasm and the nucleus and is induced in response to the DNA-damaging agent MMS |
|  | *YLR104W* | *AFUA_5G04040* | Putative protein of unknown function; mutant is deficient in amounts of cell wall mannosylphosphate; genetic interactions suggest a role in ER-associated protein degradation (ERAD) |
|  | *YGR235C* | *AFUA_6G08630* | Putative protein of unknown function; the authentic, non-tagged protein is detected in highly purified mitochondria in high-throughput studies |
|  | *YBR053C* | *AFUA_4G08610* | Putative protein of unknown function; induced by cell wall perturbation |
|  | *YCR016W* | *AFUA_3G11310* | Putative protein of unknown function; green fluorescent protein (GFP)-fusion protein localizes to the nucleolus and nucleus; predicted to be involved in ribosome biogenesis |
|  | *YPR170W-B* | *AFUA_5G06150* | Putative protein of unknown function, conserved in fungi; partially overlaps the dubious genes YPR169W-A, YPR170W-A and YRP170C |
|  | *YLR326W* | *AFUA_5G12410* | Putative protein of unknown function, predicted to be palmitoylated |
|  | *YJR085C* | *AFUA_1G03720* | Putative protein of unknown function; GFP-fusion protein is induced in response to the DNA-damaging agent MMS; the authentic, non-tagged protein is detected in highly purified mitochondria in high-throughput studies |
|  | *YER010C* | *AFUA_4G10940* | Protein of unknown function, forms a ring-shaped homotrimer; has similarity to members of the prokaryotic rraA family; possibly involved in a phosphotransfer reaction |
|  | *YLR099W-A* | *AFUA_3G08590* | Putative protein of unknown function |
|  | *YGR111W* | *AFUA_2G15900* | Putative protein of unknown function; green fluorescent protein (GFP)-fusion protein localizes to both the cytoplasm and the nucleus |
|  | *YER134C* | *AFUA_6G13100* | Putative protein of unknown function; non-essential gene |
|  | *YJR141W* | *AFUA_3G07970* | Essential protein of unknown function |
|  | *YAL044W-A* | *AFUA_7G01520* | Putative protein of unknown function; similar to S. pombe uvi31 which is a putative DNA repair protein |
|  | *YCL057C-A* | *AFUA_1G13195* | Putative protein of unknown function; the authentic, non-tagged protein is detected in highly purified mitochondria in high-throughput studies |
|  | *YFR016C* | *AFUA_2G15180* | Putative protein of unknown function; green fluorescent protein (GFP)-fusion protein localizes to the cytoplasm and bud; interacts with Spa2p; YFL016C is not an essential gene |
|  | *YGR127W* | *AFUA_2G09530* | Putative protein of unknown function; expression is regulated by Msn2p/Msn4p, indicating a possible role in stress response |
|  | *YNL155W* | *AFUA_4G04280* | Putative protein of unknown function, contains DHHC domain, also predicted to have thiol-disulfide oxidoreductase active site |
|  | *YDR161W* | *AFUA_4G12110* | Putative protein of unknown function; non-essential gene; proposed function in rRNA and ribosome biosynthesis based on transcriptional co-regulation; genetic interactions suggest a role in ER-associated protein degradation (ERAD) |
|  | *YGR205W* | *AFUA_4G03890* | ATP-binding protein of unknown function; crystal structure resembles that of E.coli pantothenate kinase and other small kinases |
|  | *YOL073C* | *AFUA_4G11680* | Putative protein of unknown function |
|  | *YOL087C* | *AFUA_5G11870* | Putative protein of unknown function; green fluorescent protein (GFP)-fusion protein localizes to the cytoplasm; deletion mutant is sensitive to various chemicals including phenanthroline, sanguinarine, and nordihydroguaiaretic acid |
|  | *YOR111W* | *AFUA_1G15580* | Acetylserotonin methytransferase-like protein (ASMTL), putative |
|  | *YPL107W* | *AFUA_4G04470* | Putative protein of unknown function; green fluorescent protein (GFP)-fusion protein localizes to mitochondria; YPL107W is not an essential gene |
|  | *YPR147C* | *AFUA_5G07840* | Putative protein of unknown function; green fluorescent protein (GFP)-fusion protein localizes to the cytoplasm and is induced in response to the DNA-damaging agent MMS |
|  | *YOR223W* | *AFUA_4G10700* | Putative protein of unknown function |
|  | *YCR090C* | *AFUA_2G15510* | Putative protein of unknown function; green fluorescent protein (GFP)-fusion protein localizes to the cytoplasm and nucleus; YCR090C is not an essential gene |
|  | *YOL114C* | *AFUA_3G09760* | Putative protein of unknown function with similarity to human ICT1 and prokaryotic factors that may function in translation termination; YOL114C is not an essential gene |
|  | *YNR021W* | *AFUA_4G09950* | Putative protein of unknown function; green fluorescent protein (GFP)-fusion protein localizes to the endoplasmic reticulum; YNR021W is not an essential gene |
|  | *YKL100C* | *AFUA_6G02150* | Putative protein of unknown function with similarity to a human minor histocompatibility antigen and signal peptide peptidases; YKL100C is not an essential gene |
|  | *YKR023W* | *AFUA_6G02200* | Putative protein of unknown function; the authentic, non-tagged protein is detected in highly purified mitochondria in high-throughput studies |
|  | *YIL096C* | *AFUA_5G05730* | Putative protein of unknown function; associates with precursors of the 60S ribosomal subunit; predicted to be involved in ribosome biogenesis |
|  | *YCR087C-A* | *AFUA_7G04700* | Putative protein of unknown function; green fluorescent protein (GFP)-fusion protein localizes to the nucleolus; YCR087C-A is not an essential gene |
|  | *YCR051W* | *AFUA_1G09700* | Putative protein of unknown function; green fluorescent protein repeats; YCR051W is not an essential gene |
|  | *YMR244C-A* | *AFUA_3G09780* | Putative protein of unknown function; green fluorescent protein (GFP)-fusion protein localizes to both the cytoplasm and nucleus and is induced in response to the DNA-damaging agent MMS; YMR244C-A is not an essential gene |
|  | *YCL047C* | *AFUA_4G07310* | Putative protein of unknown function |
|  | *YER163C* | *AFUA_3G08560* | Putative protein of unknown function; green fluorescent protein (GFP)-fusion protein localizes to the cytoplasm and nucleus; periodically expressed during the metabolic cycle; weak similarity to bacterial cation transport protein |
|  | *YOR289W* | *AFUA_3G09390* | Putative protein of unknown function; transcription induced by the unfolded protein response; green fluorescent protein (GFP)-fusion protein localizes to both the cytoplasm and the nucleus |
|  | *YDR333C* | *AFUA_1G06850* | Putative protein of unknown function |
|  | *YGR021W* | *AFUA_6G04090* | Putative protein of unknown function; the authentic, non-tagged protein is detected in highly purified mitochondria in high-throughput studies |
|  | *YMR265C* | *AFUA_3G09080* | Putative protein of unknown function |
|  | *YPR153W* | *AFUA_2G08400* | Putative protein of unknown function |
|  | *YGL010W* | *AFUA_8G05310* | Putative protein of unknown function; YGL010W is not an essential gene |
|  | *YDL133W* | *AFUA_1G12980* | Putative protein of unknown function |
|  | *YML079W* | *AFUA_5G11860* | Non-essential protein of unknown function with structural resemblance to plant storage and ligand binding proteins and to some enzymes (epimerase, germin); localizes to the nucleus and cytoplasm |
|  | *YLR063W* | *AFUA_1G13460* | Putative S-adenosylmethionine-dependent methyltransferase; green fluorescent protein (GFP)-fusion protein localizes to the cytoplasm; YLR063W is not an essential gene |
|  | *YMR185W* | *AFUA_6G10360* | Putative protein of unknown function; conflicting evidence on whether null mutant is viable with elongated buds, or inviable |
|  | *YBR141C* | *AFUA_5G05660* | Putative S-adenosylmethionine-dependent methyltransferase; green fluorescent protein (GFP)-fusion protein localizes to the nucleolus; YBR141C is not an essential gene |
|  | *YOL107W* | *AFUA_6G12610* | Putative protein of unknown function; green fluorescent protein (GFP)-fusion protein localizes to the cytoplasm and colocalizes in a punctate pattern with the early golgi/COPI vesicles; YOL107W is not an essential protein |
|  | *YPL162C* | *AFUA_2G05940* | Putative protein of unknown function; green fluorescent protein (GFP)-fusion protein localizes to the membrane of vacuole with cell cycle-correlated morphology |
|  | *YLR290C* | *AFUA_4G09810* | Putative protein of unknown function; the authentic, non-tagged protein is detected in highly purified mitochondria in high-throughput studies; YLR290C is not an essential gene |
|  | *YNL115C* | *AFUA_1G03090* | Putative protein of unknown function; green fluorescent protein (GFP)-fusion protein localizes to mitochondria; YNL115C is not an essential gene |
|  | *YML002W* | *AFUA_1G09870* | Putative protein of unknown function; expression induced by heat and by calcium shortage |
|  | *YHR122W* | *AFUA_8G03980* | Protein of unknown function required for establishment of sister chromatid cohesion; synthetically lethal with RFC5, an RF-C subunit that links replication to cohesion establishment; YHR122W is an essential gene |
|  | *YOR021C* | *AFUA_1G08950* | Putative protein of unknown function; YOR021C is not an essential gene; predicted to be involved in rRNA processing and ribosome biogenesis and in biopolymer catabolism |
|  | *YMR114C* | *AFUA_4G13150* | Protein of unknown function; may interact with ribosomes, based on co-purification experiments; green fluorescent protein (GFP)-fusion protein localizes to the nucleus and cytoplasm; YMR114C is not an essential gene |
|  | *YOR131C* | *AFUA_4G13290* | Putative protein of unknown function; non-essential gene; overexpression causes a cell cycle delay or arrest |
|  | *YNL200C* | *AFUA_5G08360* | Putative protein of unknown function; the authentic, non-tagged protein is detected in highly purified mitochondria in high-throughput studies |
|  | *YBL036C* | *AFUA_4G04300* | Putative non-specific single-domain racemase based on structural similarity; binds pyridoxal 5'-phosphate; expression of GFP-fusion protein induced in response to the DNA-damaging agent MMS |
|  | *YPL225W* | *AFUA_4G08810* | Protein of unknown function that may interact with ribosomes, based on co-purification experiments; green fluorescent protein (GFP)-fusion protein localizes to the cytoplasm |
|  | *YBL055C* | *AFUA_4G07010* | 3'-->5' exonuclease and endonuclease with a possible role in apoptosis; has similarity to mammalian and C. elegans apoptotic nucleases |
|  | *YJR008W* | *AFUA_2G03970* | Putative protein of unknown function; expression repressed by inosine and choline in an Opi1p-dependent manner; expression induced by mild heat-stress on a non-fermentable carbon source |
|  | *YJL055W* | *AFUA_6G04800* | Putative protein of unknown function, proposed to be involved in the metabolism of purine and pyrimidine base analogues; deletion mutants are sensitive to HAP and AHA; overexpression confers resistance to 5-FOA and 5-FU |
|  | *YKL151C* | *AFUA_2G10120* | Putative protein of unknown function; YKL151C promoter contains STREs and expression is induced by heat shock or methyl methanesulfonate; green fluorescent protein (GFP)-fusion protein localizes to the cytoplasm |
|  | *YPR045C* | *AFUA_4G04110* | Protein of unknown function; involvement in splicing based on pre-mRNA accumulation defect for many intron-containing genes; contains a SAC3 domain; physically interacts with Csn12p; YRP045C is not an essential gene |
|  | *YMR259C* | *AFUA_2G14180* | Putative protein of unknown function; green fluorescent protein (GFP)-fusion protein localizes to the cytoplasm; YMR259C is not an essential gene |
|  | *YMR178W* | *AFUA_5G11210* | Putative protein of unknown function; green fluorescent protein (GFP)-fusion protein localizes to both the cytoplasm and nucleus and is induced in response to the DNA-damaging agent MMS; YMR178W is not an essential gene |
|  | *YER140W* | *AFUA_2G04390* | Putative protein of unknown function; the authentic, non-tagged protein is detected in highly purified mitochondria in high-throughput studies; genetic interactions suggest a role in folding of ER membrane proteins |
|  | *YMR099C* | *AFUA_4G08880* | Possible apospory-associated protein c |
|  | *YDL156W* | *AFUA_6G12330* | Putative protein of unknown function; protein sequence contains three WD domains (WD-40 repeat); green fluorescent protein (GFP)-fusion protein localizes to the cytoplasm and nucleus |
|  | *YLR422W* | *AFUA_1G04340* | Protein of unknown function with similarity to human DOCK proteins (guanine nucleotide exchange factors); interacts with Ino4p; green fluorescent protein (GFP)-fusion protein localizes to the cytoplasm, YLR422W is not an essential protein |
|  | *YER156C* | *AFUA_1G08980* | Putative protein of unknown function; interacts with Hsp82p and copurifies with Ipl1p; expression is copper responsive and downregulated in strains deleted for MAC1, a copper-responsive transcription factor; similarity to mammalian MYG1 |
|  | *YGR207C* | *AFUA_5G07300* | Protein that interacts with frataxin (Yfh1p); putative homolog of mammalian electron transfer flavoprotein complex subunit ETF-beta; authentic, non-tagged protein is detected in highly purified mitochondria in high-throughput studies |
|  | *YDR266C* | *AFUA_1G02010* | Protein of unknown function that may interact with ribosomes, based on co-purification experiments;green fluorescent protein (GFP)-fusion protein localizes to the cytoplasm; contains a RING finger domain |
|  | *YJR098C* | *AFUA_7G04510* | Putative protein of unknown function; the authentic, non-tagged protein is detected in highly purified mitochondria in high-throughput studies |
|  | *YIL001W* | *AFUA_6G02680* | Putative protein of unknown function; contains a BTB/POZ domain which generally function in protein interactions; deletion slightly improved competitive fitness in rich media; GFP-tagged protein is localized to the cytoplasm |
|  | *YPR091C* | *AFUA_3G06130* | Putative protein of unknown function; may interact with ribosomes, based on co-purification experiments; green fluorescent protein (GFP)-fusion protein localizes to the endoplasmic reticulum; YPR091C is not an essential gene |
|  | *YNL011C* | *AFUA_5G12650* | Putative protein of unknown function; YNL011C is not an essential gene |
|  | *YGR149W* | *AFUA_3G08240* | Putative protein of unknown function; predicted to be an integal membrane protein |
|  | *YPR097W* | *AFUA_3G08020* | Protein that contains a Phox homology domain and binds phosphoinositides; the authentic, non-tagged protein is detected in highly purified mitochondria in high-throughput studies |
|  | *YNL313C* | *AFUA_7G05600* | Essential protein of unknown function; fluorescent protein (GFP or YFP)-tagged protein localizes to cytoplasm and nucleus |
|  | *YDL086W* | *AFUA_1G07130* | Putative protein of unknown function; the authentic, non-tagged protein is detected in highly purified mitochondria in high-throughput studies; YDL086W is not an essential gene |
|  | *YIL108W* | *AFUA_3G11160* | Zinc metalloproteinase |
|  | *YIL091C* | *AFUA_1G09320* | Nucleolar protein that cosediments with the 40S ribosomal subunit and is required for its biogenesis; proposed to function as an RNA helicase based on structure prediction and remote homology searches |
|  | *YPL009C* | *AFUA_2G09170* | Protein of unknown function; may interact with ribosomes, based on co-purification experiments; green fluorescent protein (GFP)-fusion protein localizes to the cytoplasm; YPL009C is not an essential gene |
|  | *YGL114W* | *AFUA_1G13620* | Putative protein of unknown function; predicted member of the oligopeptide transporter family of membrane transporters |
|  | *YOR093C* | *AFUA_1G15010* | Putative protein of unknown function; deletion causes sensitivity to unfolded protein response-inducing agents |
